# Supplementary material for: Neural Bayes estimation and selection of complex bivariate extremal dependence models
Source: Extremes (Boston). 2025 Dec 5;29(2):347–86. doi: 10.1007/s10687-025-00521-8 (PMC13263241; doi:10.1007/s10687-025-00521-8)
Supplement: Supplementary file 1 — (zip 5334 KB) [file 10687_2025_521_MOESM1_ESM.zip › Supplementary/supplementary.pdf]

Supplementary Material for *Neural Bayes estimation and selection of complex bivariate extremal dependence models*

L. M. André[1*], J. L. Wadsworth[2], R. Huser[3]

[1] Namur Institute for Complex Systems, University of Namur, Rue Grafé 2, Namur 5000, Belgium

[2] School of Mathematical Sciences, Lancaster University, LA1 4YF, United Kingdom

[3] Statistics Program, Computer, Electrical and Mathematical Sciences and Engineering Division, King Abdullah University of Science and Technology (KAUST), Saudi Arabia

[*] Correspondence to: lidiamandre@gmail.com

October 20, 2025

S1 DeepSets architecture

A schematic of the DeepSets architecture (recall Section 2.1 of the main paper) used is shown in Figure S1. This is based on Sainsbury-Dale et al. (2024).

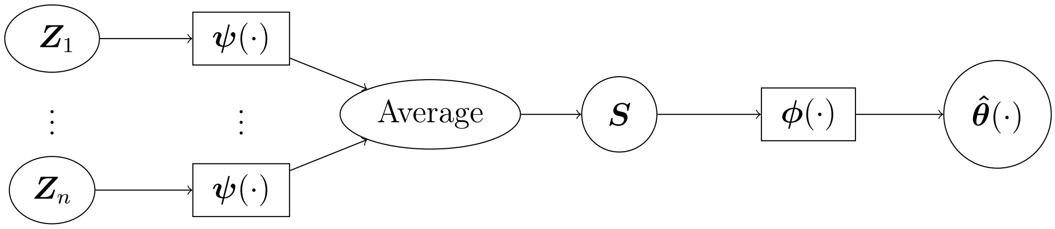

Figure S1: In the first step, the data inputs $\boldsymbol{Z}_1, \ldots, \boldsymbol{Z}_n$ are transformed independently through neural network $\boldsymbol{\psi}(\cdot)$, They are then aggregated through the elementwise average, obtaining the summary statistic $\boldsymbol{S}$. In the last step, neural network $\boldsymbol{\phi}(\cdot)$ maps the summary statistic $\boldsymbol{S}$ to an estimate of the vector of model parameters $\hat{\boldsymbol{\theta}}(\cdot)$.

Table S1: Summary of the neural network architecture used to train the NBE. The input array to the first layer represents the dimension $d = 2$ of data set $\boldsymbol{Z}$; this differs for uncensored and censored data. For the censored case, a bilinear layer is used instead, and an extra dimension for the indicator vector $\boldsymbol{I}$ is needed. In addition, the input layer of $\boldsymbol{\phi}(\cdot)$ has an extra dimension in the case of censored data with random censoring level τ. The output array $[p]$ of the last layer represents the number of parameters in the model.

Neural network	Input dimension	Output dimension
$\boldsymbol{\psi}(\cdot)$	$[2]$ or $[2, 2]$	$[128]$
	$[128]$	$[128]$
	$[128]$	$[256]$
$\boldsymbol{\phi}(\cdot)$	$[256]$ or $[257]$	$[128]$
	$[128]$	$[p]$

S2 Parameter estimation assessment

In this section, we present the simulation studies done for the remaining models considered in this work. In Section S2.1, we show the performance of the NBEs for uncensored data in five configurations of the weighted copula model (WCM) from Section 3.4 of the main paper. Where feasible, a comparison with maximum likelihood inference is presented. In Section S2.2, we show the performance of the NBEs when the sample size and censoring level are kept fixed, and when the sample size is assumed variable but the censoring level is still fixed for Model W. Finally, in Sections S2.3, S2.4 and S2.5, we present the results for the remaining three models from Section 3.3 of the main paper. In all of these cases, a comparison with censored maximum likelihood estimation is given, wherein the censoring scheme used is that of the main paper (i.e., outlined in Wadsworth et al. (2017)).

The neural network architecture used for parameter estimation (recall Section 4.2 of the main paper) is given in Table S1.

S2.1 Weighted copula model

We consider now five additional configurations of the WCM. For the first two models, we assume c_b and c_t to be one-parameter copulas, while for the remaining three configurations (Sections S2.1.3, S2.1.4 and S2.1.5) c_b is assumed to be a Gaussian copula, and c_t is one of the flexible copulas mentioned in Section 3.3 of the main paper. For these three models configurations, the likelihood is infeasible and hence no comparison with MLE is provided. In all the models, we take $\pi(x_1, x_2; \gamma) = (x_1x_2)^\gamma$ as the weighting function. Since preliminary analysis indicated that the neural network was struggling to learn γ, we set $\kappa = \log \gamma$ and estimate κ instead. Lastly, the model-based $\chi(u)$ estimates of the

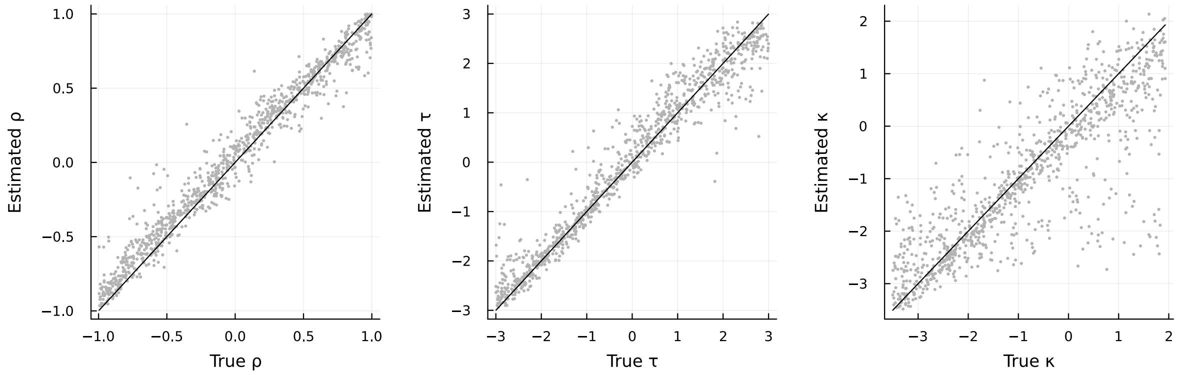

Figure S2: Assessment of the NBE when c_b is a Gaussian copula with correlation parameter ρ, c_t is a logistic copula with parameter $\tau_L = \text{logit}(\alpha_L)$, and with weighting function $\pi(x_1, x_2; \kappa) = (x_1 x_2)^{\exp(\kappa)}$, $x_1, x_2 \in (0, 1)$ for a sample size of $n = 1000$.

WCM are obtained using a Monte Carlo approximation with 500 000 samples.

S2.1.1 Model 1: c_b is a Gaussian copula and c_t is a logistic copula

For the first model, we consider the copula tailored to the body c_b to be a Gaussian copula with correlation parameter $\rho \in (-1, 1)$, and the copula tailored to the tail c_t to be a logistic copula with $\alpha_L \in (0, 1]$. Similarly to the weighting function parameter γ, we take an alternative parameterisation and set $\tau_L = \text{logit}(\alpha_L)$. Additionally, we set $\rho \sim \text{Unif}(-1, 1)$, $\tau_L \sim \text{Unif}(-3, 3)$, which results in $\alpha_L \in (0.05, 0.95)$, and $\kappa \sim \text{Unif}(-3.51, 1.95)$, which leads to $\gamma \in (-0.03, 7.03)$, as the priors for the parameters. The performance of the NBE is assessed in Figure S2 where the true values of the parameters are compared with their estimated values. It can be seen that parameter κ exhibits a bit of variability, while parameters ρ and τ_L are estimated quite well via the NBE. The coverage probabilities and average length of the 95% uncertainty intervals obtained via a non-parametric bootstrap procedure (as described in Section 4.2 of the main paper) are shown in Table S2. Similarly to the main paper, we compute the coverage probabilities of 95% uncertainty intervals, and their average length, for $\chi(u)$ at levels $u = \{0.50, 0.80, 0.95\}$; the results are shown on the right of Table S2. According to these results, the true $\chi(u)$ is within the confidence intervals in more than 77% of the time, which suggest that this measure is well derived from the NBE.

Comparison with maximum likelihood estimation

Since the likelihood of this model is feasible, though computationally intensive, we compare the estimations obtained by the NBE to the MLEs. With the assigned priors, we

Table S2: Coverage probability and average length of the 95% uncertainty intervals for the parameters (left) and for $\chi(u)$ at levels $u = \{0.50, 0.80, 0.95\}$ (right) obtained via a non-parametric bootstrap procedure averaged over 1000 models fitted using a NBE (rounded to 2 decimal places).

Parameter	Coverage	Length
ρ	0.71	0.26
τ_L	0.75	0.76
κ	0.69	1.33

$\chi(u)$	Coverage	Length
$\chi(0.50)$	0.77	0.05
$\chi(0.80)$	0.79	0.08
$\chi(0.95)$	0.78	0.09

generate five different parameter vectors $\boldsymbol{\theta} = (\rho, \tau_L, \kappa)'$ and corresponding data sets, each of which with $n = 1000$. Additionally, each data set is simulated 100 times. The results are shown in Figure S3; it can be seen that the NBE estimates are generally more biased, and sometimes more variable, than the MLEs. However, they are less likely to have big outliers as the neural network is trained in a bounded interval. Despite slightly more biased, the estimates obtained with the NBE are generally good. Furthermore, it is substantially faster to obtain an estimate through NBE than through maximum likelihood. In particular, on average, the MLE took 3 hours and 12 minutes to evaluate, while the NBE took 0.653 seconds; this means that the NBE is about 17 663 times faster—a substantial improvement in computational time.

S2.1.2 Model 2: c_b is a Frank copula and c_t is a Joe copula

For the second model, we consider c_b to be a Frank copula (Frank, 1979) with parameter $\beta_F \in \mathbb{R}$, and c_t to be a Joe copula (Joe, 1996) with $\alpha_J > 1$. As priors for the model parameters, we take $\beta_F \sim \text{Unif}(-15, 15)$, $\alpha_J \sim \text{Unif}(1, 15)$ and $\kappa \sim \text{Unif}(-3.51, 1.95)$. The performance of the NBE is assessed in Figure S4 where the true values of the parameters are compared with their estimated values. It can be seen that all the parameters are estimated quite well with the NBE, with β_F and α_J showing a bit of variability for lower and higher values, respectively. The coverage probabilities and average length of the 95% uncertainty intervals obtained via a non-parametric bootstrap procedure for the parameter estimates and for $\chi(u)$ at levels $u \in \{0.50, 0.80, 0.95\}$ are shown in Table S3. The lower coverage rates given on the left table reflect the bias shown by the parameter estimates. However, the results for $\chi(u)$ suggest that the NBE is able to capture the dependence structure of the data, especially for higher u, with the true $\chi(u)$ being within the confidence intervals in more than 59% of the time.

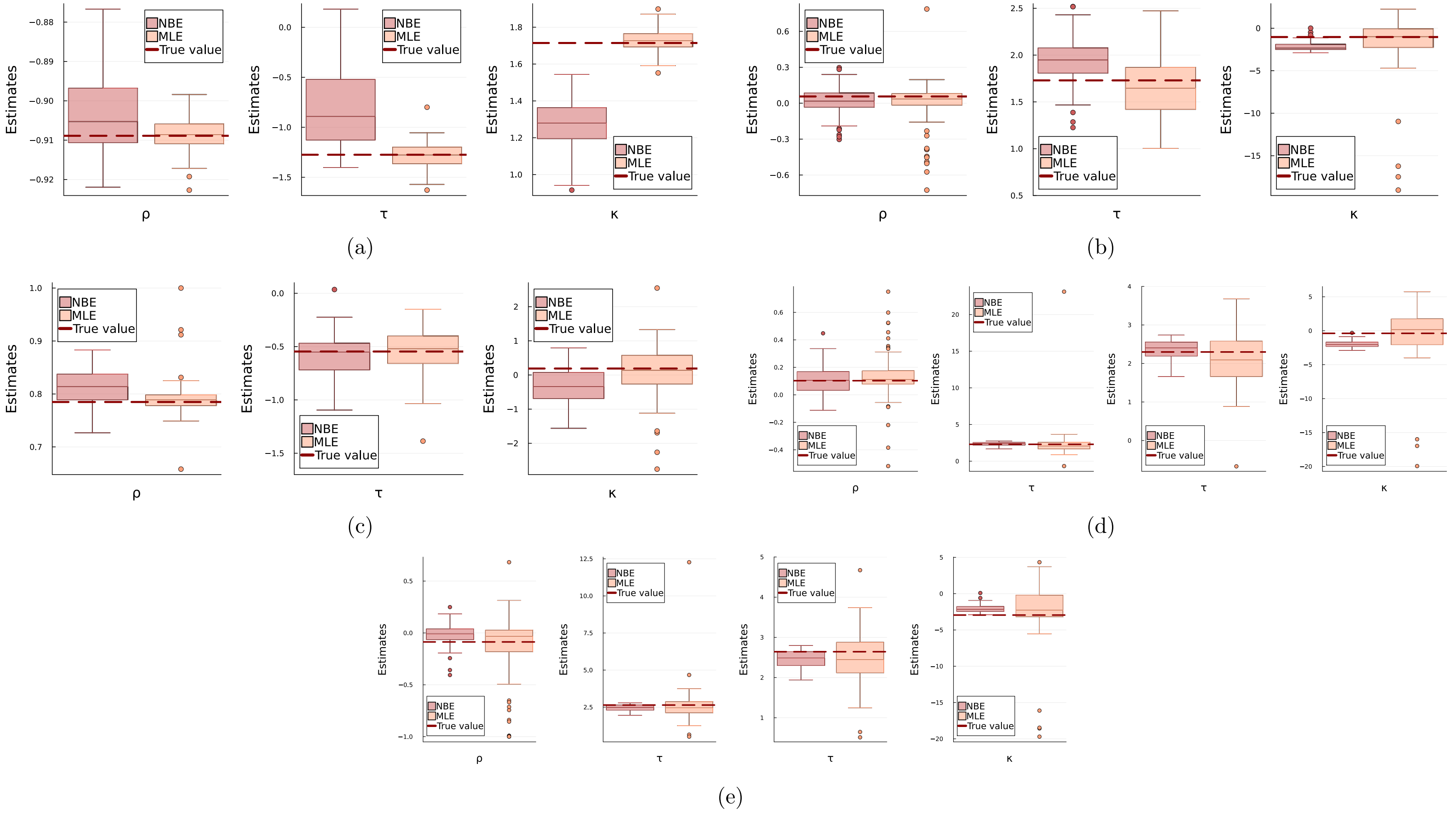

Figure S3: Comparison between parameter estimates $\hat{\boldsymbol{\theta}} = (\hat{\rho}, \hat{\tau}_L, \hat{\kappa})'$ given by MLE (orange) and by NBE (red) for 100 samples with $n = 1000$. The true parameter values are given by the red line. (a) $\boldsymbol{\theta} = (0.91, -1.27, 1.71)'$, (b) $\boldsymbol{\theta} = (0.91, 1.73, -1.03)'$, (c) $\boldsymbol{\theta} = (0.91, -0.55, 0.19)'$, (d) $\boldsymbol{\theta} = (0.91, 2.30, -0.38)'$ and (e) $\boldsymbol{\theta} = (0.91, 2.64, -2.95)'$. For better visualisation, the larger outliers obtained through MLE were removed for $\hat{\tau}_L$ in (d) and (e).

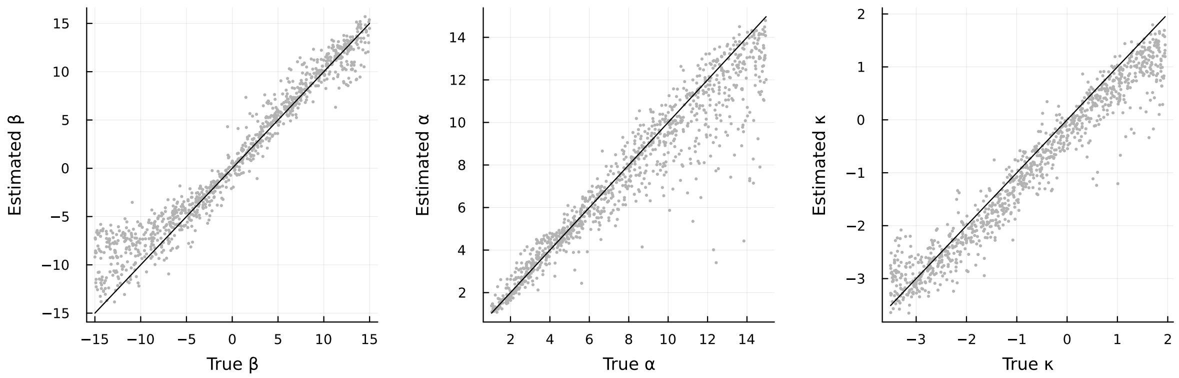

Figure S4: Assessment of the NBE when c_b is a Frank copula with parameter β, c_t is a Joe copula with parameter α_J, and with weighting function $\pi(x_1, x_2; \kappa) = (x_1 x_2)^{\exp(\kappa)}$, $x_1, x_2 \in (0, 1)$ for a sample size of $n = 1000$.

Table S3: Coverage probability and average length of the 95% uncertainty intervals for the parameters (left) and for $\chi(u)$ at levels $u = \{0.50, 0.80, 0.95\}$ (right) obtained via a non-parametric bootstrap procedure averaged over 1000 models fitted using a NBE (rounded to 2 decimal places).

Parameter	Coverage	Length
β_F	0.68	3.03
α_J	0.72	1.95
κ	0.62	0.92

$\chi(u)$	Coverage	Length
$\chi(0.50)$	0.61	0.05
$\chi(0.80)$	0.59	0.06
$\chi(0.95)$	0.64	0.06

Comparison with maximum likelihood estimation

For this model the likelihood is also feasible (and computational expensive). Therefore, as before, we compare the estimations obtained by the NBE and by the MLE for five different parameter vectors $\boldsymbol{\theta} = (\beta_F, \alpha_J, \kappa)'$, generated with the pre-specified priors, and corresponding data sets (each with $n = 1000$). Again, each data set is simulated 100 times; the results are shown in Figure S5. Similarly to the first model, the NBE estimates are generally more biased than the MLEs, are less prone to have large outliers, and are generally good. While, on average, the MLE took 52 minutes to evaluate, the NBE took 0.203 seconds, which is about 15 339 times faster.

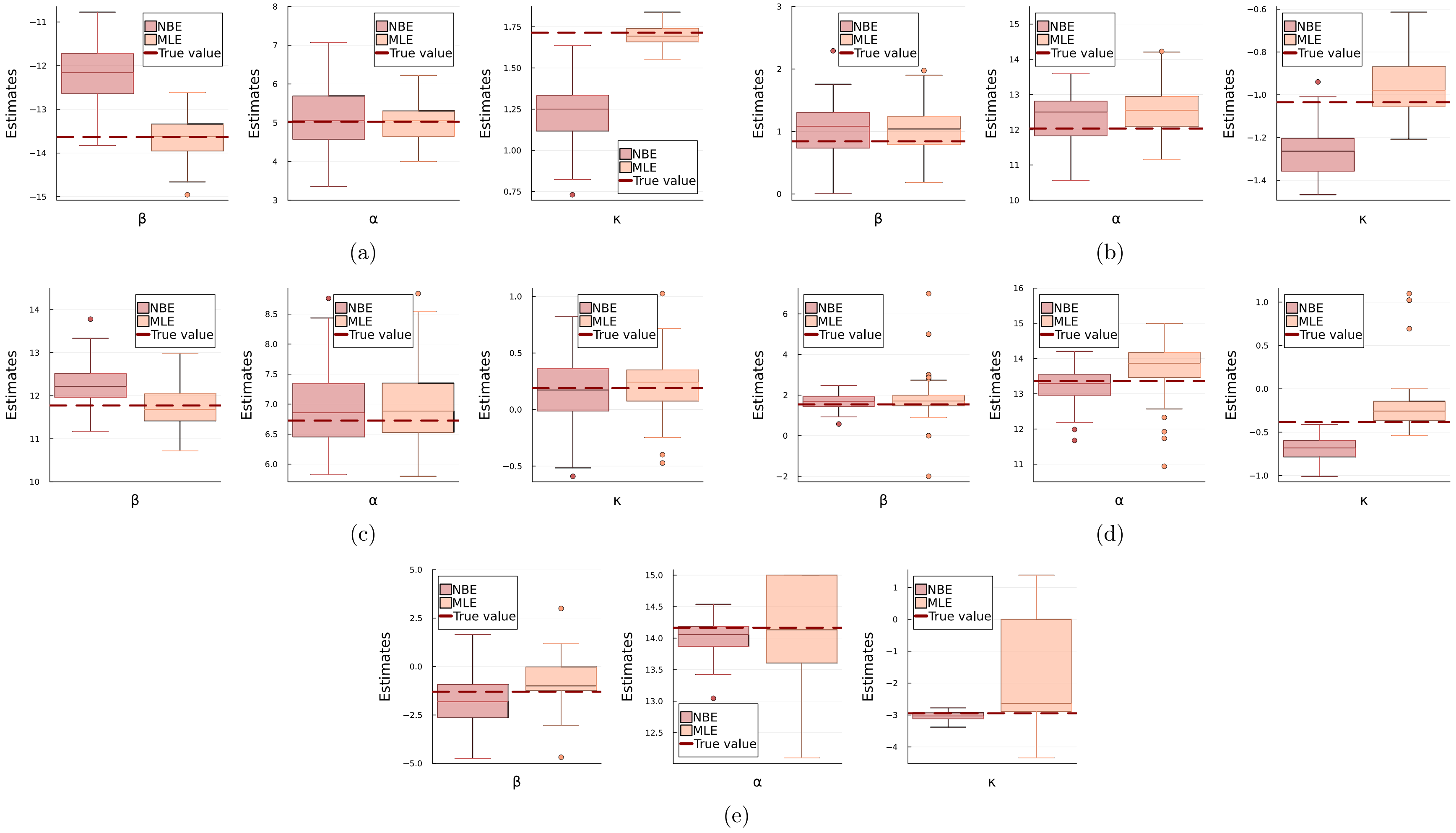

Figure S5: Comparison between parameter estimates $\hat{\boldsymbol{\theta}} = (\hat{\beta}_F, \hat{\alpha}_J, \hat{\kappa})'$ given by MLE (orange) and by NBE (red) for 100 samples with $n = 1000$. The true parameter values are given by the red line. (a) $\boldsymbol{\theta} = (-13.63, 5.02, 1.71)'$, (b) $\boldsymbol{\theta} = (0.84, 12.04, -1.03)'$, (c) $\boldsymbol{\theta} = (11.77, 6.73, 0.19)'$, (d) $\boldsymbol{\theta} = (1.54, 13.36, -0.38)'$ and (e) $\boldsymbol{\theta} = (-1.30, 14.17, -2.95)'$.

Table S4: Coverage probability and average length of the 95% uncertainty intervals for the parameters (left) and for $\chi(u)$ at levels $u = \{0.50, 0.80, 0.95\}$ (right) obtained via a non-parametric bootstrap procedure averaged over 1000 models fitted using a NBE (rounded to 2 decimal places).

Parameter	Coverage	Length
ρ	0.85	0.24
α	0.60	4.28
ξ	0.71	0.56
κ	0.73	1.16

$\chi(u)$	Coverage	Length
$\chi(0.50)$	0.91	0.06
$\chi(0.80)$	0.89	0.09
$\chi(0.95)$	0.85	0.11

S2.1.3 Model 3: c_b is a Gaussian copula and c_t is Model W

For the third model, we consider c_t to be Model W, for which the priors for the parameters are those mentioned in Section 4.1 from the main paper. Figure S6 displays the performance of the NBE. Despite the variability shown, especially by α and κ, the NBE provides good estimates overall. The coverage probabilities and average length of the 95% uncertainty intervals for the parameters and for $\chi(u)$ at levels $u = \{0.50, 0.80, 0.95\}$ obtained via a non-parametric bootstrap procedure are given in Table S4 on the left and right, respectively. The results for the parameter uncertainty are in agreement with Figure S6, where the coverage probability for α is the lowest and its average length the highest. However, as shown by the coverage probabilities for $\chi(u)$, this bias does not affect this dependence quantity. More specifically, the true value is within the confidence intervals in more than 85% of the time.

S2.1.4 Model 4: c_b is a Gaussian copula and c_t is Model HW

For the forth model, we consider c_t to be Model HW with the priors for the model parameters mentioned in Section 4.1 from the main paper. Figure S7 displays the performance of the NBE, showing that δ and ω seem to be over-estimated by the NBE for lower values. Table S5 shows the coverage probabilities and average length of the 95% uncertainty intervals obtained via a non-parametric bootstrap procedure for the parameters on the left, and for $\chi(u)$ at levels $u = \{0.50, 0.80, 0.95\}$ on the right. The results for the parameter estimates mirror the variability shown in Figure S7, where the coverage probabilities for ω and δ are the lowest. The coverage probabilities of the 95% uncertainty intervals for $\chi(u)$ show that the true value is within the confidence intervals in more than 86% of the time, indicating that despite the bias shown by the estimation, this dependence measure is well calibrated.

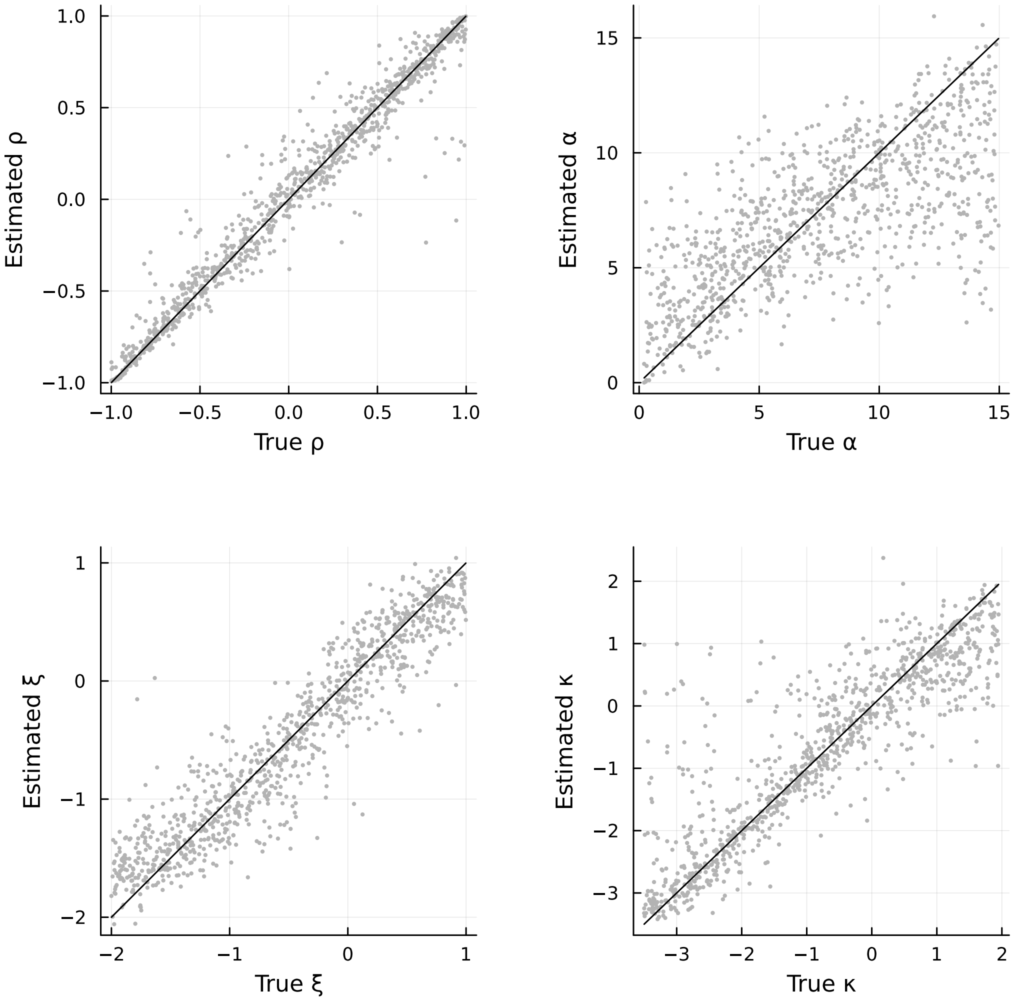

Figure S6: Assessment of the NBE when c_b is a Gaussian copula with correlation parameter ρ, c_t is Model W with parameters $(\alpha, \xi)'$ and with weighting function $\pi(x_1, x_2; \kappa) = (x_1 x_2)^{\exp(\kappa)}$, $x_1, x_2 \in (0, 1)$ for a sample size of $n = 1000$.

Table S5: Coverage probability and average length of the 95% uncertainty intervals for the parameters (left) and for $\chi(u)$ at levels $u = \{0.50, 0.80, 0.95\}$ (right) obtained via a non-parametric bootstrap procedure averaged over 1000 models fitted using a NBE (rounded to 2 decimal places).

Parameter	Coverage	Length
ρ	0.80	0.28
δ	0.58	0.15
ω	0.44	0.47
κ	0.71	1.33

$\chi(u)$	Coverage	Length
$\chi(0.50)$	0.91	0.07
$\chi(0.80)$	0.88	0.10
$\chi(0.95)$	0.86	0.12

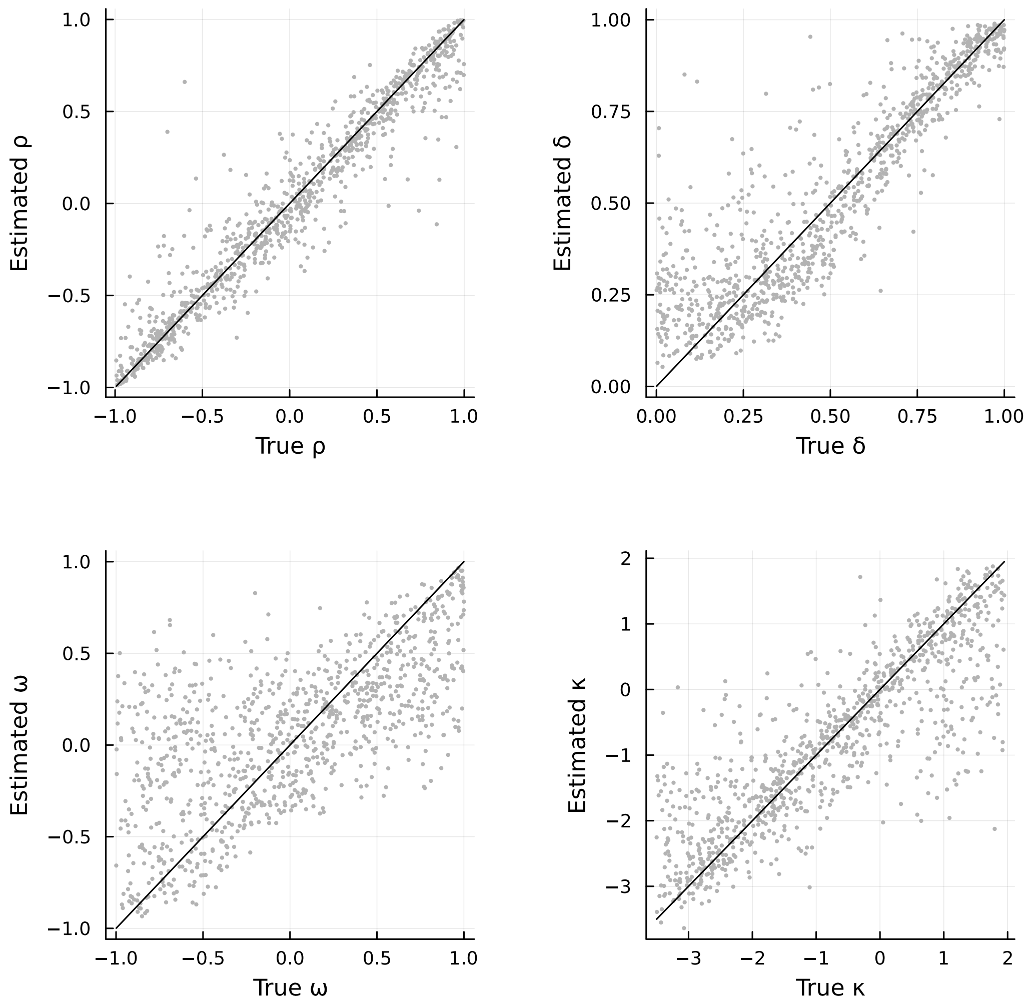

Figure S7: Assessment of the NBE when c_b is a Gaussian copula with correlation parameter ρ, c_t is Model HW with parameters $(\delta, \omega)'$, and with weighting function $\pi(x_1, x_2; \kappa) = (x_1 x_2)^{\exp(\kappa)}$, $x_1, x_2 \in (0, 1)$ for a sample size of $n = 1000$.

S2.1.5 Model 5: c_b is a Gaussian copula and c_t is Model E2

For the final model, we take c_t to be Model E2 with the priors for the model parameters mentioned in Section 4.1 from the main paper. Figure S8 shows the performance of the NBE. Similarly to Model 3, there is some variability in the NBEs, especially for α. This parameter is also the one with lowest coverage probability and wider interval for the parameters estimation procedure, as shown in left of Table S6. Similarly to the previous models, the coverage probabilities for $\chi(u)$ at levels $u = \{0.50, 0.80, 0.95\}$, shown in the right of Table S6, indicate that this measure is well captured by the NBE, with the true value lying within the confidence intervals in at least 83% of the time.

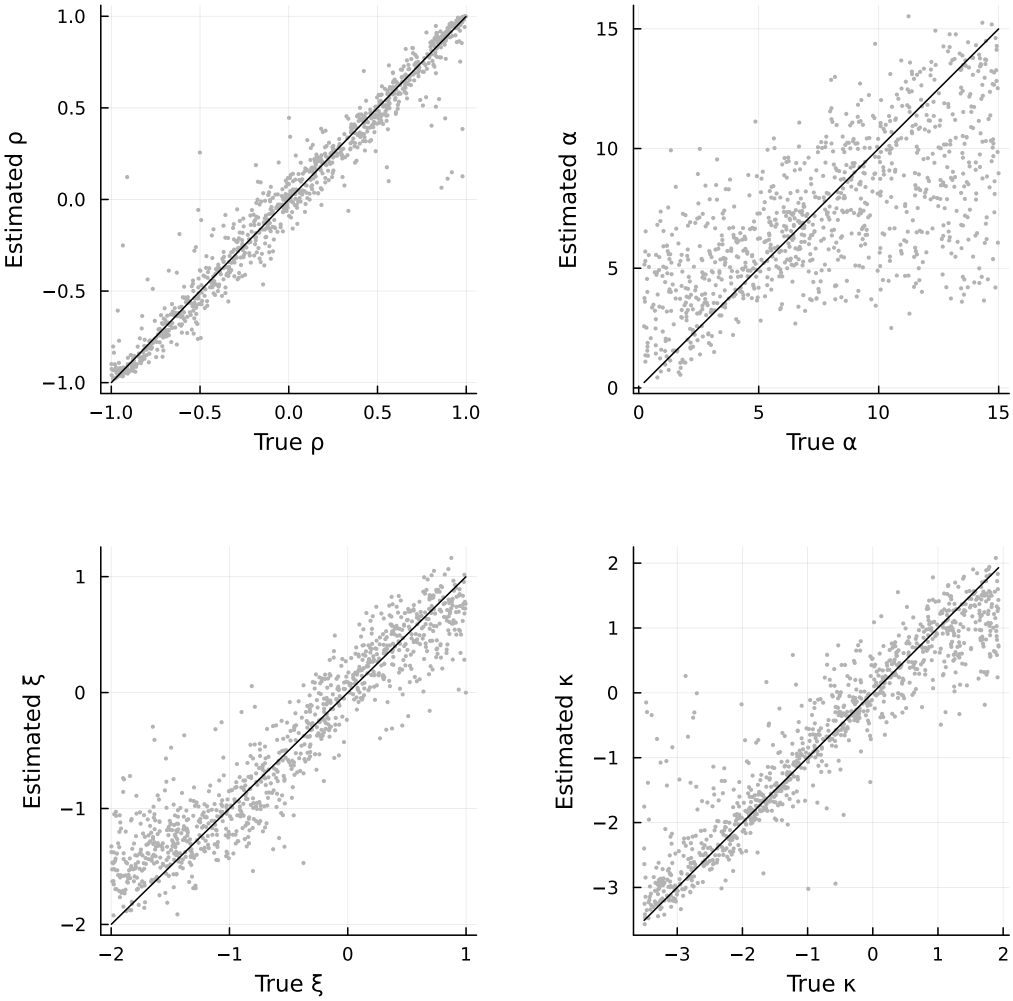

Figure S8: Assessment of the NBE when c_b is a Gaussian copula with correlation parameter ρ, c_t is Model E2 with parameters $(\alpha, \xi)'$ and with weighting function $\pi(x_1, x_2; \kappa) = (x_1 x_2)^{\exp(\kappa)}$, $x_1, x_2 \in (0, 1)$ for a sample size of $n = 1000$.

Table S6: Coverage probability and average length of the 95% uncertainty intervals for the parameters (left) and for $\chi(u)$ at levels $u = \{0.50, 0.80, 0.95\}$ (right) obtained via a non-parametric bootstrap procedure averaged over 1000 models fitted using a NBE (rounded to 2 decimal places).

Parameter	Coverage	Length
ρ	0.83	0.22
α	0.57	4.48
ξ	0.72	0.59
κ	0.75	0.96

$\chi(u)$	Coverage	Length
$\chi(0.50)$	0.88	0.05
$\chi(0.80)$	0.84	0.07
$\chi(0.95)$	0.83	0.10

S2.2 Model W

S2.2.1 Variable sample size and censoring level

Comparison with censored maximum likelihood estimation

The comparison between the NBE and CMLE for the remaining three parameter vectors considered in the simulation study of Section 4.2 of the main paper is given in Figure S9.

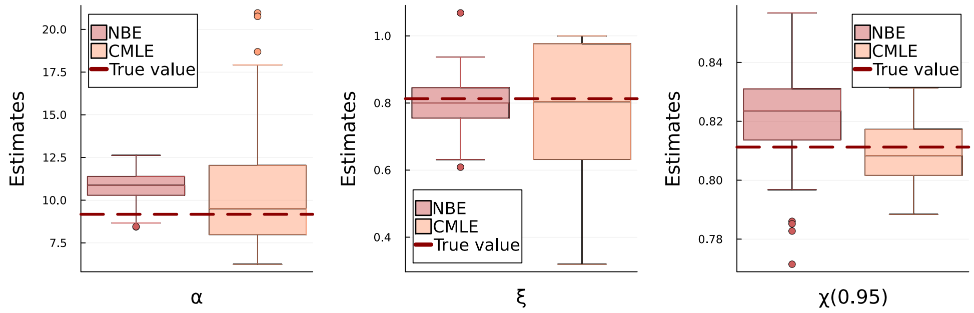

(a)

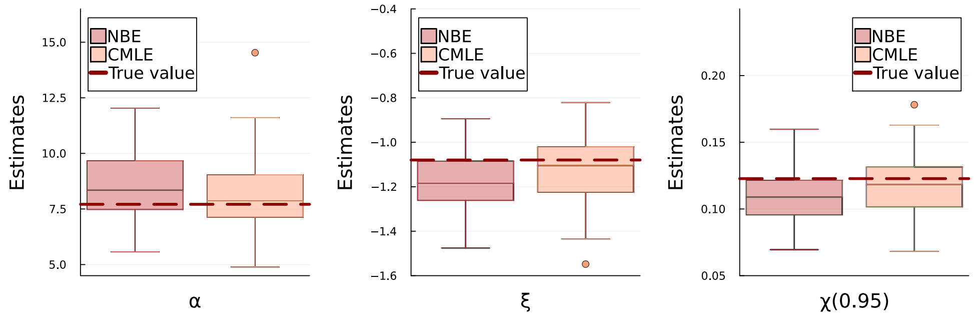

(b)

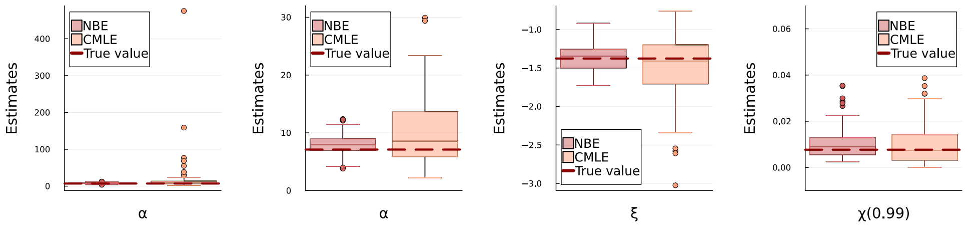

(c)

Figure S9: Comparison between parameter estimates $\hat{\boldsymbol{\theta}} = (\hat{\alpha}, \hat{\xi})'$ given by CMLE (orange) and by NBE (red), and corresponding $\chi(u)$, for 100 samples with $n = 1000$. The true parameters are given by the red line. (a) $\boldsymbol{\theta}_3 = (9.17, 0.81)'$ with $\tau_3 = 0.80$ and $u = 0.95$, (b) $\boldsymbol{\theta}_4 = (7.71, -1.08)'$ with $\tau_4 = 0.73$ and $u = 0.95$, and (c) $\boldsymbol{\theta}_5 = (7.10, -1.38)'$ with $\tau_5 = 0.98$ and $u = 0.99$. For better visualisation, the larger outliers obtained through MLE are removed for $\hat{\alpha}$ in (e).

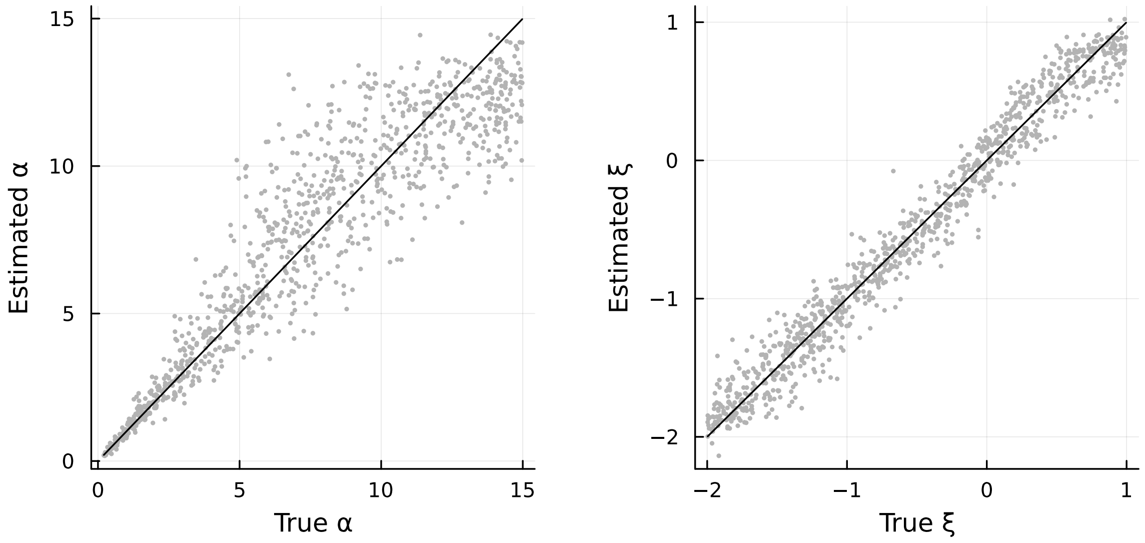

Figure S10: Assessment of the NBE for Model W with parameters $\boldsymbol{\theta} = (\alpha, \xi)'$ for a sample size of $n = 1000$ and fixed censoring level $\tau = 0.8$.

For comparison with the simulation study of Model W given in Section 4.2, we now present the results for when both sample size n and censoring level τ are kept fixed, and for when the sample size is assumed variable but the censoring level is kept fixed.

S2.2.2 Fixed sample size and censoring level

We first assume that the sample size and the censoring level are kept fixed at $n = 1000$ and $\tau = 0.8$, respectively, and the performance of the NBE is shown in Figure S10. Similarly to the case presented in Section 4.2 of the main paper, the NBE exhibits some bias for larger values of α. This is also noticeable with the average length of the 95% uncertainty intervals obtained via a non-parametric bootstrap procedure given in Table S7. It can also be seen that the coverage probabilities are slightly higher than the ones from Section 4.2. This might be due to the fact that there are less unknown variables in this configuration. Finally, the coverage probabilities of 95% uncertainty intervals, and their average length, for $\chi(u)$ at levels $u = \{0.80, 0.95, 0.99\}$ are shown on the right of Table S7. The results are similar to the ones presented in the main paper, with a slightly higher coverage for larger u.

Comparison with censored maximum likelihood estimation

We compare the estimations obtained by the NBE and by the MLE for the five parameter vectors $\boldsymbol{\theta} = (\alpha, \xi)'$ considered in Section 4.2 with now fixed $\tau = 0.8$. Likewise before, each

Table S7: Coverage probability and average length of the 95% uncertainty intervals for the parameters (left) and for $\chi(u)$ at levels $u = \{0.80, 0.95, 0.99\}$ (right) obtained via a non-parametric bootstrap procedure averaged over 1000 models fitted using a NBE (rounded to 2 decimal places).

Parameter	Coverage	Length
α	0.80	3.98
ξ	0.89	0.52

$\chi(u)$	Coverage	Length
$\chi(0.80)$	0.91	0.07
$\chi(0.95)$	0.92	0.09
$\chi(0.99)$	0.92	0.10

Table S8: Coverage probability and average length of the 95% uncertainty intervals for the parameters (left) and for $\chi(u)$ at levels $u = \{0.80, 0.95, 0.99\}$ (right) obtained via a non-parametric bootstrap procedure averaged over 1000 models fitted using a NBE (rounded to 2 decimal places).

Parameter	Coverage	Length
α	0.73	3.53
ξ	0.80	0.46

$\chi(u)$	Coverage	Length
$\chi(0.80)$	0.90	0.06
$\chi(0.95)$	0.88	0.08
$\chi(0.99)$	0.85	0.09

data set is simulated 100 times and has a sample size of $n = 1000$. The results are shown in Figure S11; these are fairly similar to those obtained when n and τ are assumed unknown, and given in the main paper. This is also the configuration for which censored MLE is faster; for instance, on average, the CMLE took 159.298 seconds, while the NBE was 731 times faster with an average time of 0.218 seconds.

S2.2.3 Variable sample size and fixed censoring level

We now assume the sample size is unknown but we keep the censoring level fixed at $\tau = 0.8$. The performance of the NBE is given in Figure S12, where a similar behaviour to the results obtained either when n is assumed fixed or when τ is also assumed unknown. The coverage probabilities of the 95% uncertainty intervals obtained via (non-parametric) bootstrap, shown in Table S8, are now slightly lower than the ones from the case when n is assumed fixed at 1000. The coverage probabilities of 95% uncertainty intervals, and their average length, for $\chi(u)$ at levels $u = \{0.80, 0.95, 0.99\}$ are shown on the right of Table S8, and are similar in magnitude to the corresponding results presented in the main paper.

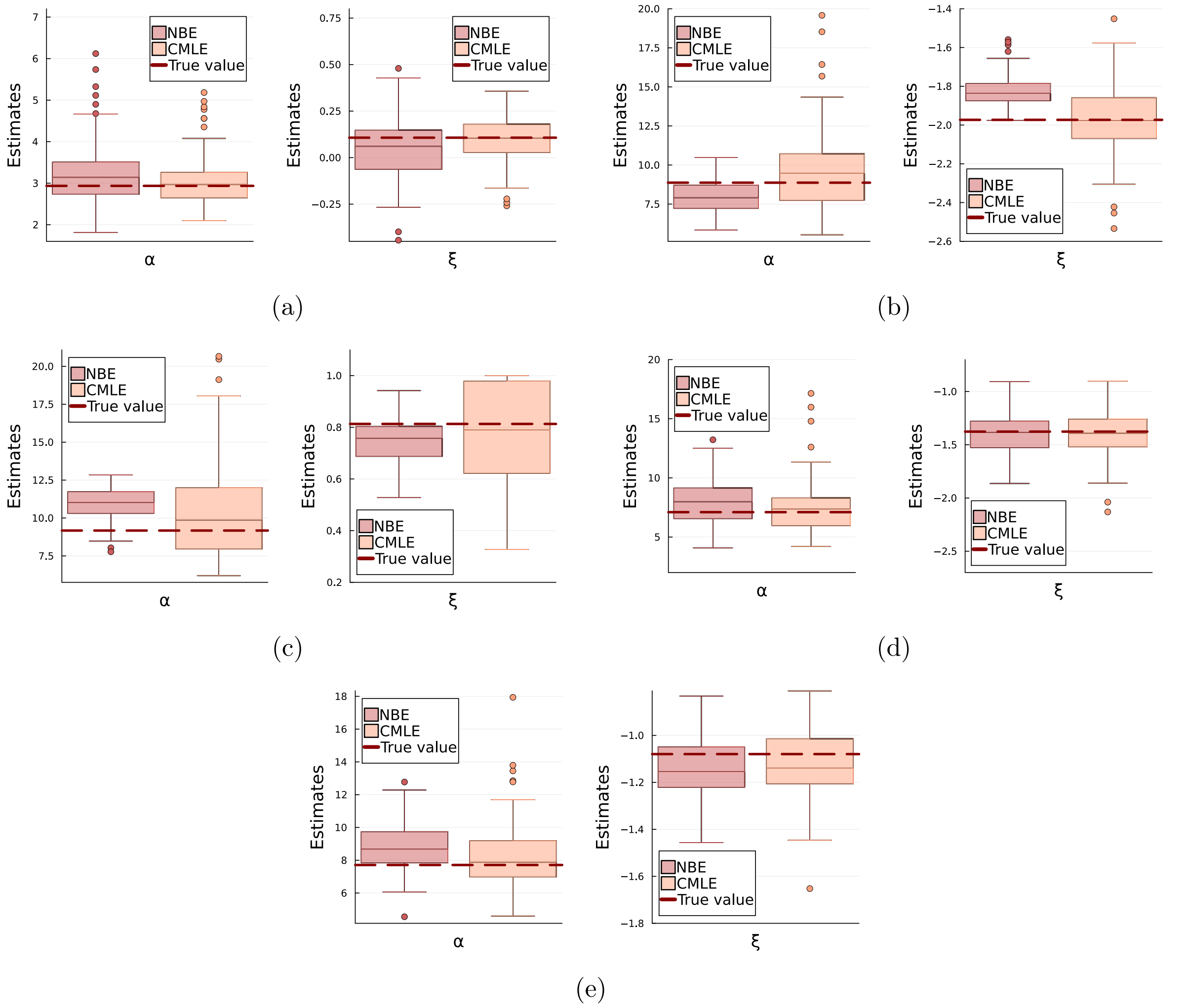

Figure S11: Comparison between parameter estimates $\hat{\boldsymbol{\theta}} = (\hat{\alpha}, \hat{\xi})'$ given by CMLE (orange) and by NBE (red) for 100 samples with $n = 1000$. The true parameters are given by the red line. (a) $\boldsymbol{\theta} = (2.94, 0.11)'$, (b) $\boldsymbol{\theta} = (8.87, -1.97)'$, (c) $\boldsymbol{\theta} = (9.17, 0.81)'$, (d) $\boldsymbol{\theta} = (7.10, -1.38)'$ and (e) $\boldsymbol{\theta} = (7.71, -1.08)'$.

Comparison with censored maximum likelihood estimation

The same five parameter vectors $\boldsymbol{\theta} = (\alpha, \xi)'$ considered for the cases where n is fixed at 1000 and the one presented in Section 4.2 are used to compare the NBE and CMLE estimates. Similarly to the previous case, we fix $\tau = 0.8$, and each data set with $n = 1000$ is simulated 100 times. No evident differences to the estimates obtained when n is assumed fixed and when n and τ are assumed unknown are visible from the results in Figure S13. For this case, the average time to get a NBE is of 0.470 seconds, which is about 339 faster than CMLE on average.

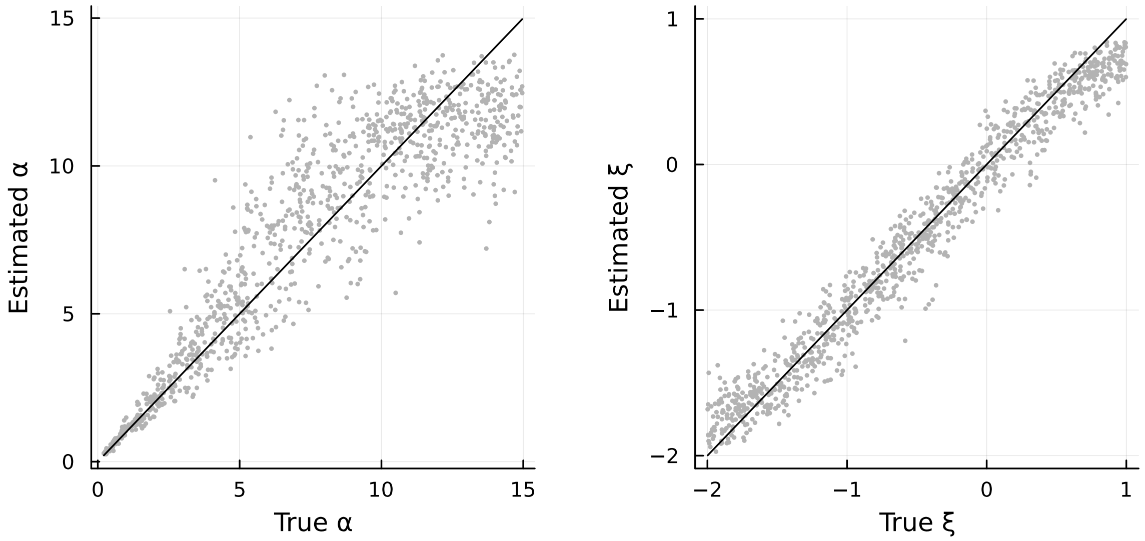

Figure S12: Assessment of the NBE for Model W with parameters $\boldsymbol{\theta} = (\alpha, \xi)'$ for a sample size of $n = 1000$ and fixed censoring level $\tau = 0.8$.

S2.2.4 General conclusions

The results with fixed censoring level ($\tau = 0.8$) with fixed ($n = 1000$) and variable sample size exhibit similar findings. In the case where both τ and n are fixed, however, the obtained bootstrap-based intervals have better coverage. When comparing the estimates given by the NBEs with the ones obtained by classical inference techniques, fixing one or both n and τ did not improve the performance of the estimators.

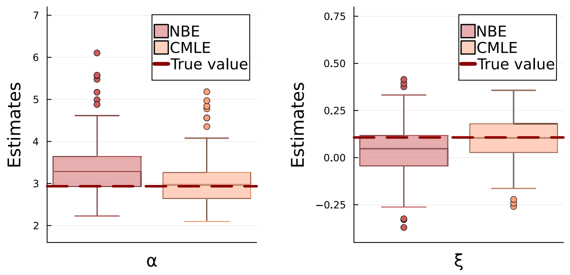

(a)

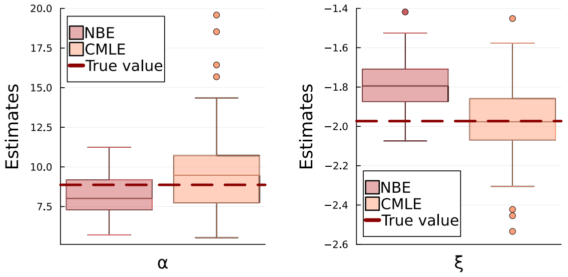

(b)

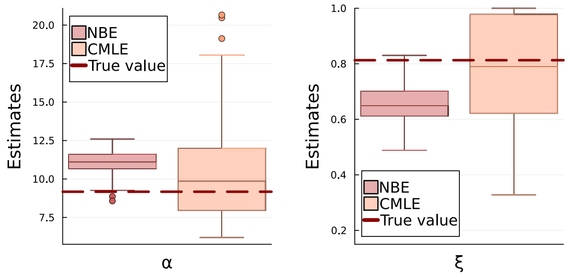

(c)

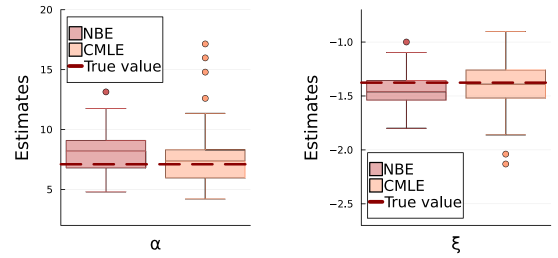

(d)

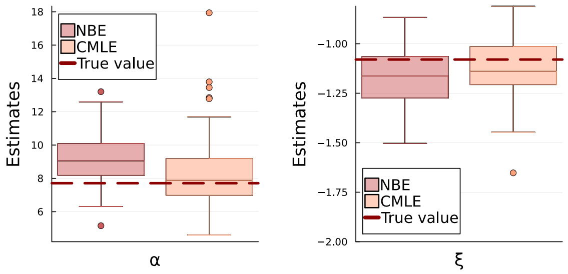

(e)

Figure S13: Comparison between parameter estimates $\hat{\boldsymbol{\theta}} = (\hat{\alpha}, \hat{\xi})'$ given by CMLE (orange) and by NBE (red) for 100 samples with $n = 1000$. The true parameters are given by the red line. (a) $\boldsymbol{\theta} = (2.94, 0.11)'$, (b) $\boldsymbol{\theta} = (8.87, -1.97)'$, (c) $\boldsymbol{\theta} = (9.17, 0.81)'$, (d) $\boldsymbol{\theta} = (7.10, -1.38)'$ and (e) $\boldsymbol{\theta} = (7.71, -1.08)'$.

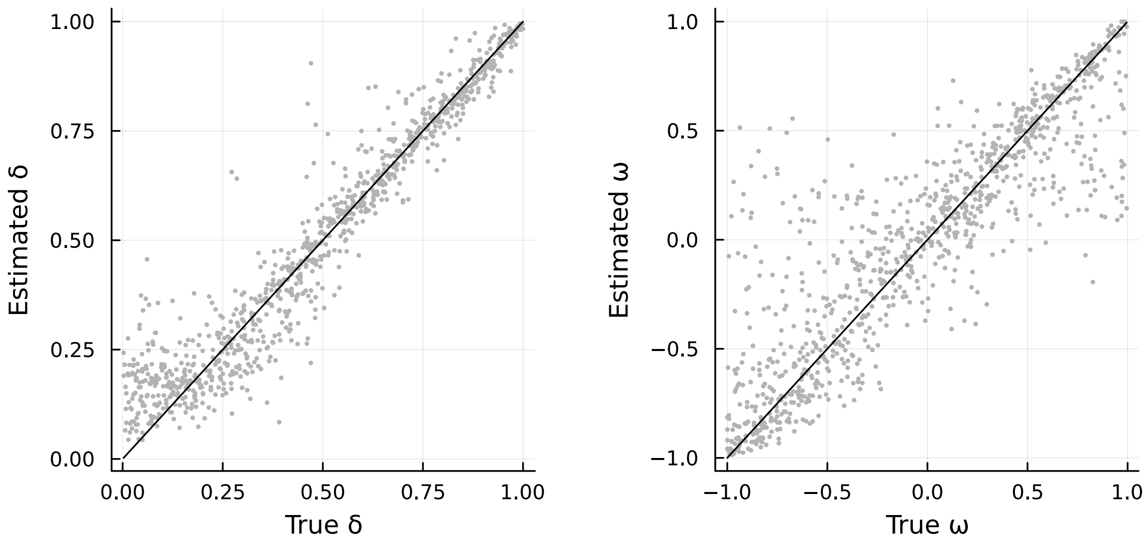

Figure S14: Assessment of the NBE for Model HW, where $\boldsymbol{V}$ follows a bivariate Gaussian copula, with parameters $\boldsymbol{\theta} = (\delta, \omega)'$ for a sample size of $n = 1000$.

Table S9: Coverage probability and average length of the 95% uncertainty intervals for the parameters (left) and for $\chi(u)$ at levels $u = \{0.80, 0.95, 0.99\}$ (right) obtained via a non-parametric bootstrap procedure averaged over 1000 models fitted using a NBE (rounded to 2 decimal places).

Parameter	Coverage	Length
δ	0.71	0.14
ω	0.75	0.48

$\chi(u)$	Coverage	Length
$\chi(0.80)$	0.90	0.08
$\chi(0.95)$	0.90	0.09
$\chi(0.99)$	0.90	0.10

S2.3 Model HW

We assess the performance of the NBE for Model HW. The results are shown in Figure S14 and Table S9. Similarly to Model W, there is some variability in the estimates, in particular for lower values of δ and ω. The coverage probabilities of 95% uncertainty intervals, and their average length, for $\chi(u)$ at levels $u = \{0.80, 0.95, 0.99\}$, shown on the right of Table S8, indicate that even with biased results, the NBE is able to characterise the extremal dependence at high levels of u. We note that, similarly to the study involving Model W given in the main paper, the coverage probabilities for $\chi(u)$ are achieved with new data sets for 1000 parameter configurations, each generated with a fixed censoring level $\tau = 0.8$.

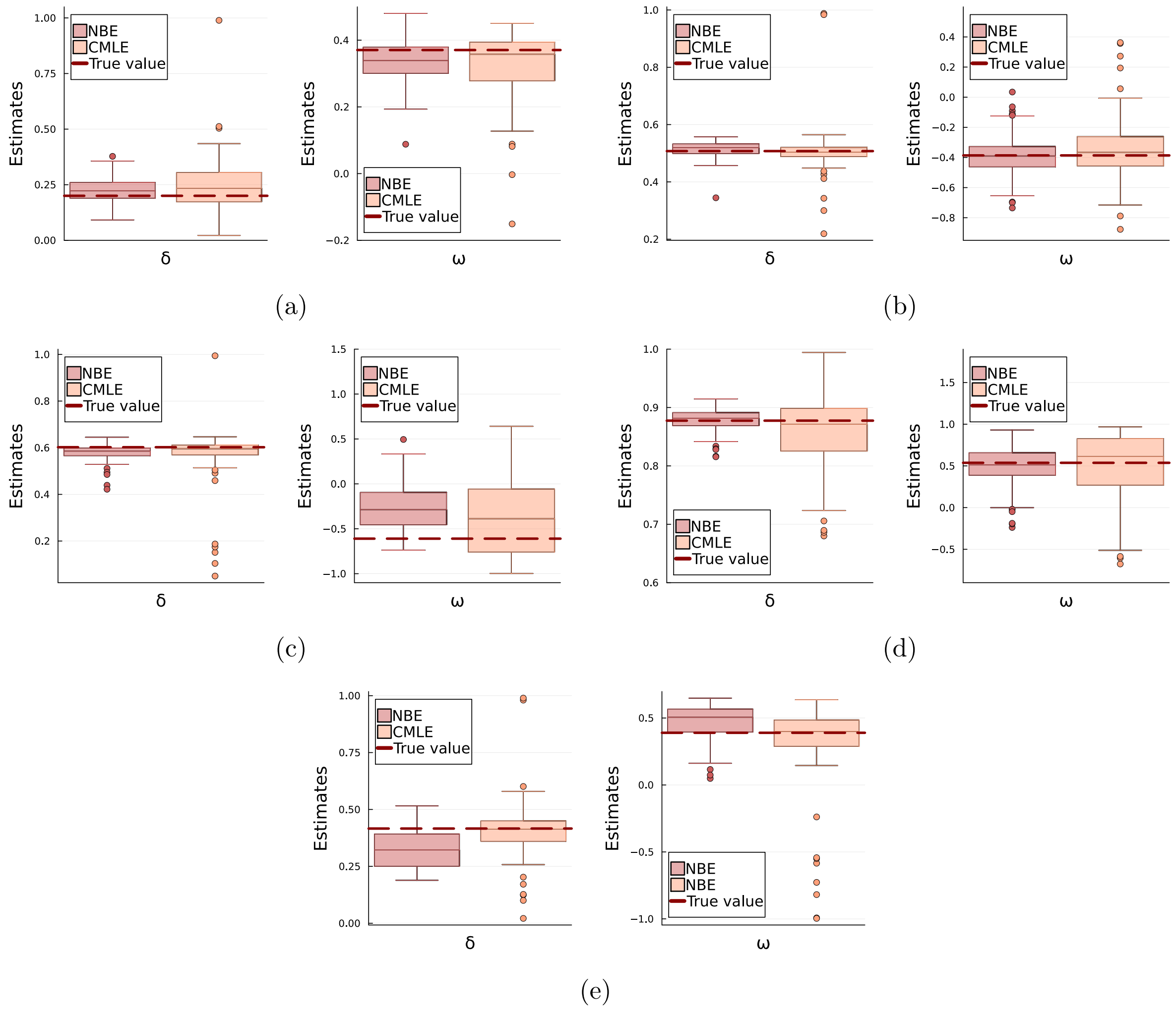

Figure S15: Comparison between parameter estimates $\hat{\boldsymbol{\theta}} = (\hat{\delta}, \hat{\omega})'$ given by CMLE (orange) and by NBE (red) for 100 samples with $n = 1000$. The true parameters are given by the red line. (a) $\boldsymbol{\theta} = (0.20, 0.37)'$ with $\tau = 0.65$, (b) $\boldsymbol{\theta} = (0.51, -0.39)'$ with $\tau = 0.76$, (c) $\boldsymbol{\theta} = (0.60, -0.61)'$ with $\tau = 0.95$, (d) $\boldsymbol{\theta} = (0.88, 0.54)'$ with $\tau = 0.57$ and (e) $\boldsymbol{\theta} = (0.42, 0.39)'$ with $\tau = 0.91$.

Comparison with censored maximum likelihood estimation

Similarly to the previous cases, we generate five parameter vectors from the priors considered in the main paper, along with the corresponding data sets of size $n = 1000$, and simulate each data set 100 times. The comparison between the NBE and CMLE is shown in Figure S15; the estimates given by the NBE are quite good, particularly for lower censoring levels. As for computational times, the CMLE took 688.837 seconds on average, while the NBE was 2 542 times faster with an average of 0.271 seconds.

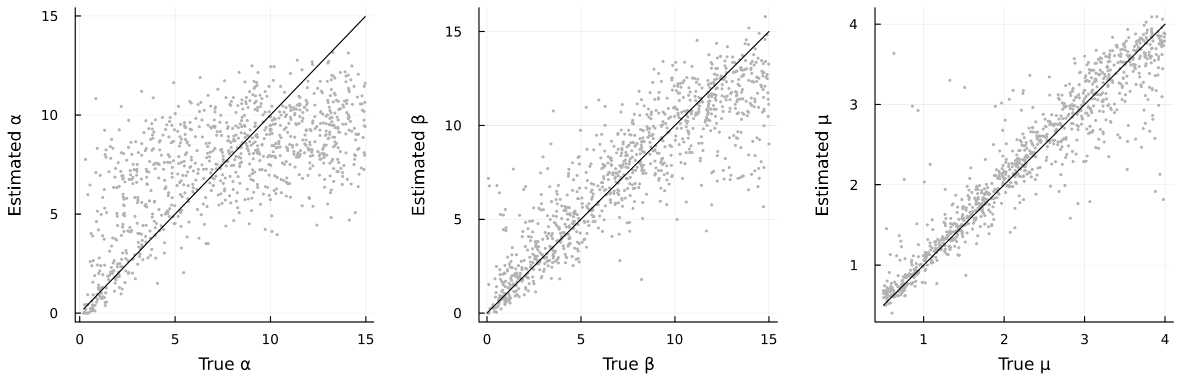

Figure S16: Assessment of the NBE for Model E1 with parameters $\boldsymbol{\theta} = (\alpha, \beta, \mu)'$ for a sample size of $n = 1000$.

Table S10: Coverage probability and average length of the 95% uncertainty intervals for the parameters (left) and for $\chi(u)$ at levels $u = \{0.80, 0.95, 0.99\}$ (right) obtained via a non-parametric bootstrap procedure averaged over 1000 models fitted using a NBE (rounded to 2 decimal places).

Parameter	Coverage	Length
α	0.44	3.60
β	0.68	3.36
μ	0.77	0.60

$\chi(u)$	Coverage	Length
$\chi(0.80)$	0.82	0.11
$\chi(0.95)$	0.82	0.11
$\chi(0.99)$	0.81	0.12

S2.4 Model E1

Figure S16 and Table S10 show the performance of the NBE for Model E1. As can be seen, the parameters α and β have the lowest coverage probability and highest average length of their 95% uncertainty intervals; this is in agreement with the variability shown when comparing the true values with their estimated values in Figure S16. As before, we compute the coverage probabilities of the 95% confidence intervals for $\chi(u)$ at levels $u = \{0.80, 0.95, 0.99\}$ by considering new data sets for 1000 parameter configurations, each generated with a fixed censoring level $\tau = 0.8$. The results, given on the right of Table S10, indicate that the bias shown by the NBE does not seem to influence the estimation of $\chi(u)$. In particular, the true value is within the confidence intervals in more than 81% of the time.

Comparison with censored maximum likelihood estimation

We compare the estimations obtained by the NBE and by the CMLE for five parameter vectors $\boldsymbol{\theta} = (\alpha, \beta, \mu)'$ generated from the priors considered in the main paper. Each corresponding data set has $n = 1000$ and is simulated 100 times. Similarly to the other models considered, the NBE is more biased than the CMLE and, in some cases, can be more variable than the CMLE. Despite that, on average, the CMLE took 17 minutes, whereas the NBE took 0.159 seconds, meaning that the NBE is about 6 565 times faster.

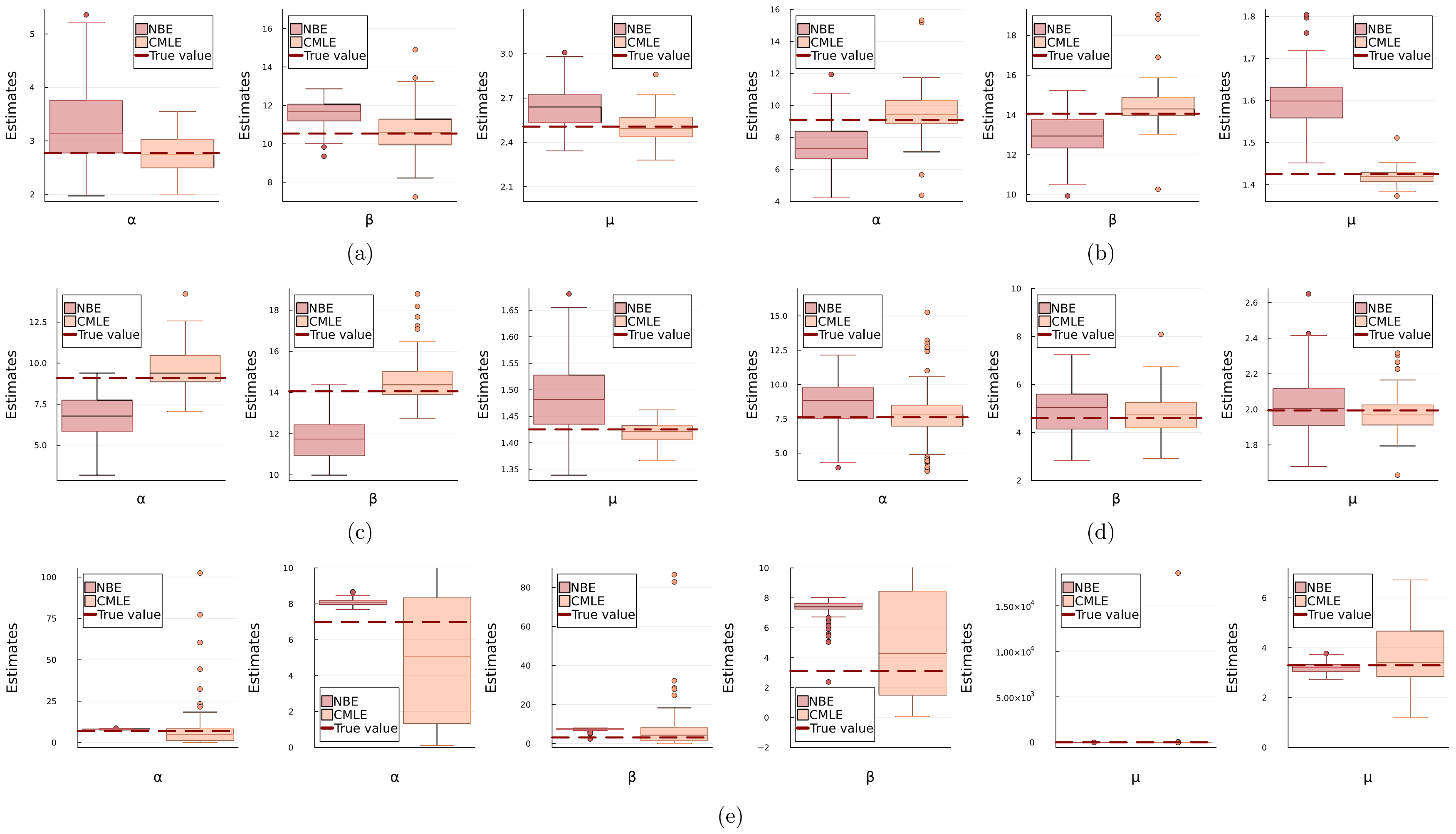

Figure S17: Comparison between parameter estimates $\hat{\boldsymbol{\theta}} = (\hat{\alpha}, \hat{\beta}, \hat{\mu})'$ given by CMLE (orange) and by NBE (red) for 100 samples with $n = 1000$. The true parameter values are given by the red line. (a) $\boldsymbol{\theta} = (2.77, 10.54, 2.51)'$ with $\tau = 0.79$, (b) $\boldsymbol{\theta} = (9.09, 14.06, 1.43)'$ with $\tau = 0.60$, (c) $\boldsymbol{\theta} = (9.09, 14.06, 1.43)'$ with $\tau = 0.80$, (d) $\boldsymbol{\theta} = (7.61, 4.60, 1.99)'$ with $\tau = 0.73$ and (e) $\boldsymbol{\theta} = (6.99, 3.12, 3.30)'$ with $\tau = 0.98$. For better visualisation, the larger values obtained through CMLE were removed for $\boldsymbol{\theta}$ in (e).

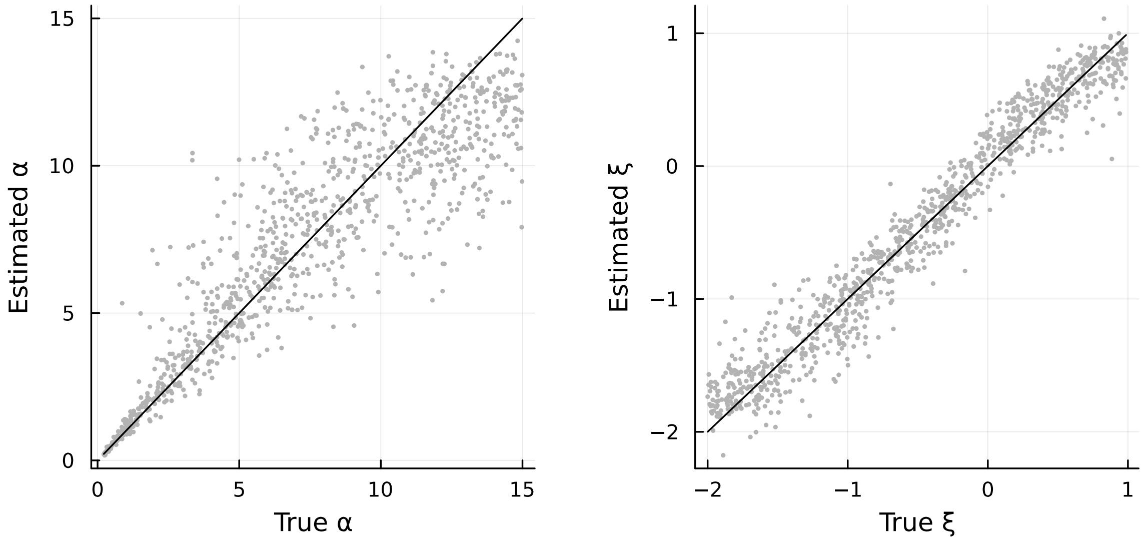

Figure S18: Assessment of the NBE for Model E2 with parameters $\boldsymbol{\theta} = (\alpha, \xi)'$ for a sample size of $n = 1000$.

Table S11: Coverage probability and average length of the 95% uncertainty intervals for the parameters (left) and for $\chi(u)$ at levels $u = \{0.80, 0.95, 0.99\}$ (right) obtained via a non-parametric bootstrap procedure averaged over 1000 models fitted using a NBE (rounded to 2 decimal places).

Parameter	Coverage	Length
α	0.71	3.64
ξ	0.81	0.52

$\chi(u)$	Coverage	Length
$\chi(0.80)$	0.94	0.06
$\chi(0.95)$	0.90	0.10
$\chi(0.99)$	0.87	0.12

S2.5 Model E2

For the final model, we consider Model E2, for which the performance of the NBE is shown in Figure S18 and Table S11. The parameter α shows the highest variability, especially for larger values, with its 95% uncertainty interval being wider and having lower coverage probability. The coverage probabilities of 95% uncertainty intervals of $\chi(u)$ at levels $u = \{0.80, 0.95, 0.99\}$, shown on the right of Table S11, indicate that the this measure is well calibrated, with the true $\chi(u)$ lying within the intervals at least 87% of the time in spite of the bias shown by the NBE. As before, the results for $\chi(u)$ are obtained with new data sets for 1000 parameter configurations with a fixed censoring level $\tau = 0.8$.

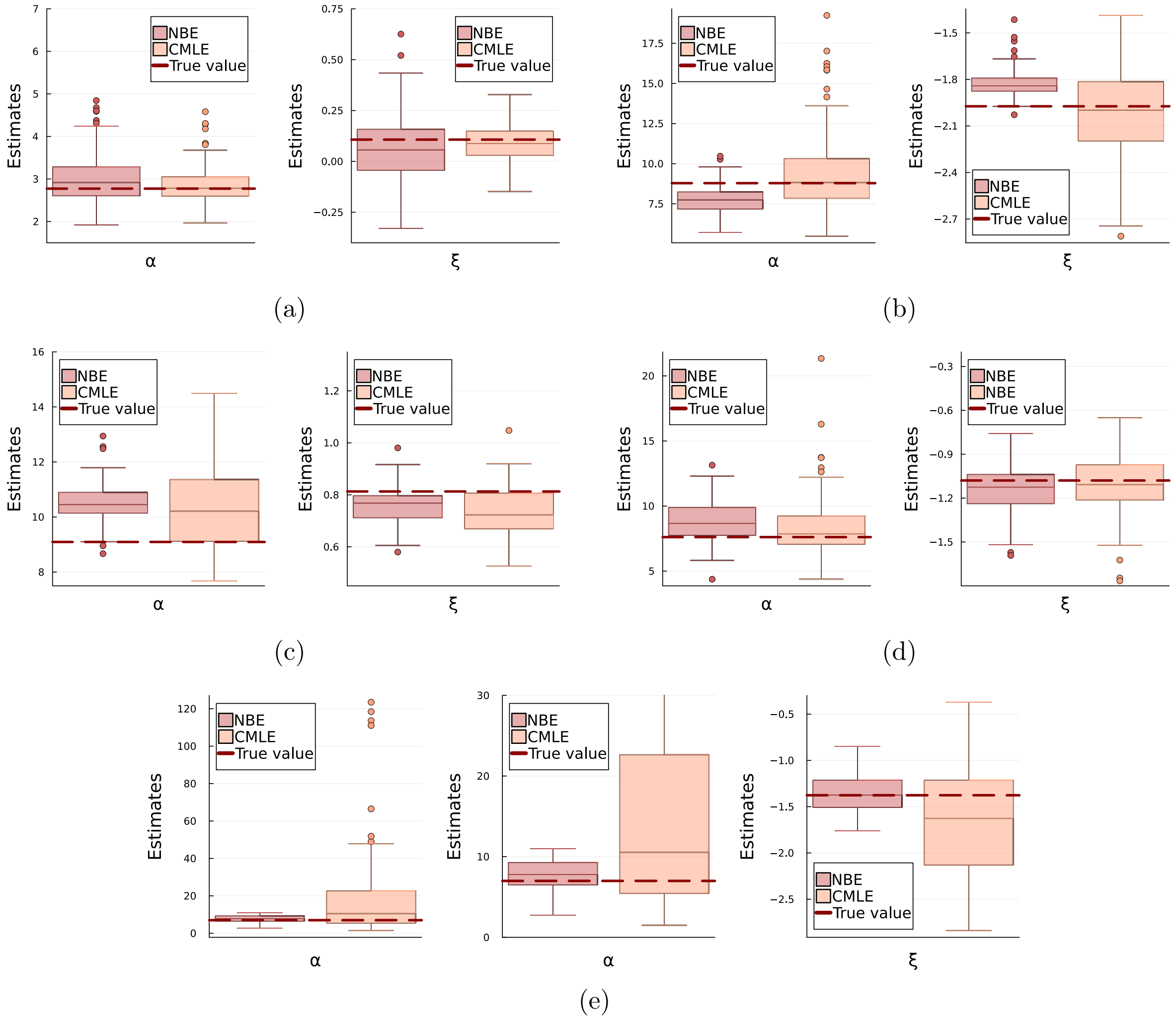

Figure S19: Comparison between parameter estimates $\hat{\boldsymbol{\theta}} = (\hat{\alpha}, \hat{\xi})'$ given by CMLE (orange) and by NBE (red) for 100 samples with $n = 1000$. The true parameter values are given by the red line. (a) $\boldsymbol{\theta} = (2.77, 0.11)'$ with $\tau = 0.79$, (b) $\boldsymbol{\theta} = (8.79, -1.97)'$ with $\tau = 0.60$, (c) $\boldsymbol{\theta} = (9.09, 0.81)'$ with $\tau = 0.80$, (d) $\boldsymbol{\theta} = (7.61, -1.08)'$ with $\tau = 0.73$ and (e) $\boldsymbol{\theta} = (6.99, -1.38)'$ with $\tau = 0.98$. For better visualisation, the larger outliers obtained through CMLE were removed for $\hat{\alpha}$ in (e).

Comparison with censored maximum likelihood estimation

The estimations obtained by the NBE and the CMLE are assessed for five parameter vectors $\boldsymbol{\theta} = (\alpha, \xi)'$ and their corresponding data sets with $n = 1000$, each simulated 100 times. The results, shown in Figure S19, indicate that the NBE is more biased than the CMLE. However, as with the previous models, the NBE is about 1 052 times faster than CMLE; in particular, the CMLE took 585.729 seconds on average to compute, whilst the NBE took 0.557 seconds.

Table S12: Summary of the neural network architecture used for the NBC. The input array to the first layer represents the dimension d of data set $\boldsymbol{Z}$ and the one-hot encoded vector $\boldsymbol{I}$; see Section 2.1.3. The output array of the last layer of neural network ψ differ based on the number of models K : for $K = 2$, we have $w_\psi = 128$, while for $K = 4$, $w_\psi = 256$. The output array $[K]$ of the last layer of neural network ϕ represents the output class probabilities $\hat{\boldsymbol{p}}$.

Neural network	Input dimension	Output dimension
$\boldsymbol{\psi}(\cdot)$	$[2, 2]$	$[128]$
	$[128]$	$[128]$
	$[128]$	$[w_\psi]$
$\boldsymbol{\phi}(\cdot)$	$[d_\psi + 1]$	$[128]$
	$[128]$	$[K]$

S3 Model selection assessment

The neural network architecture used for model selection (recall Section 4.3 of the main paper) is given in Table S12.

S3.1 Effect of sample size N

We perform an additional study to analyse the effect of sample size n on the comparative differences between the NBC and BIC. To do so, we have considered 4 cases when $K = 4$, and generated 500 models m, each with samples of size $n \in N \in \{200, 500, 1400, 2000\}$. The results are shown in Figure S20. As can be expected since a lower n results in fewer number of exceedances for likelihood-based estimation, the BIC performs generally worse than the NBC for $n = 200$ and $n = 500$. The NBC is more consistent across the different sample sizes; however, as with the study from Section 4.3 of the main paper, it still struggles to correctly identify samples from Model W.

The case of $n = 2000$ presents interesting results, as well. Recall that the NBC was trained for sample sizes between 100 and 1500. By considering a sample size of $n = 2000$, we were interested in assessing the performance of the NBC for a sample size outside of the interval considered. The bottom right panel of Figure S20 shows that the NBC is still able to correctly identify the majority of the data sets, and better than BIC.

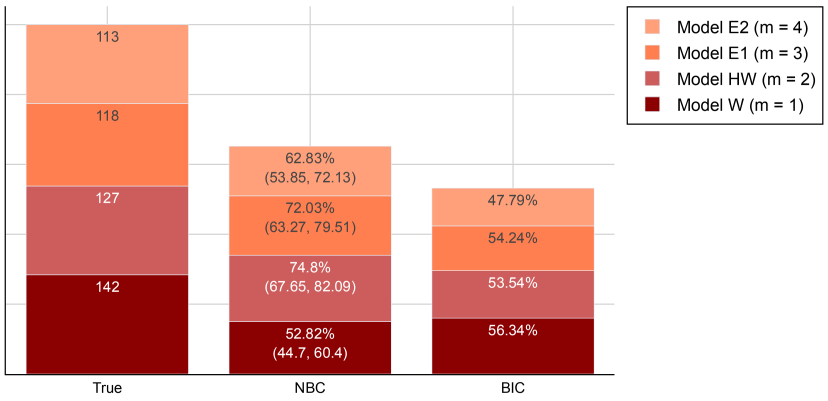

(a) $n = 200$.

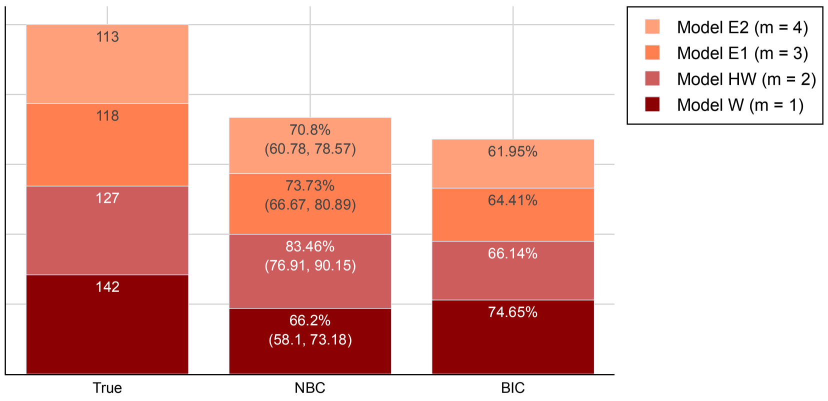

(b) $n = 500$.

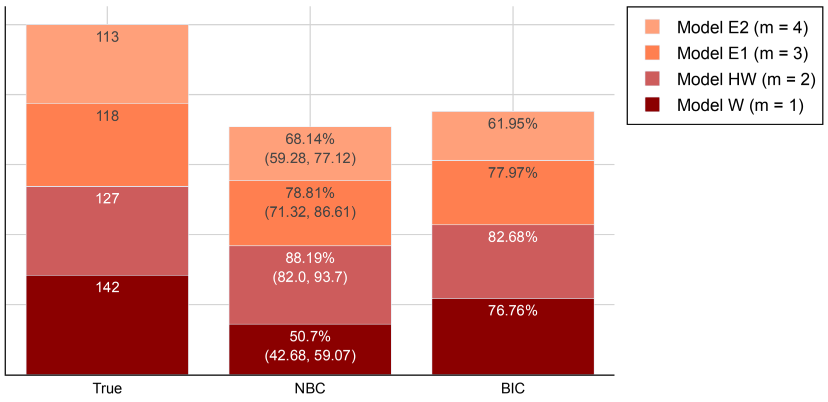

(c) $n = 1400$.

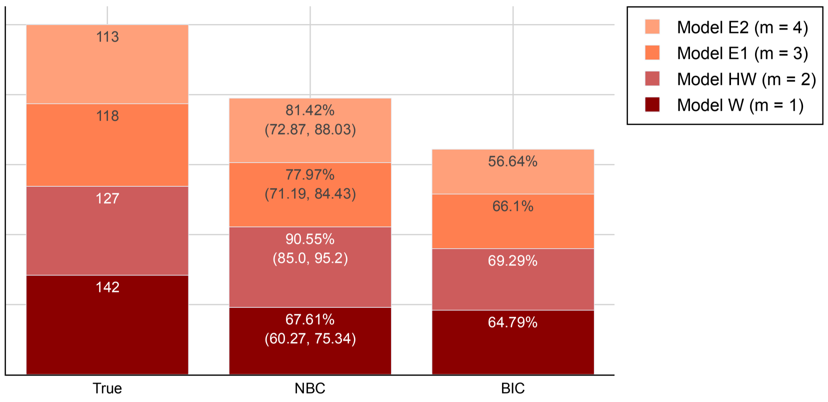

(d) $n = 2000$.

Figure S20: Proportion (in %) of correctly identified data sets when $K = 4$ through the NBCs (middle) and through BIC (right) for $n \in \{200, 500, 1400, 2000\}$. The true counts of data sets generated from models $m = 1$ (red), $m = 2$ (light red), $m = 3$ (orange) and $m = 4$ (light orange) are given in the left bar plot. The 95% confidence intervals for the proportions of correctly identified data sets by the NBC are given in brackets.

Table S13: Model selection procedure obtained through the probabilities given by the NBC and through BIC (left), and parameter estimates given by the NBE and by the CMLE (right) for the selected model (in bold). All the values are rounded up to 3 decimal places.

Model	$\hat{\boldsymbol{p}}_{\text{NBC}}$	BIC
Model W	4.609×10^{-5}	567.348
Model HW	**0.987**	**558.482**
Model E1	2.392×10^{-8}	565.636
Model E2	0.013	564.636

Method	Model parameters
NBE (Model HW)	$(\hat{\delta}, \hat{\omega}) = (0.201, 0.400)$
CMLE (Model HW)	$(\hat{\delta}, \hat{\omega}) = (0.107, 0.442)$

S4 Misspecified scenarios

We present now two examples, one for each study performed in Section 4.4 from the main paper. For each case, the best model is selected through the trained NBC, and the vector of parameters is estimated using the NBE trained for inference on the selected model. In addition, a comparison with classical model selection tools and inference is given. As a further diagnostic, we compare $\chi(u)$ for $u \in (0,1)$ obtained with the NBE for the estimated model with its empirical counterparts, the true values, and those obtained by the model selected and estimated using BIC and CMLE.

Results for model selection through the NBC and BIC for the Gaussian data case can be seen on the left of Table S13, and the estimates for the vector of parameters obtained by the NBE and CMLE are on the right. The NBC and BIC both select Model HW as the most suitable one for the data set. Additionally, both models indicate the presence of asymptotic independence since $\hat{\delta} \leq 0.5$. This is in agreement with the underlying Gaussian data being AI. The comparison between $\chi(u)$ obtained by the models estimated through the NBE and the CMLE, with the true values of $\chi(u)$ based on the Gaussian copula, and their empirical estimates, for $u \in [0.75, 0.99]$ are shown in the left panel of Figure S21. The model estimates obtained through the CMLE are slightly closer to the truth than the ones given by the NBE; however, both estimates are closer to the empirical estimates. Overall, the extremal dependence behaviour of the data is well captured by the trained NBE.

Table S13 gives the results for model selection and parameter estimation for the logistic data case. Again, for the model selection, the NBC and BIC agree and select Model HW as the best model to fit the data set. Looking at the parameter that indicates the extremal dependence structure, we have $\hat{\delta} > 0.5$, both correctly suggesting the presence of asymptotic dependence. The comparison between $\chi(u)$ obtained by the models estimated

Table S14: Model selection procedure obtained through the probabilities given by the NBC and through BIC (left), and parameter estimates given by the NBE and by the CMLE (right) for the selected model (in bold). All the values are rounded to 3 decimal places.

Model	$\hat{\boldsymbol{p}}_{\text{NBC}}$	BIC
Model W	7.774×10^{-5}	-55.802
**Model HW**	$\mathbf{0.999}$	$\mathbf{-57.846}$
Model E1	2.104×10^{-7}	-56.164
Model E2	0.001	-64.802

Method	Model parameters
NBE (Model HW)	$(\hat{\delta}, \hat{\omega}) = (0.640, -0.147)$
CMLE (Model HW)	$(\hat{\delta}, \hat{\omega}) = (0.621, 0.523)$

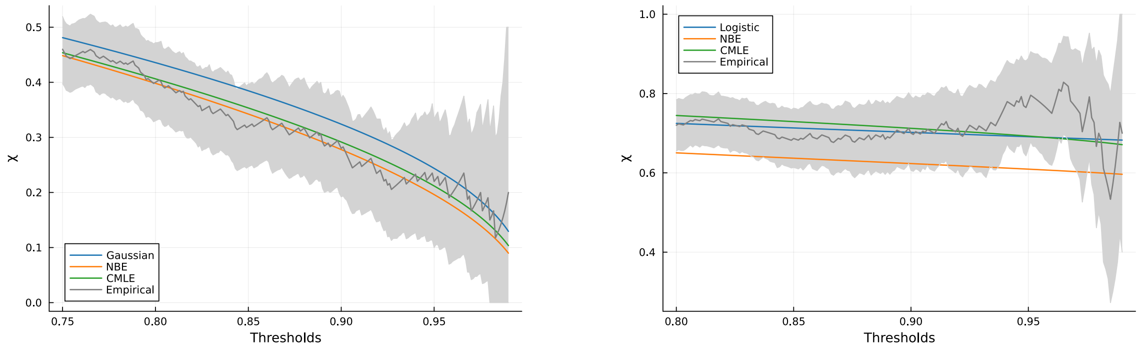

(a)

(b)

Figure S21: Model-based $\chi(u)$ given by the NBE (in orange) and by the CMLE (in green), and empirical $\chi(u)$ (in grey) for $u \in [\tau, 0.99]$. The 95% confidence bands were obtained by boostrapping. (a) $\chi(u)$ for a Gaussian copula with correlation parameter $\rho = 0.5$ (in blue) and censoring level $\tau = 0.75$, and (b) $\chi(u)$ for a logistic distribution with dependence parameter $\alpha_L = 0.4$ (in blue) and censoring level $\tau = 0.8$. Note that $\chi(u)$ for the logistic data and for the model given by the CMLE almost overlap (right).

through the NBE and the CMLE, with the true values $\chi(u)$ for the logistic data, and their empirical estimates, for $u \in [0.8, 0.99]$ is shown in right panel of Figure S21. For this case, the estimated model $\chi(u)$ given by the CMLE almost overlaps with the true values for the logistic data. On the other hand, the model $\chi(u)$ estimated by the NBE seems to under-estimate the truth. However, as before, the extremal dependence structure is still reasonably well captured with the trained NBE.

S5 Case study: changes in geomagnetic field fluctuations

In this section, we summarise the results for the remaining censoring levels for each pair of locations. Contrarily to the main paper, we only show the selected model for each censoring level and the type of extremal dependence estimated with it.

Pair (SCO, STF)

Table S15: Model selected by the NBC for censoring levels $\tau = \{0.60, 0.65, \ldots, 0.95\}$ and parameter estimates given by the trained NBE for pair (SCO, STF). All the values are rounded up to 3 decimal places.

τ	Model	$\hat{\boldsymbol{p}}_{\text{NBC}}$	$\hat{\boldsymbol{\theta}}_{\text{NBE}}$	Extremal dependence
0.60	Model HW	0.999	$(\hat{\delta}, \hat{\omega}) = (0.170, 0.743)$	AI
0.65	Model HW	0.998	$(\hat{\delta}, \hat{\omega}) = (0.178, 0.767)$	AI
0.70	Model HW	0.954	$(\hat{\delta}, \hat{\omega}) = (0.178, 0.800)$	AI
0.75	Model HW	0.906	$(\hat{\delta}, \hat{\omega}) = (0.195, 0.767)$	AI
0.80	Model HW	0.917	$(\hat{\delta}, \hat{\omega}) = (0.228, 0.742)$	AI
0.85	Model HW	0.922	$(\hat{\delta}, \hat{\omega}) = (0.258, 0.714)$	AI
0.90	Model E2	0.935	$(\hat{\alpha}, \hat{\xi}) = (3.512, -0.368)$	AI
0.95	Model E2	0.640	$(\hat{\alpha}, \hat{\xi}) = (3.616, -0.399)$	AI

Pair (SCO, STJ)

Table S16: Model selected by the NBC for censoring levels $\tau = \{0.60, 0.65, \ldots, 0.95\}$ and parameter estimates given by the trained NBE for pair (SCO, STJ). All the values are rounded up to 3 decimal places.

τ	Model	$\hat{\boldsymbol{p}}_{\text{NBC}}$	$\hat{\boldsymbol{\theta}}_{\text{NBE}}$	Extremal dependence
0.60	Model HW	1.000	$(\hat{\delta}, \hat{\omega}) = (0.085, 0.560)$	AI
0.65	Model HW	1.000	$(\hat{\delta}, \hat{\omega}) = (0.093, 0.580)$	AI
0.70	Model HW	1.000	$(\hat{\delta}, \hat{\omega}) = (0.109, 0.586)$	AI
0.75	Model HW	1.000	$(\hat{\delta}, \hat{\omega}) = (0.104, 0.591)$	AI
0.80	Model HW	0.958	$(\hat{\delta}, \hat{\omega}) = (0.105, 0.626)$	AI
0.85	Model E2	0.900	$(\hat{\alpha}, \hat{\xi}) = (2.316 - 0.791)$	AI
0.90	Model E2	0.940	$(\hat{\alpha}, \hat{\xi}) = (2.748, -0.834)$	AI
0.95	Model E2	0.875	$(\hat{\alpha}, \hat{\xi}) = (3.223, -0.782)$	AI

Pair (STF, STJ)

Table S17: Model selected by the NBC for censoring levels $\tau = \{0.60, 0.65, \ldots, 0.95\}$ and parameter estimates given by the trained NBE for pair (STF, STJ). All the values are rounded up to 3 decimal places.

τ	Model	$\hat{\boldsymbol{p}}_{\text{NBC}}$	$\hat{\boldsymbol{\theta}}_{\text{NBE}}$	Extremal dependence
0.60	Model HW	1.000	$(\hat{\delta}, \hat{\omega}) = (0.106, 0.558)$	AI
0.65	Model HW	1.000	$(\hat{\delta}, \hat{\omega}) = (0.113, 0.571)$	AI
0.70	Model HW	1.000	$(\hat{\delta}, \hat{\omega}) = (0.117, 0.588)$	AI
0.75	Model HW	0.996	$(\hat{\delta}, \hat{\omega}) = (0.134, 0.585)$	AI
0.80	Model HW	0.920	$(\hat{\delta}, \hat{\omega}) = (0.125, 0.610)$	AI
0.85	Model E2	0.672	$(\hat{\alpha}, \hat{\xi}) = (2.420, -0.849)$	AI
0.90	Model E2	0.727	$(\hat{\alpha}, \hat{\xi}) = (2.573, -0.846)$	AI
0.95	Model E2	0.832	$(\hat{\alpha}, \hat{\xi}) = (3.711, -0.772)$	AI

References

Frank, M. J. (1979). On the simultaneous associativity of $F(x, y)$ and $x + y - F(x, y)$. *Aequationes Mathematicae*, 19:194–226.

Joe, H. (1996). Families of m-variate distributions with given margins and $m(m-1)/2$ bivariate dependence parameters. *Lecture Notes-Monograph Series*, 28:120–141.

Sainsbury-Dale, M., Zammit-Mangion, A., and Huser, R. (2024). Likelihood-free parameter estimation with neural Bayes estimators. *The American Statistician*, 78(1):1–14.

Wadsworth, J. L., Tawn, J. A., Davison, A. C., and Elton, D. M. (2017). Modelling across extremal dependence classes. *Journal of the Royal Statistical Society: Series B*, 79:149–175.
