# Supplementary figures and images for: Neural Bayes estimation and selection of complex bivariate extremal dependence models
Source: Extremes (Boston). 2025 Dec 5;29(2):347–86. doi: 10.1007/s10687-025-00521-8 (PMC13263241; doi:10.1007/s10687-025-00521-8)

Estimates

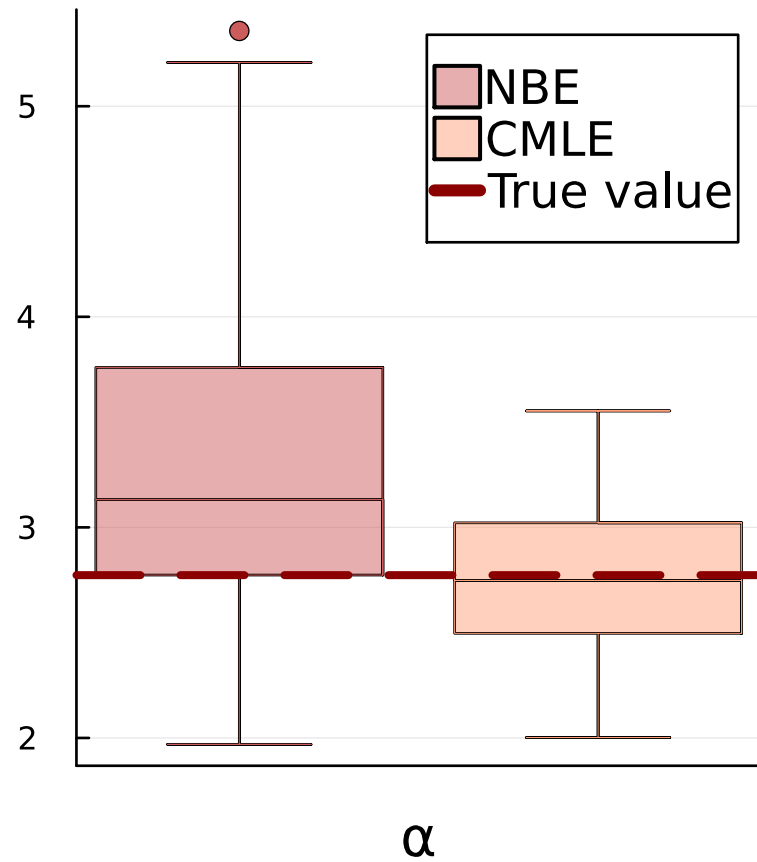

Estimates

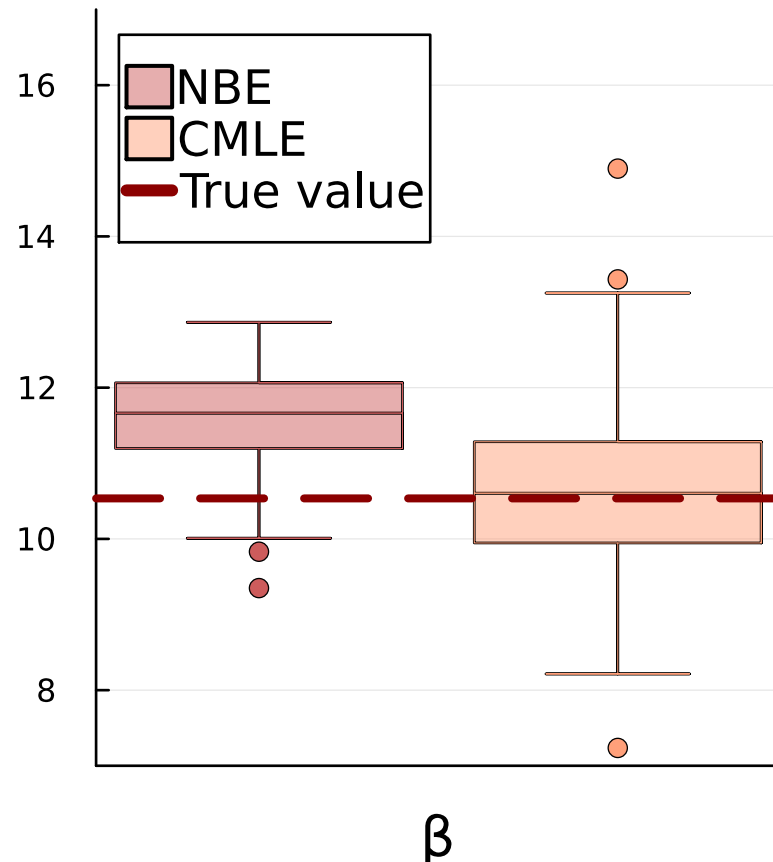

Estimates

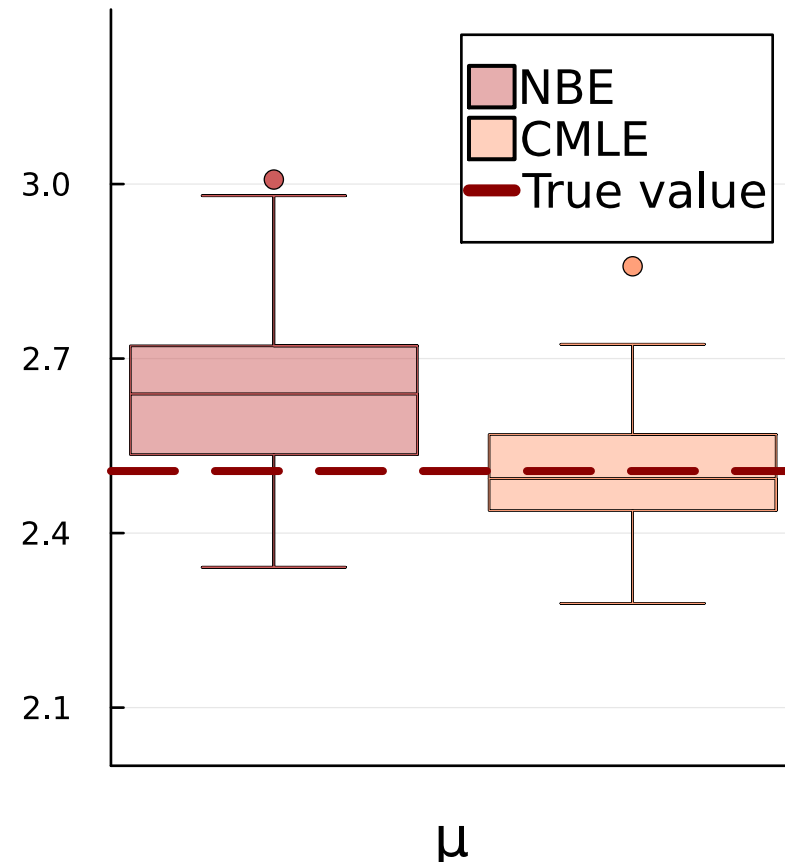

Supplement: Supplementary file 1 — (zip 5334 KB) [file 10687_2025_521_MOESM1_ESM.zip › SupplementaryMaterial/Images/box1_eng1.pdf]

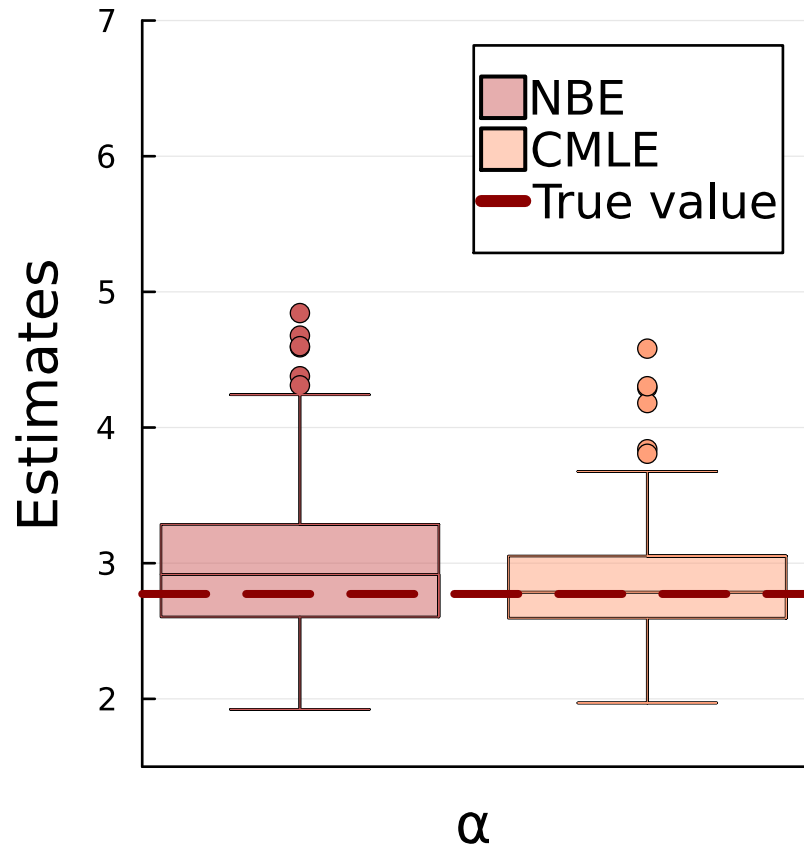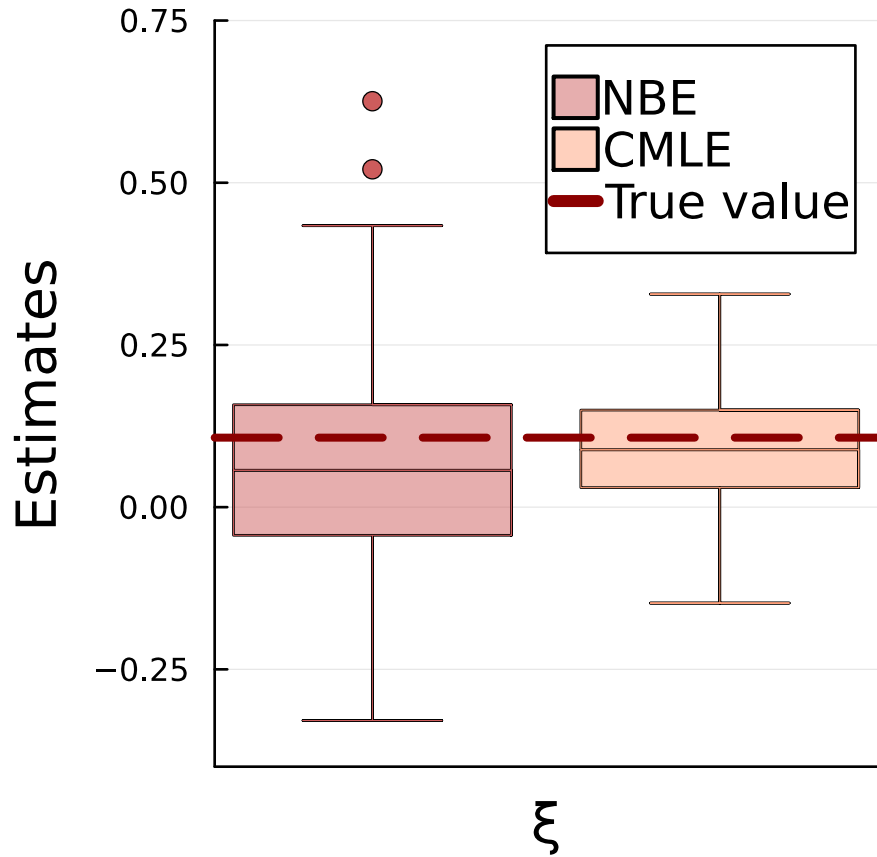

Supplement: Supplementary file 1 — (zip 5334 KB) [file 10687_2025_521_MOESM1_ESM.zip › SupplementaryMaterial/Images/box1_eng2.pdf]

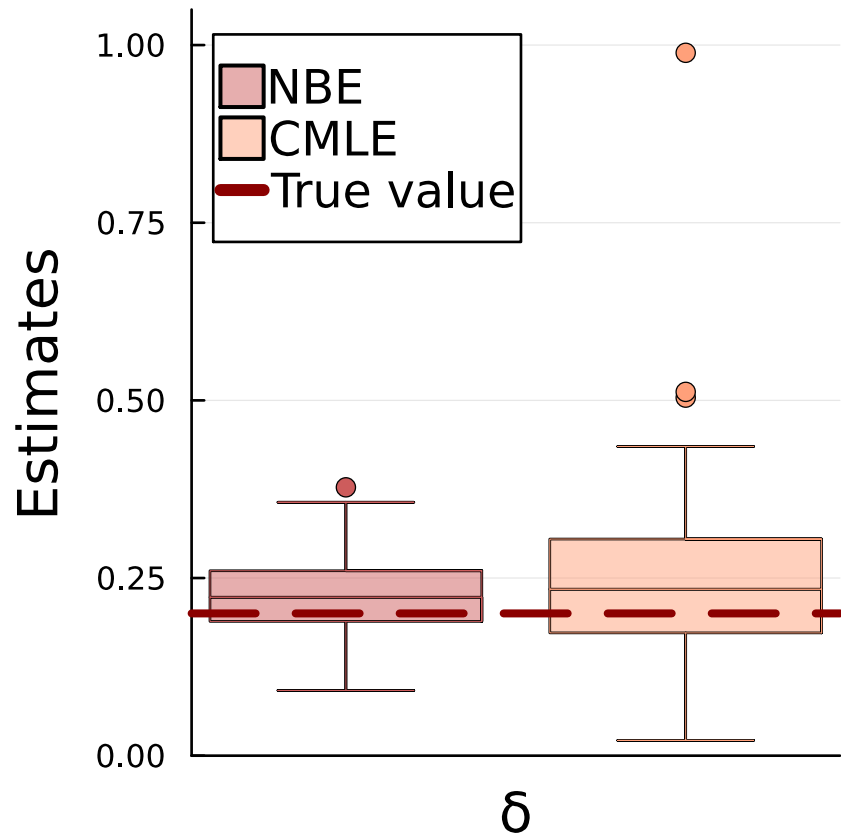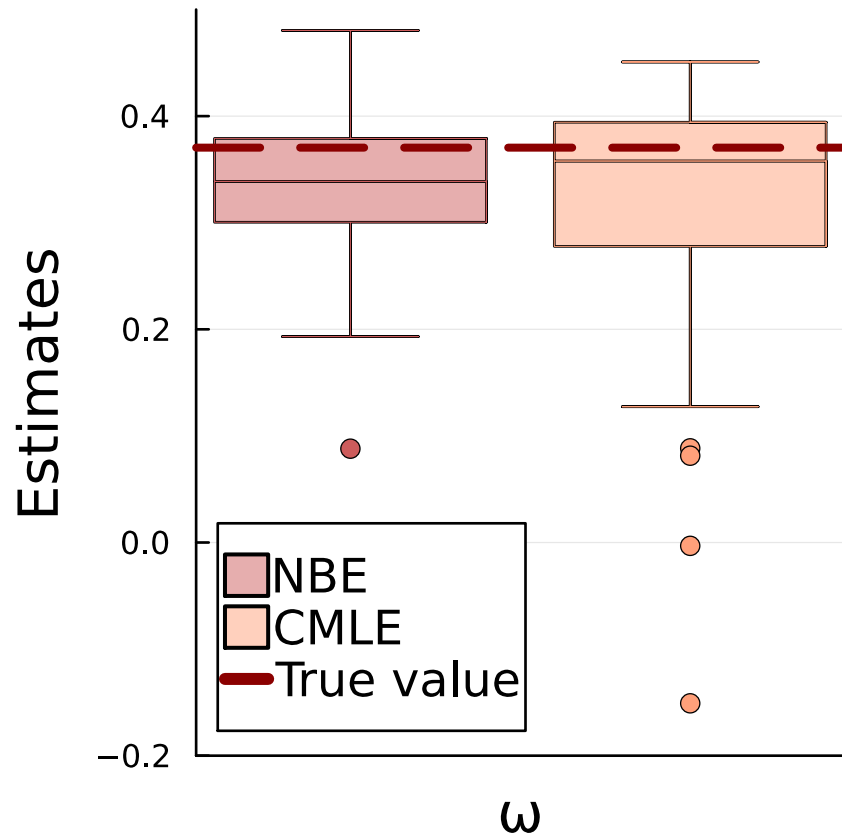

Supplement: Supplementary file 1 — (zip 5334 KB) [file 10687_2025_521_MOESM1_ESM.zip › SupplementaryMaterial/Images/box1_hwGauss.pdf]

Estimates

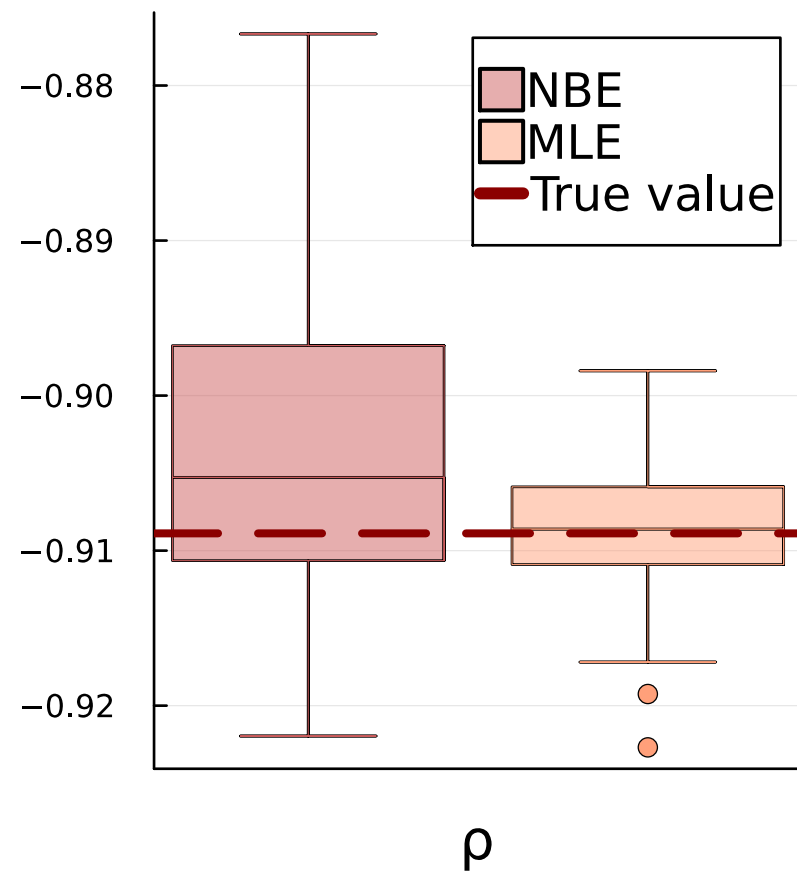

Estimates

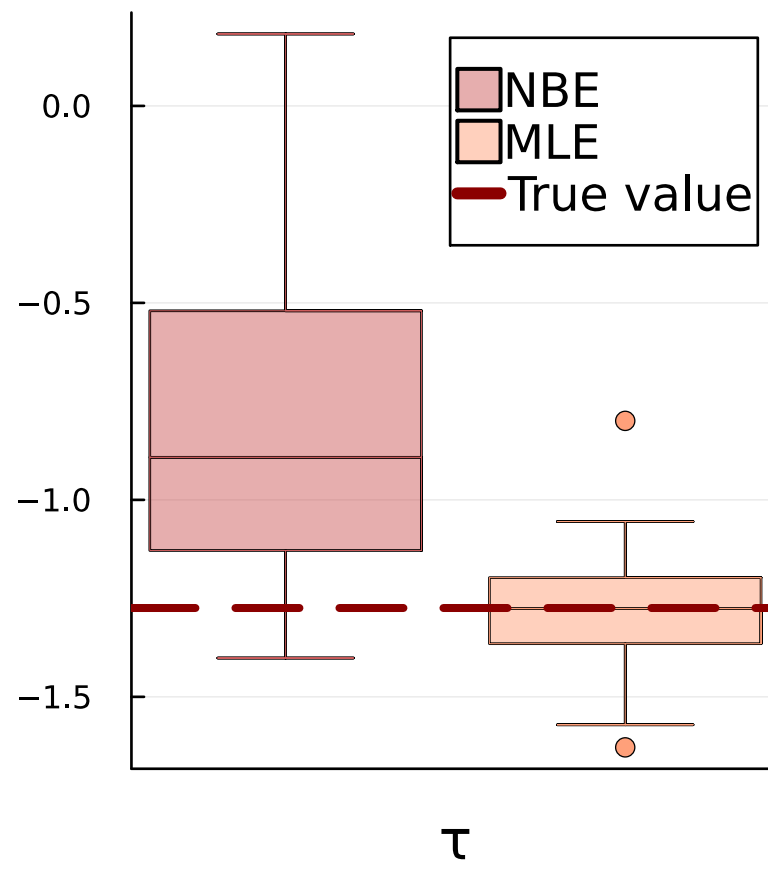

Estimates

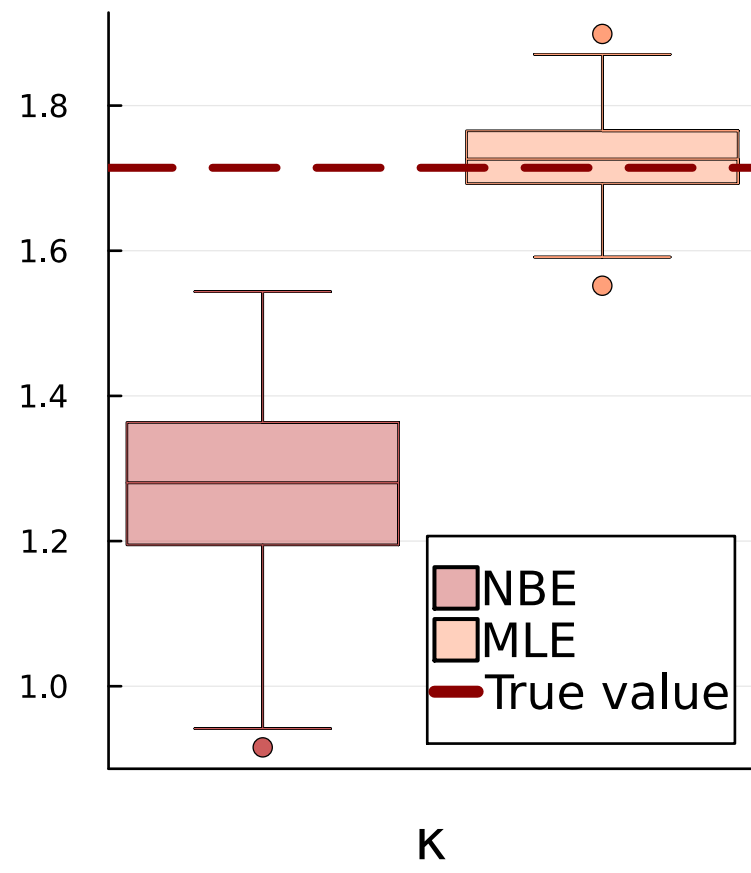

Supplement: Supplementary file 1 — (zip 5334 KB) [file 10687_2025_521_MOESM1_ESM.zip › SupplementaryMaterial/Images/box1_mod1.pdf]

Estimates

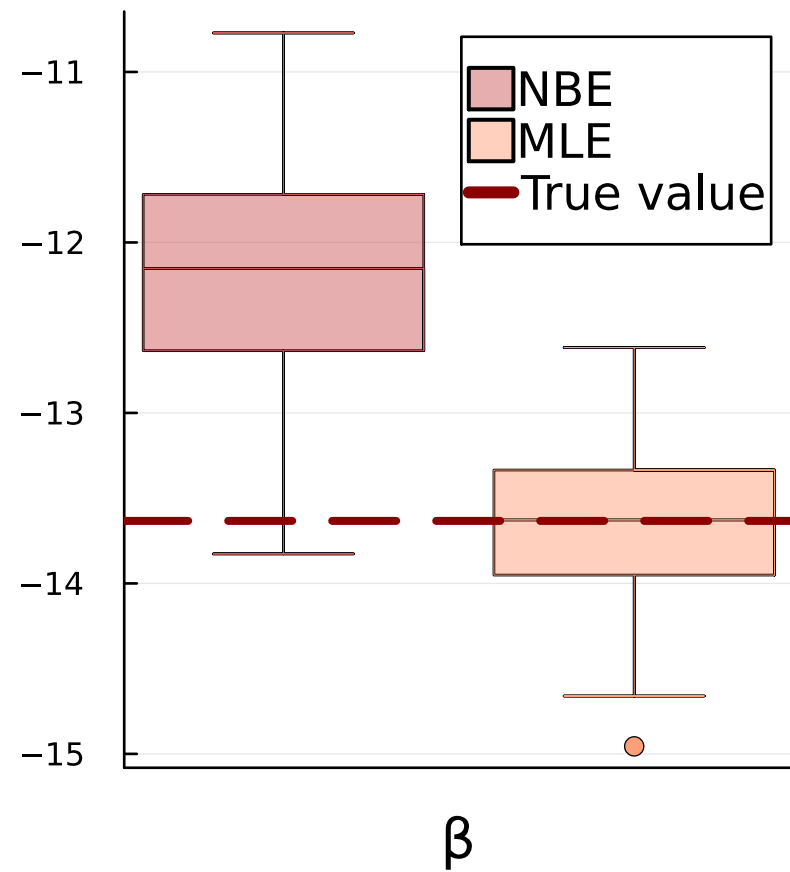

Estimates

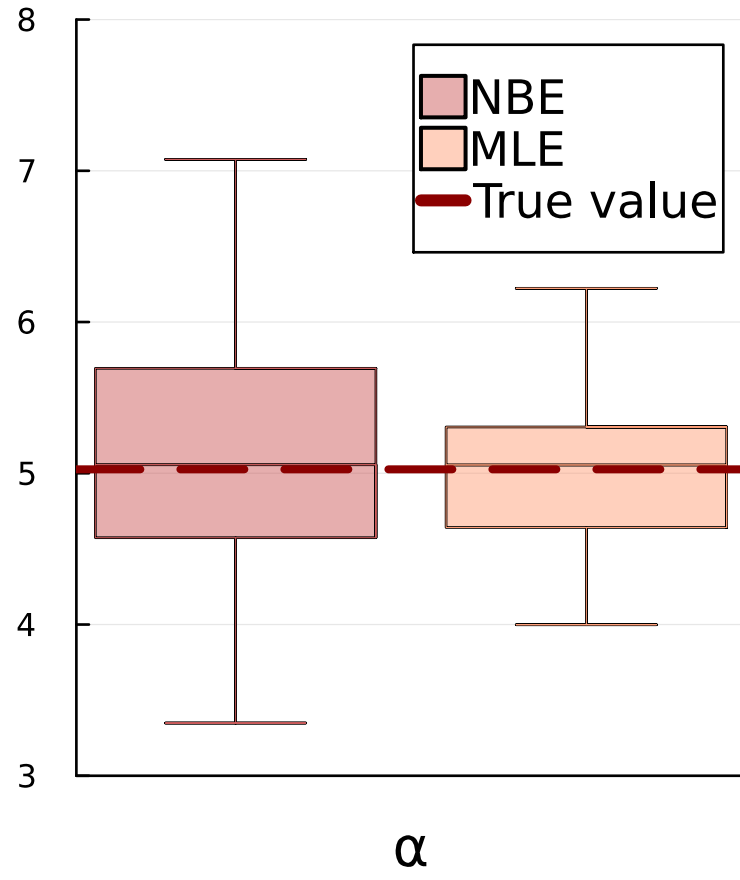

Estimates

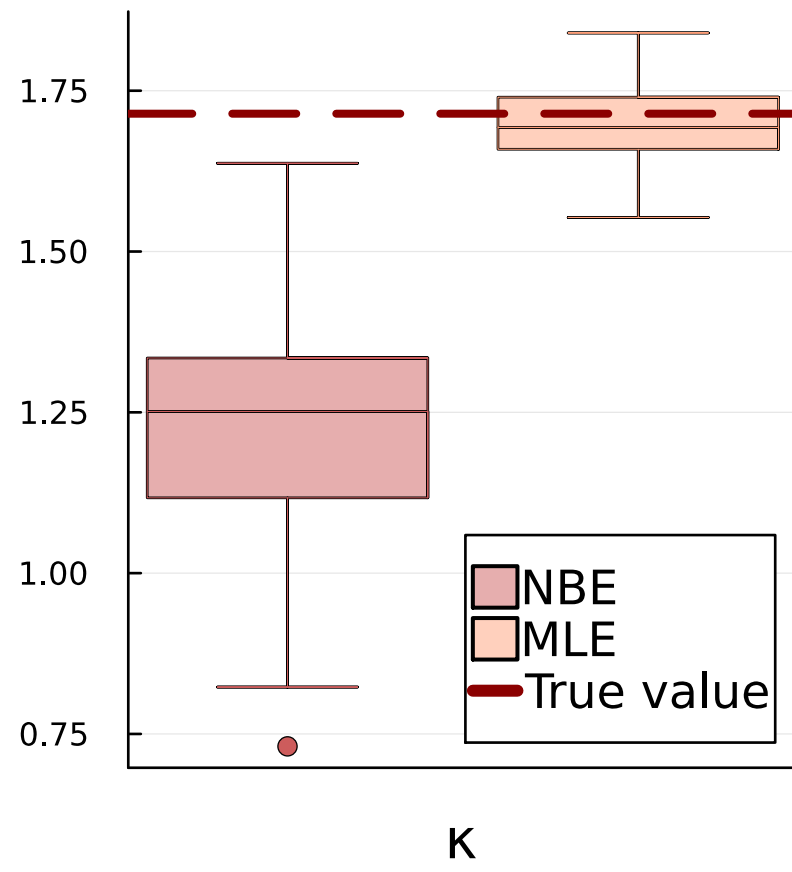

Supplement: Supplementary file 1 — (zip 5334 KB) [file 10687_2025_521_MOESM1_ESM.zip › SupplementaryMaterial/Images/box1_mod2.pdf]

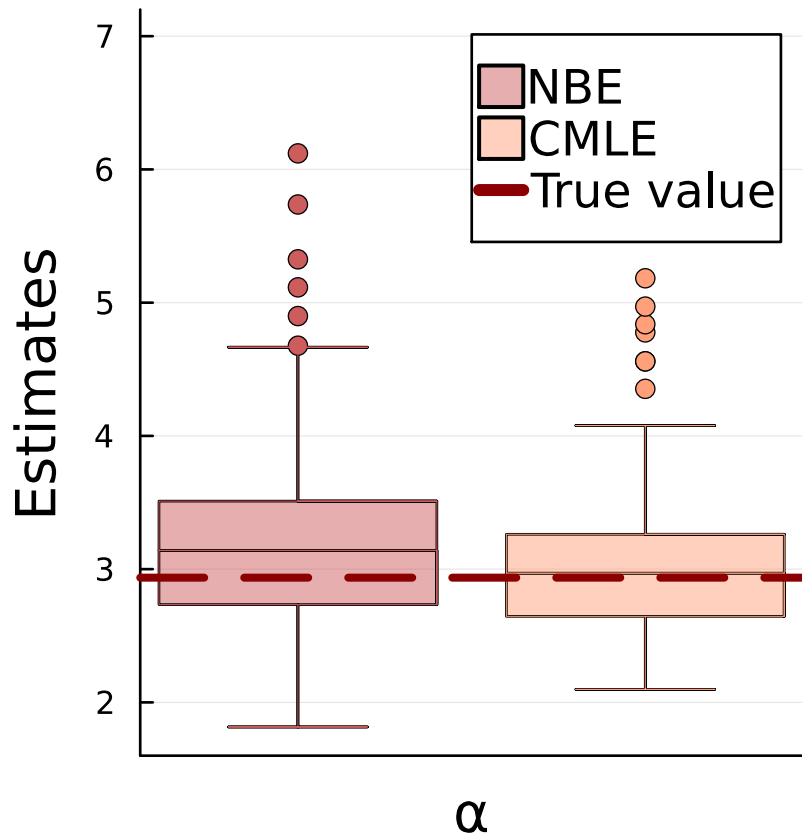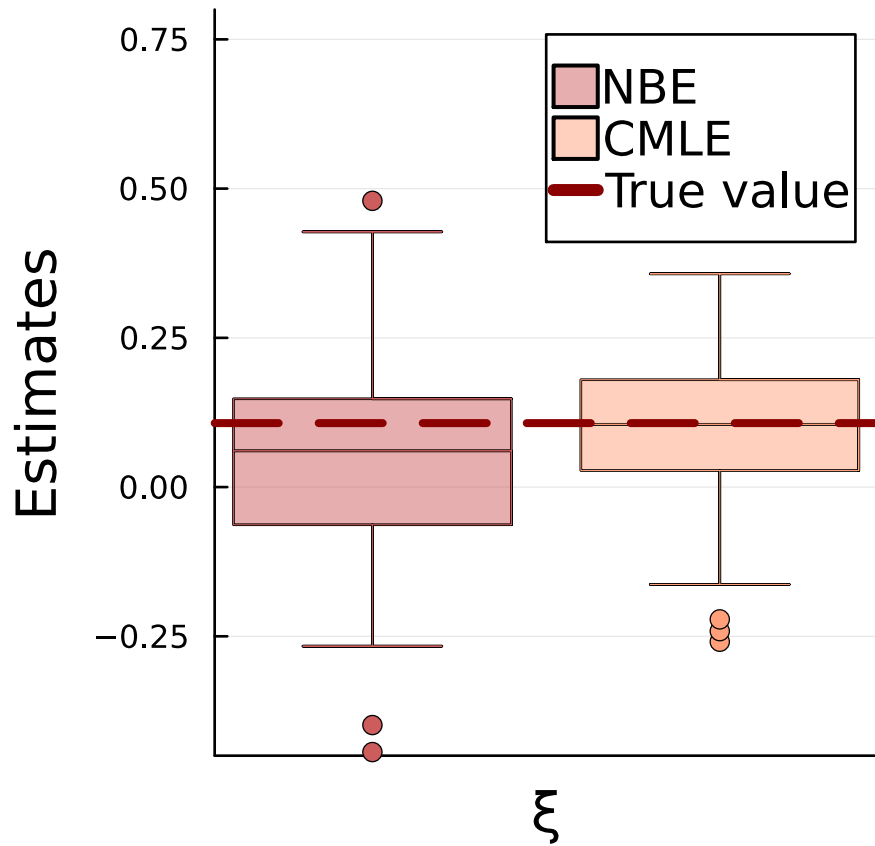

Supplement: Supplementary file 1 — (zip 5334 KB) [file 10687_2025_521_MOESM1_ESM.zip › SupplementaryMaterial/Images/box1_wadsfixed.pdf]

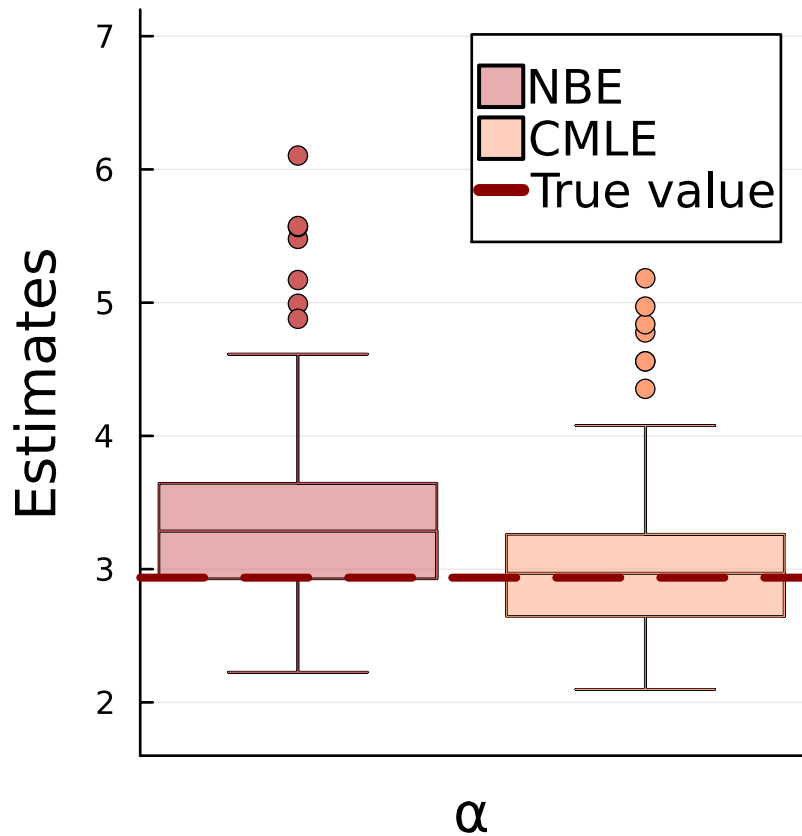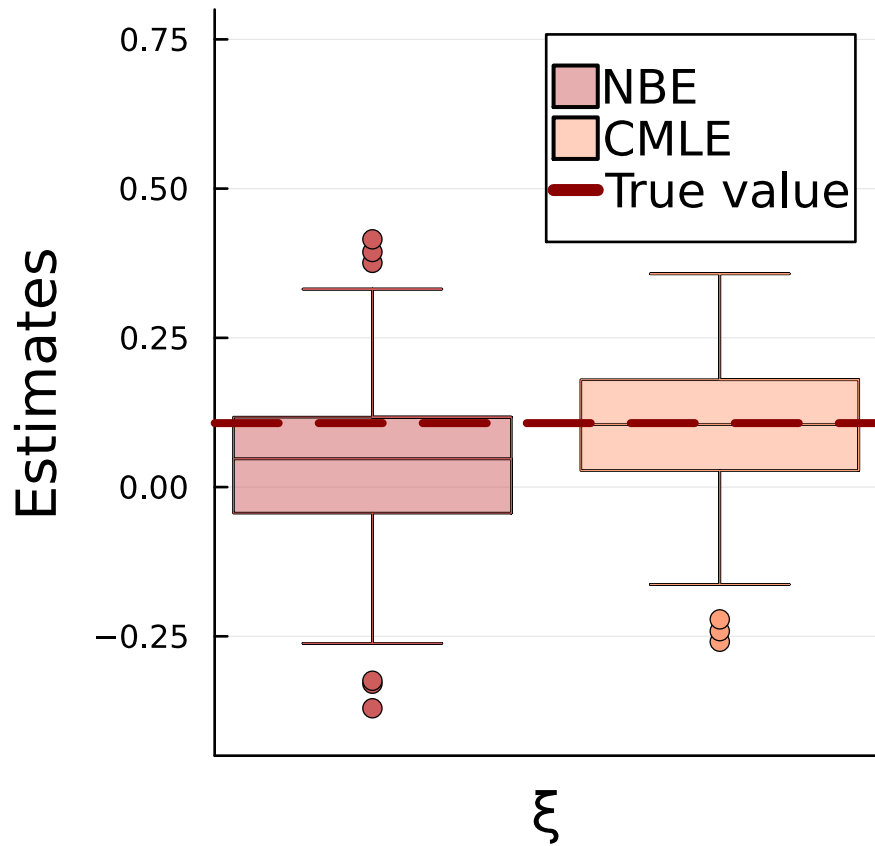

Supplement: Supplementary file 1 — (zip 5334 KB) [file 10687_2025_521_MOESM1_ESM.zip › SupplementaryMaterial/Images/box1_wadsfixedvarn.pdf]

Estimates

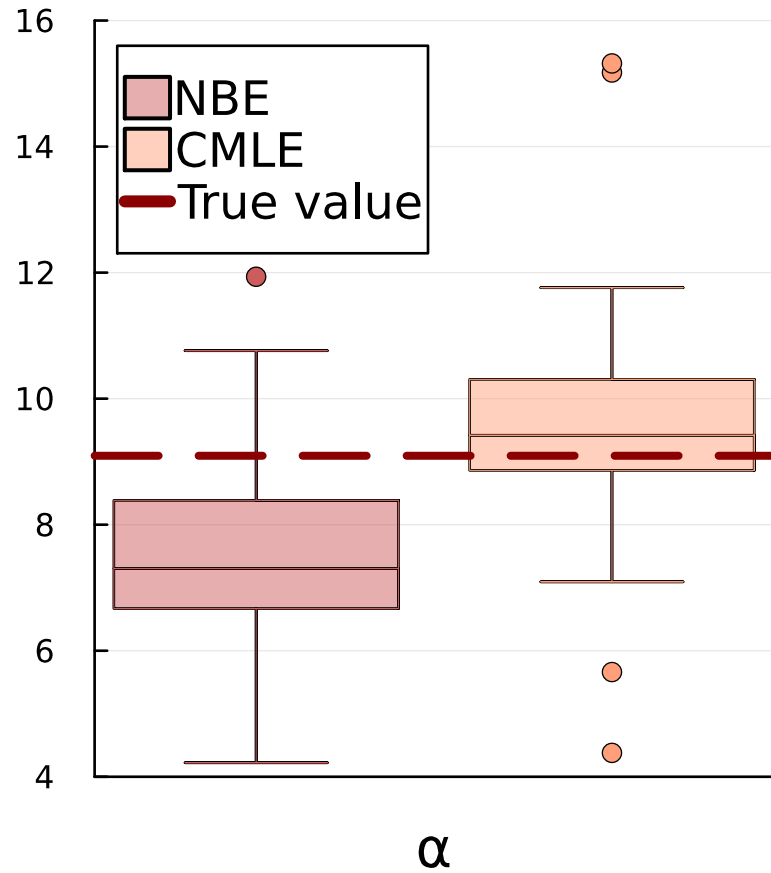

Estimates

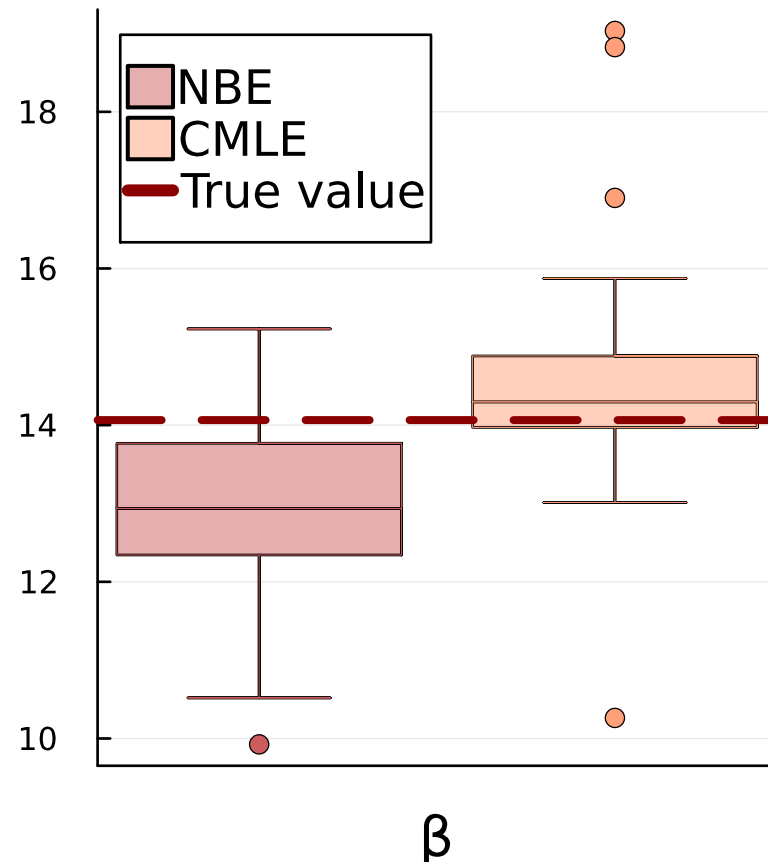

Estimates

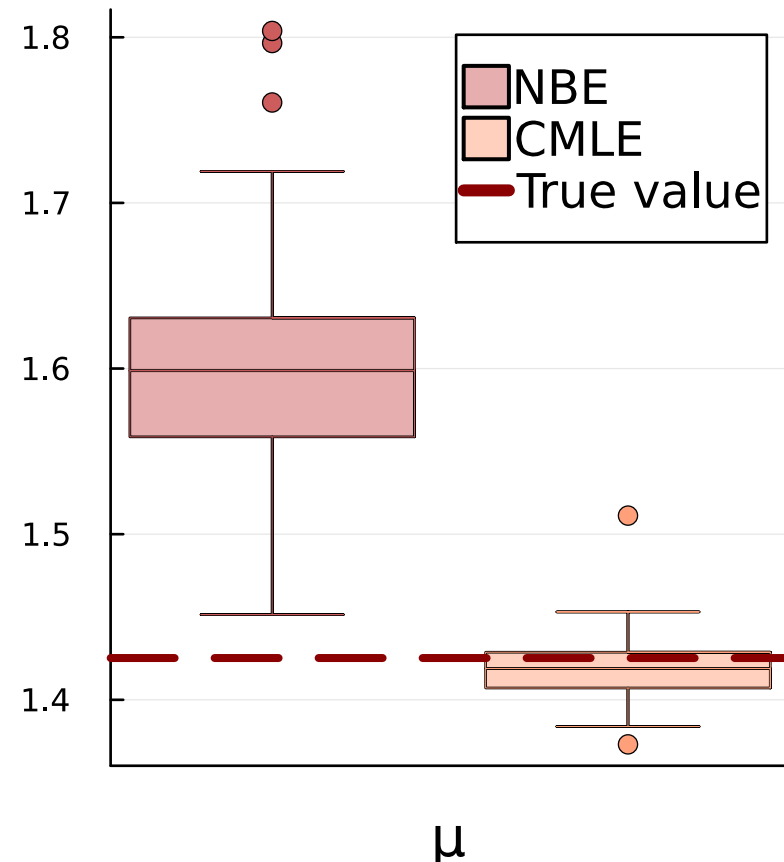

Supplement: Supplementary file 1 — (zip 5334 KB) [file 10687_2025_521_MOESM1_ESM.zip › SupplementaryMaterial/Images/box2_eng1.pdf]

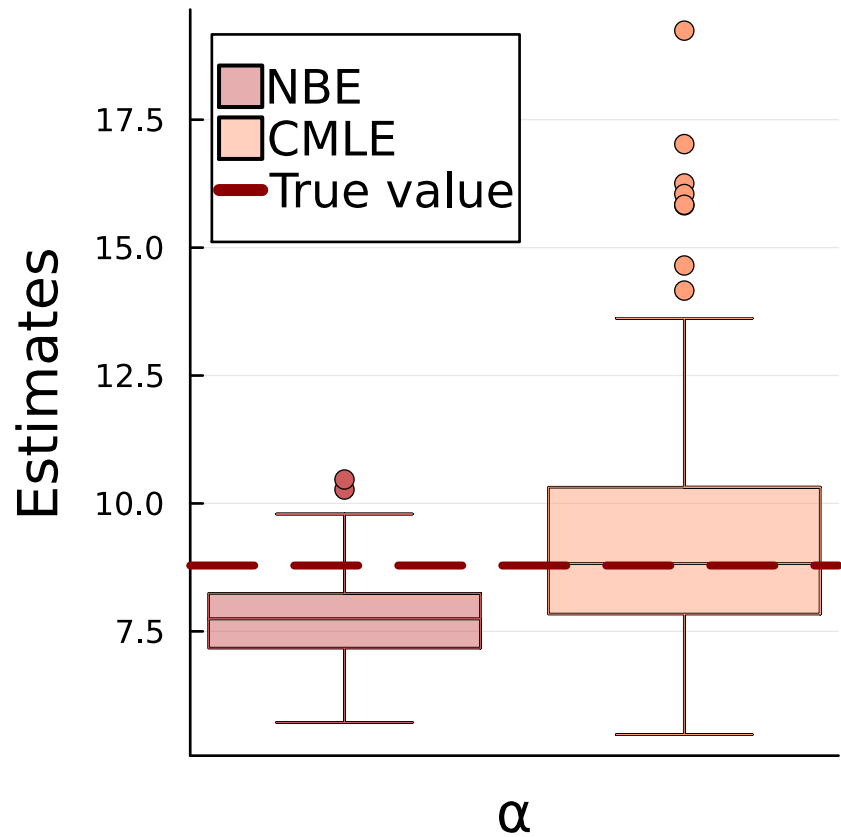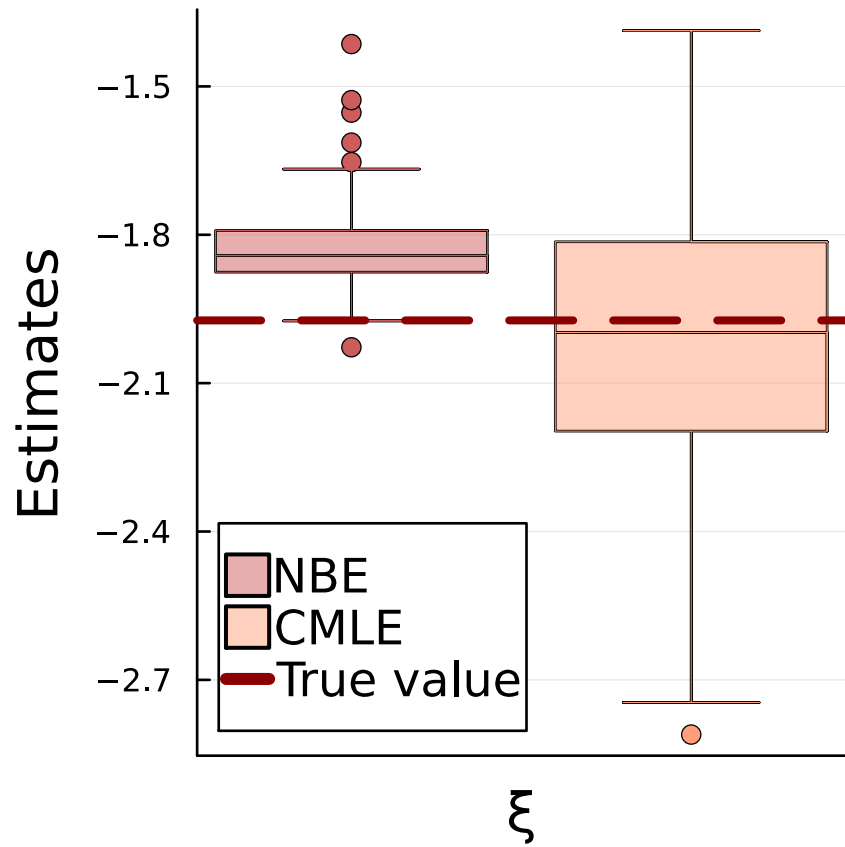

Supplement: Supplementary file 1 — (zip 5334 KB) [file 10687_2025_521_MOESM1_ESM.zip › SupplementaryMaterial/Images/box2_eng2.pdf]

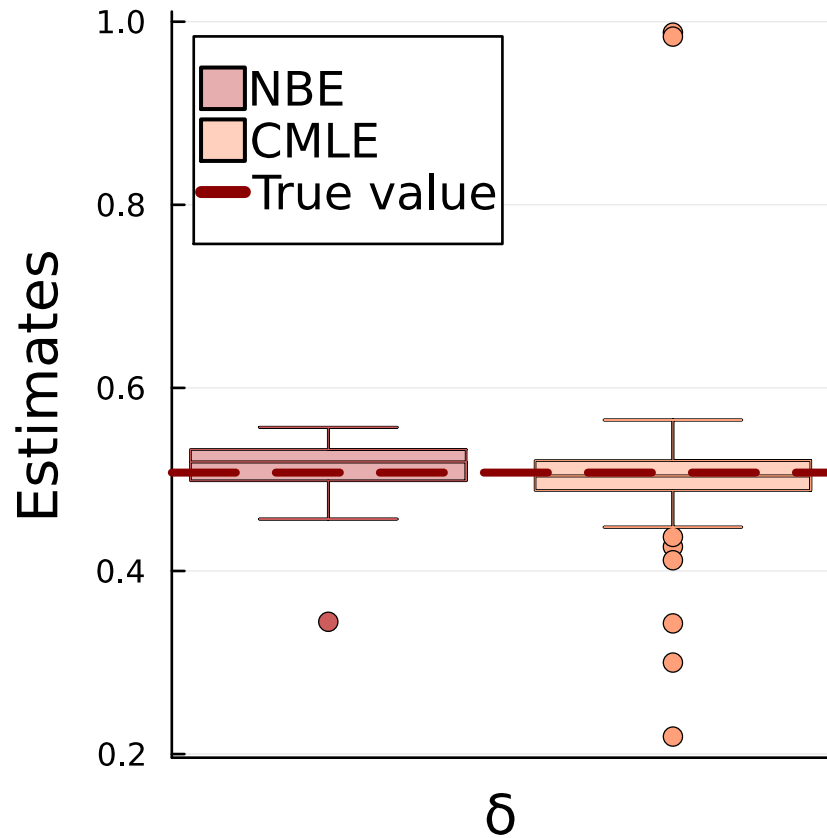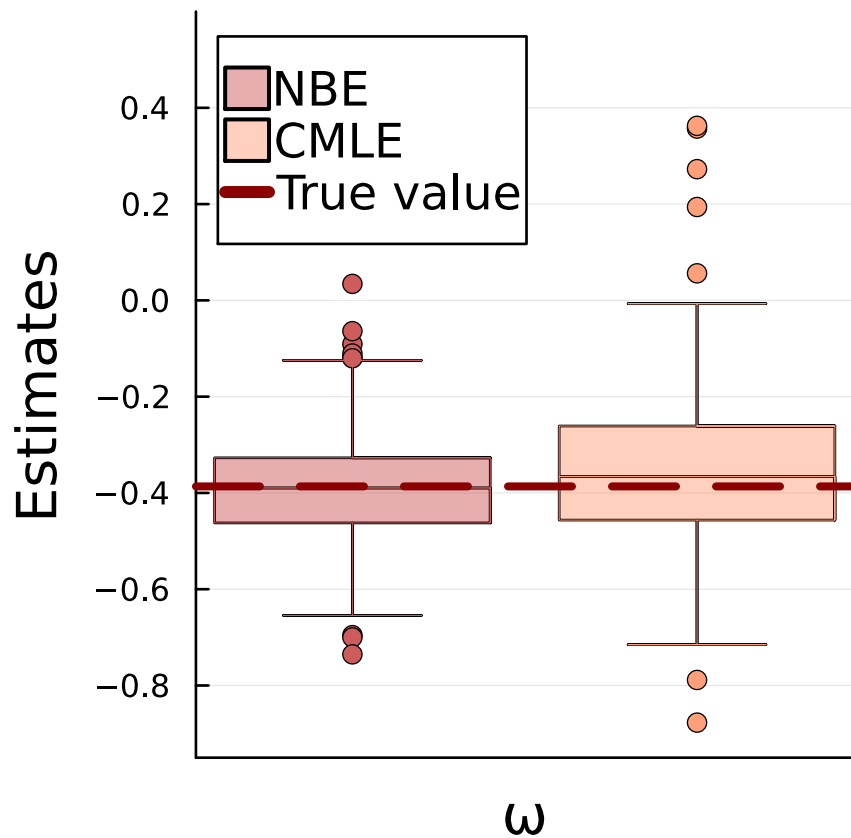

Supplement: Supplementary file 1 — (zip 5334 KB) [file 10687_2025_521_MOESM1_ESM.zip › SupplementaryMaterial/Images/box2_hwGauss.pdf]

Estimates

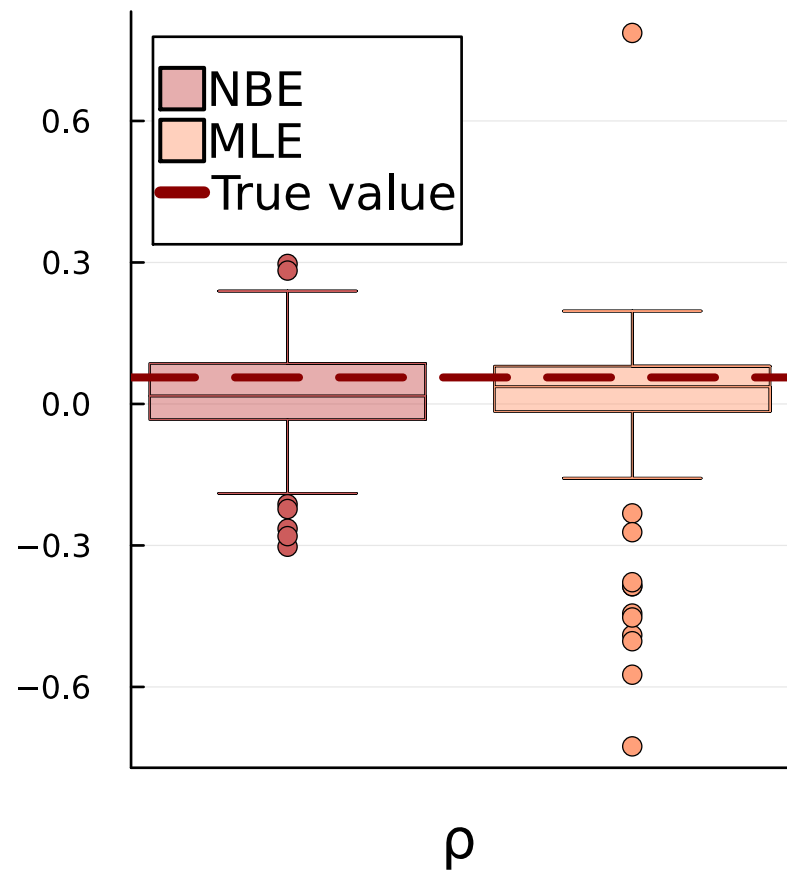

Estimates

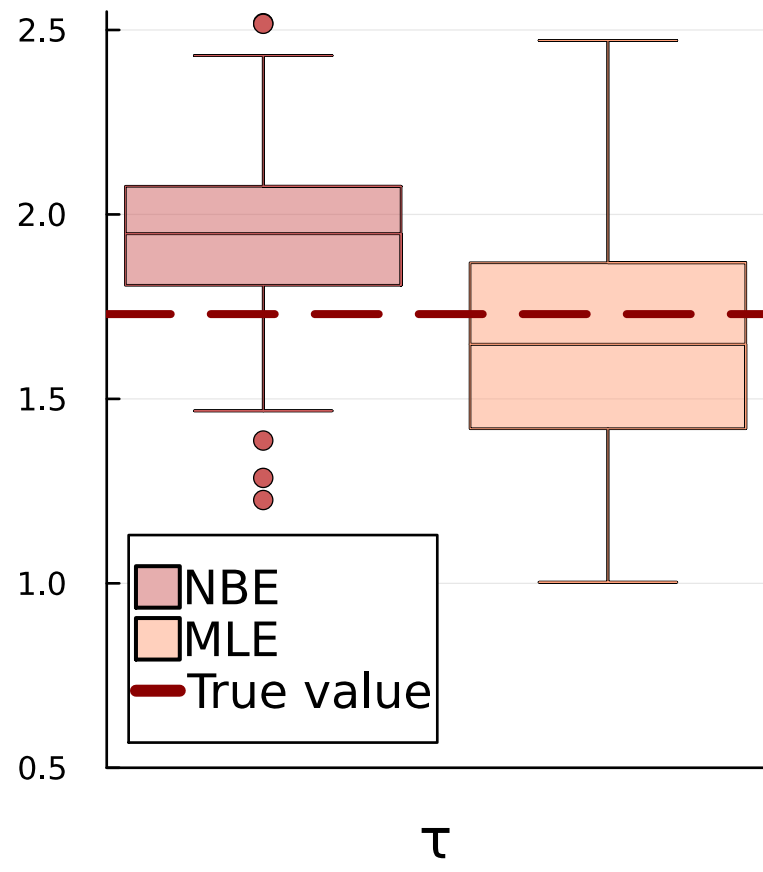

Estimates

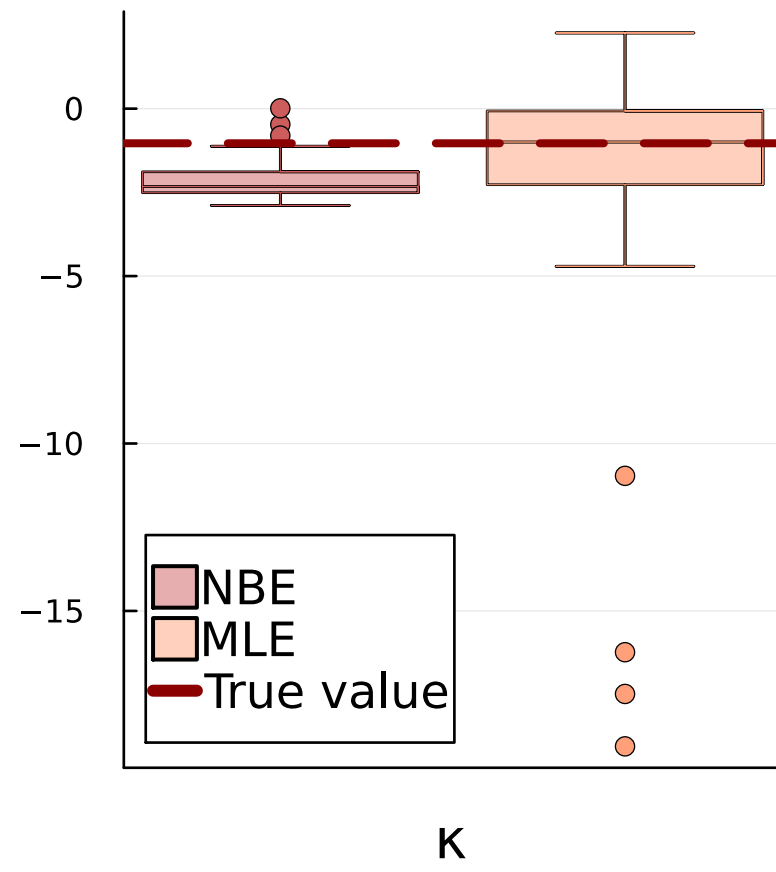

Supplement: Supplementary file 1 — (zip 5334 KB) [file 10687_2025_521_MOESM1_ESM.zip › SupplementaryMaterial/Images/box2_mod1.pdf]

Estimates

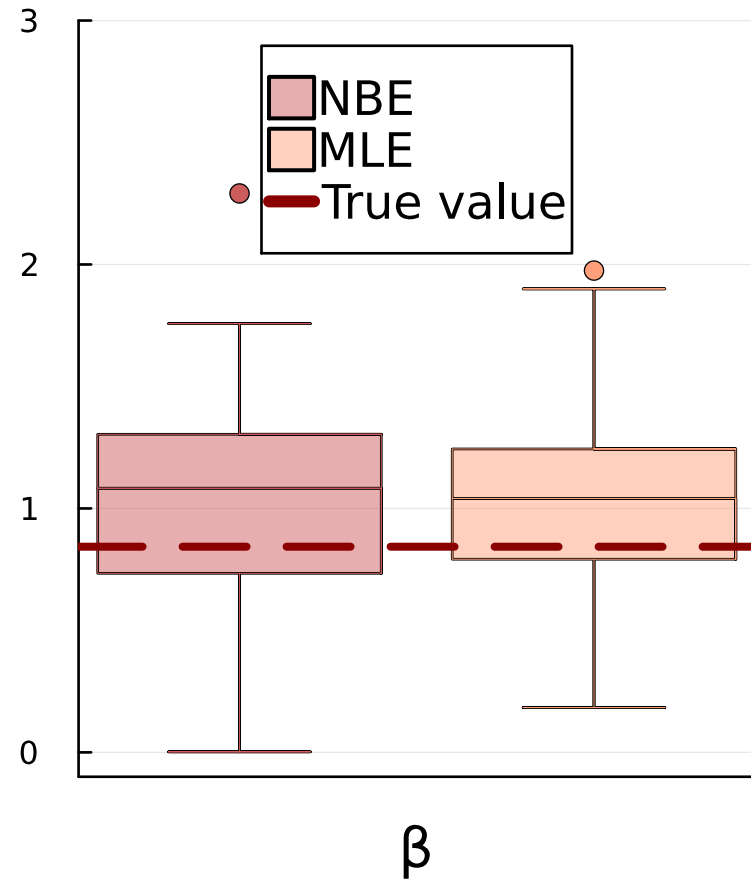

Estimates

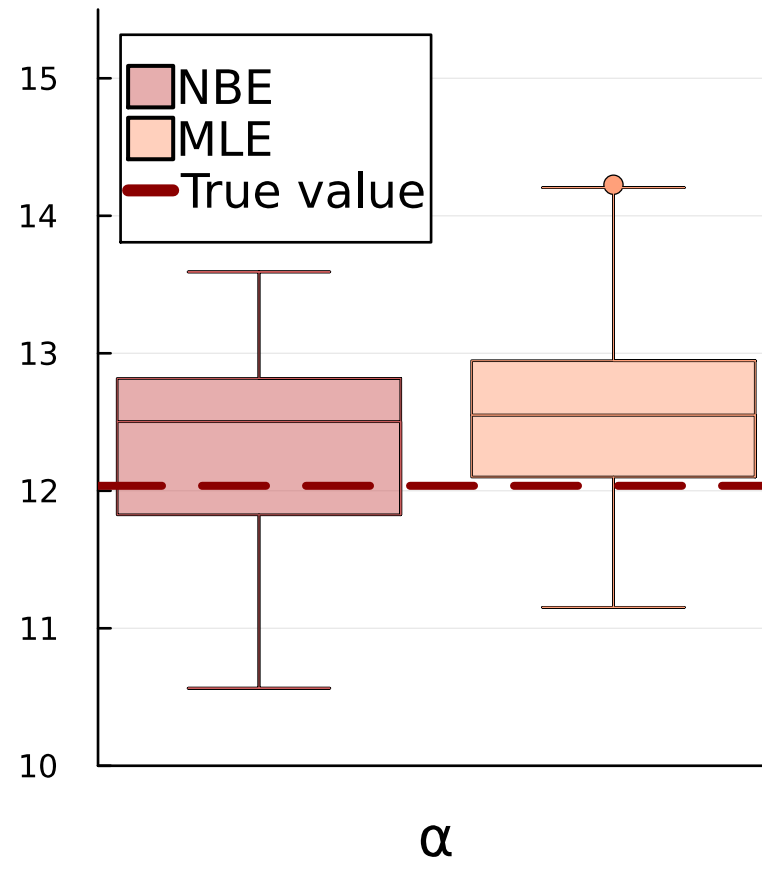

Estimates

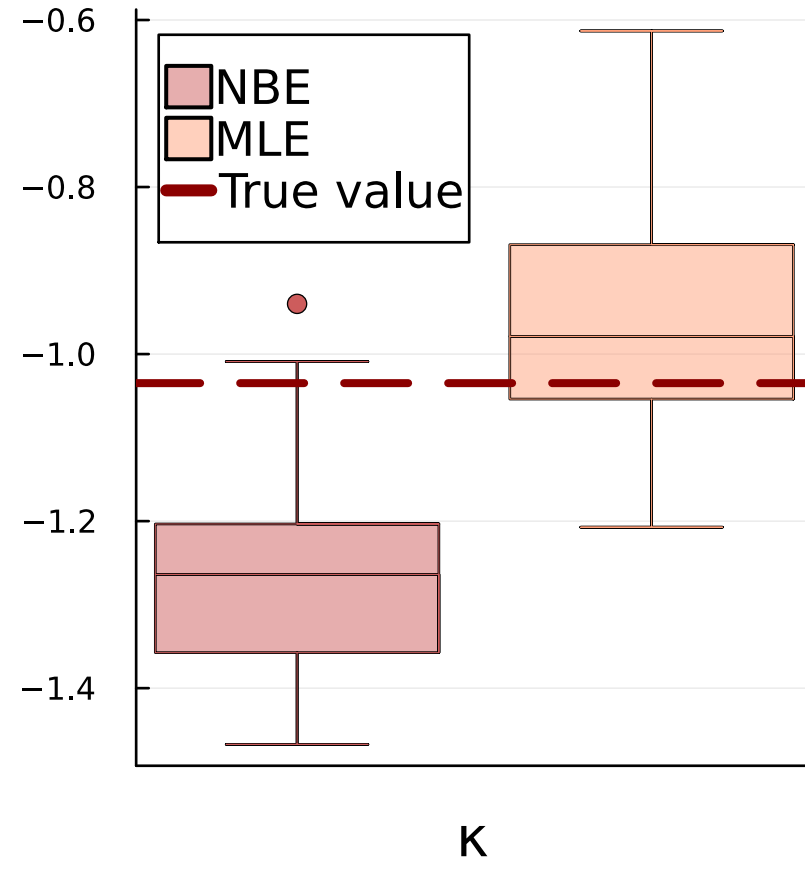

Supplement: Supplementary file 1 — (zip 5334 KB) [file 10687_2025_521_MOESM1_ESM.zip › SupplementaryMaterial/Images/box2_mod2.pdf]

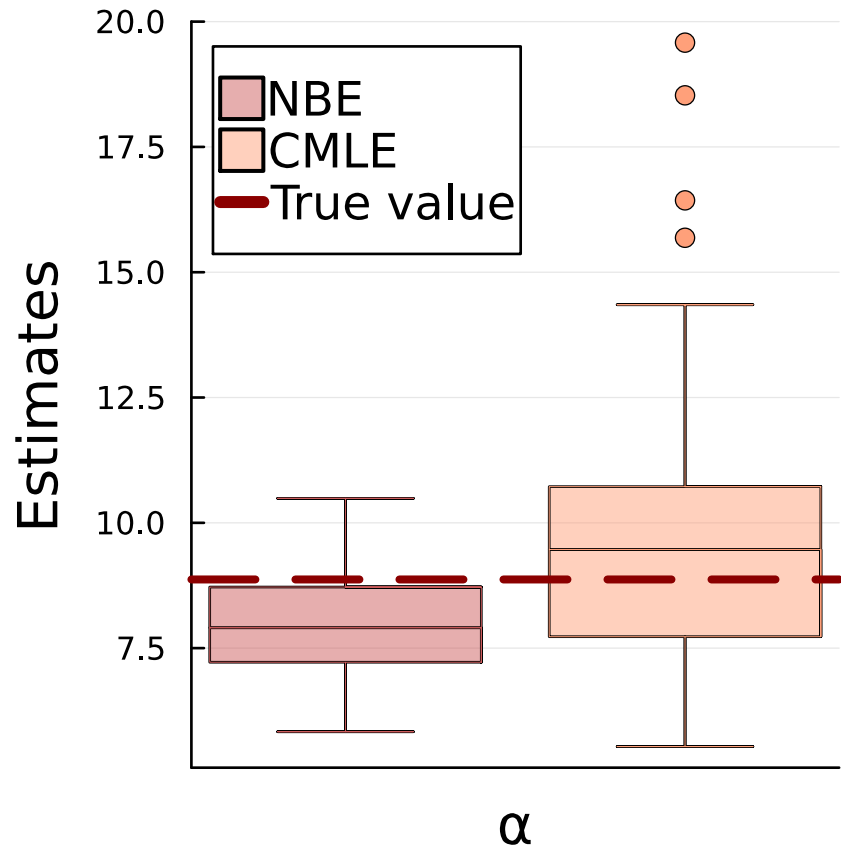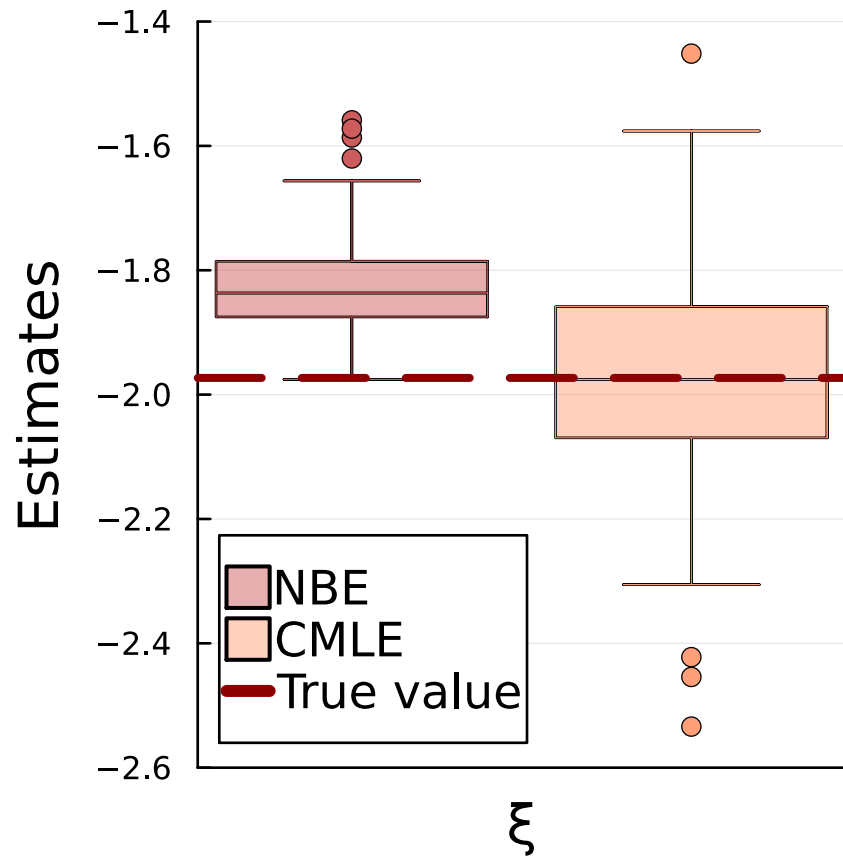

Supplement: Supplementary file 1 — (zip 5334 KB) [file 10687_2025_521_MOESM1_ESM.zip › SupplementaryMaterial/Images/box2_wadsfixed.pdf]

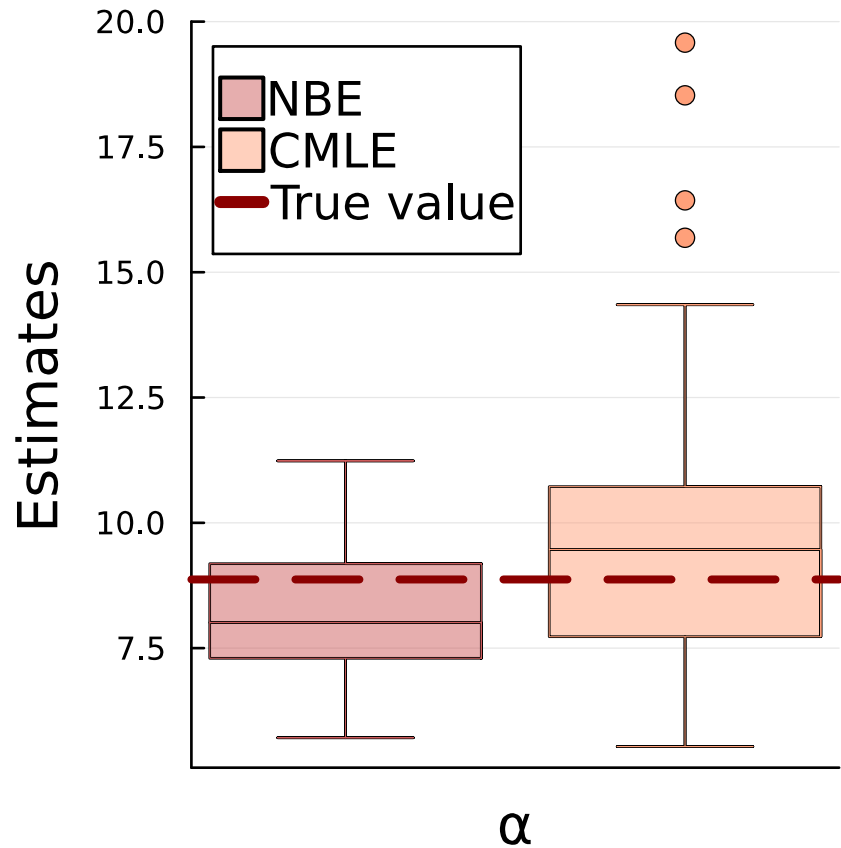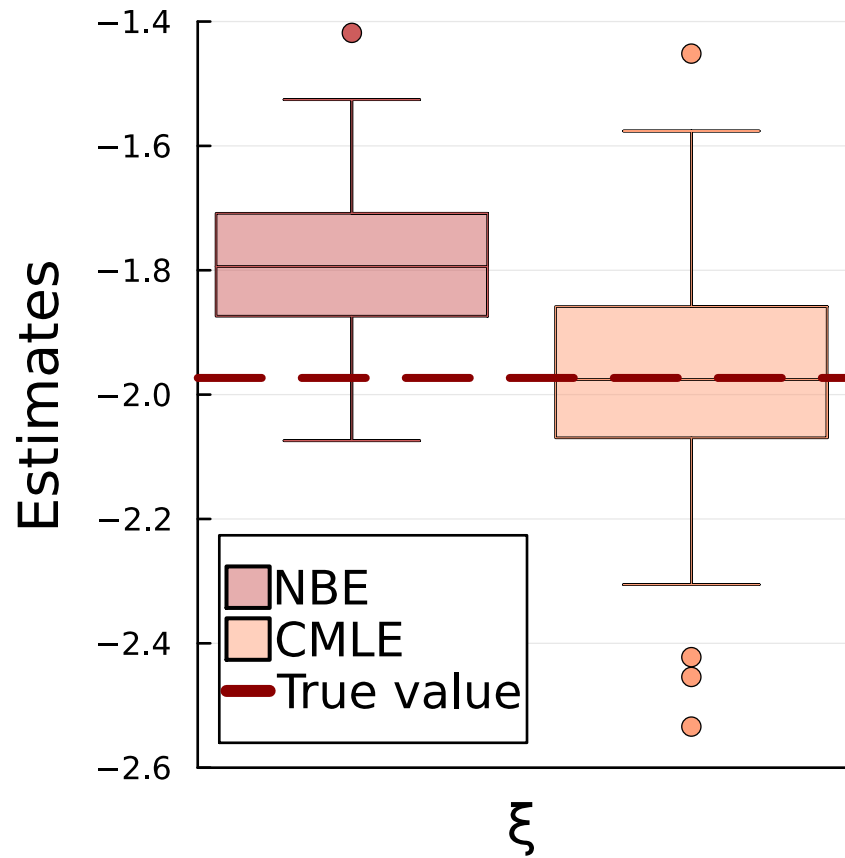

Supplement: Supplementary file 1 — (zip 5334 KB) [file 10687_2025_521_MOESM1_ESM.zip › SupplementaryMaterial/Images/box2_wadsfixedvarn.pdf]

Estimates

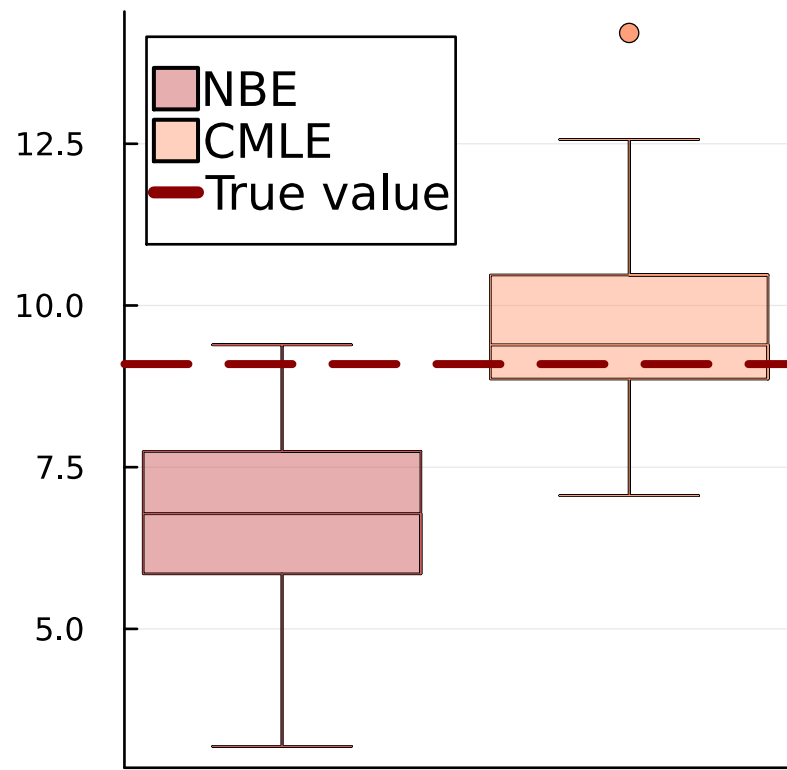

$\alpha$

Estimates

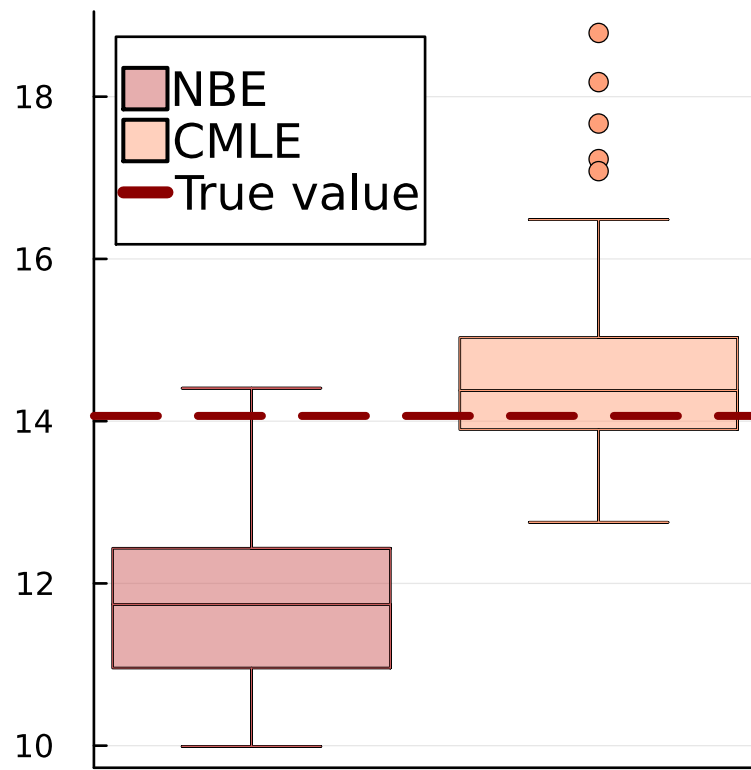

$\beta$

Estimates

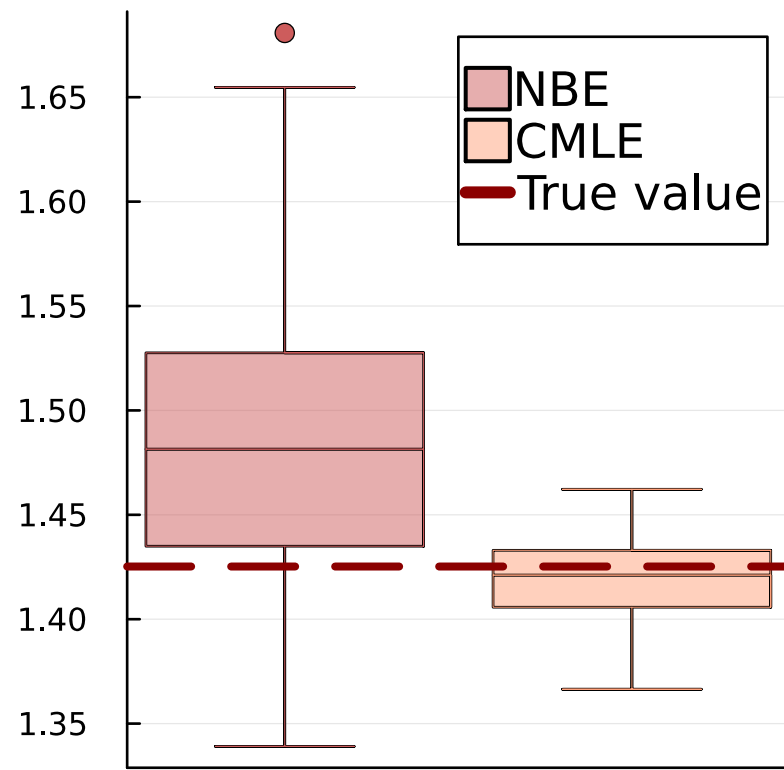

$\mu$

Supplement: Supplementary file 1 — (zip 5334 KB) [file 10687_2025_521_MOESM1_ESM.zip › SupplementaryMaterial/Images/box3_eng1.pdf]

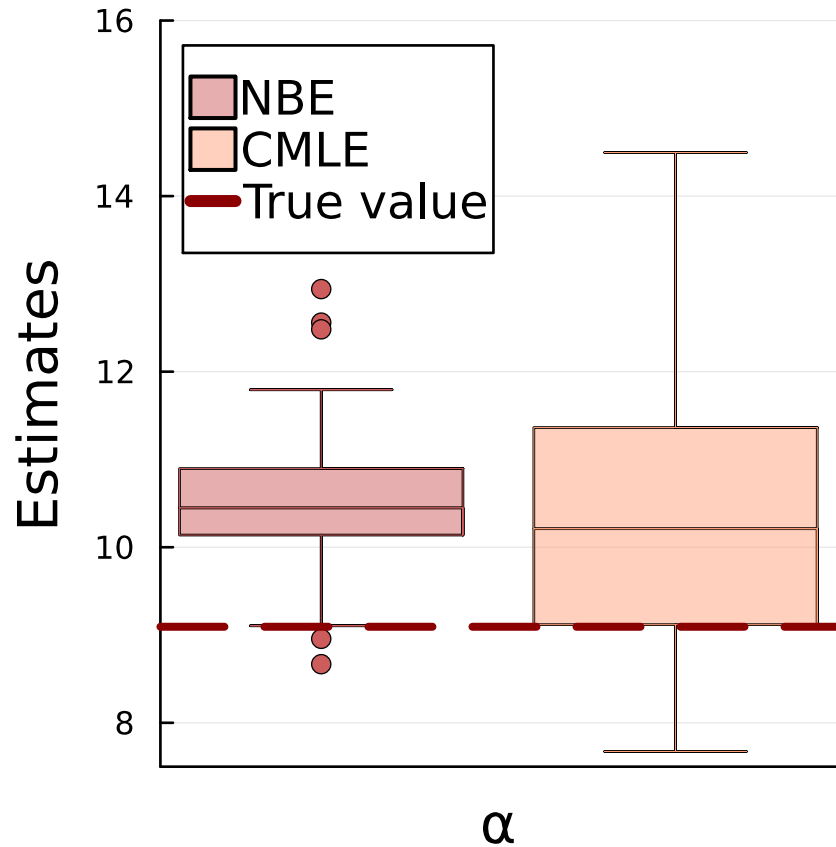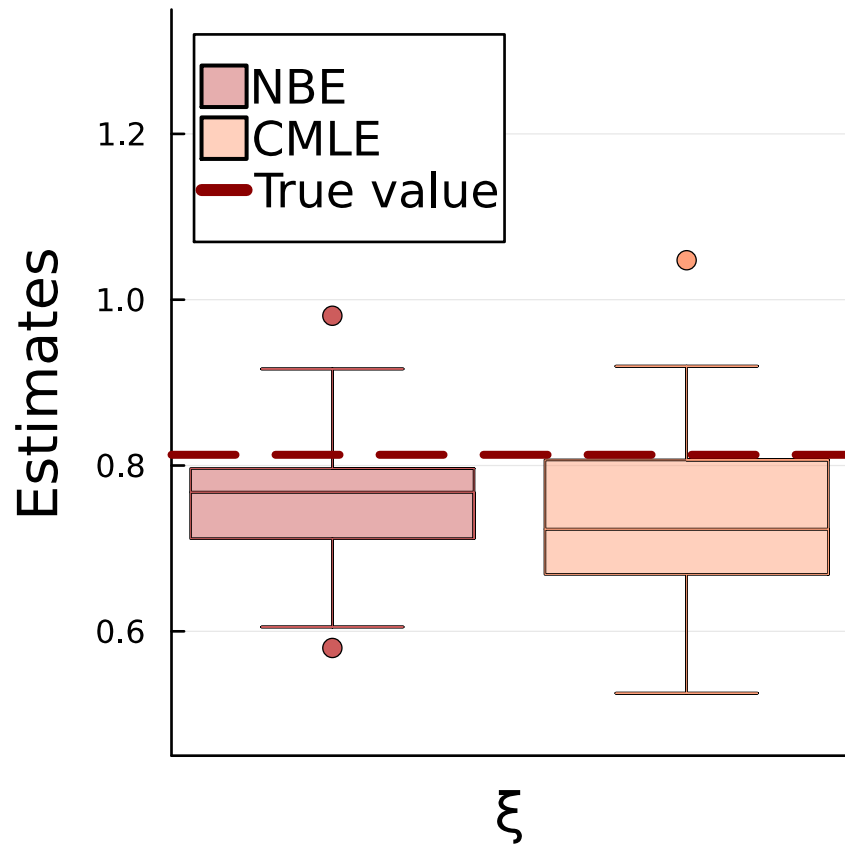

Supplement: Supplementary file 1 — (zip 5334 KB) [file 10687_2025_521_MOESM1_ESM.zip › SupplementaryMaterial/Images/box3_eng2.pdf]

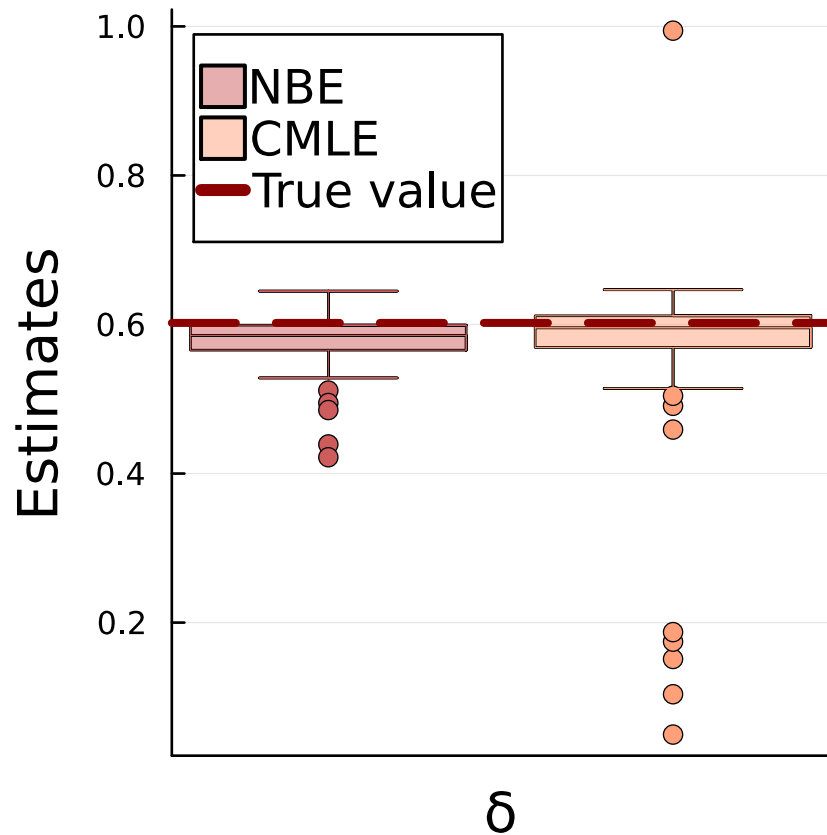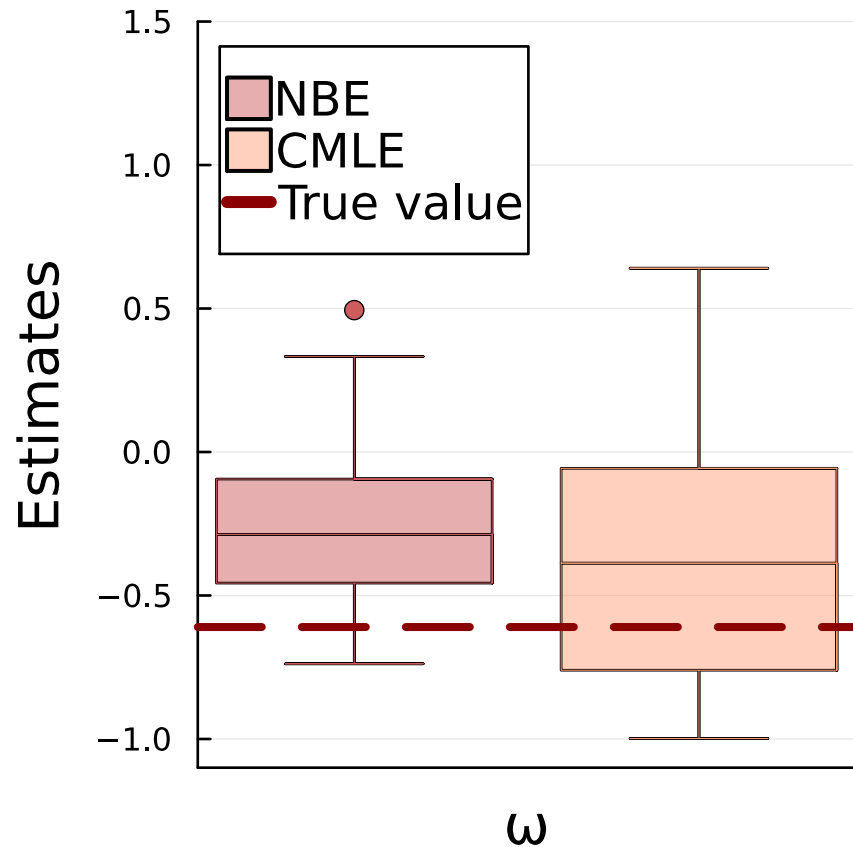

Supplement: Supplementary file 1 — (zip 5334 KB) [file 10687_2025_521_MOESM1_ESM.zip › SupplementaryMaterial/Images/box3_hwGauss.pdf]

Estimates

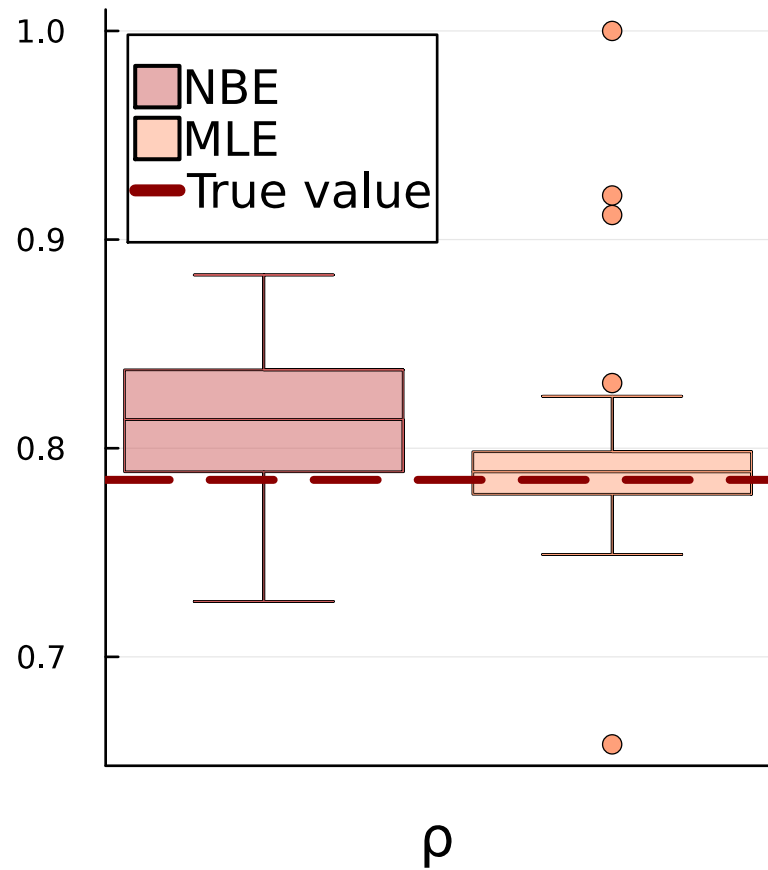

Estimates

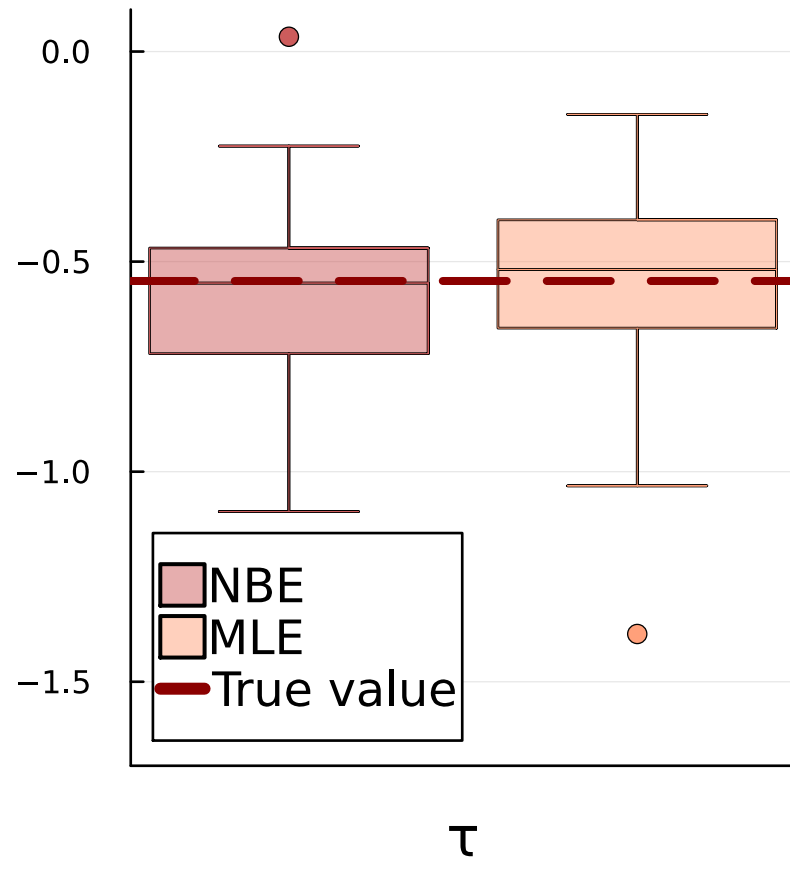

Estimates

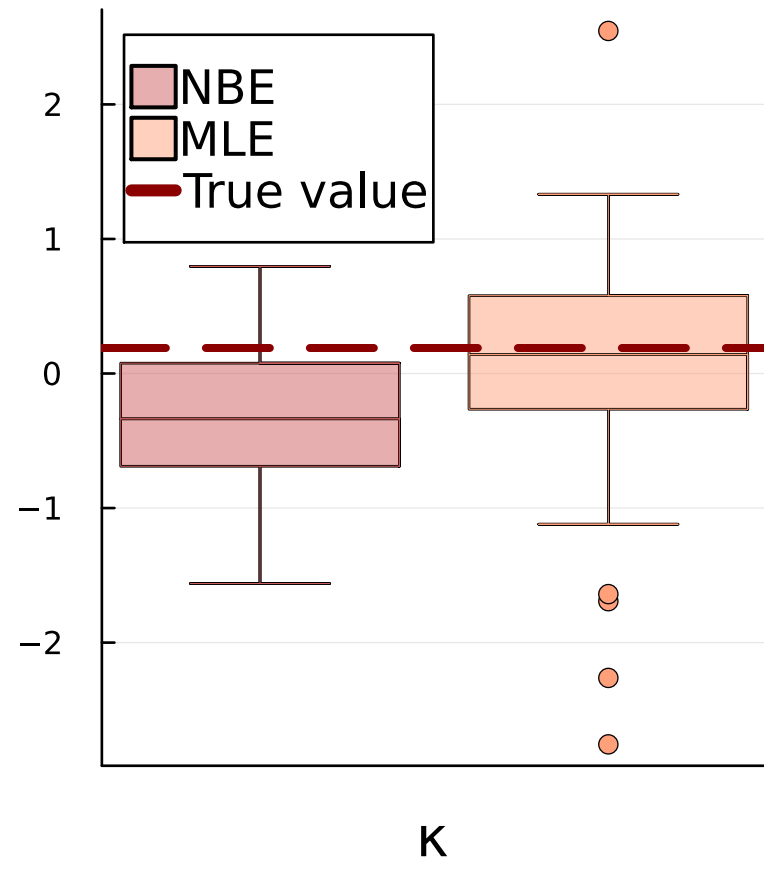

Supplement: Supplementary file 1 — (zip 5334 KB) [file 10687_2025_521_MOESM1_ESM.zip › SupplementaryMaterial/Images/box3_mod1.pdf]

Estimates

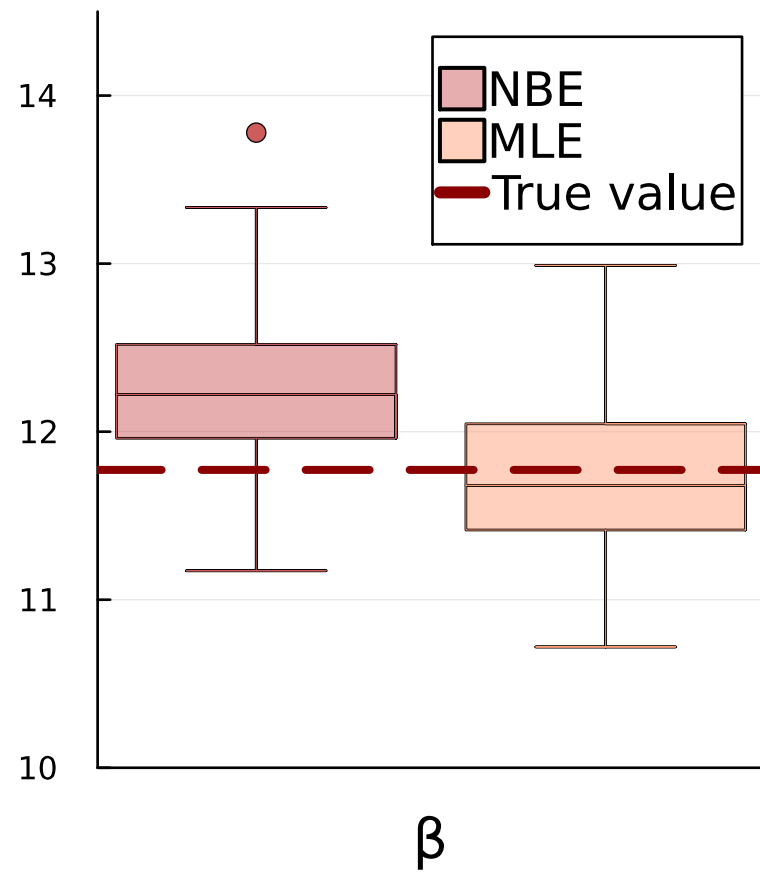

Estimates

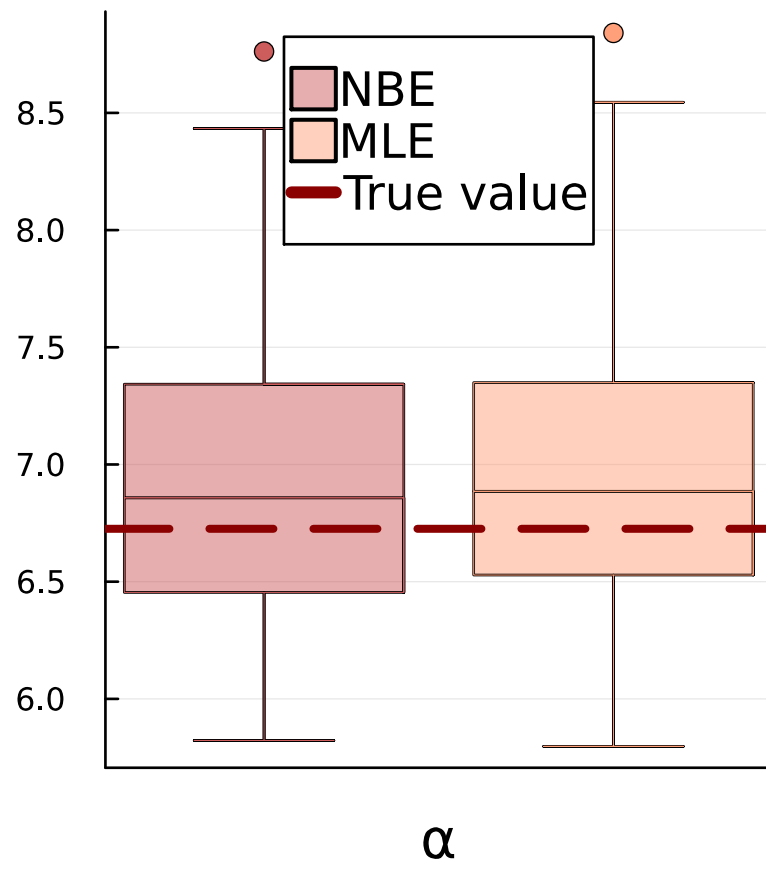

Estimates

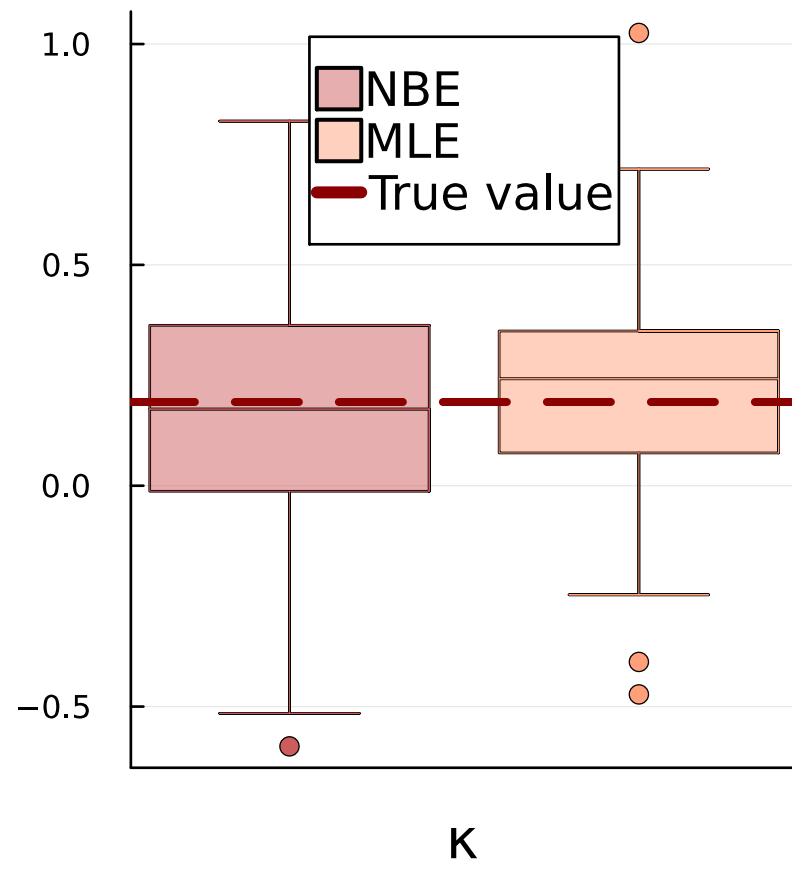

Supplement: Supplementary file 1 — (zip 5334 KB) [file 10687_2025_521_MOESM1_ESM.zip › SupplementaryMaterial/Images/box3_mod2.pdf]

Estimates

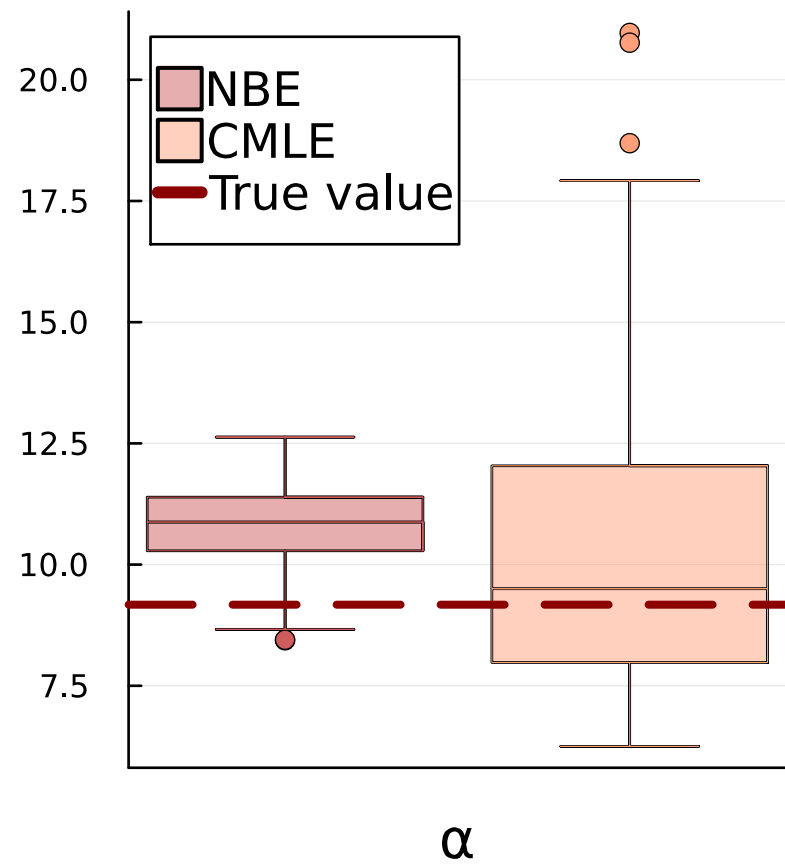

Estimates

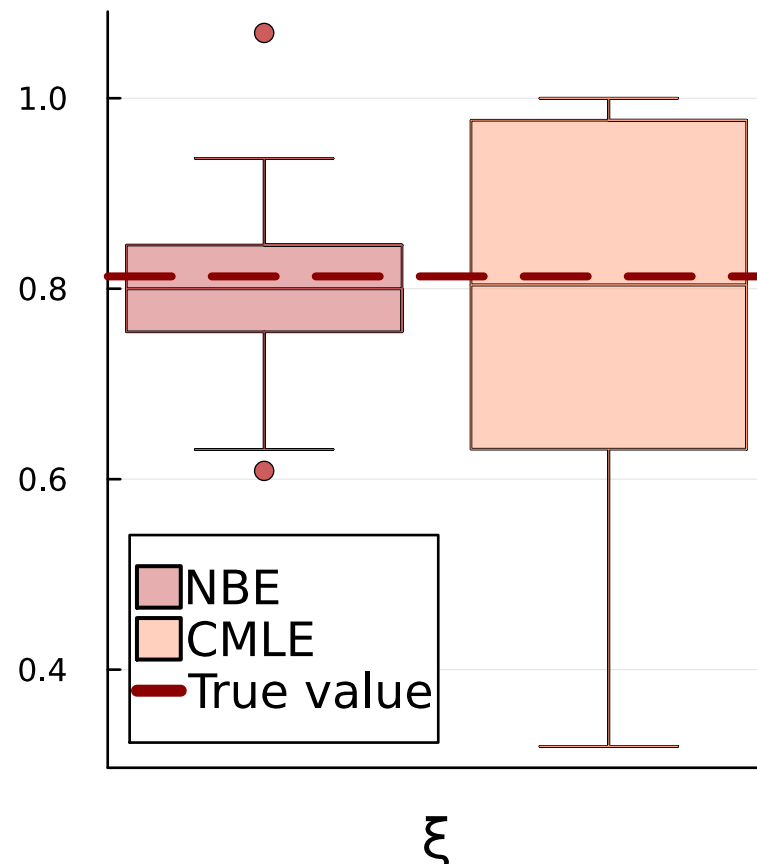

Estimates

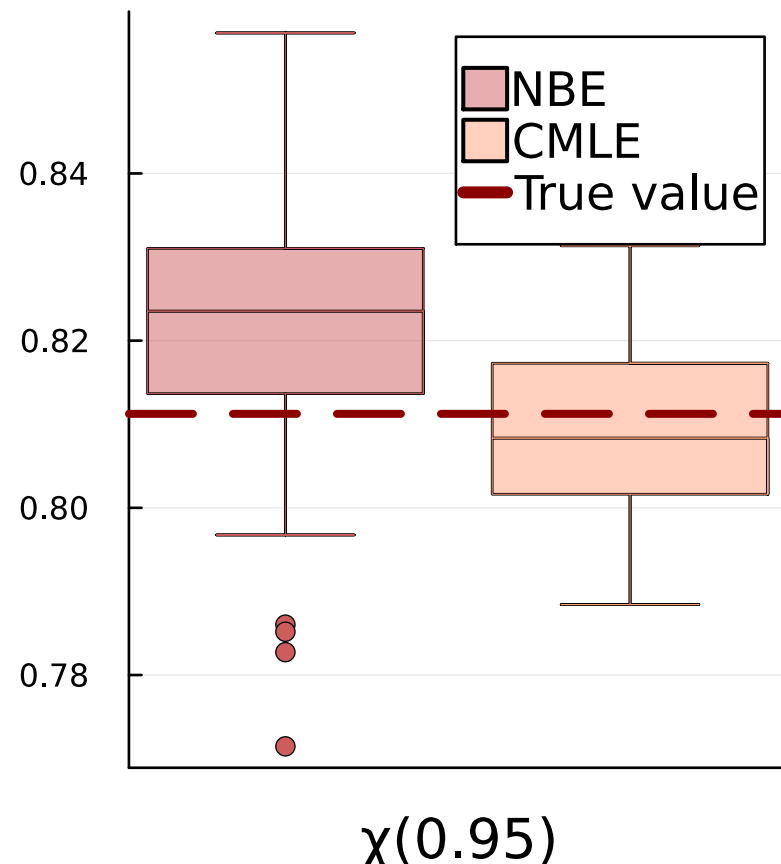

Supplement: Supplementary file 1 — (zip 5334 KB) [file 10687_2025_521_MOESM1_ESM.zip › SupplementaryMaterial/Images/box3_wads.pdf]

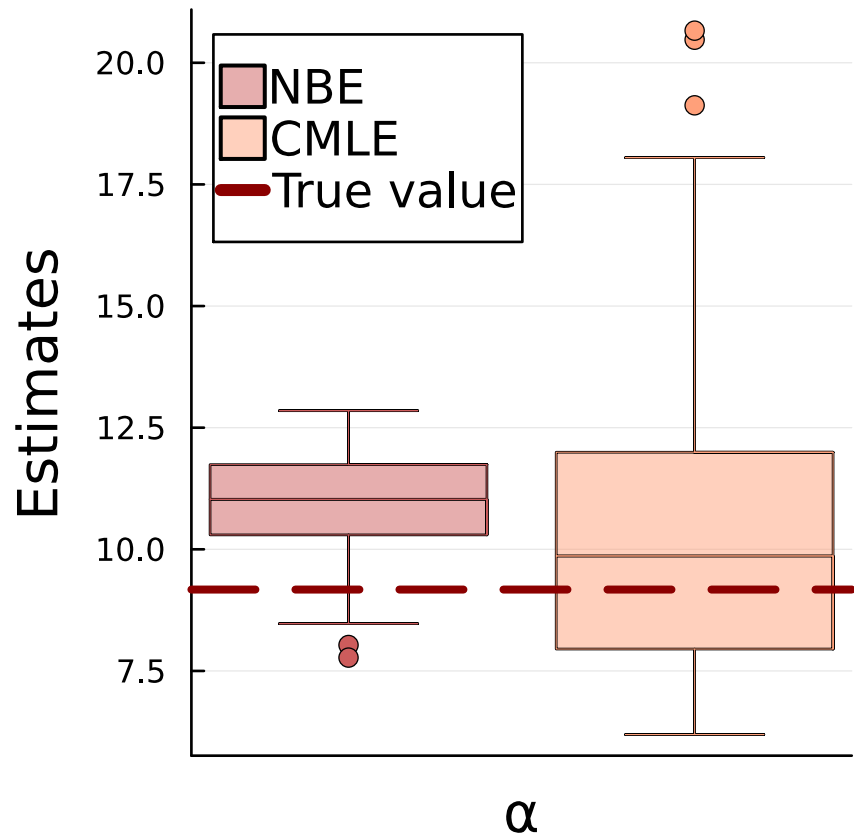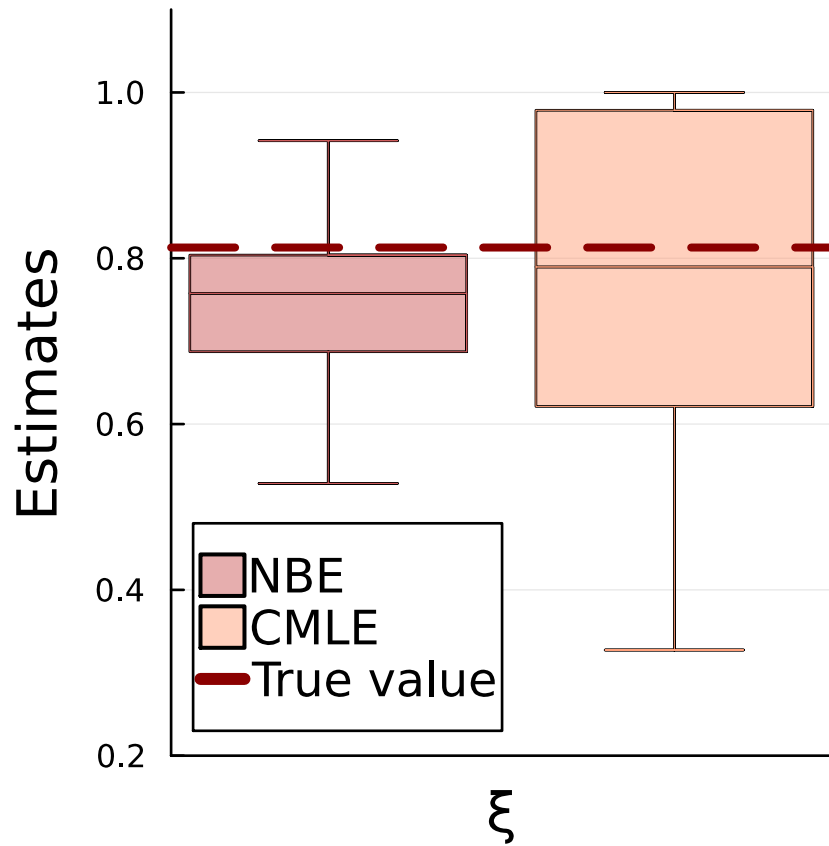

Supplement: Supplementary file 1 — (zip 5334 KB) [file 10687_2025_521_MOESM1_ESM.zip › SupplementaryMaterial/Images/box3_wadsfixed.pdf]

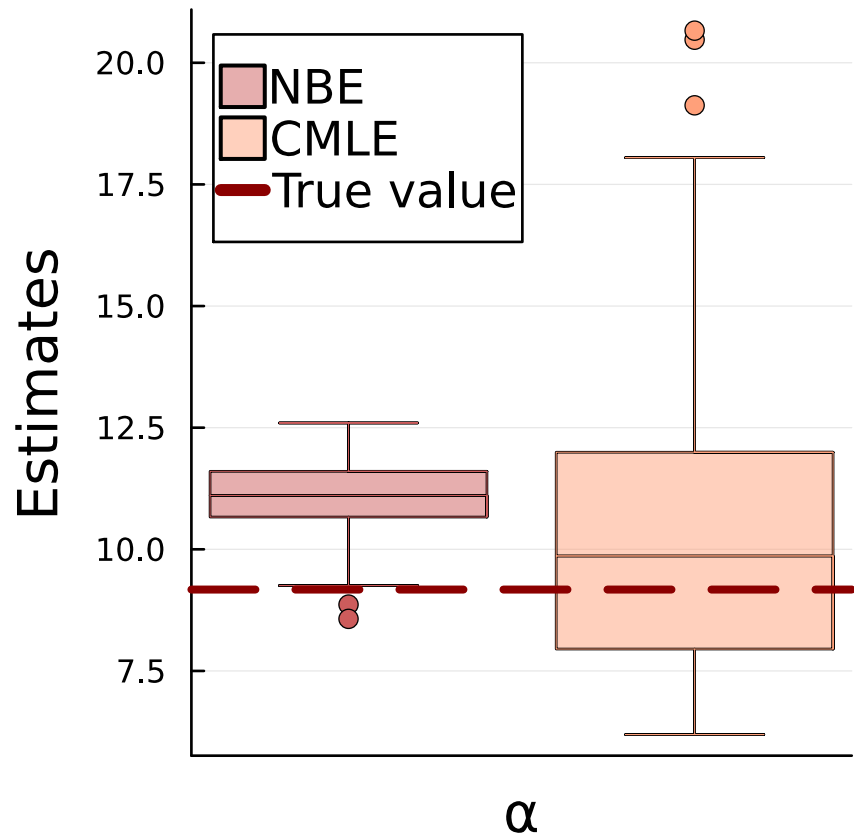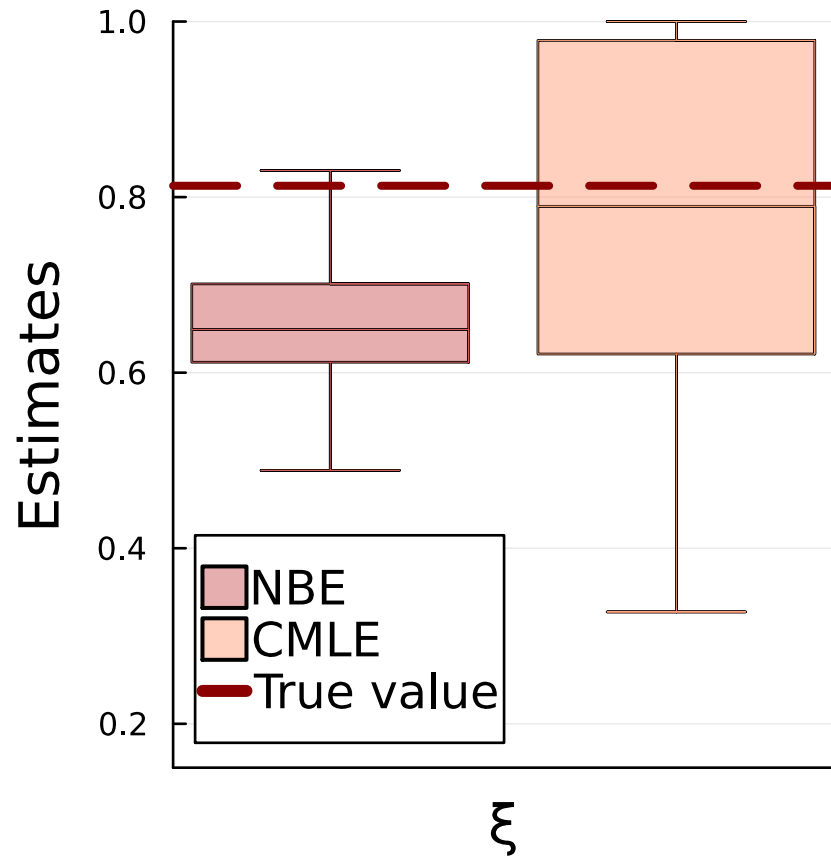

Supplement: Supplementary file 1 — (zip 5334 KB) [file 10687_2025_521_MOESM1_ESM.zip › SupplementaryMaterial/Images/box3_wadsfixedvarn.pdf]

Estimates

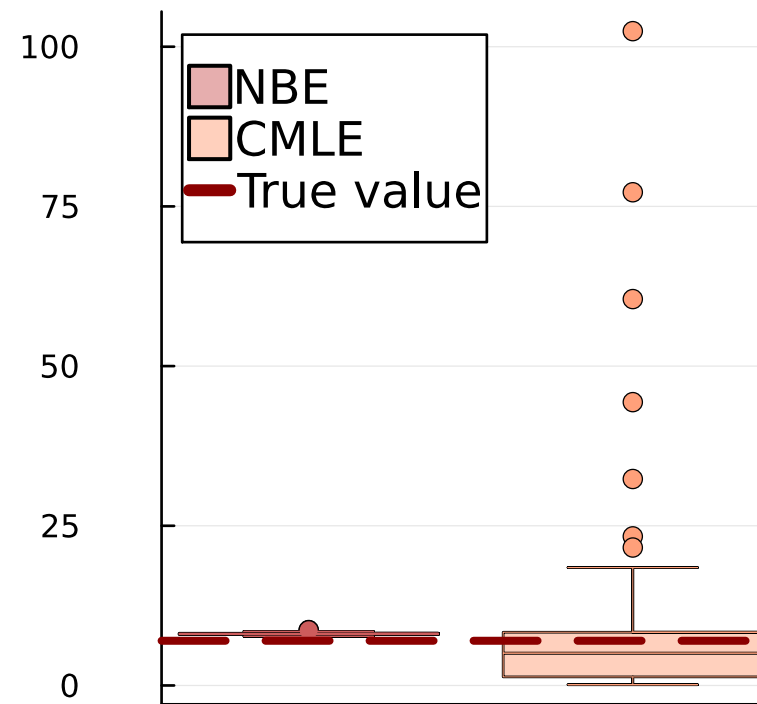

$\alpha$

Estimates

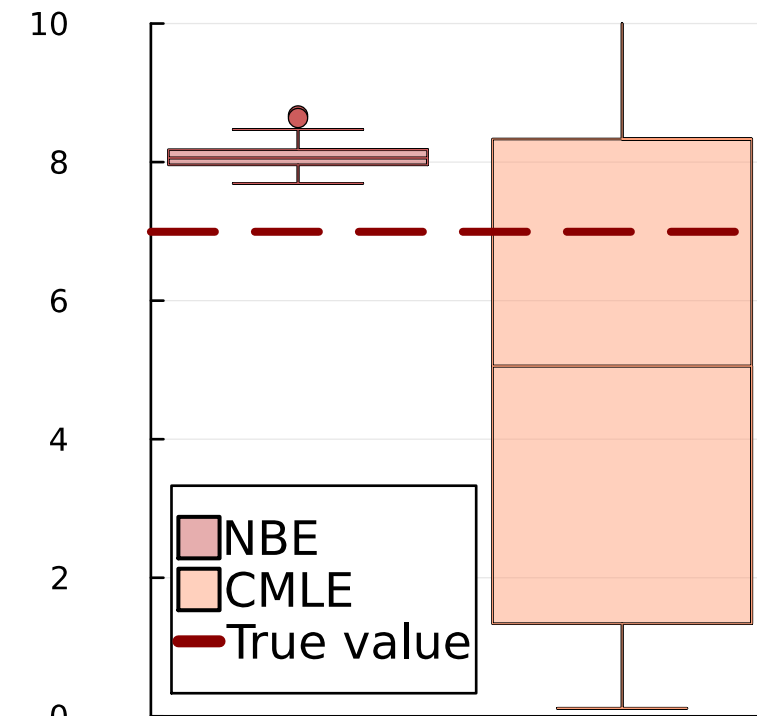

$\alpha$

Estimates

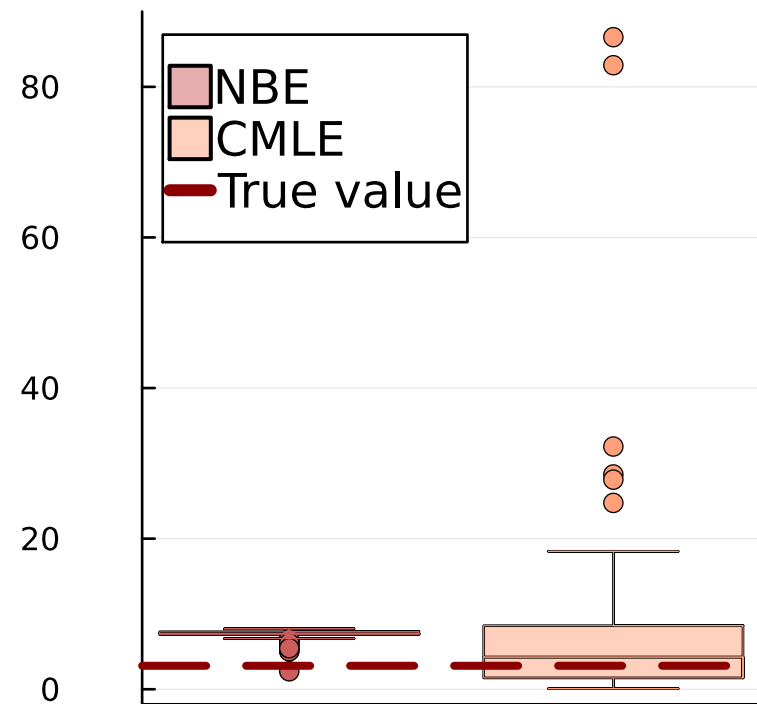

$\beta$

Estimates

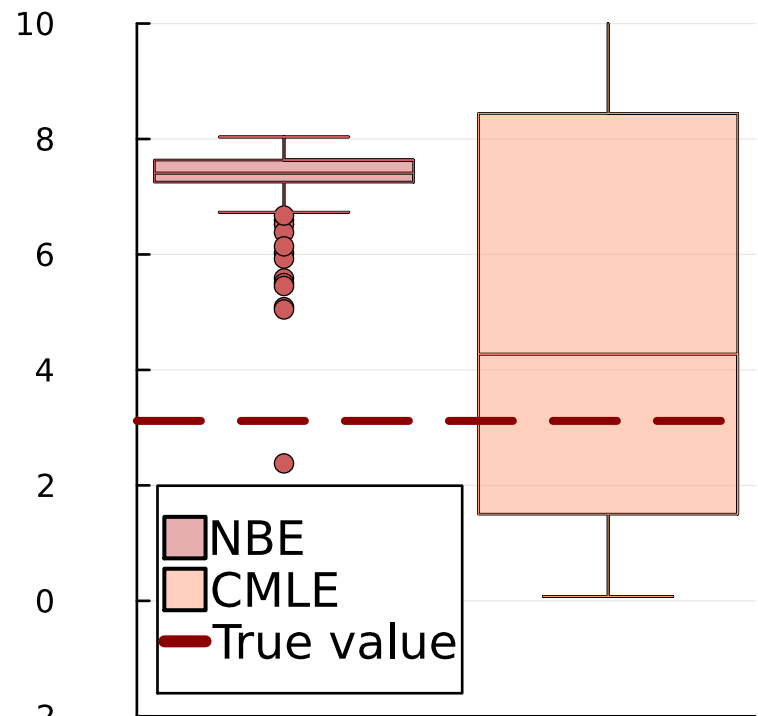

$\beta$

Estimates

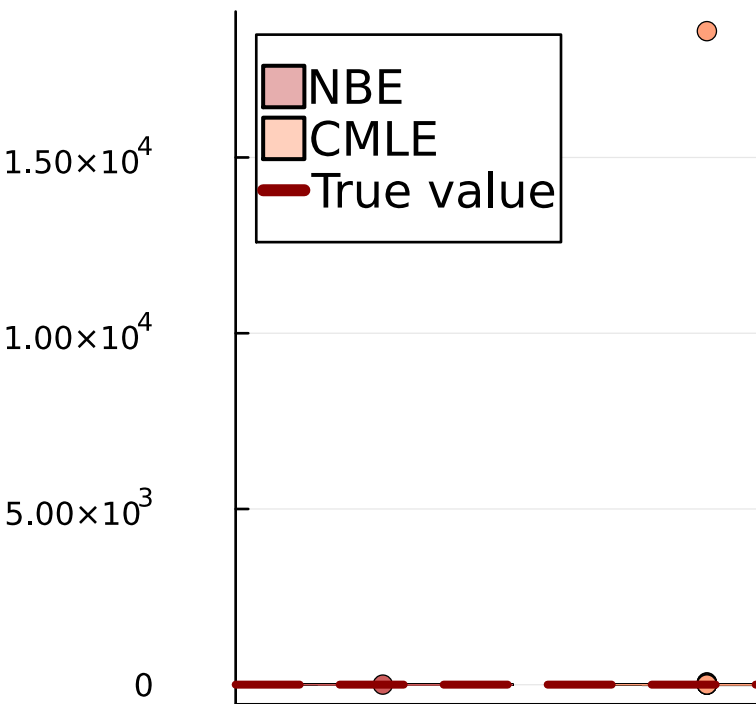

$\mu$

Estimates

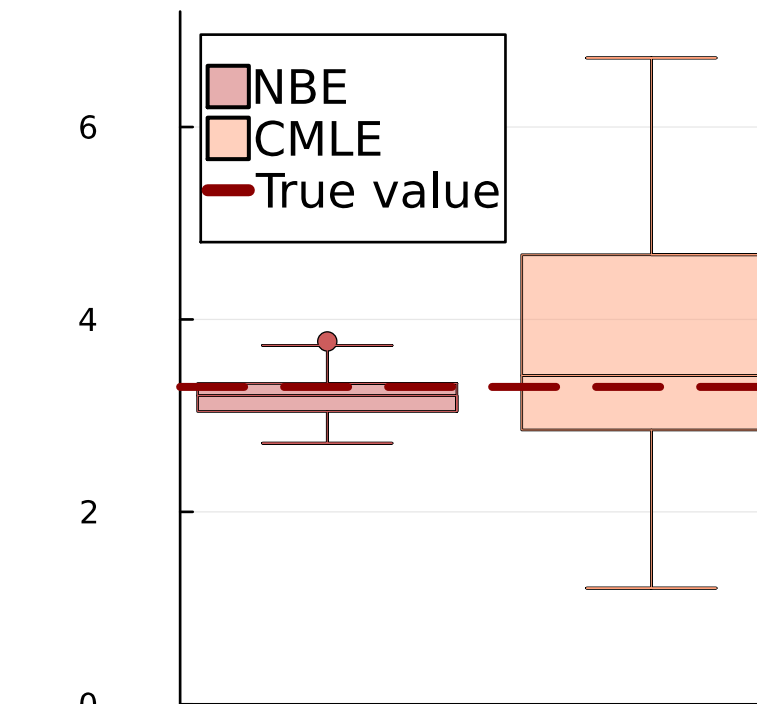

$\mu$

Supplement: Supplementary file 1 — (zip 5334 KB) [file 10687_2025_521_MOESM1_ESM.zip › SupplementaryMaterial/Images/box4_eng1.pdf]

Estimates

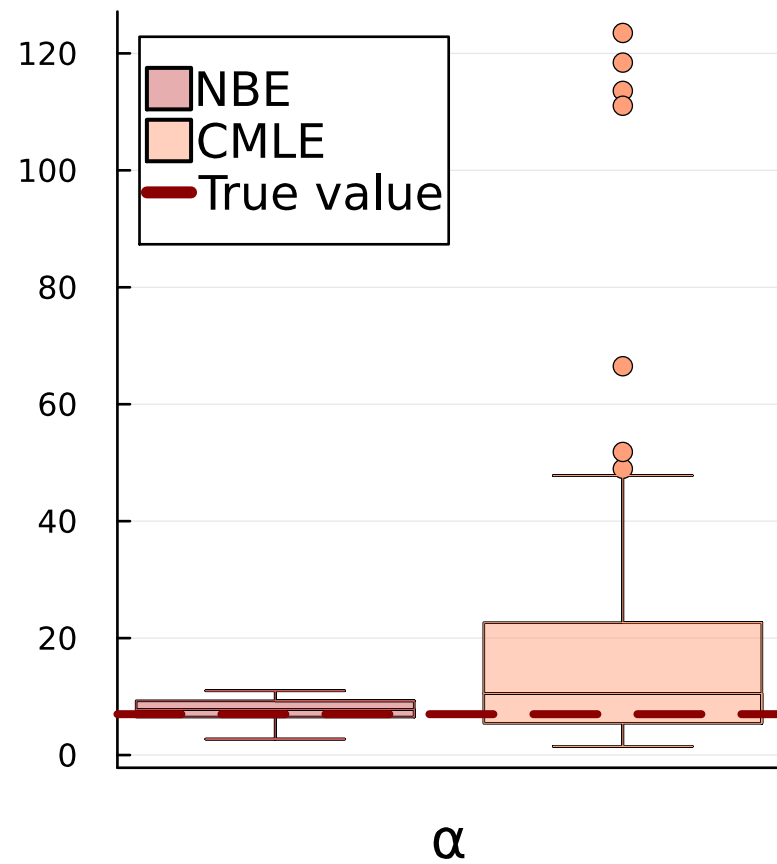

Estimates

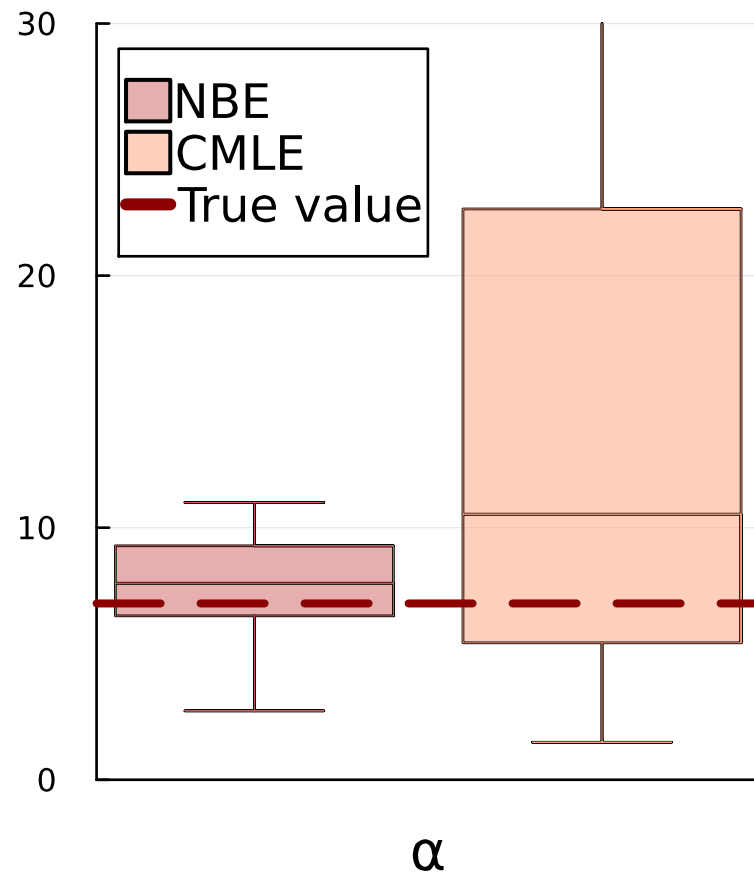

Estimates

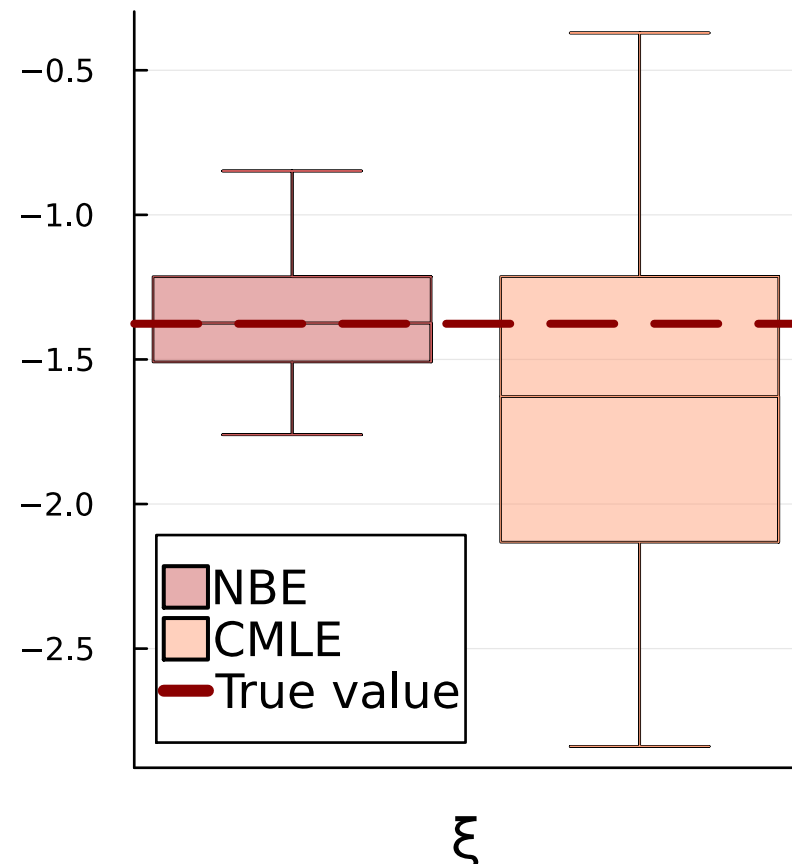

Supplement: Supplementary file 1 — (zip 5334 KB) [file 10687_2025_521_MOESM1_ESM.zip › SupplementaryMaterial/Images/box4_eng2.pdf]

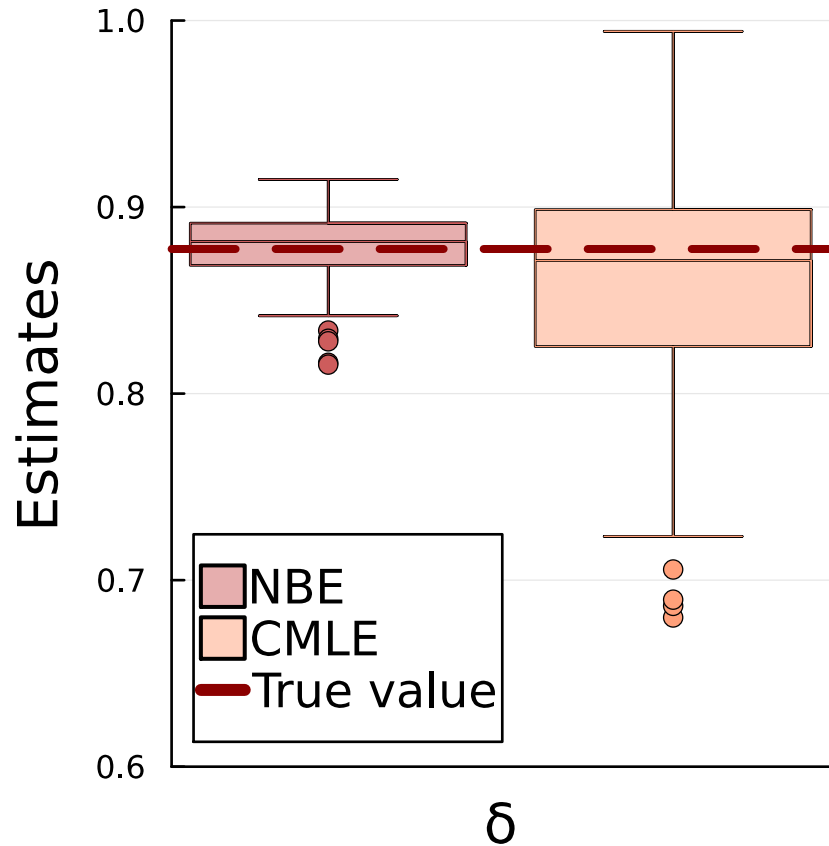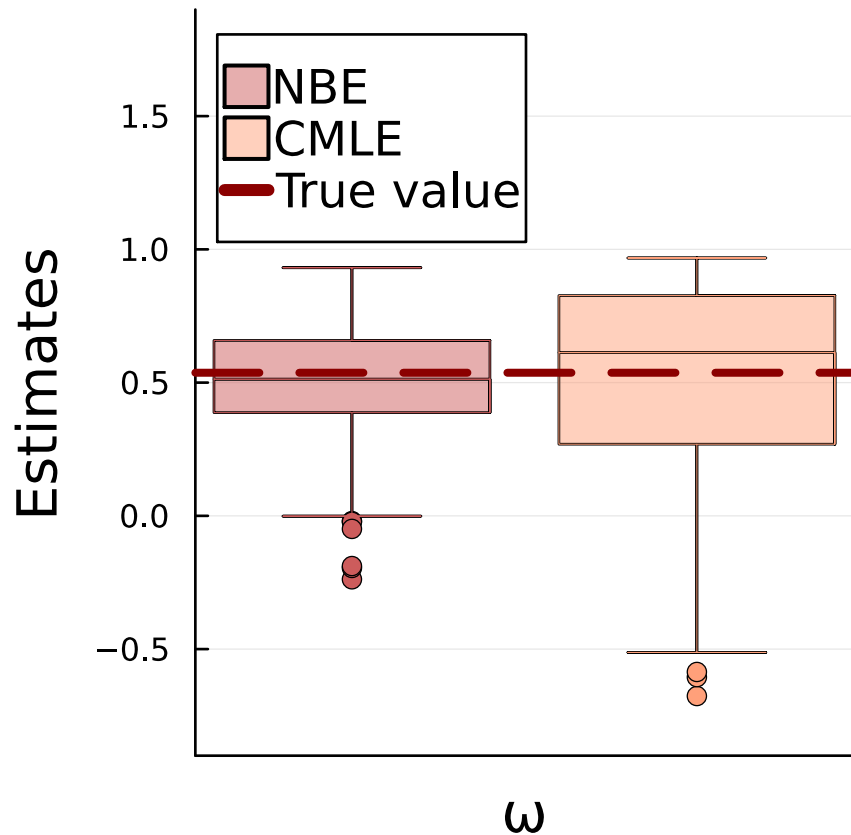

Supplement: Supplementary file 1 — (zip 5334 KB) [file 10687_2025_521_MOESM1_ESM.zip › SupplementaryMaterial/Images/box4_hwGauss.pdf]

Estimates

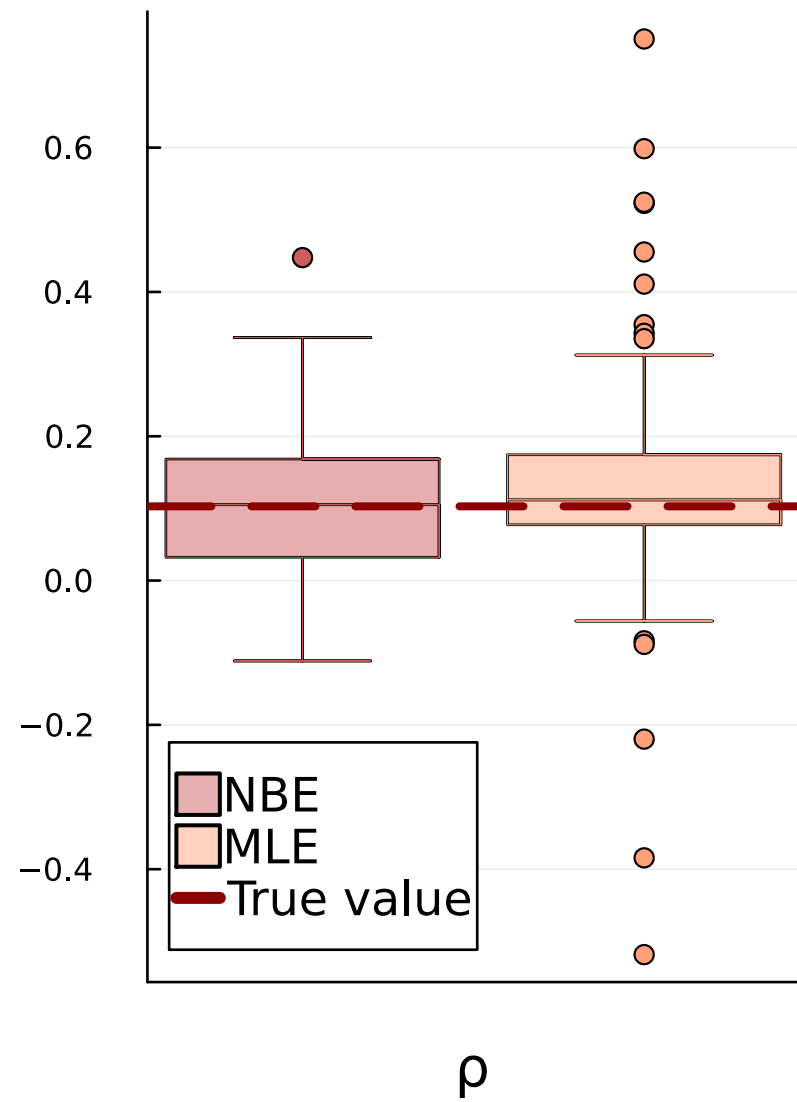

Estimates

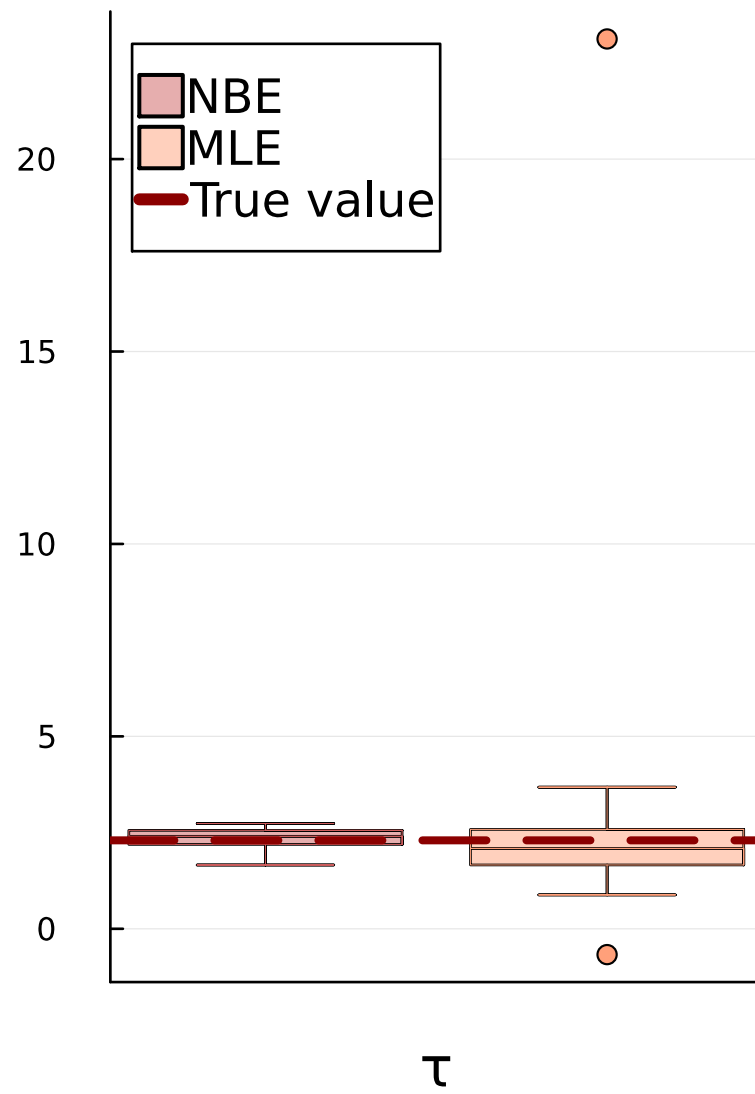

Estimates

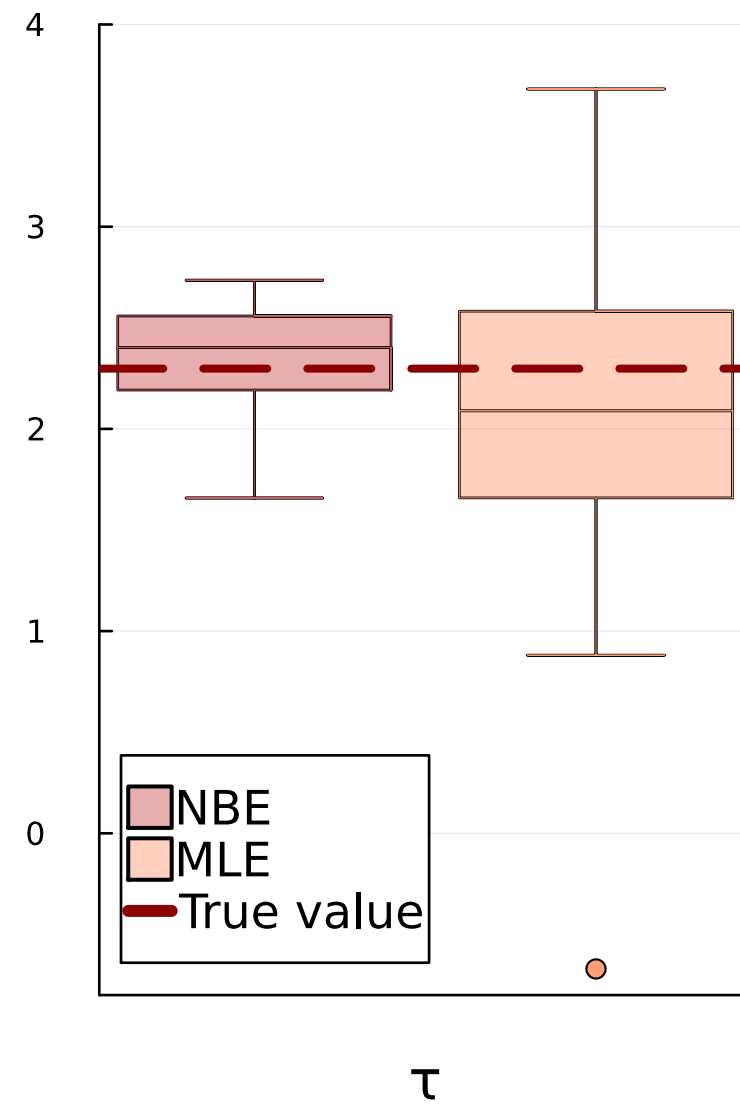

Estimates

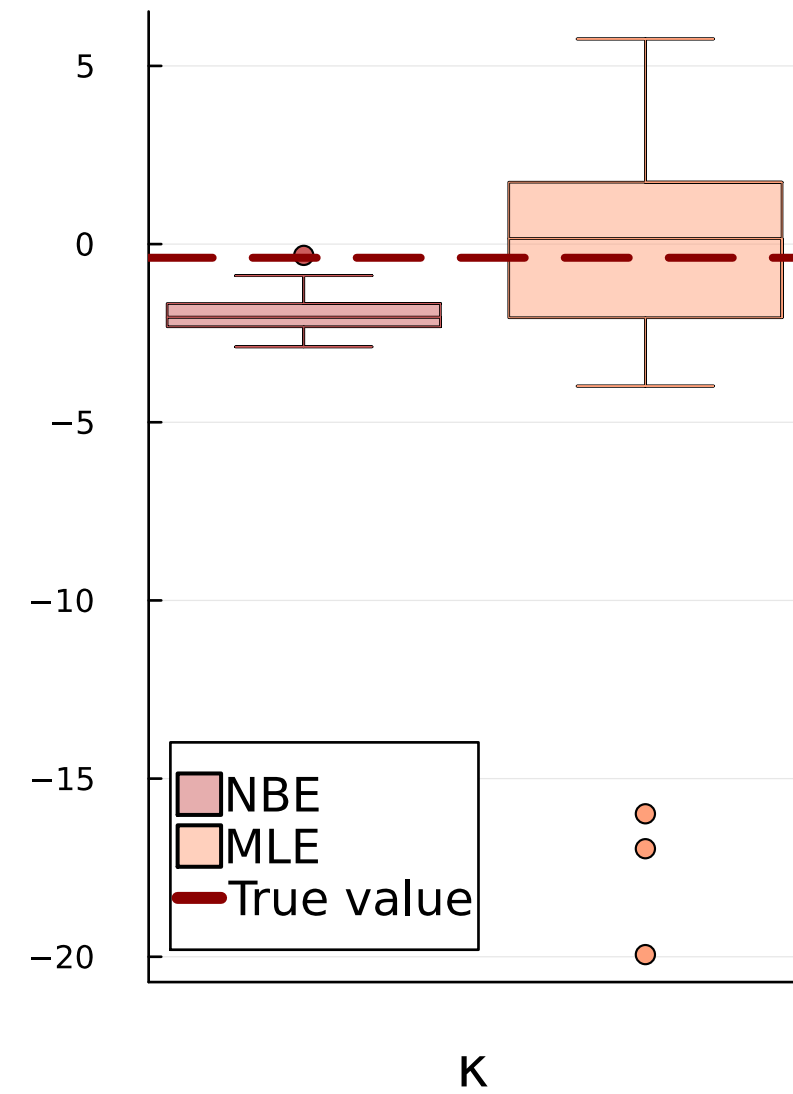

Supplement: Supplementary file 1 — (zip 5334 KB) [file 10687_2025_521_MOESM1_ESM.zip › SupplementaryMaterial/Images/box4_mod1.pdf]

Estimates

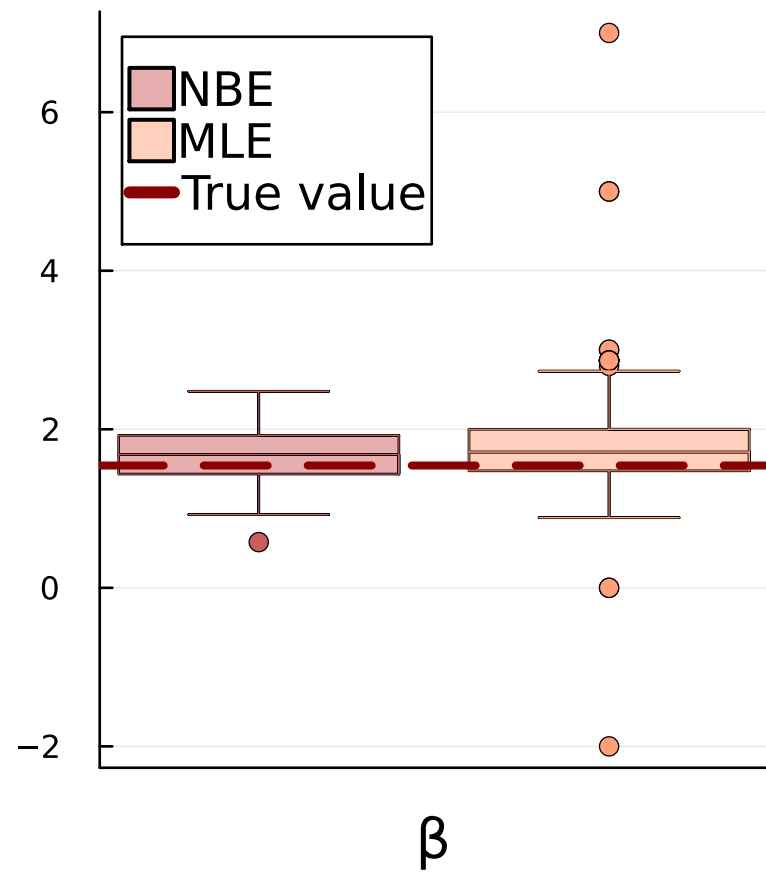

Estimates

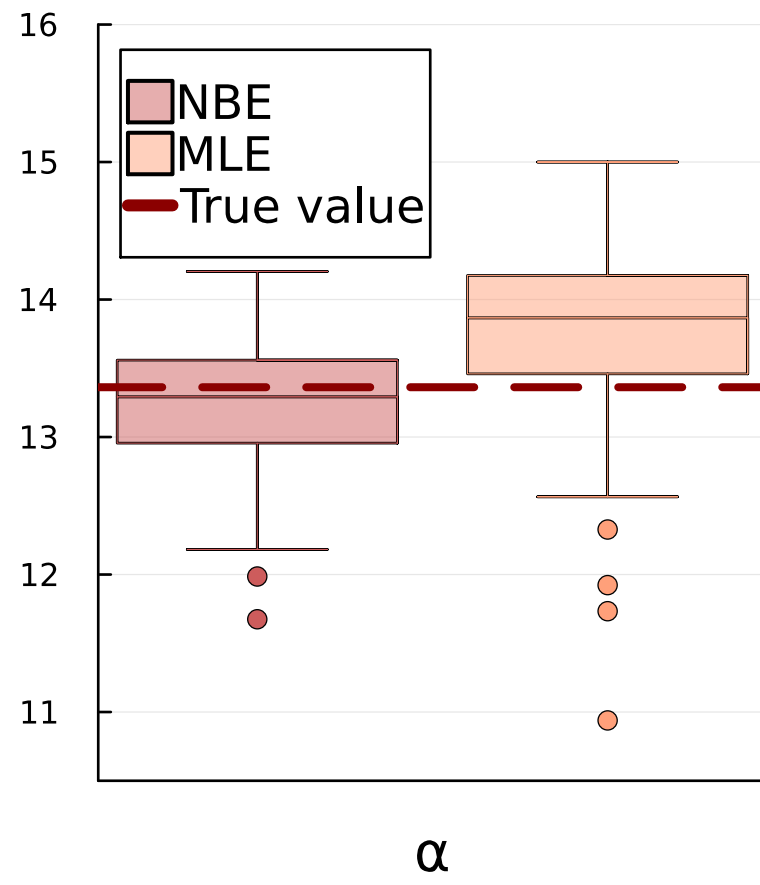

Estimates

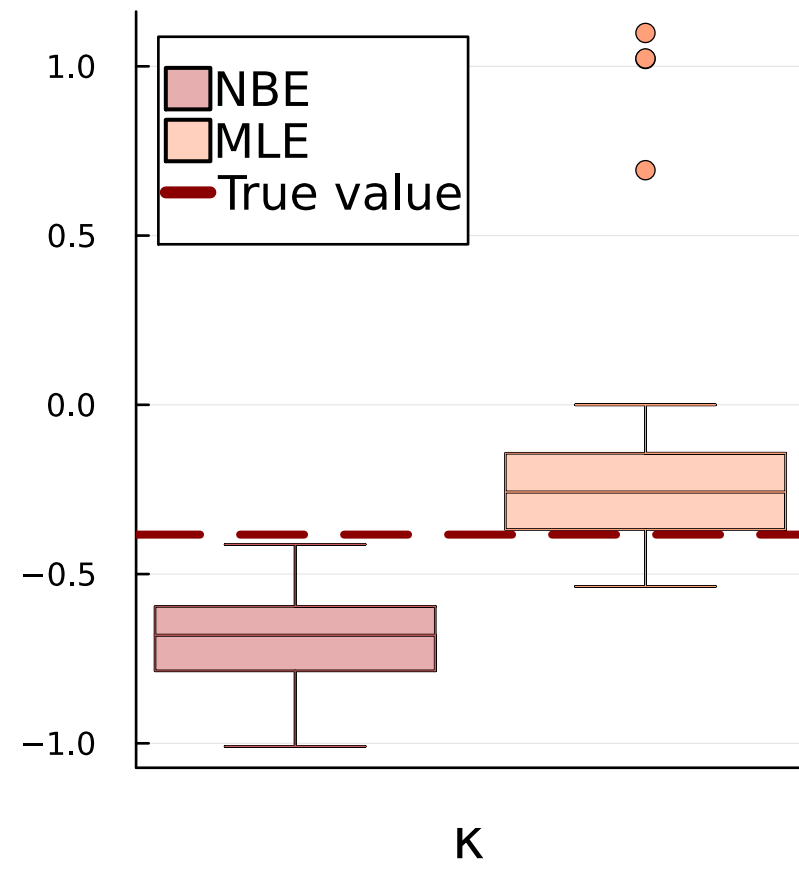

Supplement: Supplementary file 1 — (zip 5334 KB) [file 10687_2025_521_MOESM1_ESM.zip › SupplementaryMaterial/Images/box4_mod2.pdf]

Estimates

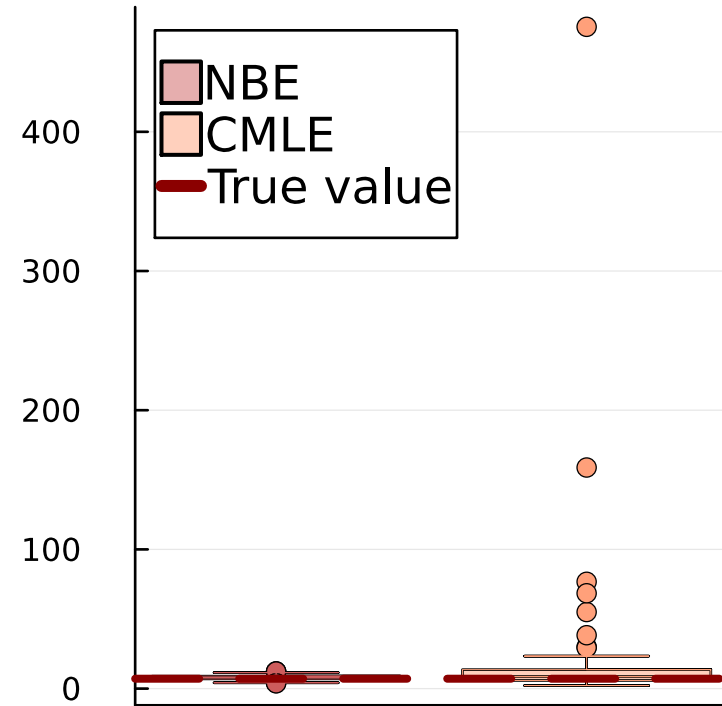 $\alpha$ 

Estimates

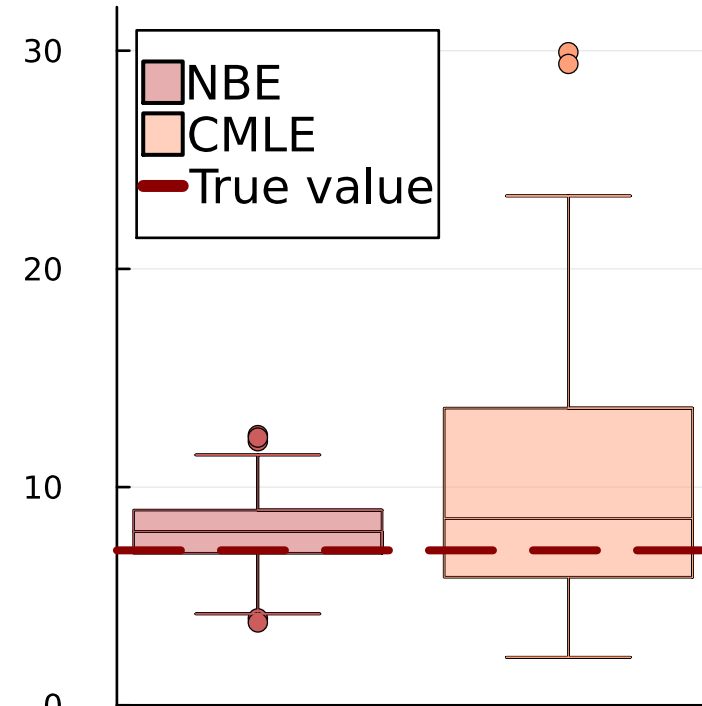 $\alpha$ 

Estimates

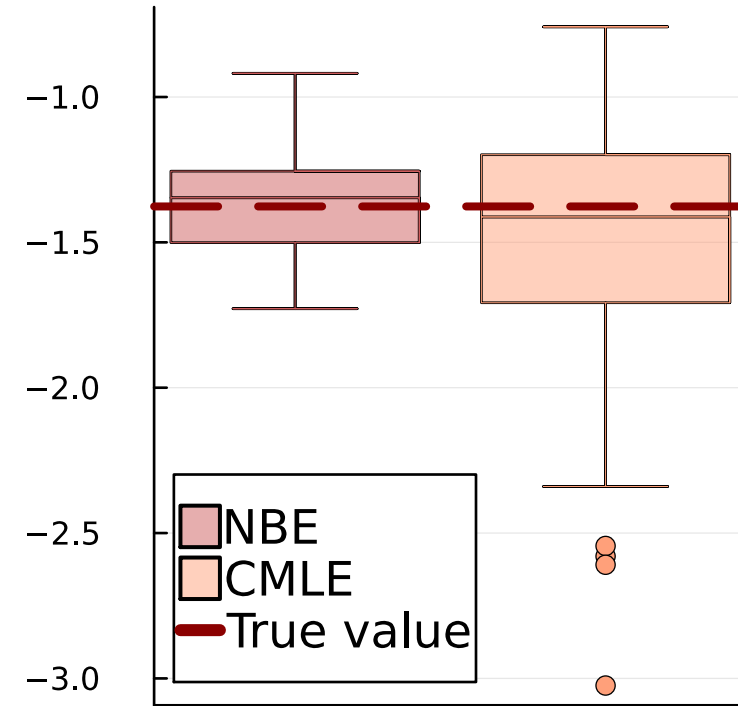 $\xi$ 

Estimates

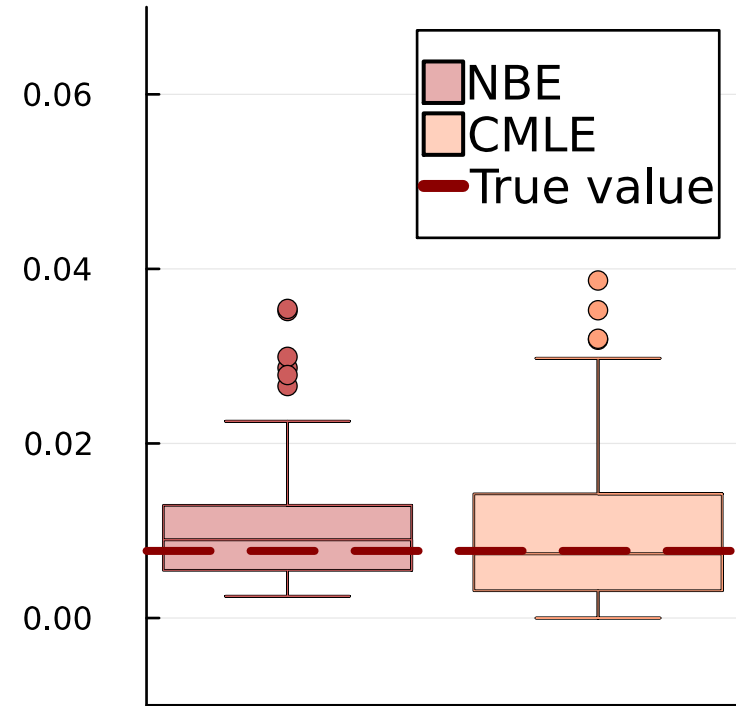 $\chi(0.99)$

Supplement: Supplementary file 1 — (zip 5334 KB) [file 10687_2025_521_MOESM1_ESM.zip › SupplementaryMaterial/Images/box4_wads.pdf]

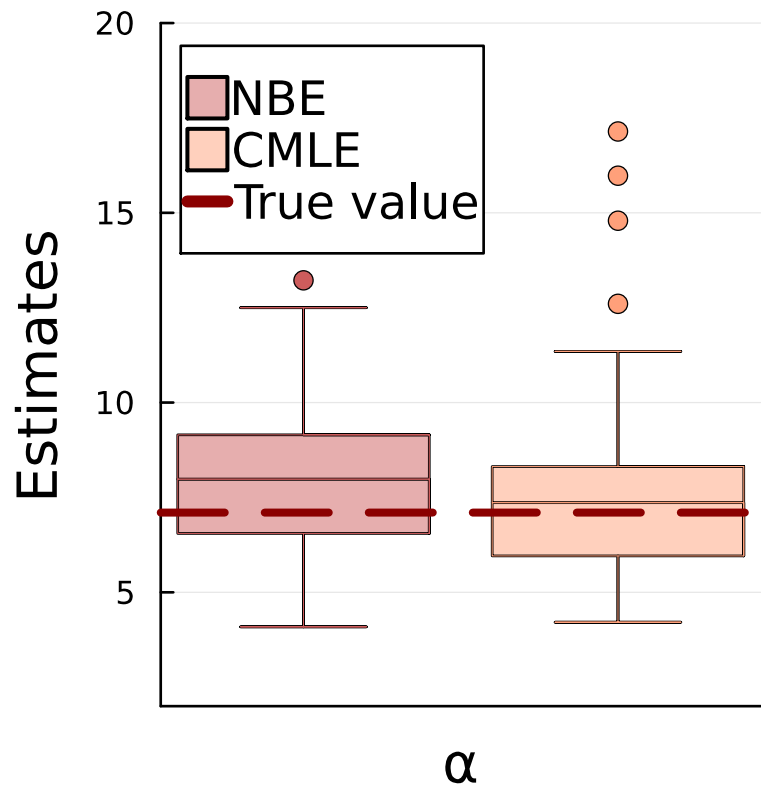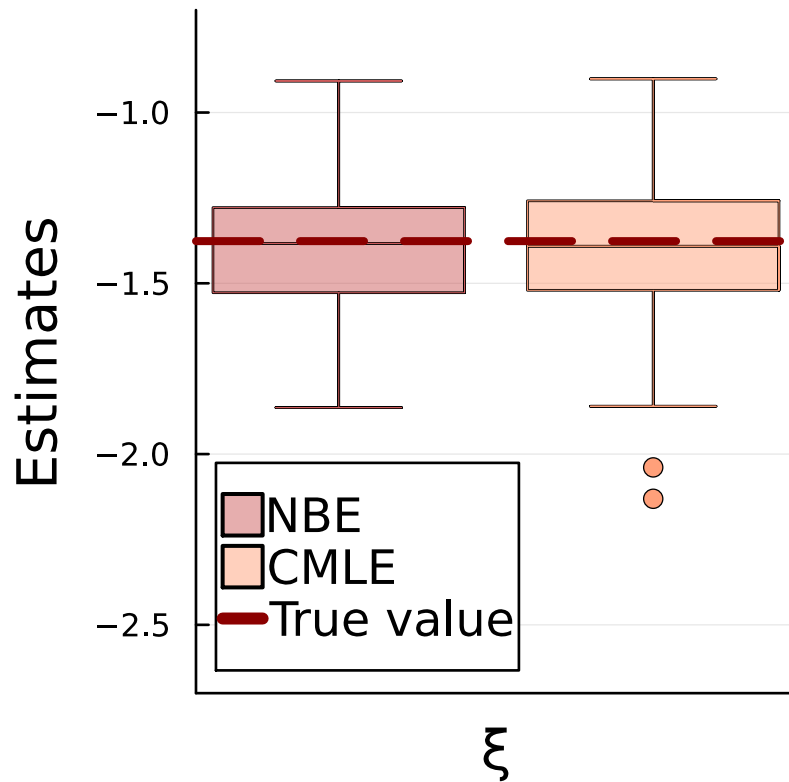

Supplement: Supplementary file 1 — (zip 5334 KB) [file 10687_2025_521_MOESM1_ESM.zip › SupplementaryMaterial/Images/box4_wadsfixed.pdf]

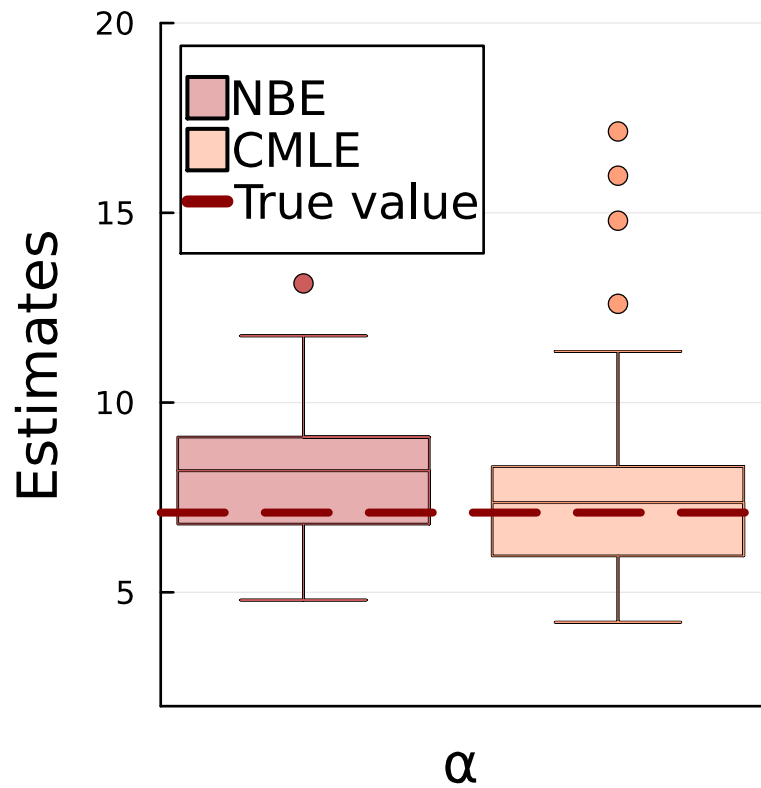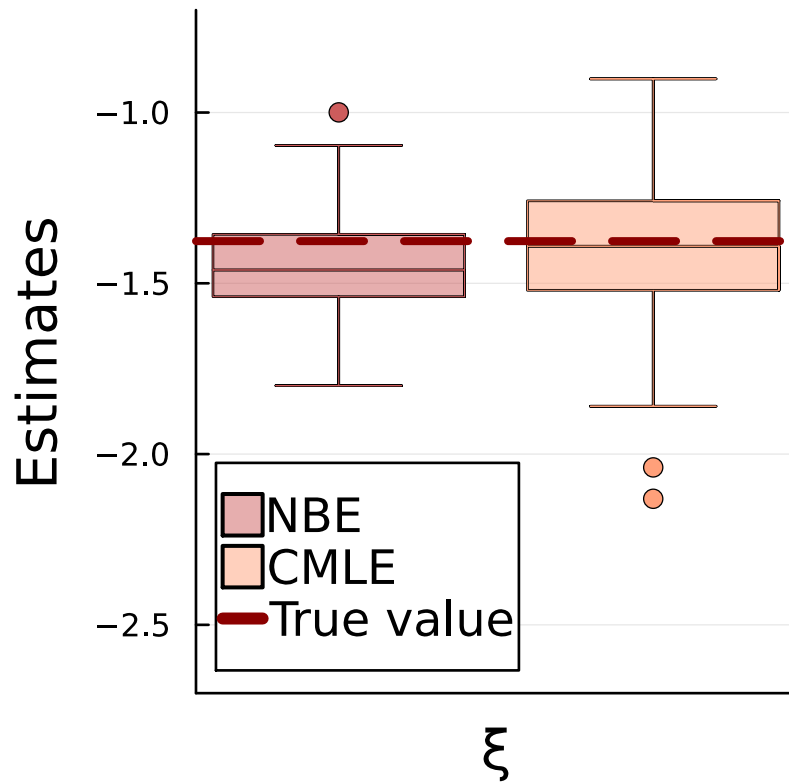

Supplement: Supplementary file 1 — (zip 5334 KB) [file 10687_2025_521_MOESM1_ESM.zip › SupplementaryMaterial/Images/box4_wadsfixedvarn.pdf]

Estimates

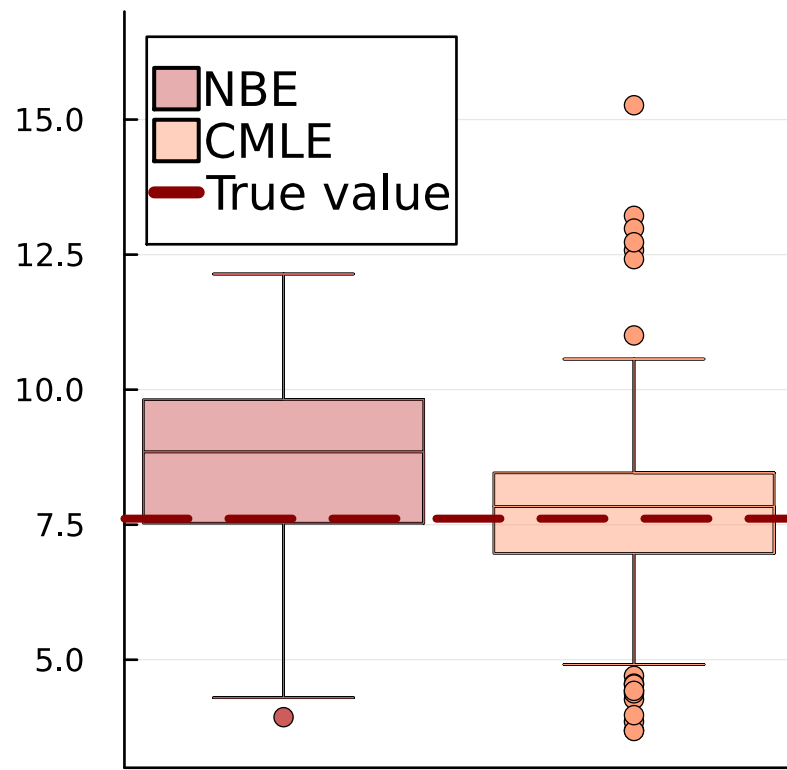 $\alpha$ 

Estimates

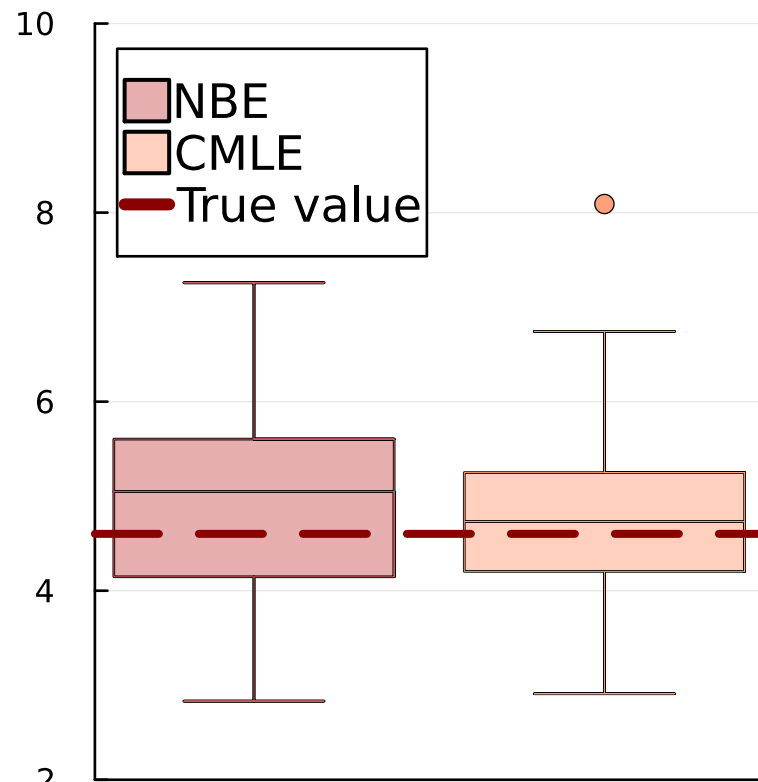 $\beta$ 

Estimates

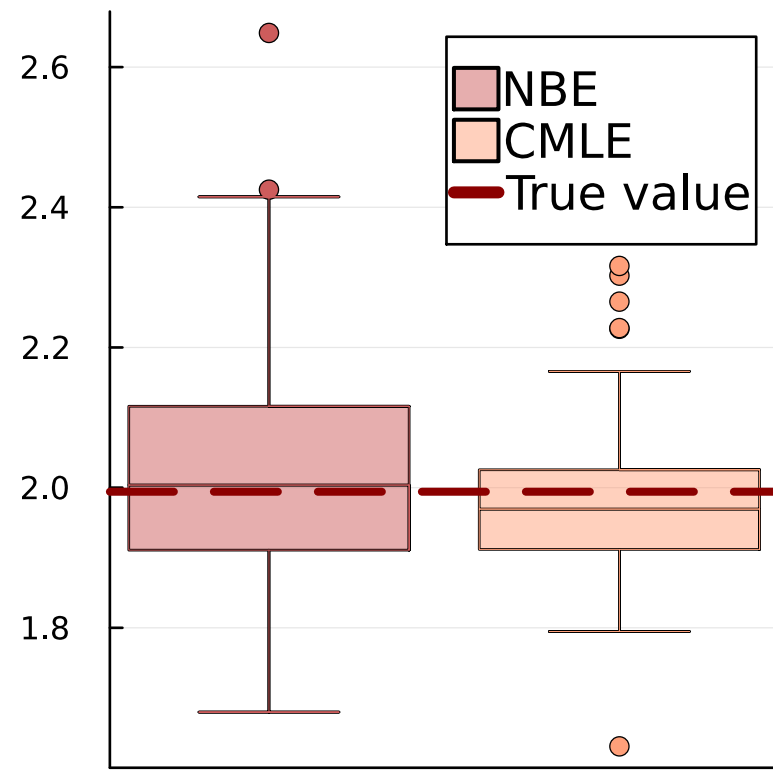 $\mu$

Supplement: Supplementary file 1 — (zip 5334 KB) [file 10687_2025_521_MOESM1_ESM.zip › SupplementaryMaterial/Images/box5_eng1.pdf]

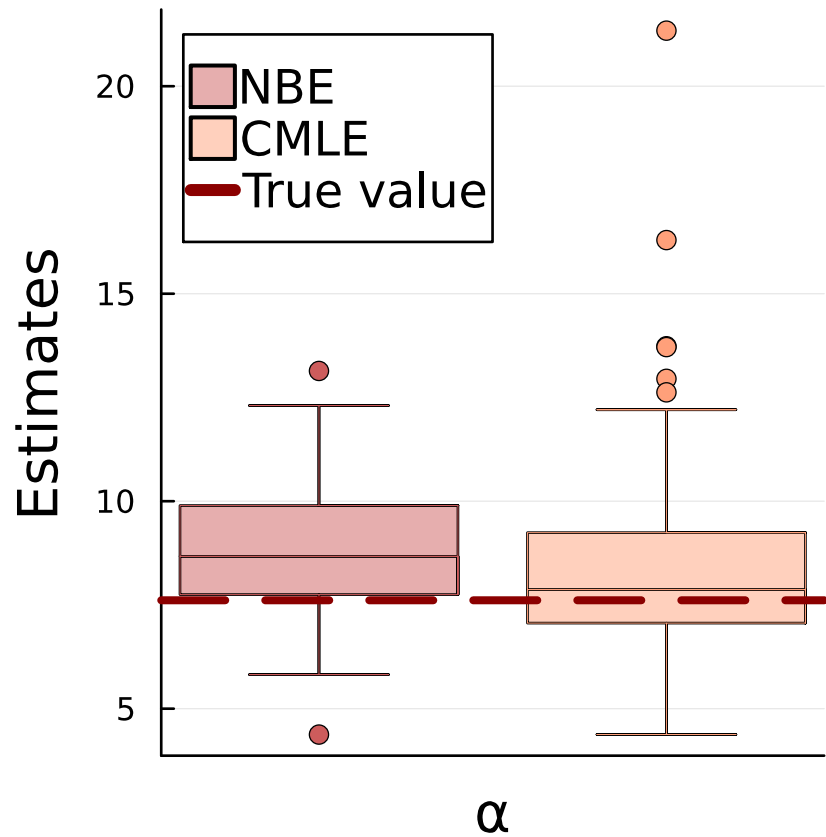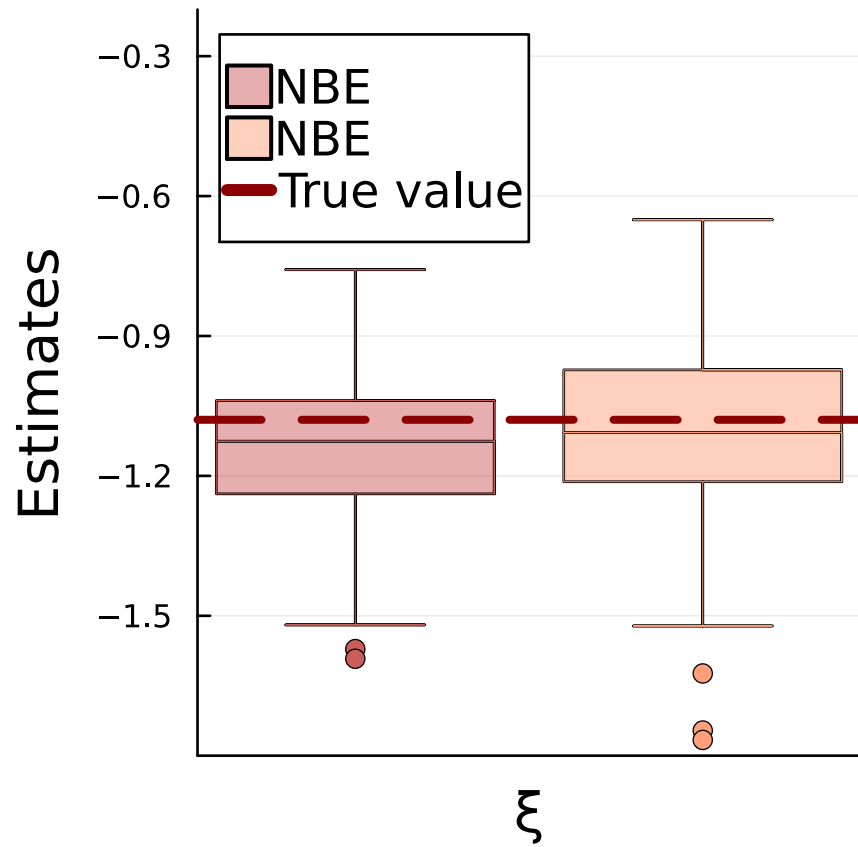

Supplement: Supplementary file 1 — (zip 5334 KB) [file 10687_2025_521_MOESM1_ESM.zip › SupplementaryMaterial/Images/box5_eng2.pdf]

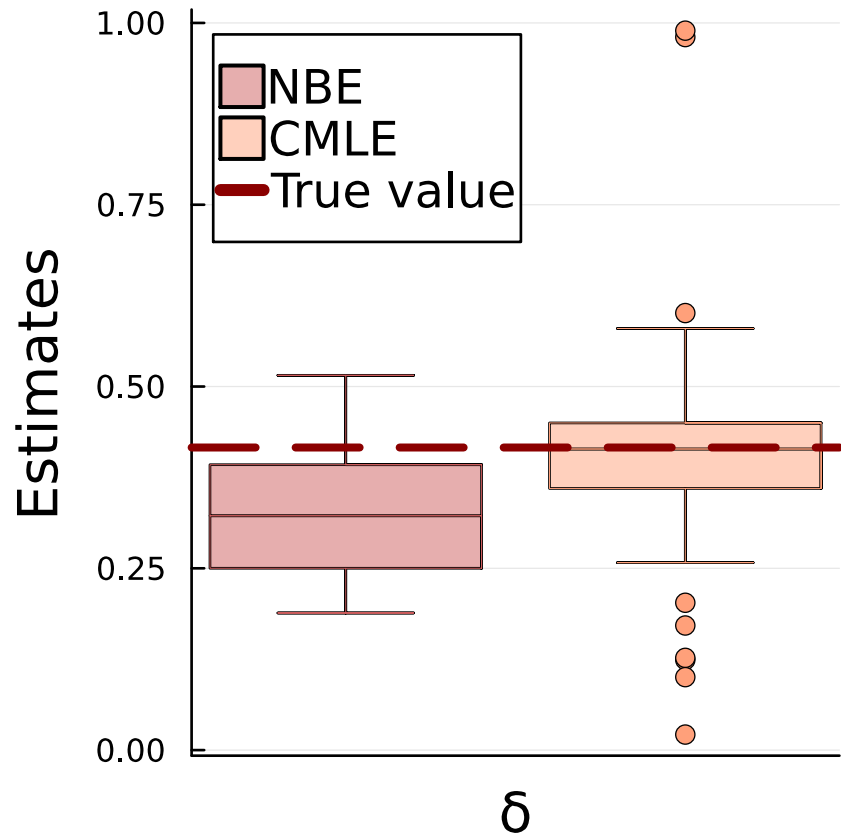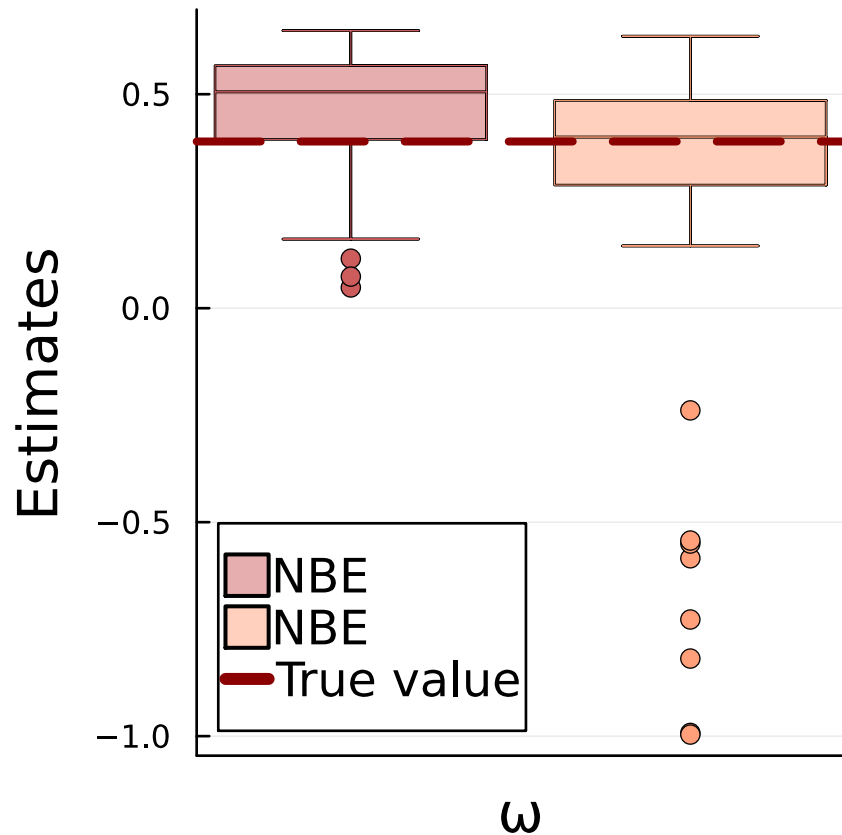

Supplement: Supplementary file 1 — (zip 5334 KB) [file 10687_2025_521_MOESM1_ESM.zip › SupplementaryMaterial/Images/box5_hwGauss.pdf]

Estimates

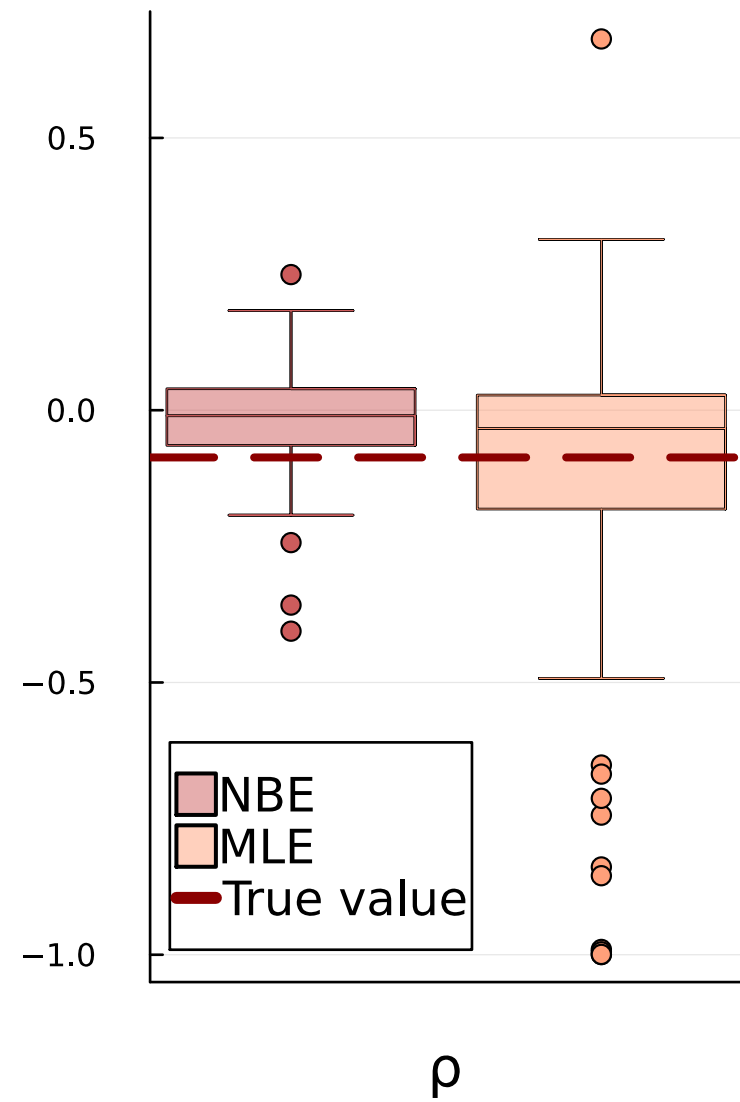

Estimates

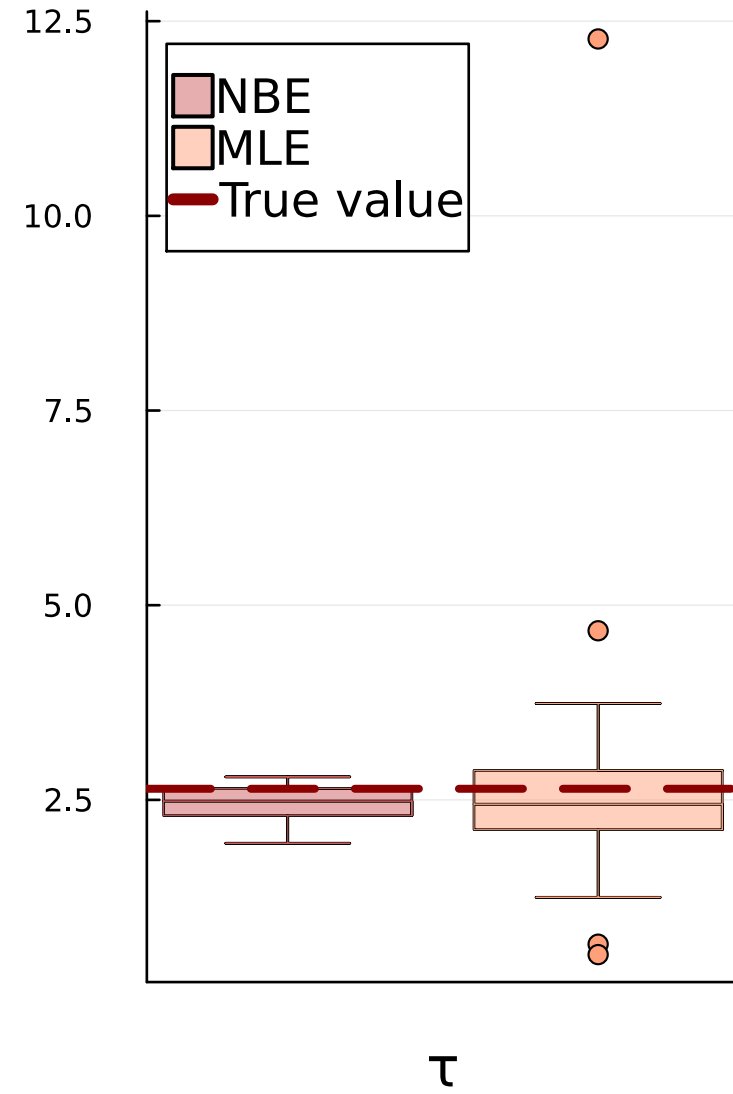

Estimates

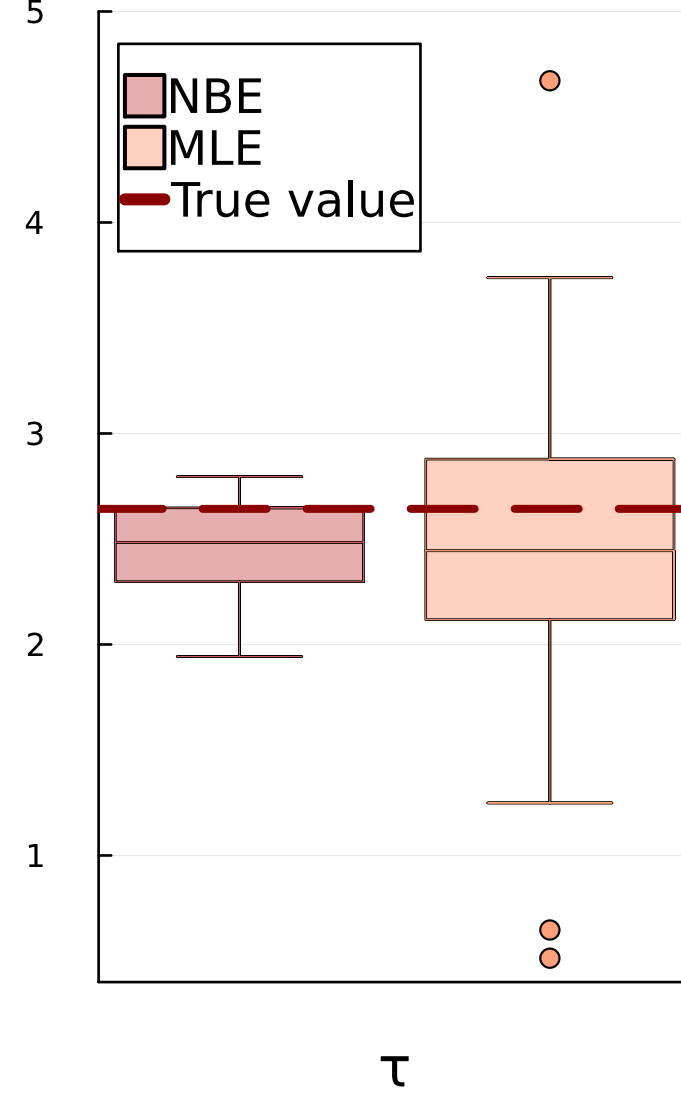

Estimates

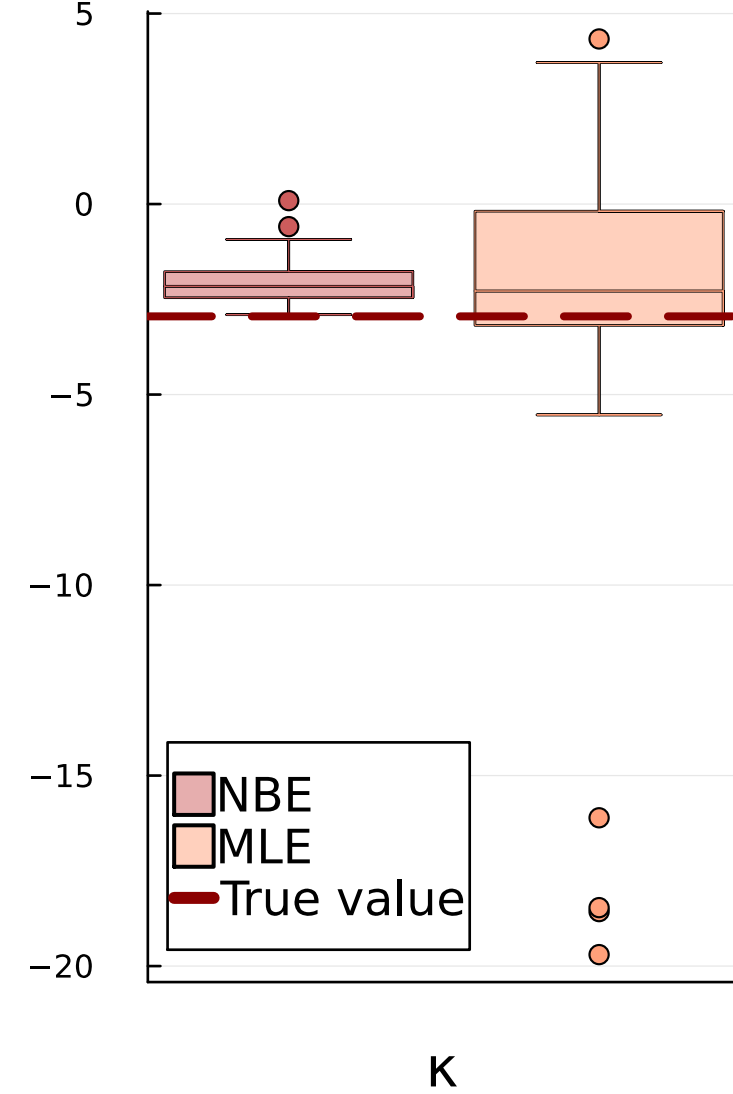

Supplement: Supplementary file 1 — (zip 5334 KB) [file 10687_2025_521_MOESM1_ESM.zip › SupplementaryMaterial/Images/box5_mod1.pdf]

Estimates

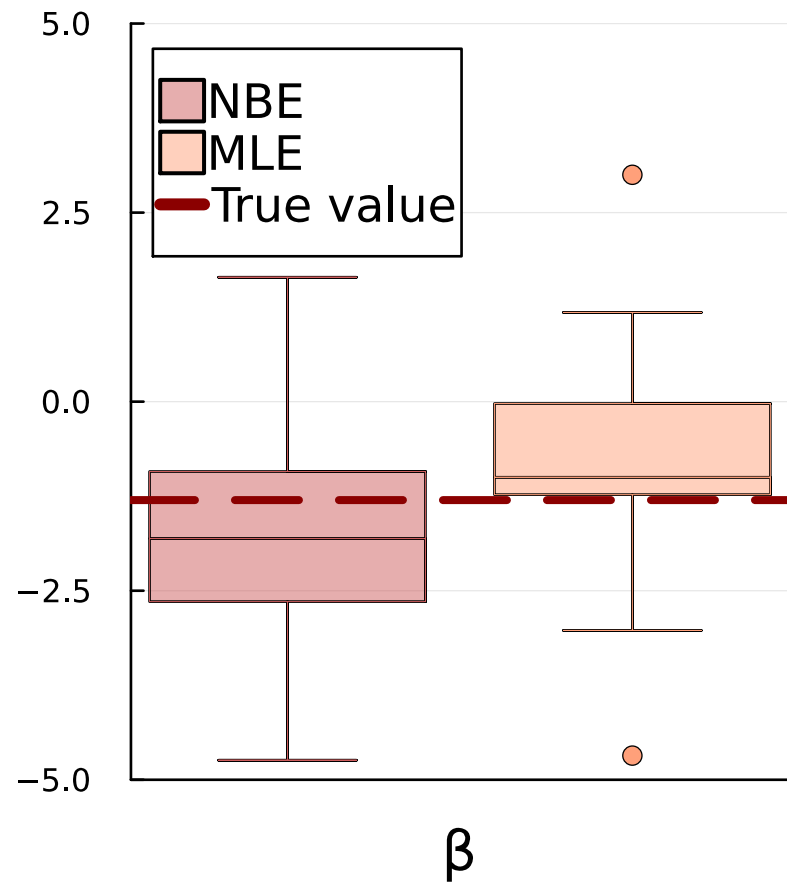

Estimates

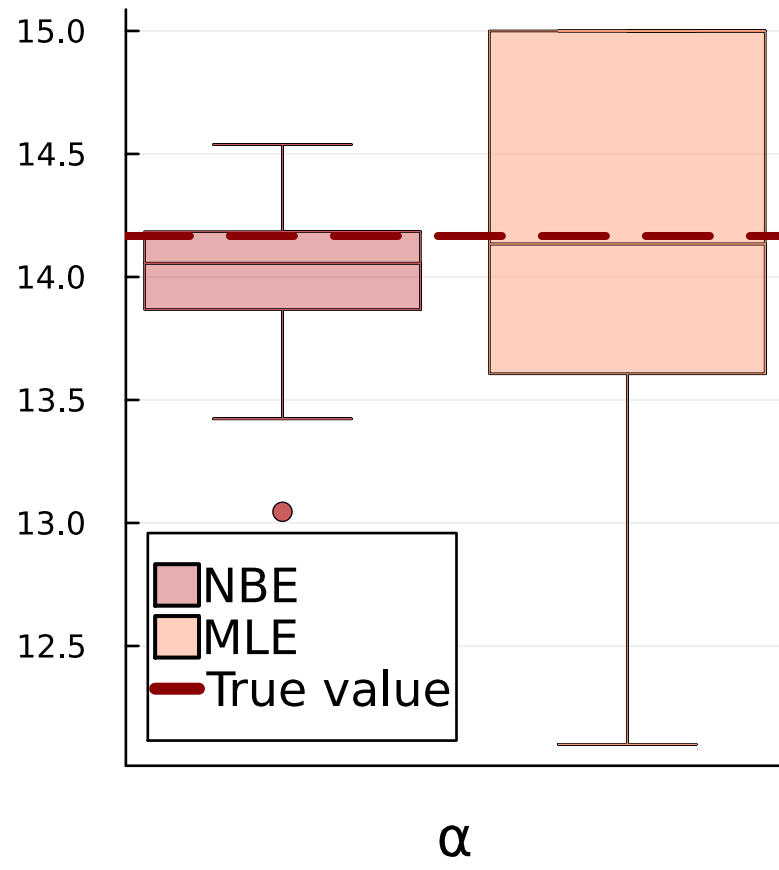

Estimates

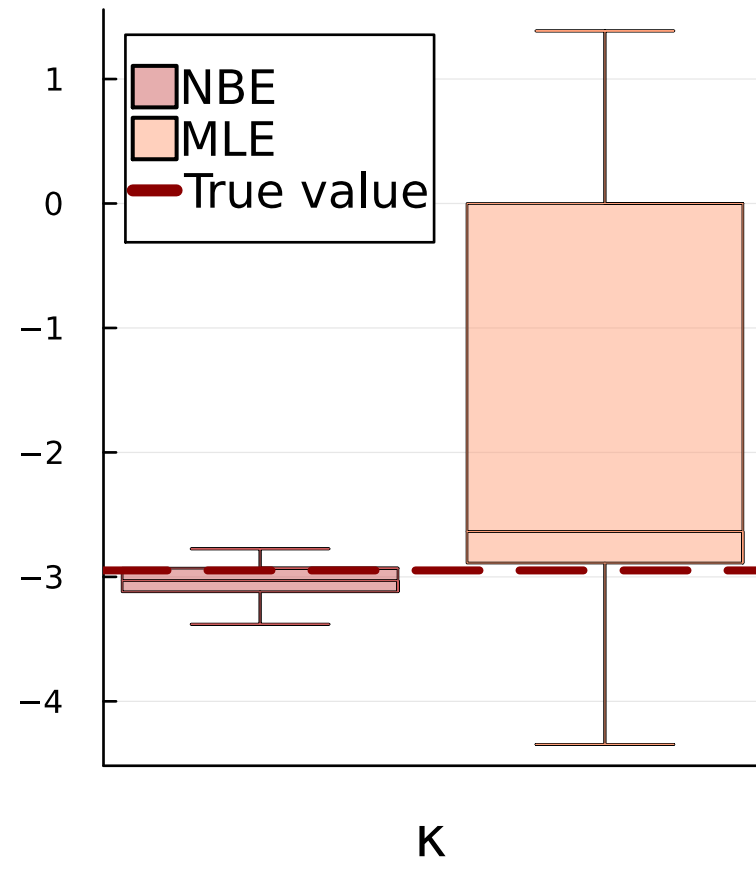

Supplement: Supplementary file 1 — (zip 5334 KB) [file 10687_2025_521_MOESM1_ESM.zip › SupplementaryMaterial/Images/box5_mod2.pdf]

Estimates

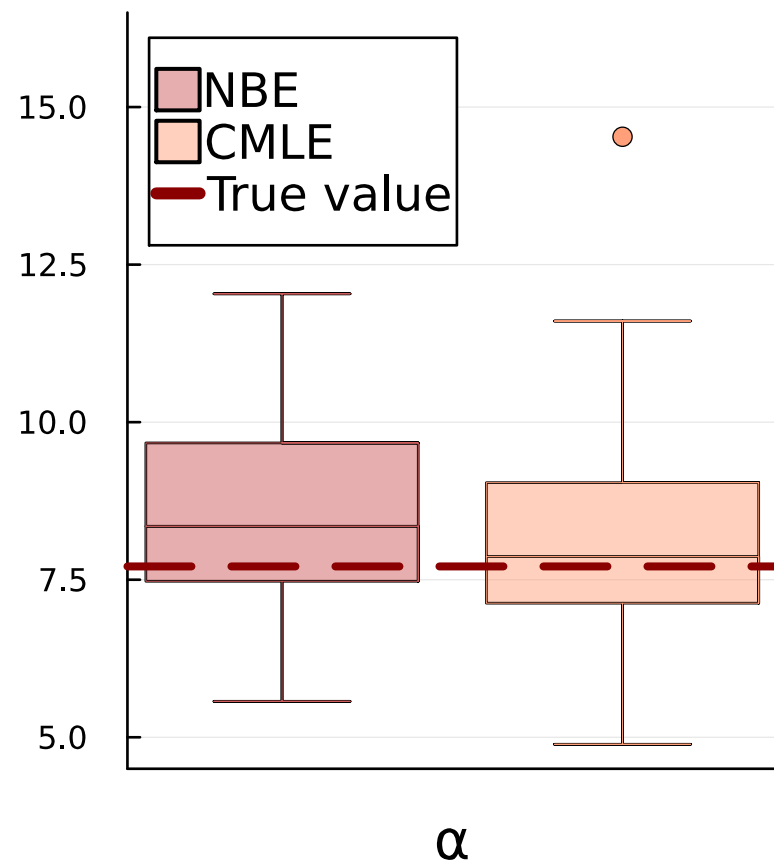

Estimates

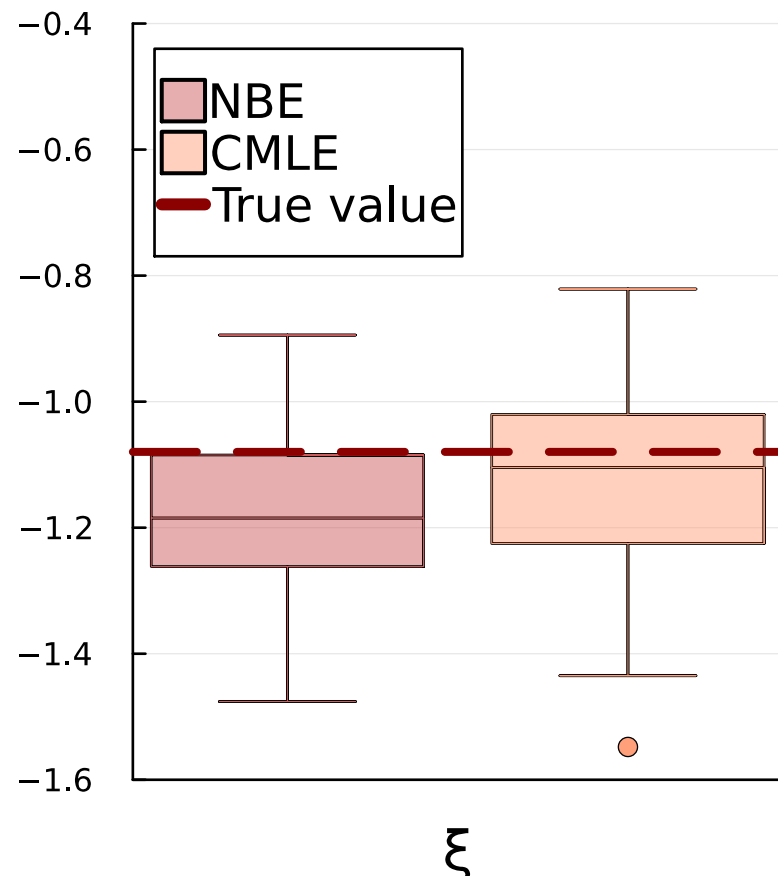

Estimates

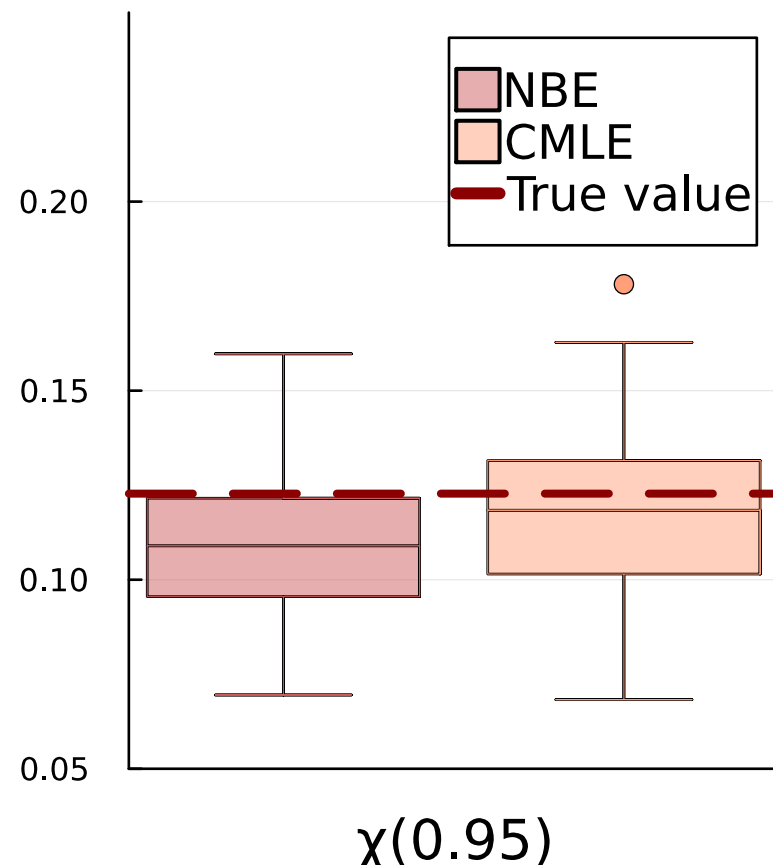

Supplement: Supplementary file 1 — (zip 5334 KB) [file 10687_2025_521_MOESM1_ESM.zip › SupplementaryMaterial/Images/box5_wads.pdf]

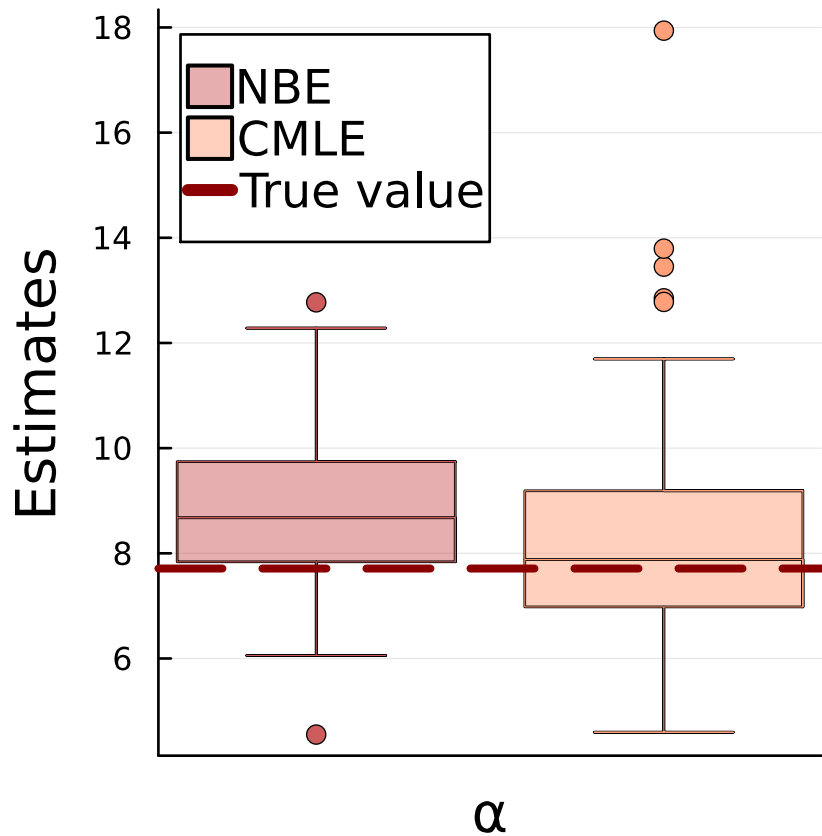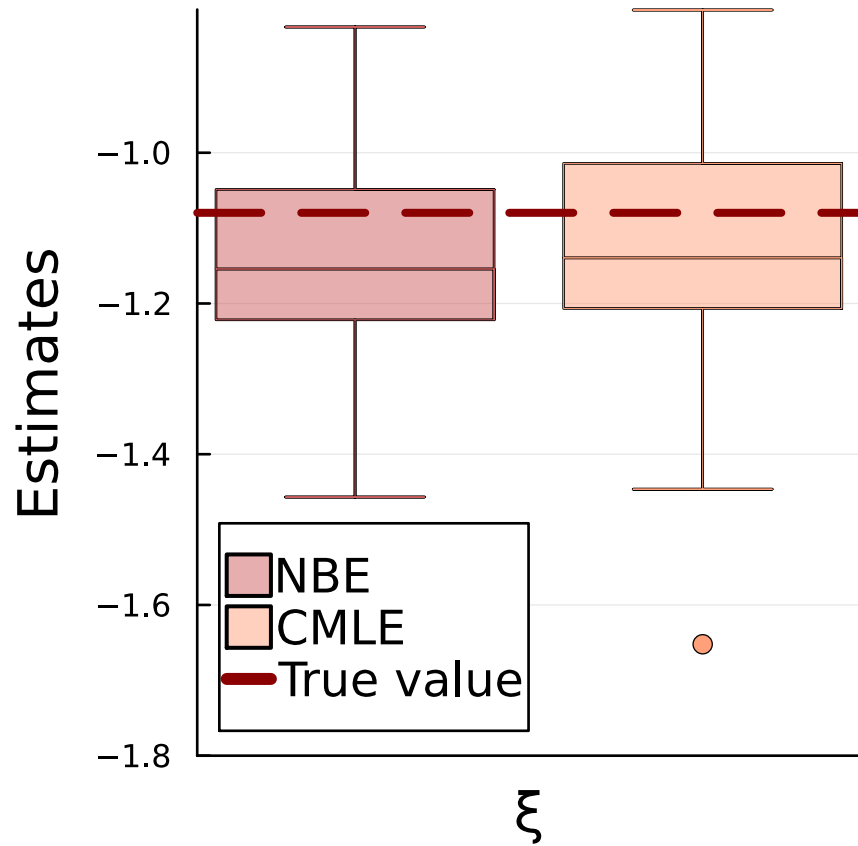

Supplement: Supplementary file 1 — (zip 5334 KB) [file 10687_2025_521_MOESM1_ESM.zip › SupplementaryMaterial/Images/box5_wadsfixed.pdf]

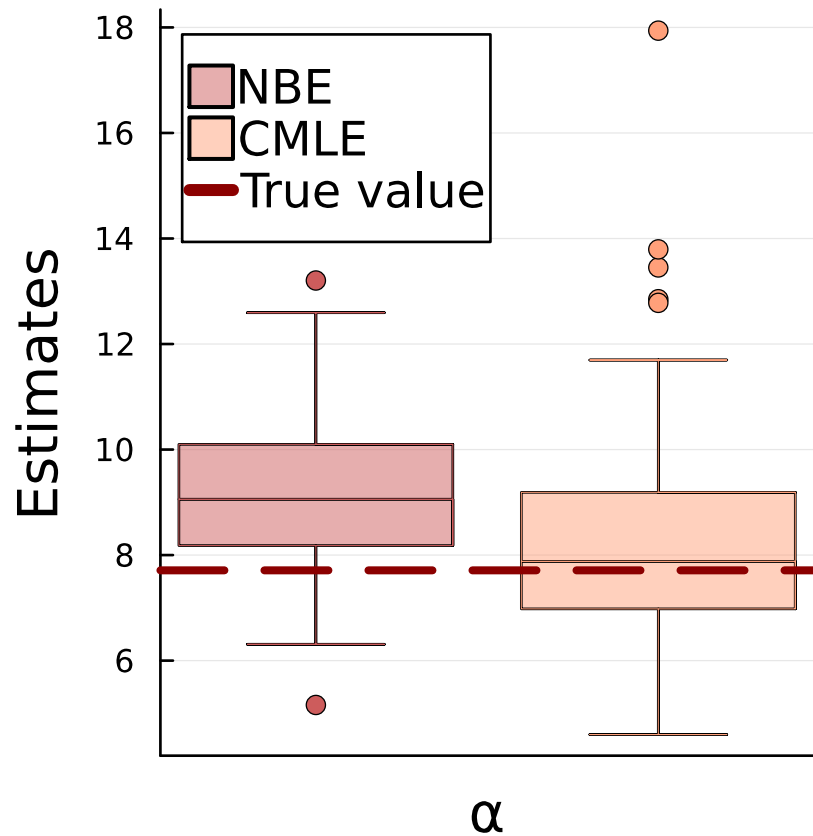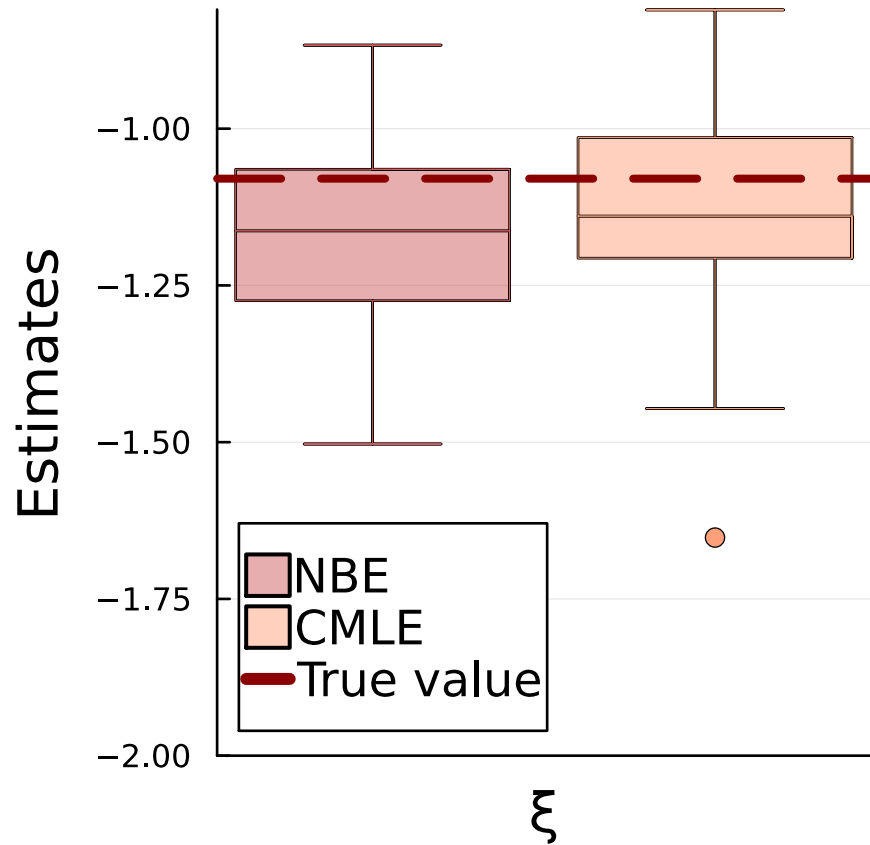

Supplement: Supplementary file 1 — (zip 5334 KB) [file 10687_2025_521_MOESM1_ESM.zip › SupplementaryMaterial/Images/box5_wadsfixedvarn.pdf]

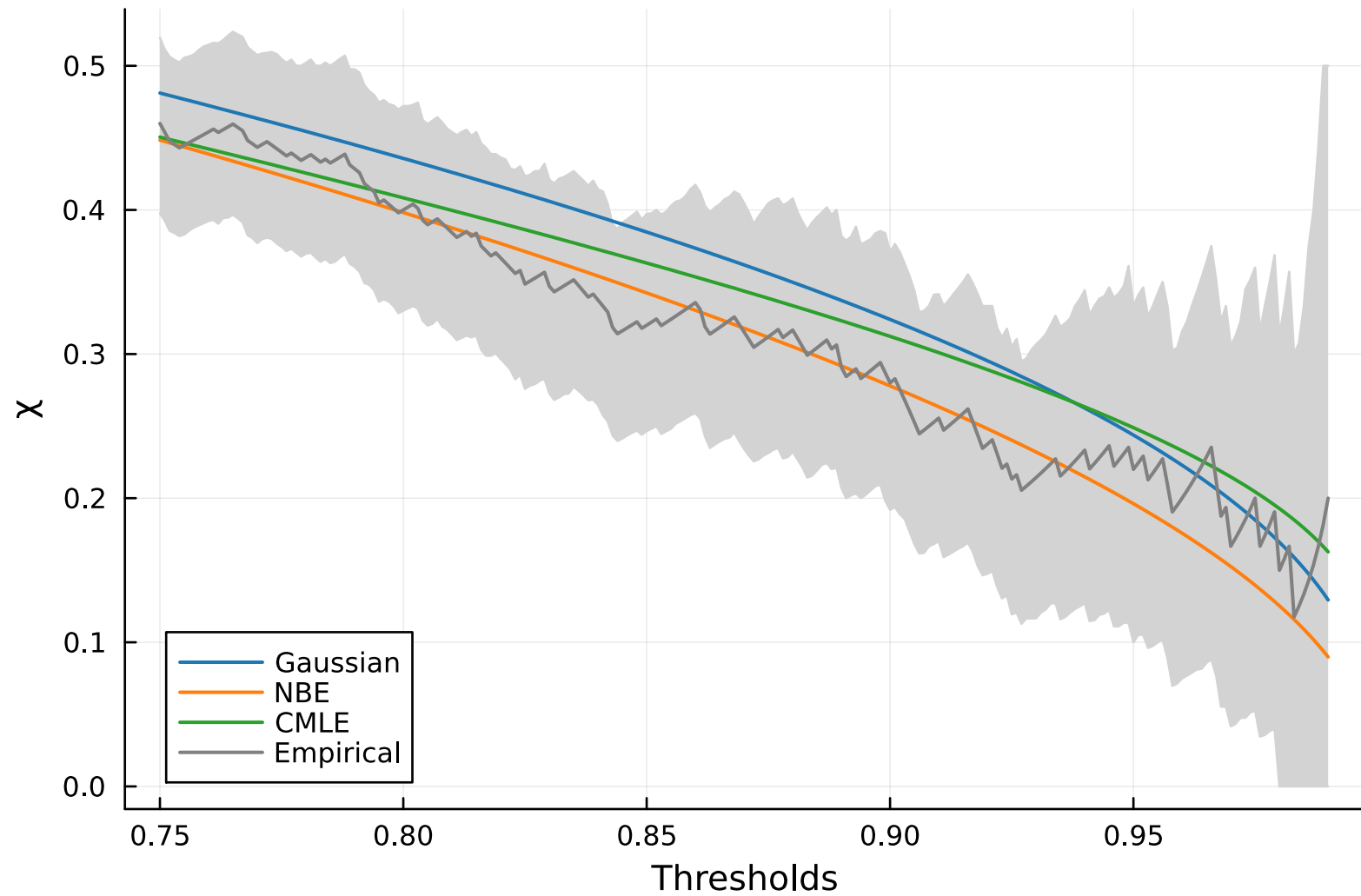

Supplement: Supplementary file 1 — (zip 5334 KB) [file 10687_2025_521_MOESM1_ESM.zip › SupplementaryMaterial/Images/chi_measures_gausE2.pdf]

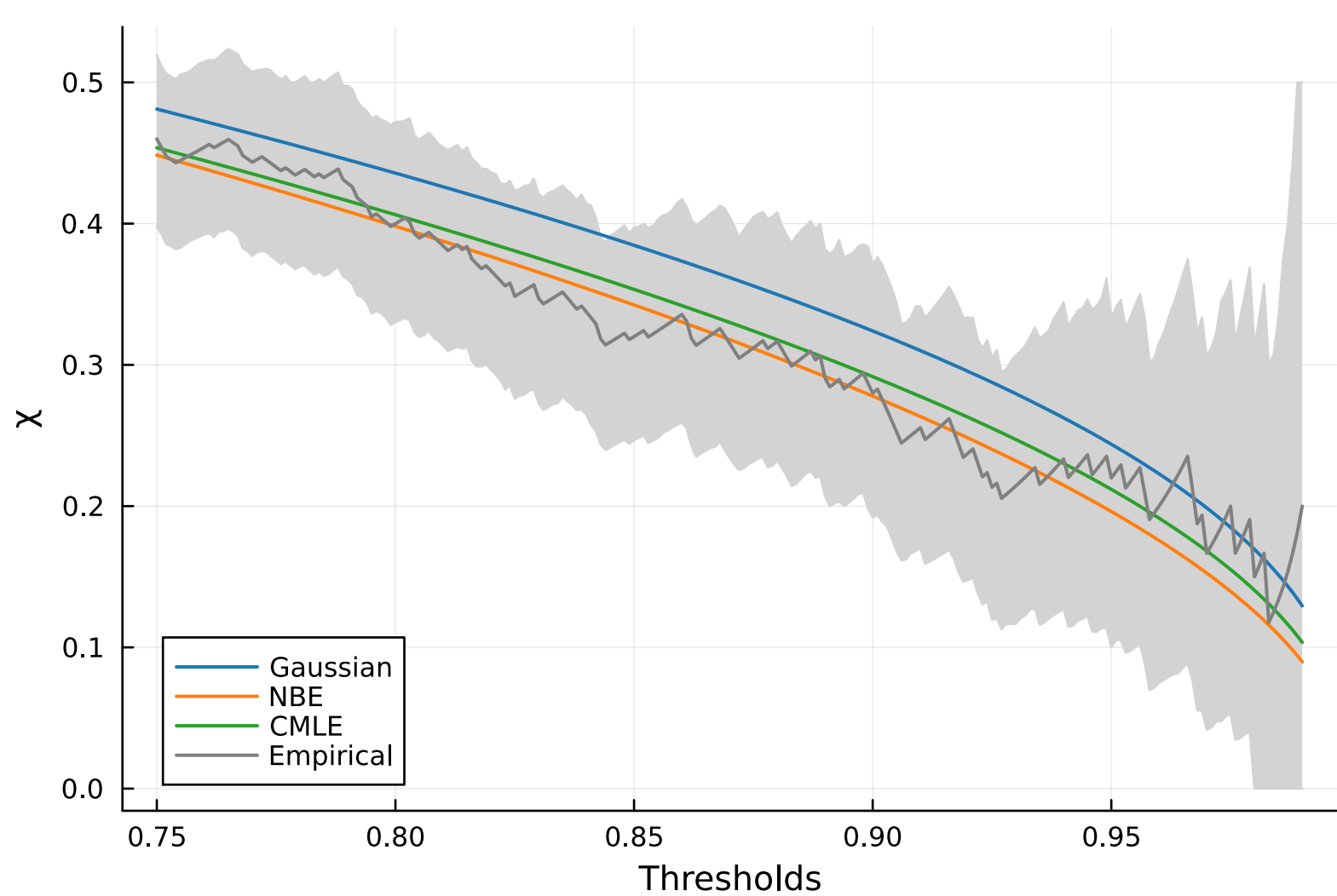

Supplement: Supplementary file 1 — (zip 5334 KB) [file 10687_2025_521_MOESM1_ESM.zip › SupplementaryMaterial/Images/chi_measures_gaus_new.pdf]

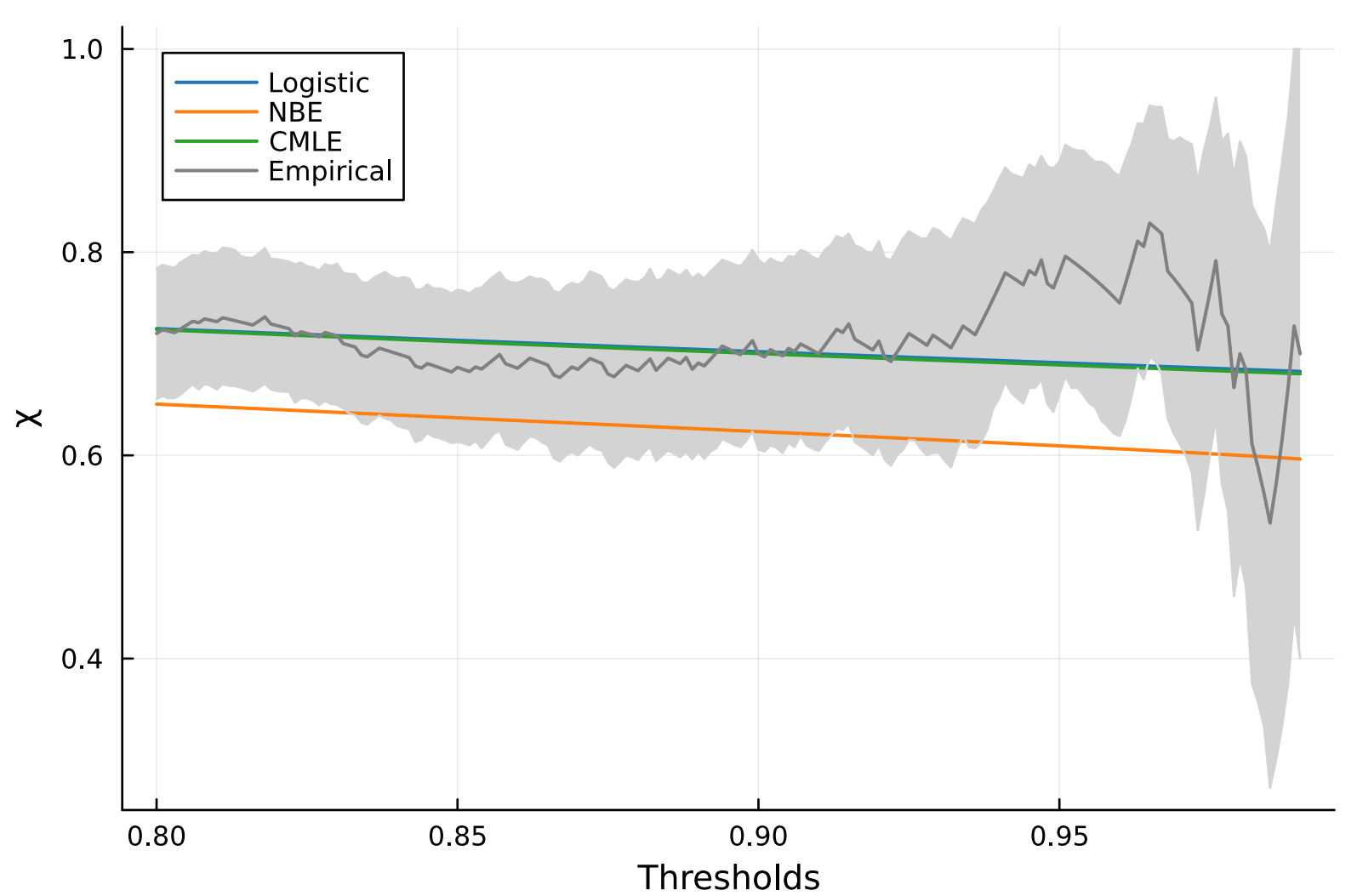

Supplement: Supplementary file 1 — (zip 5334 KB) [file 10687_2025_521_MOESM1_ESM.zip › SupplementaryMaterial/Images/chi_measures_logHW.pdf]

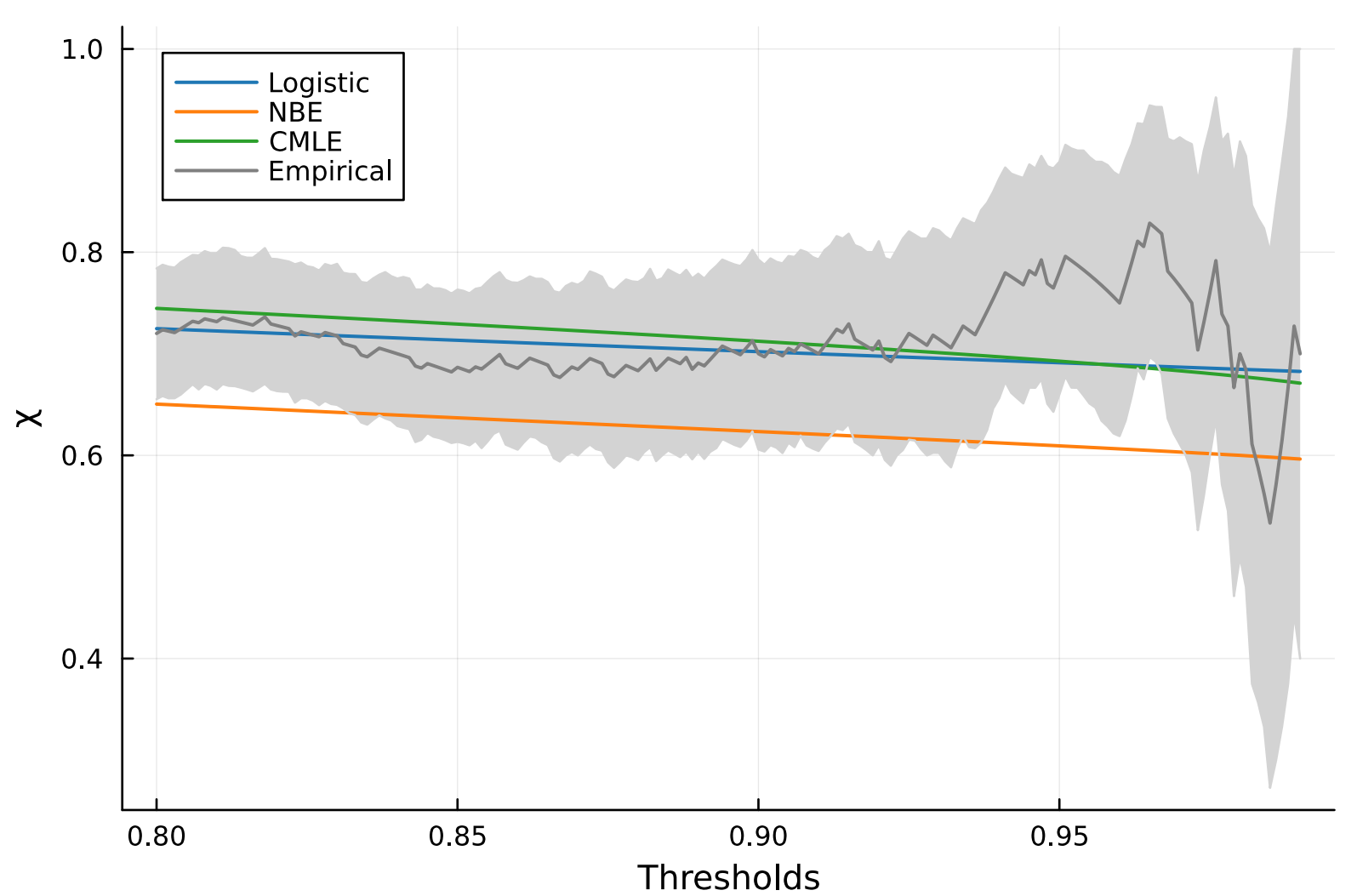

Supplement: Supplementary file 1 — (zip 5334 KB) [file 10687_2025_521_MOESM1_ESM.zip › SupplementaryMaterial/Images/chi_measures_log_new.pdf]

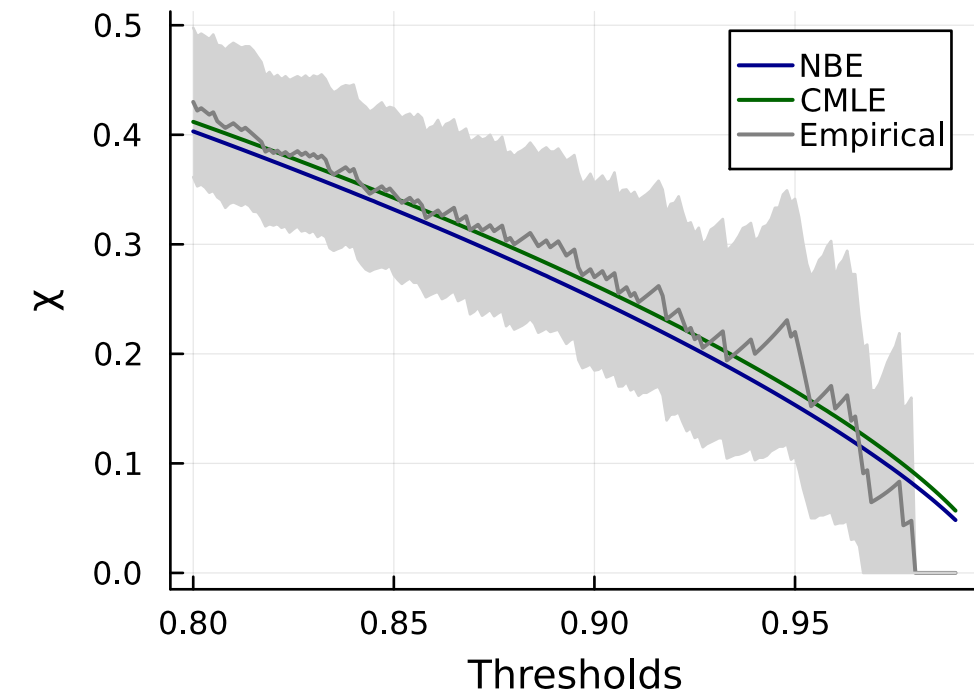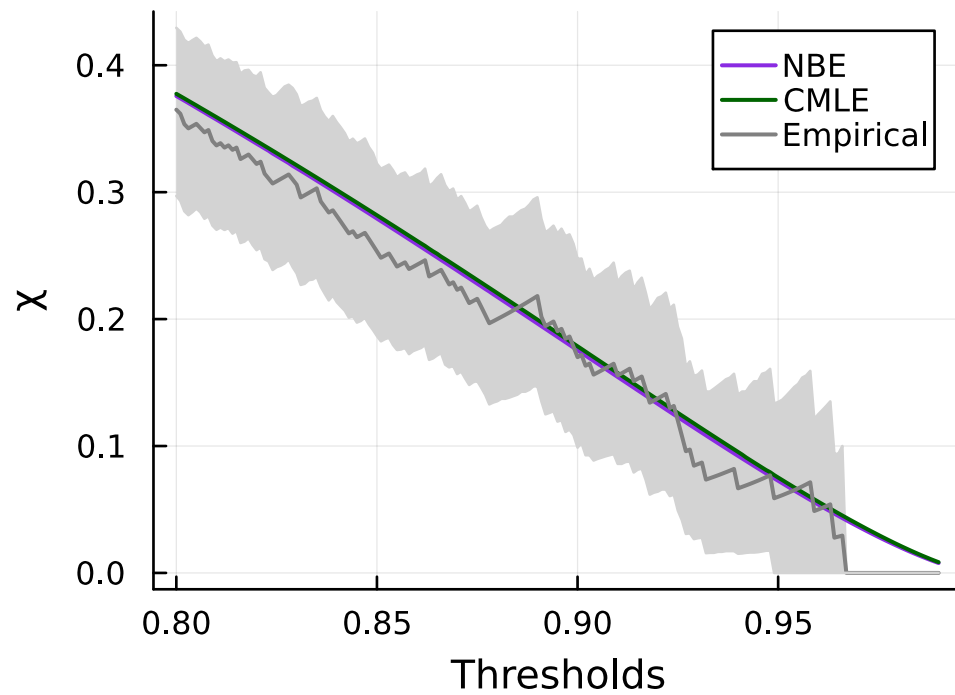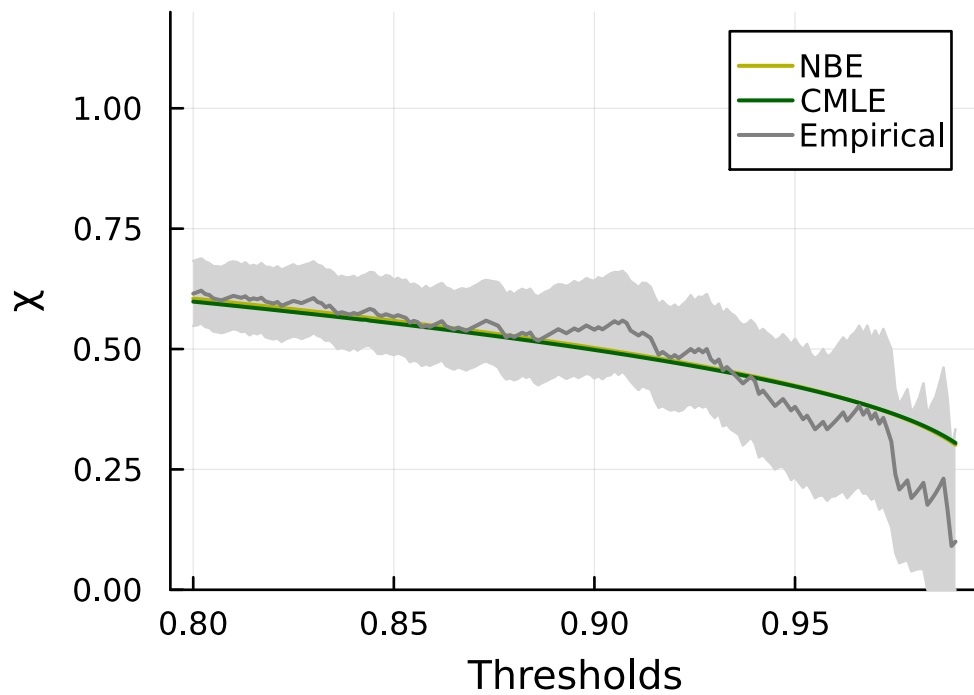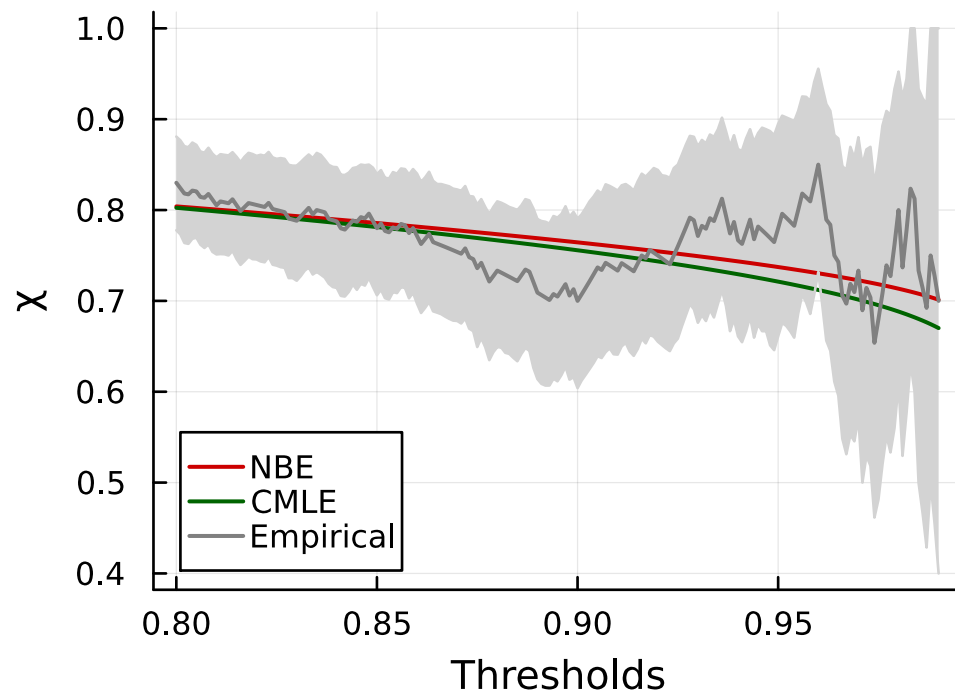

Supplement: Supplementary file 1 — (zip 5334 KB) [file 10687_2025_521_MOESM1_ESM.zip › SupplementaryMaterial/Images/chi_plots_wadsfixed.pdf]

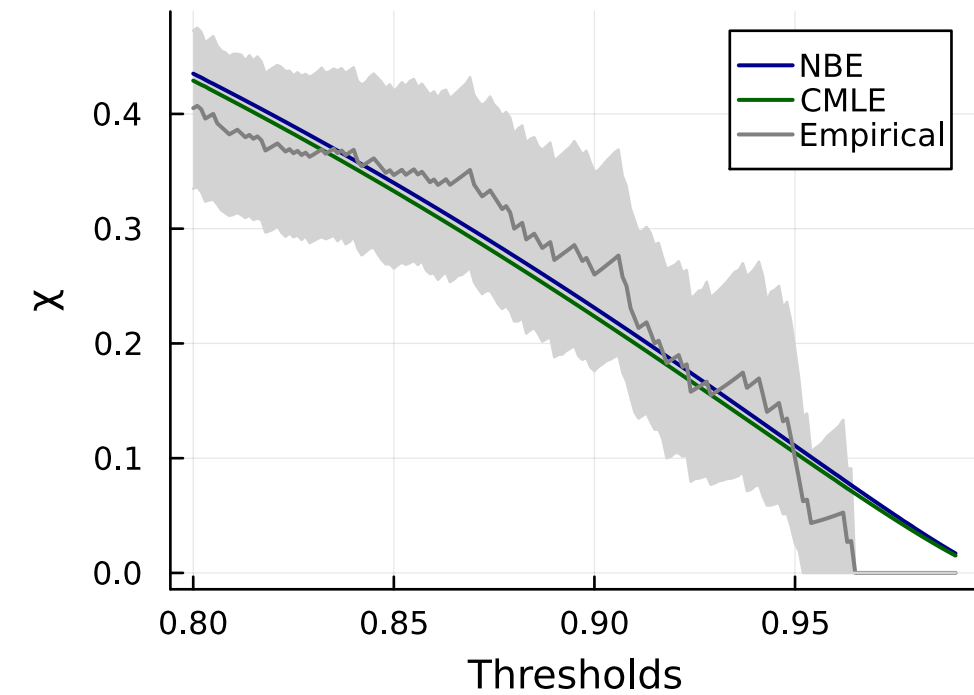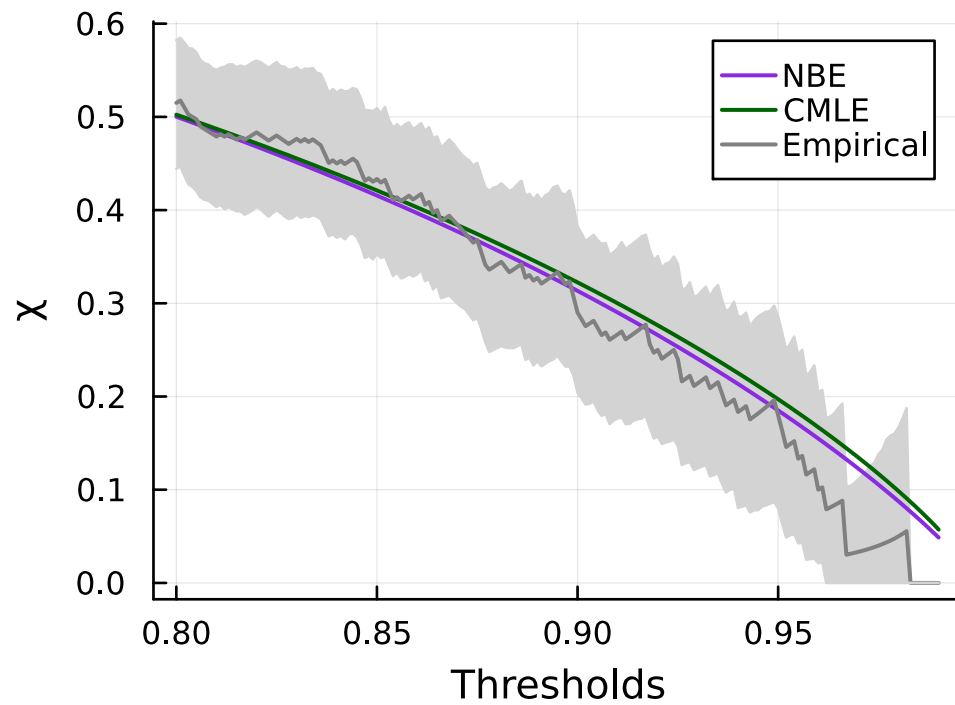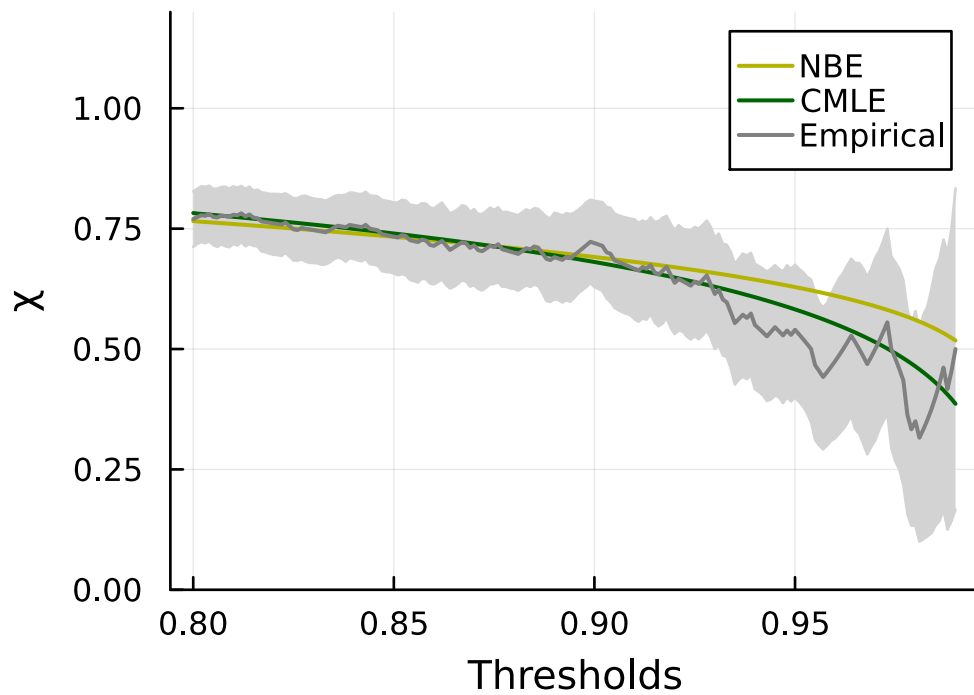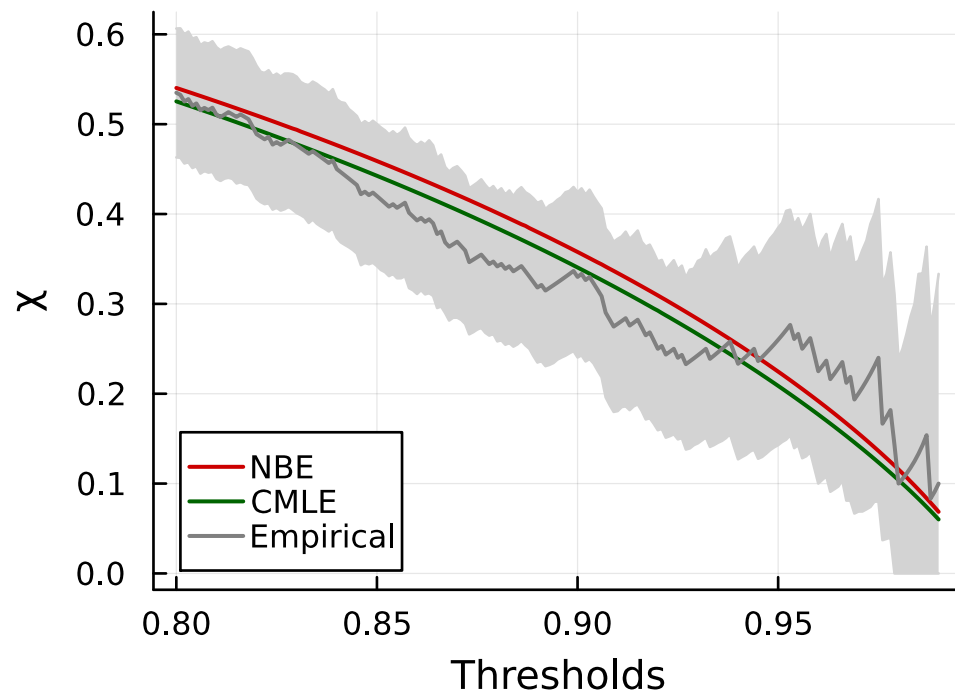

Supplement: Supplementary file 1 — (zip 5334 KB) [file 10687_2025_521_MOESM1_ESM.zip › SupplementaryMaterial/Images/chi_plots_wadsfixedvarn.pdf]

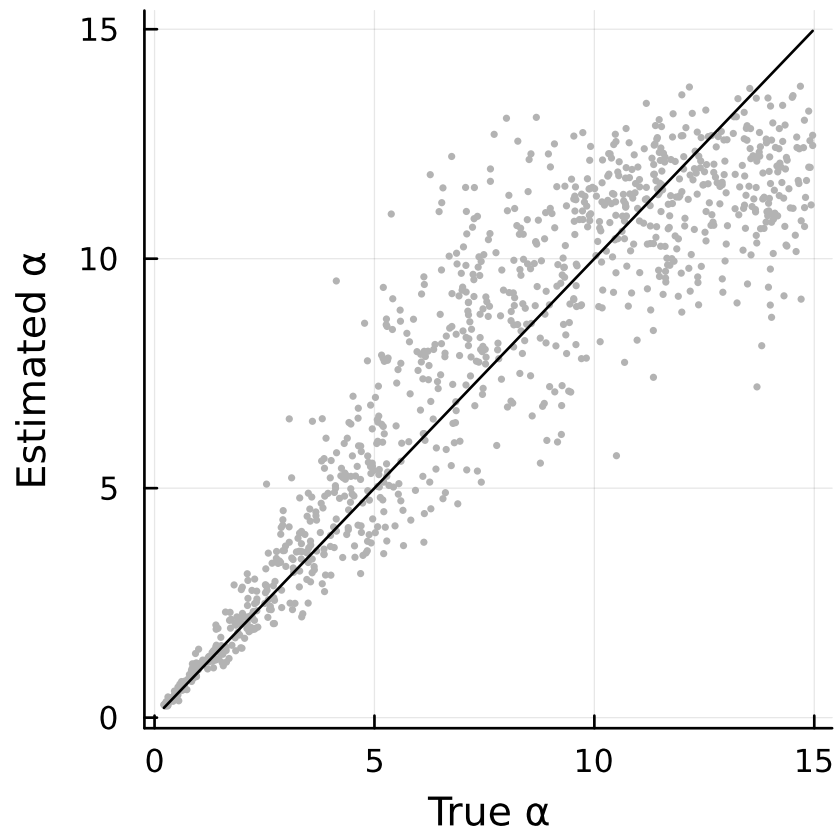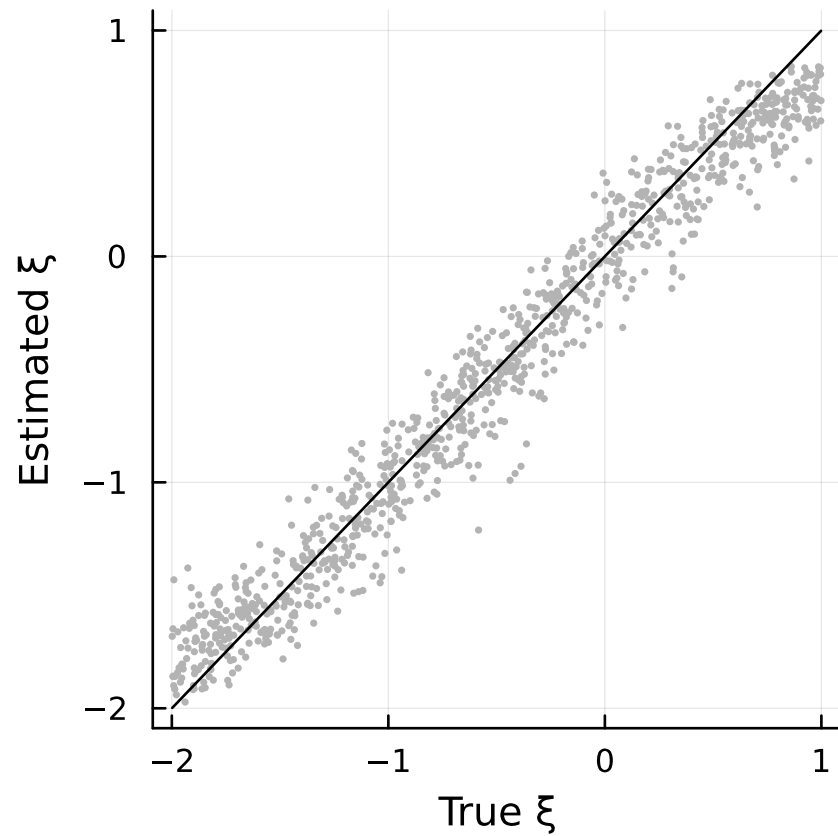

Supplement: Supplementary file 1 — (zip 5334 KB) [file 10687_2025_521_MOESM1_ESM.zip › SupplementaryMaterial/Images/estimatesNBEfixedvarn_wads.pdf]

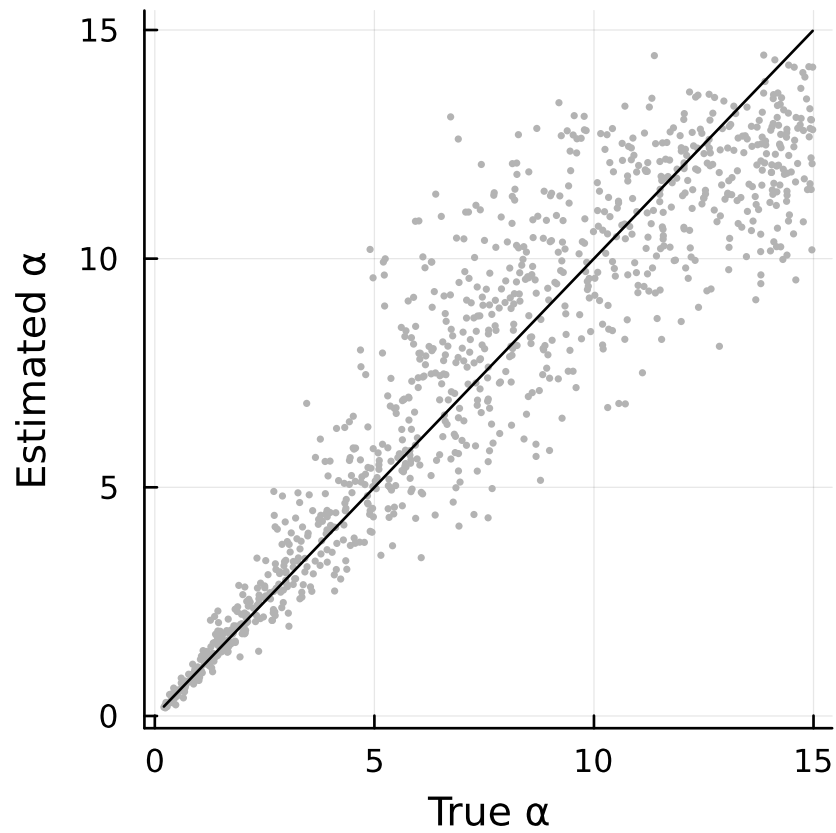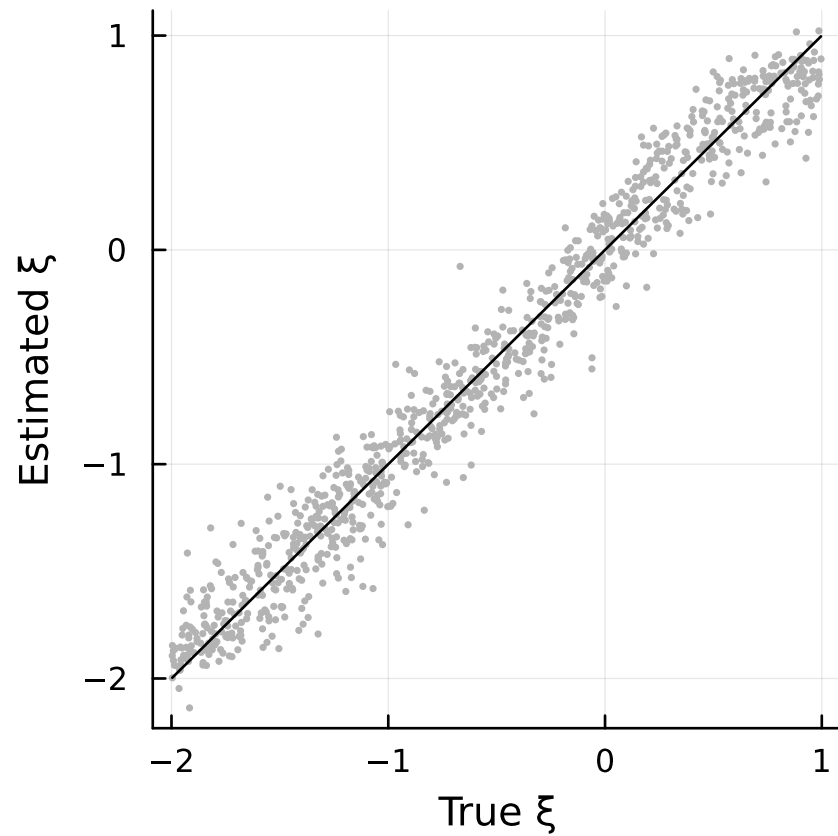

Supplement: Supplementary file 1 — (zip 5334 KB) [file 10687_2025_521_MOESM1_ESM.zip › SupplementaryMaterial/Images/estimatesNBEfixed_wads.pdf]

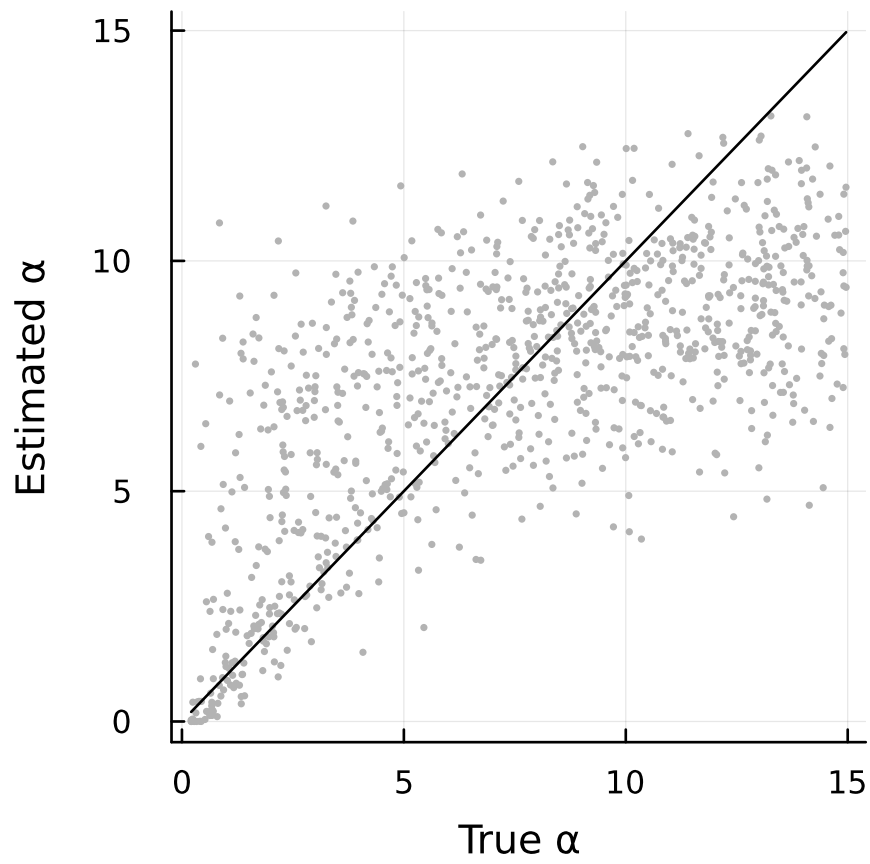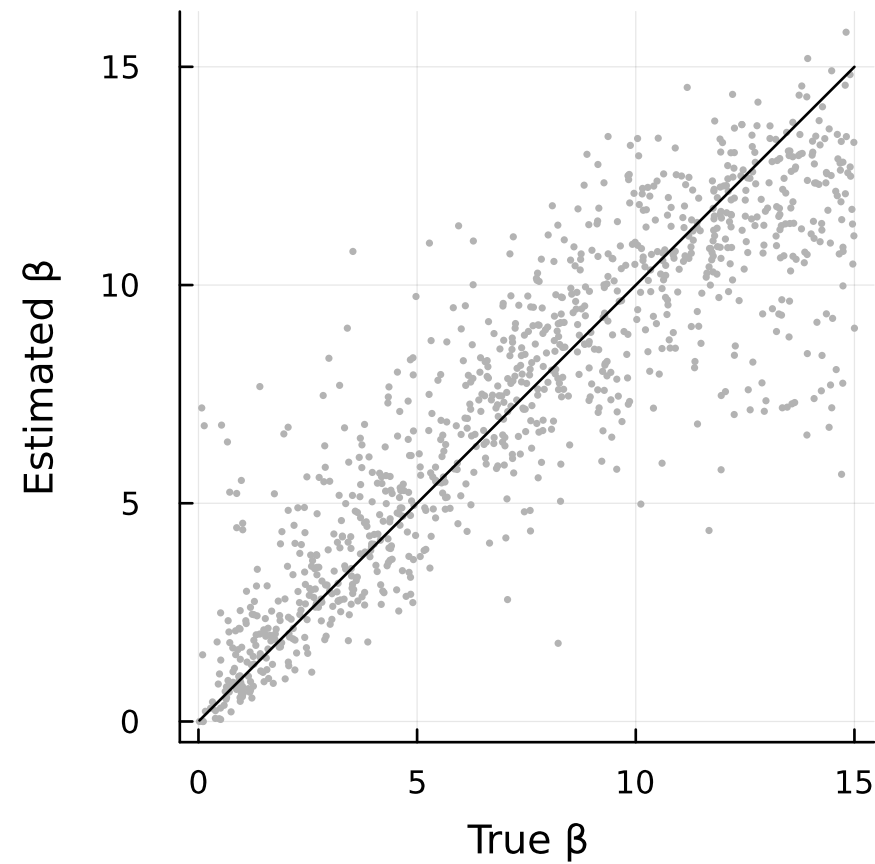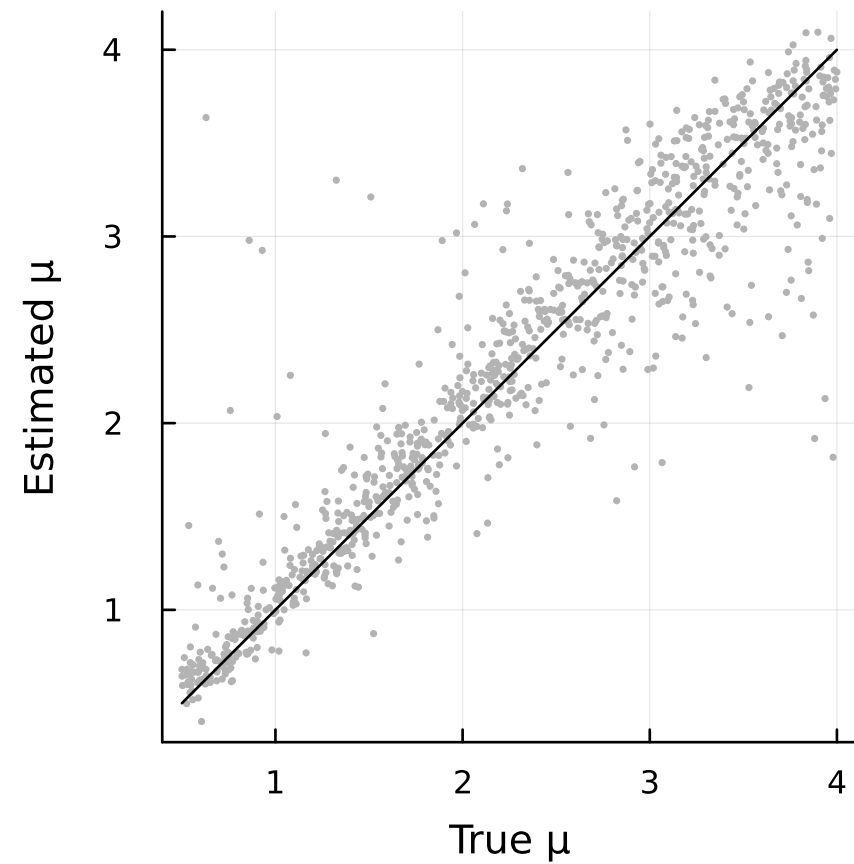

Supplement: Supplementary file 1 — (zip 5334 KB) [file 10687_2025_521_MOESM1_ESM.zip › SupplementaryMaterial/Images/estimatesNBE_eng1.pdf]

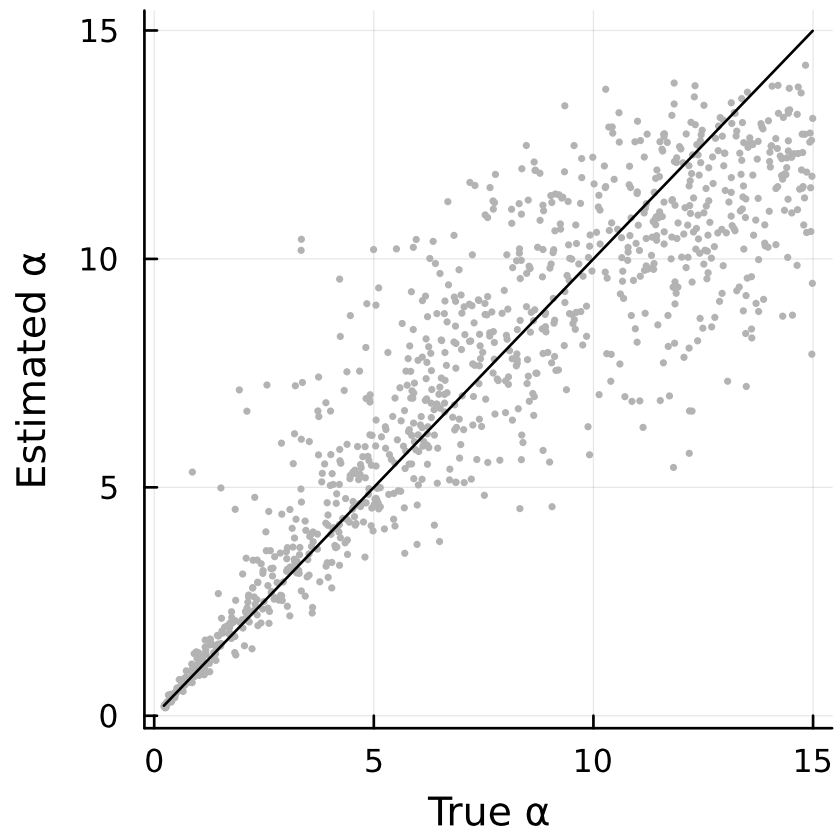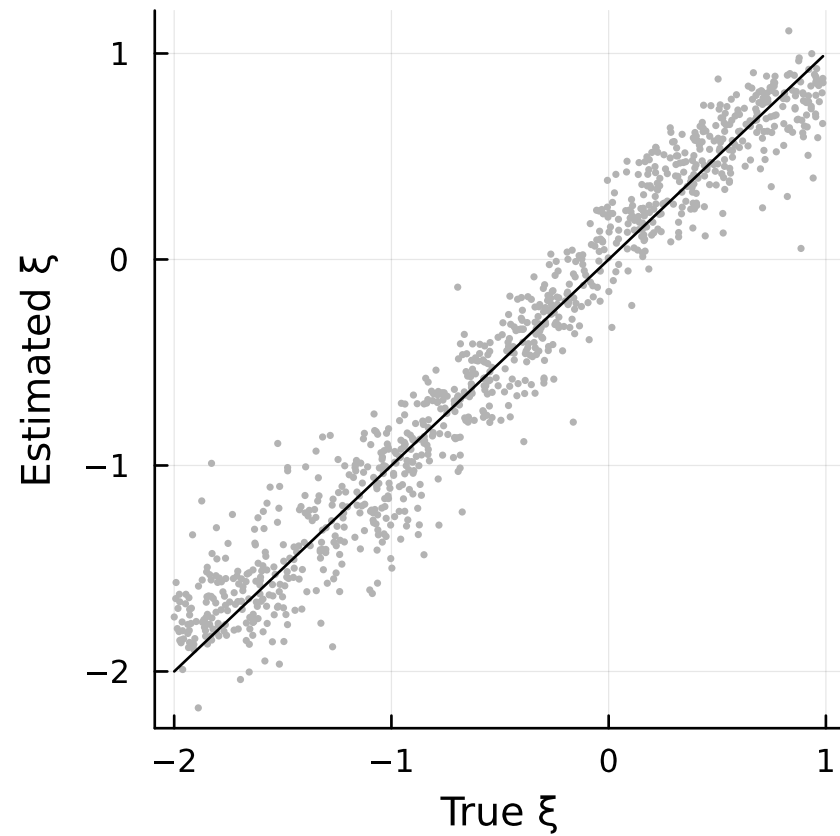

Supplement: Supplementary file 1 — (zip 5334 KB) [file 10687_2025_521_MOESM1_ESM.zip › SupplementaryMaterial/Images/estimatesNBE_eng2.pdf]

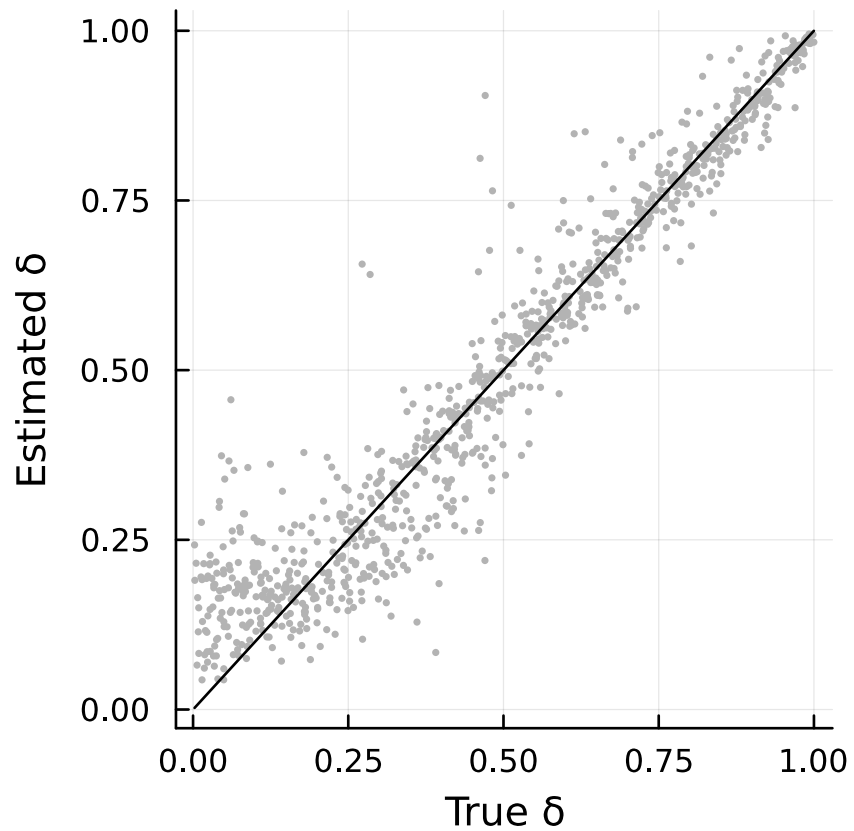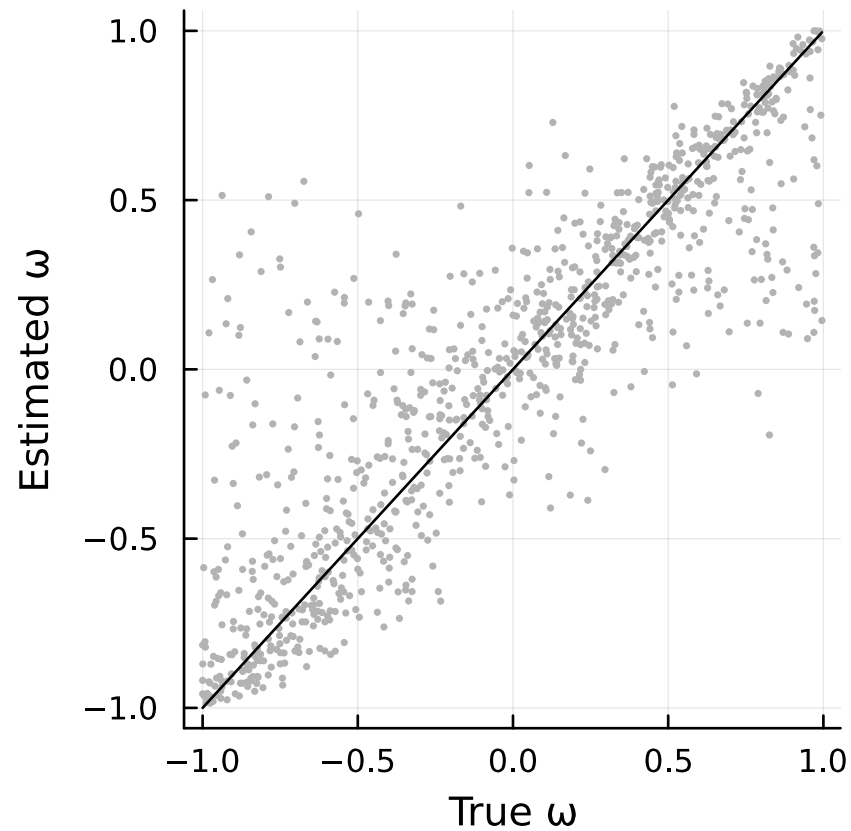

Supplement: Supplementary file 1 — (zip 5334 KB) [file 10687_2025_521_MOESM1_ESM.zip › SupplementaryMaterial/Images/estimatesNBE_hwGauss.pdf]

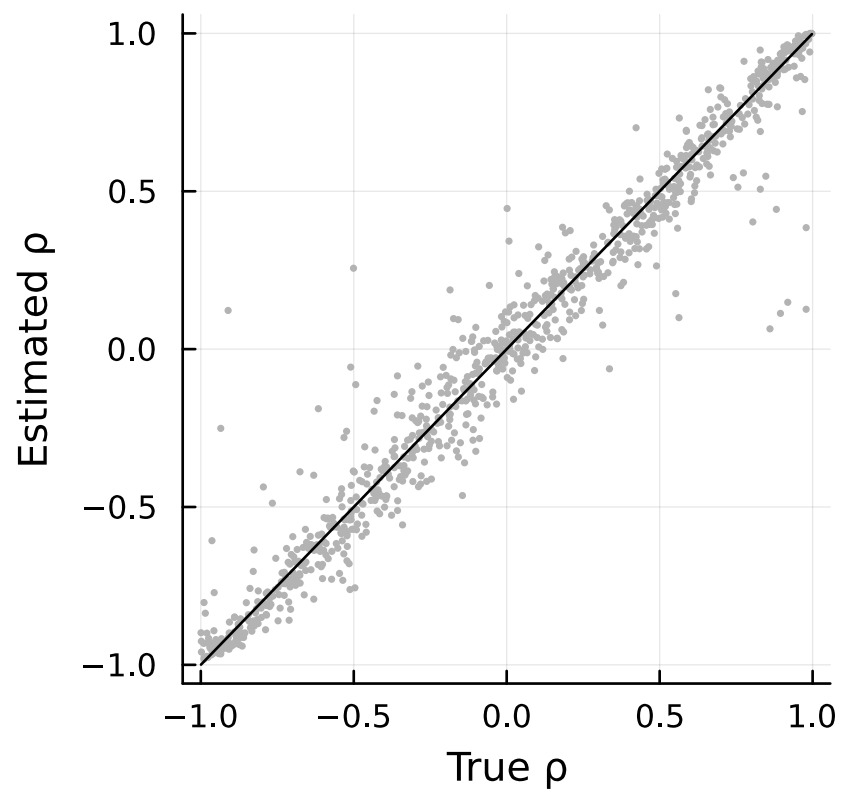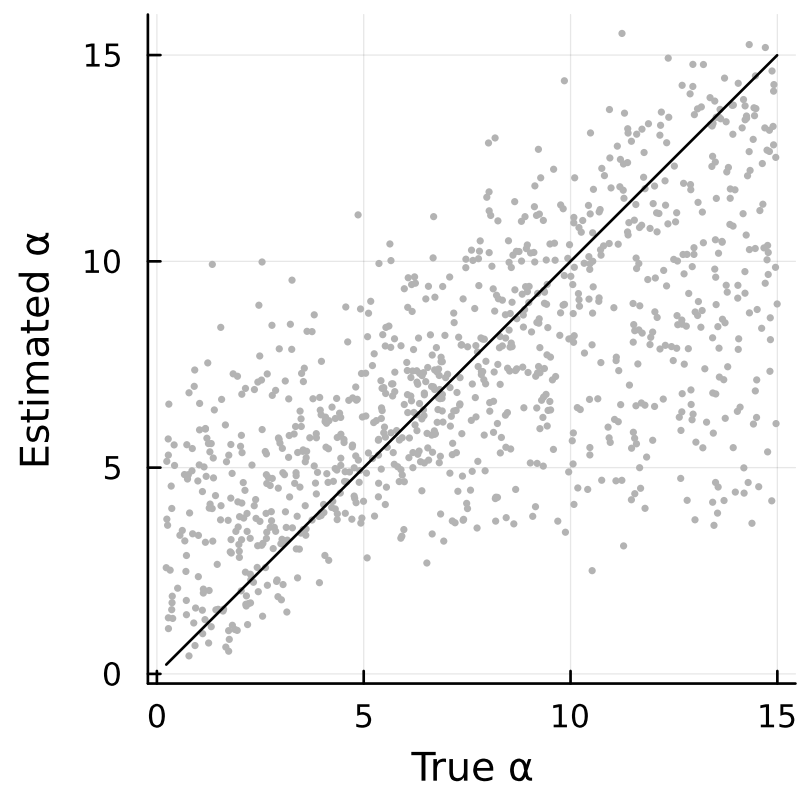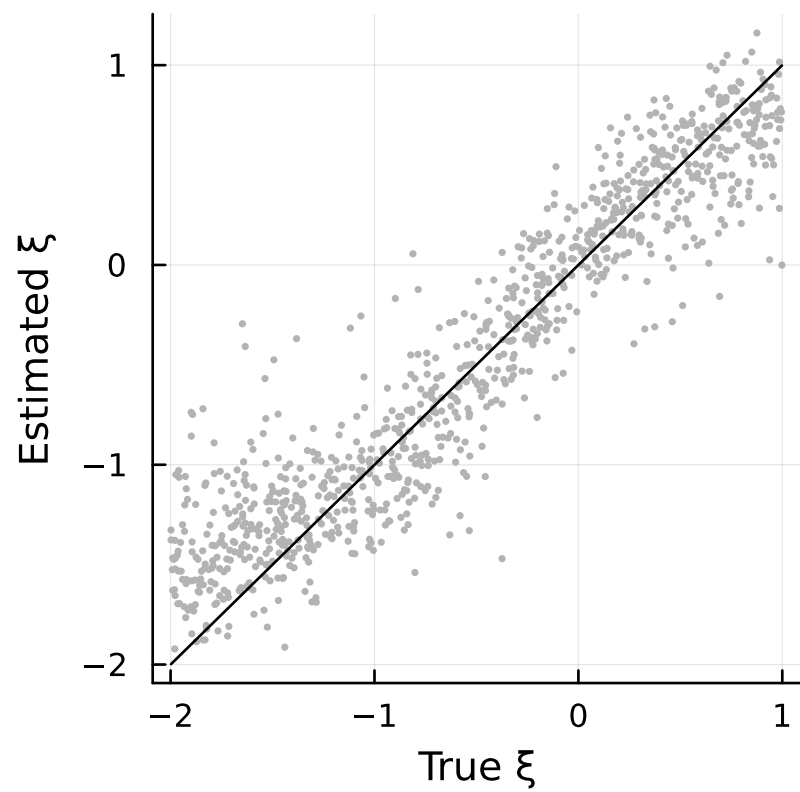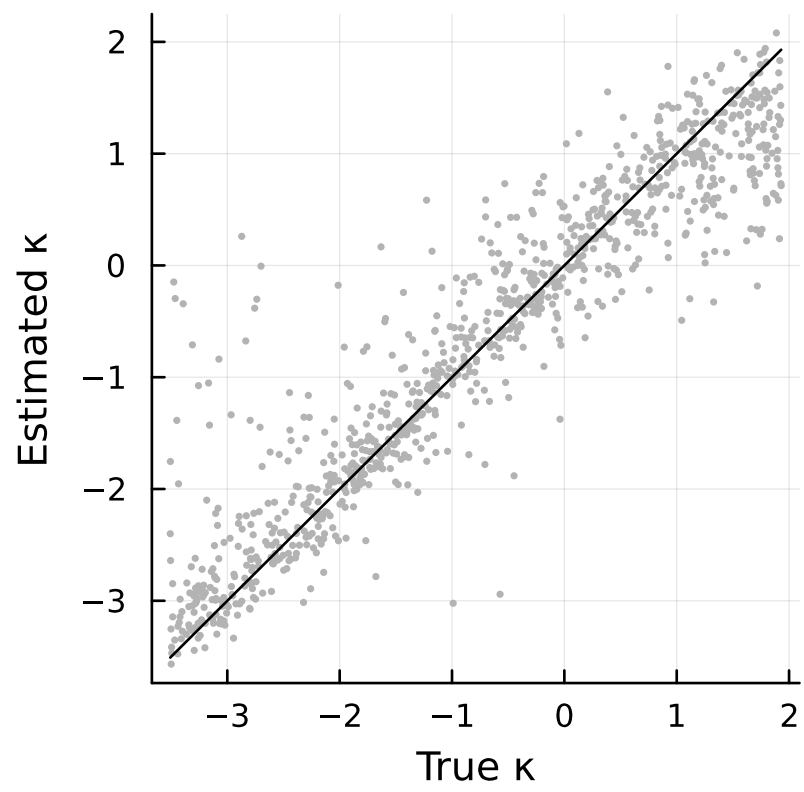

Supplement: Supplementary file 1 — (zip 5334 KB) [file 10687_2025_521_MOESM1_ESM.zip › SupplementaryMaterial/Images/estimatesNBE_wcmeng2.pdf]

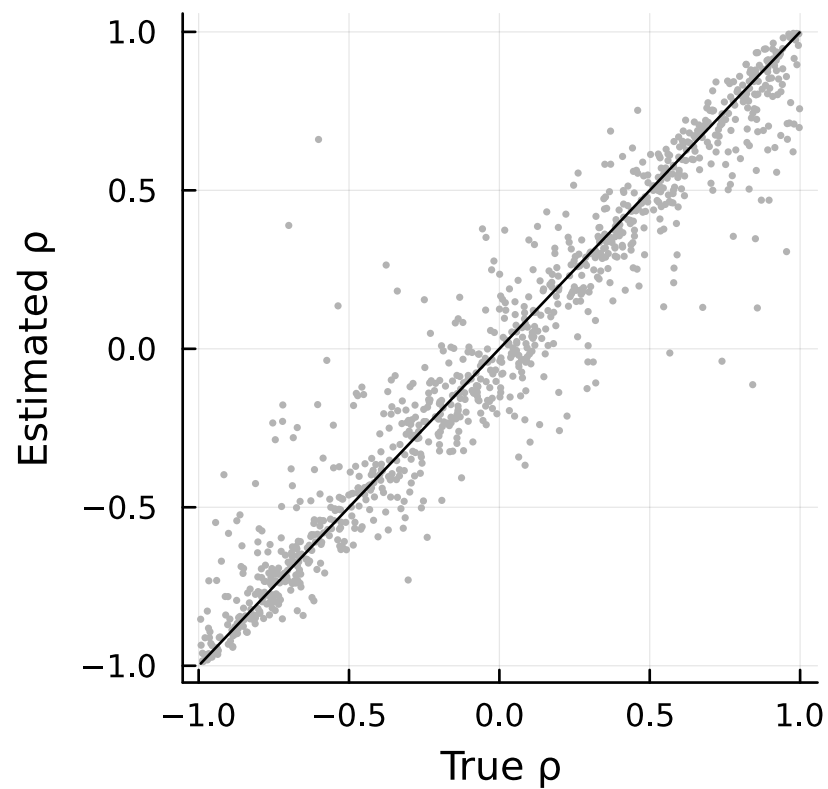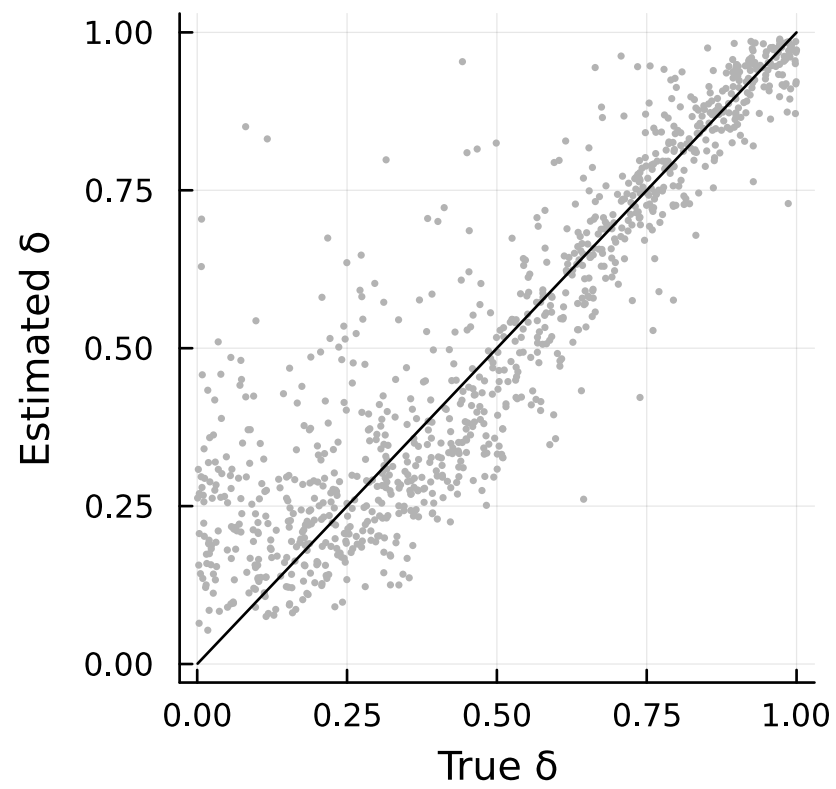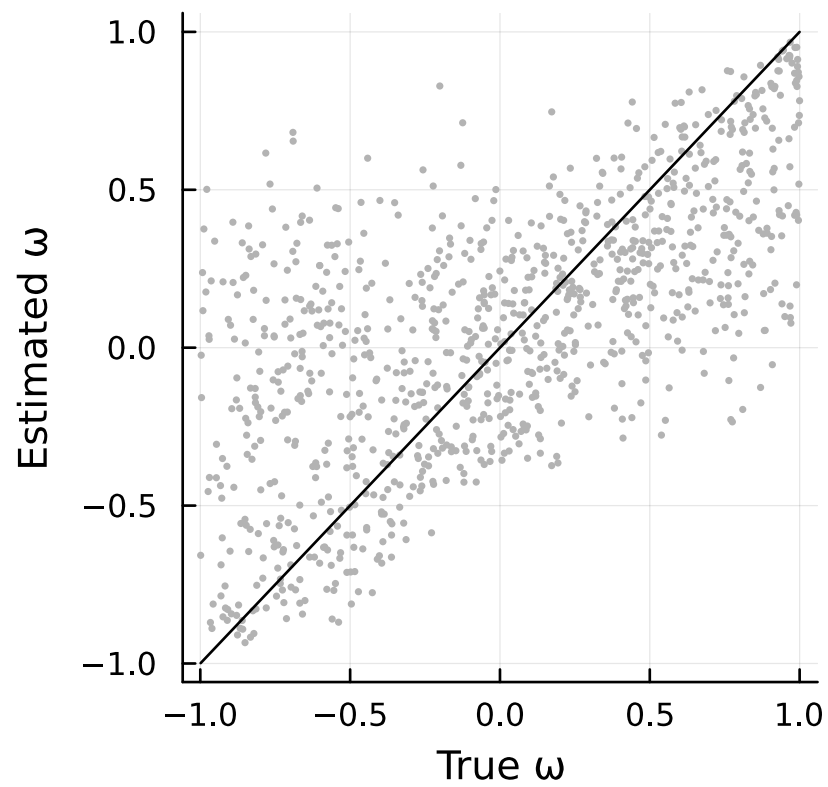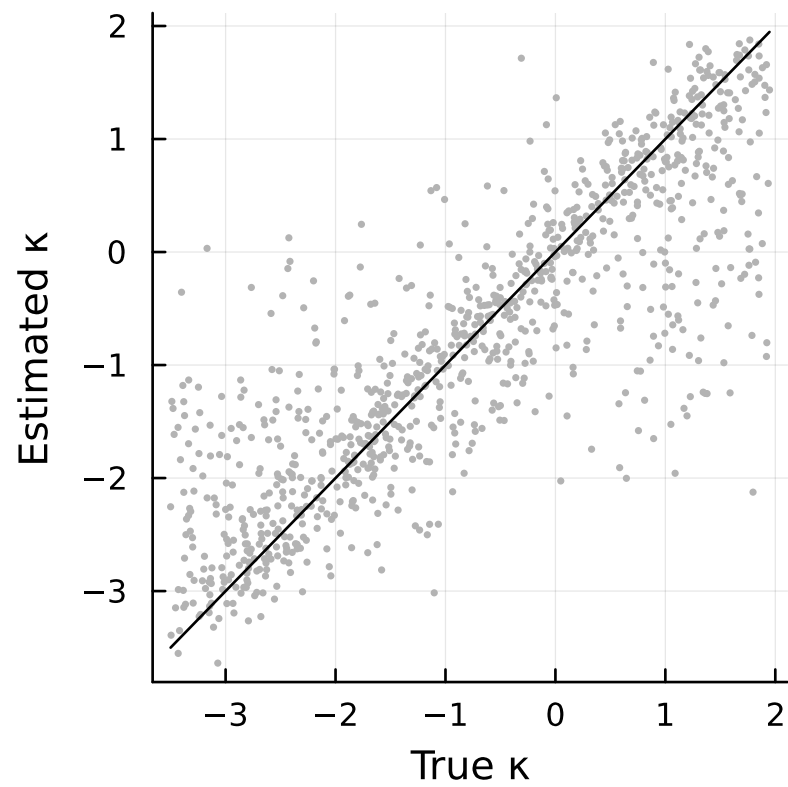

Supplement: Supplementary file 1 — (zip 5334 KB) [file 10687_2025_521_MOESM1_ESM.zip › SupplementaryMaterial/Images/estimatesNBE_wcmhwGauss.pdf]

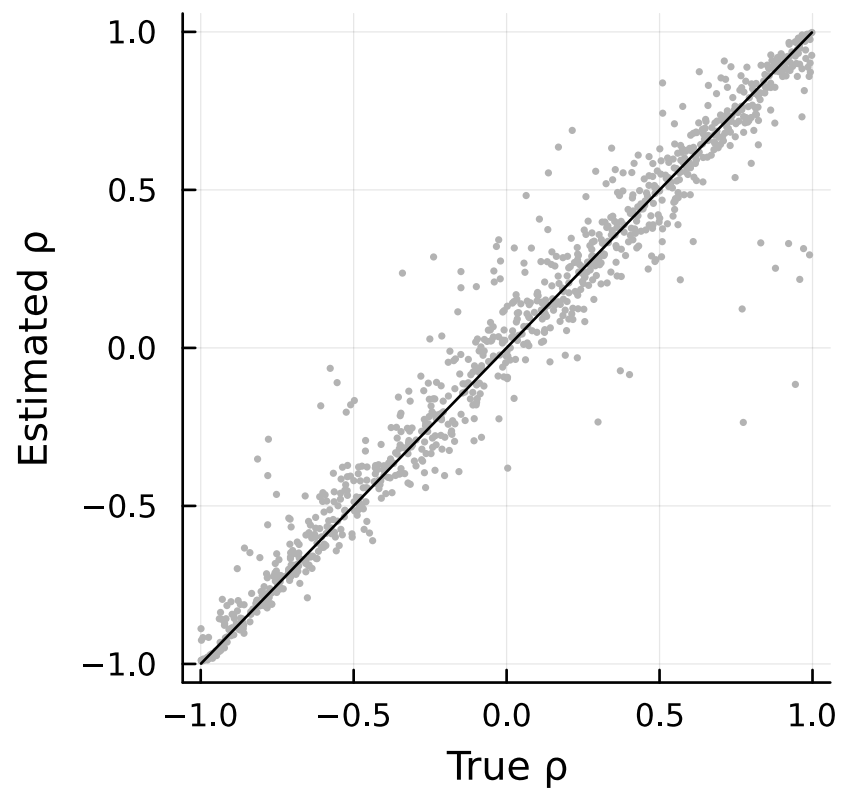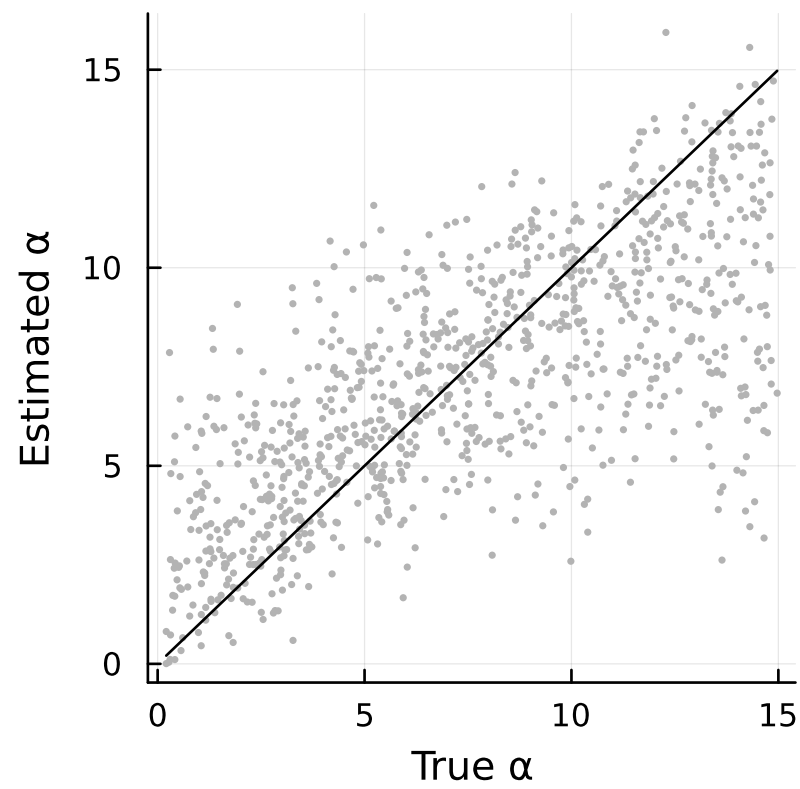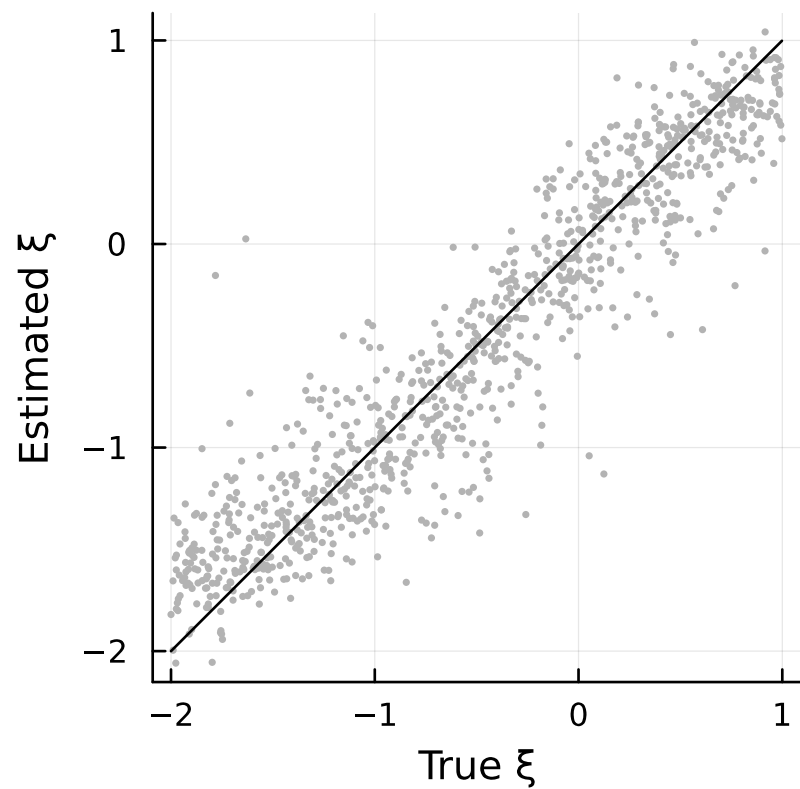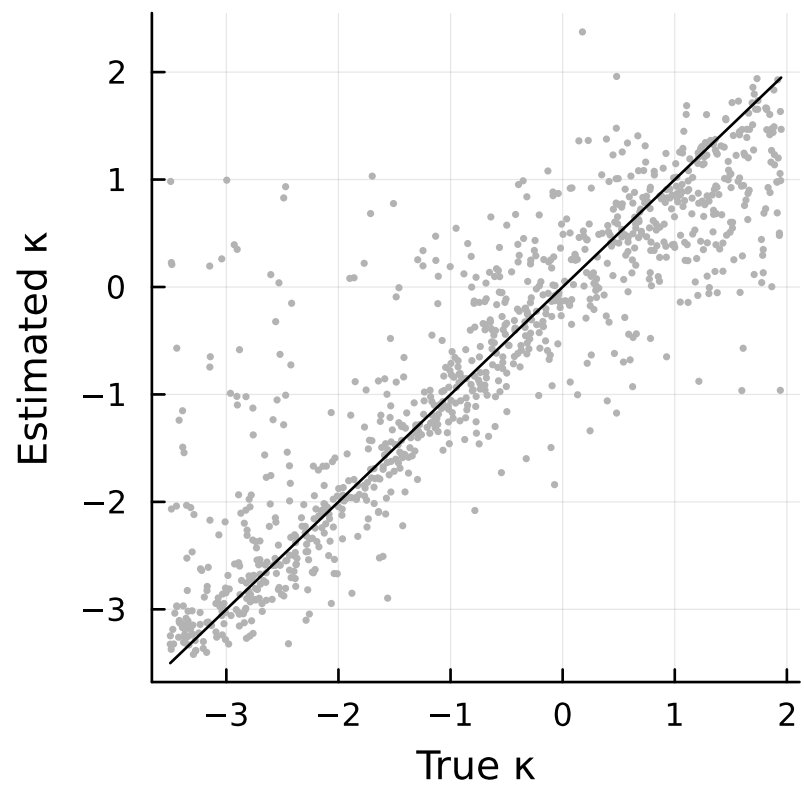

Supplement: Supplementary file 1 — (zip 5334 KB) [file 10687_2025_521_MOESM1_ESM.zip › SupplementaryMaterial/Images/estimatesNBE_wcmwads.pdf]

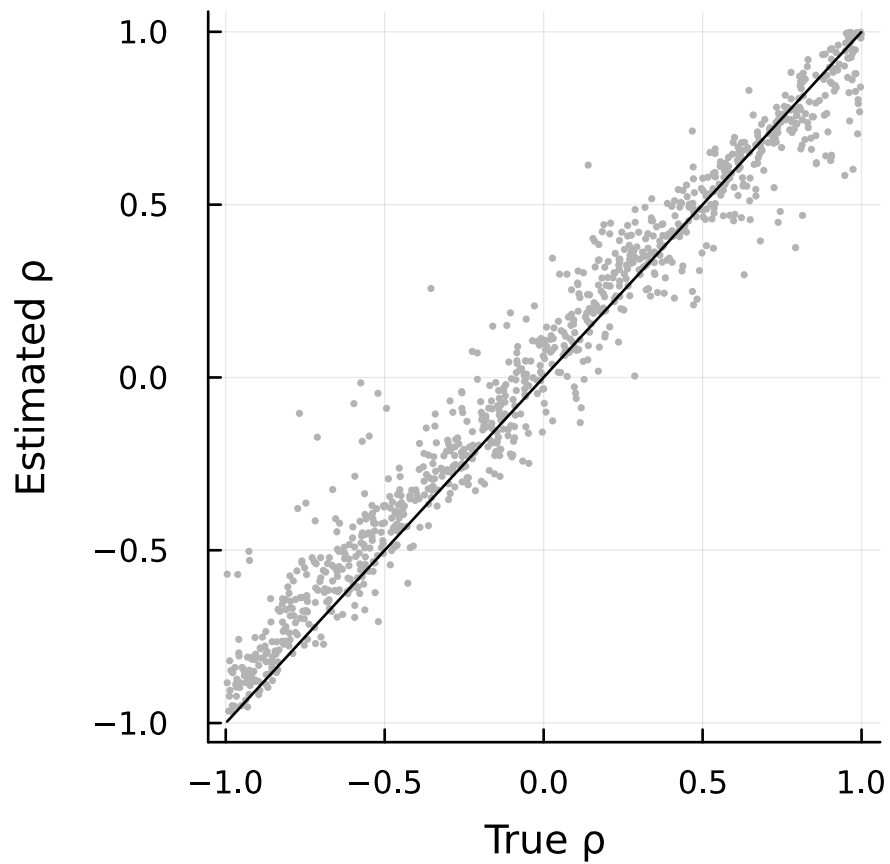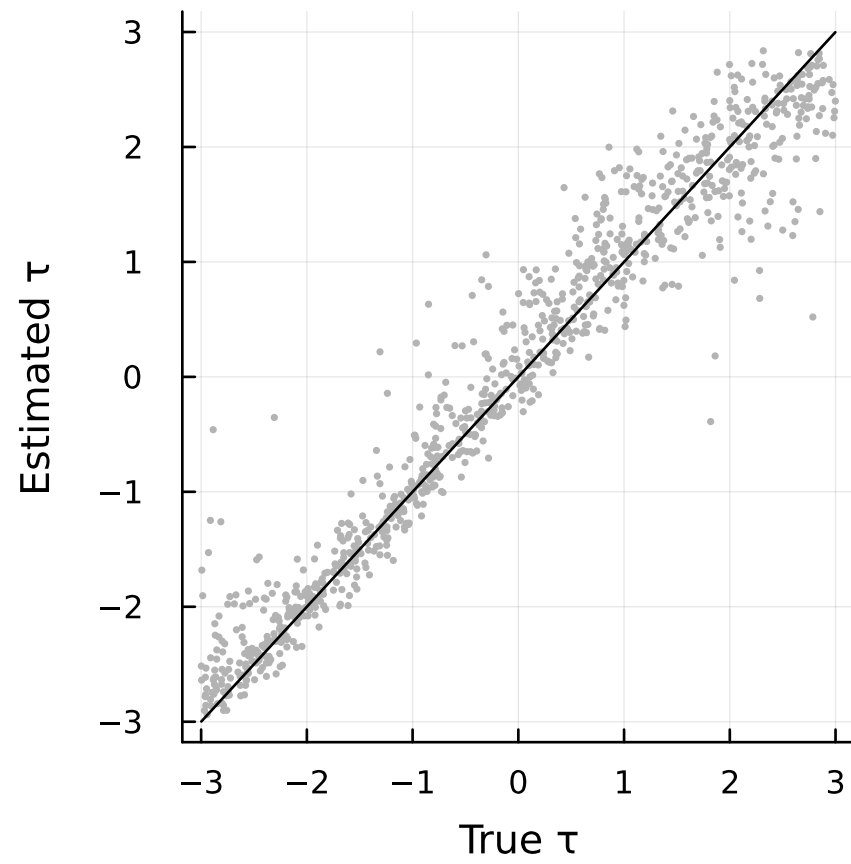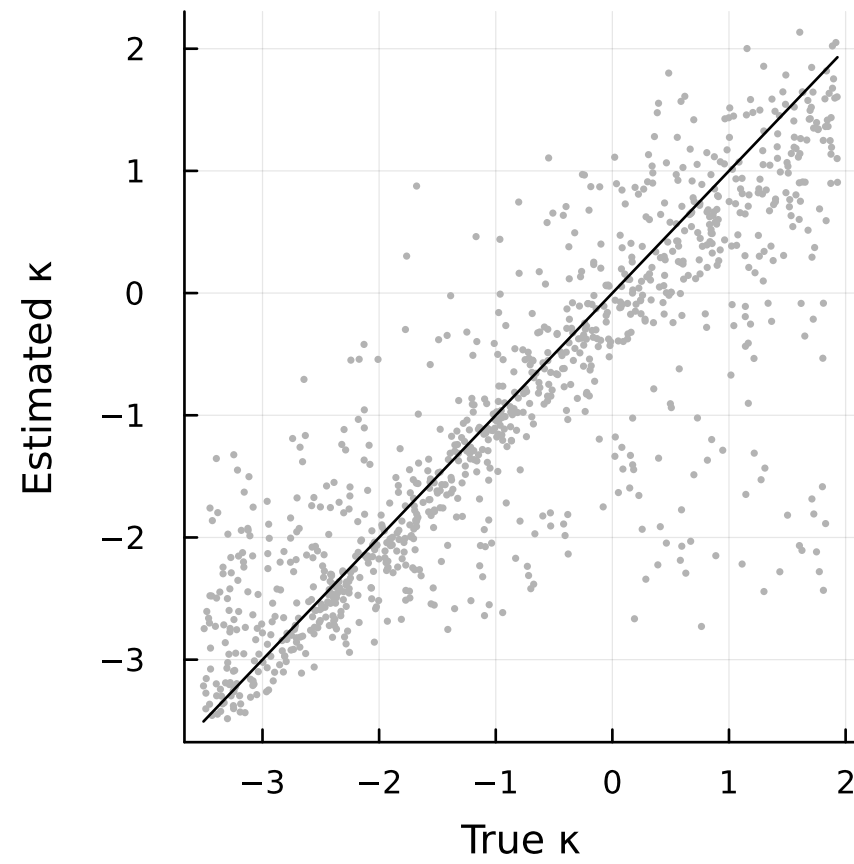

Supplement: Supplementary file 1 — (zip 5334 KB) [file 10687_2025_521_MOESM1_ESM.zip › SupplementaryMaterial/Images/estimatesNBE_wcm_mod1.pdf]

Estimated  $\beta$ 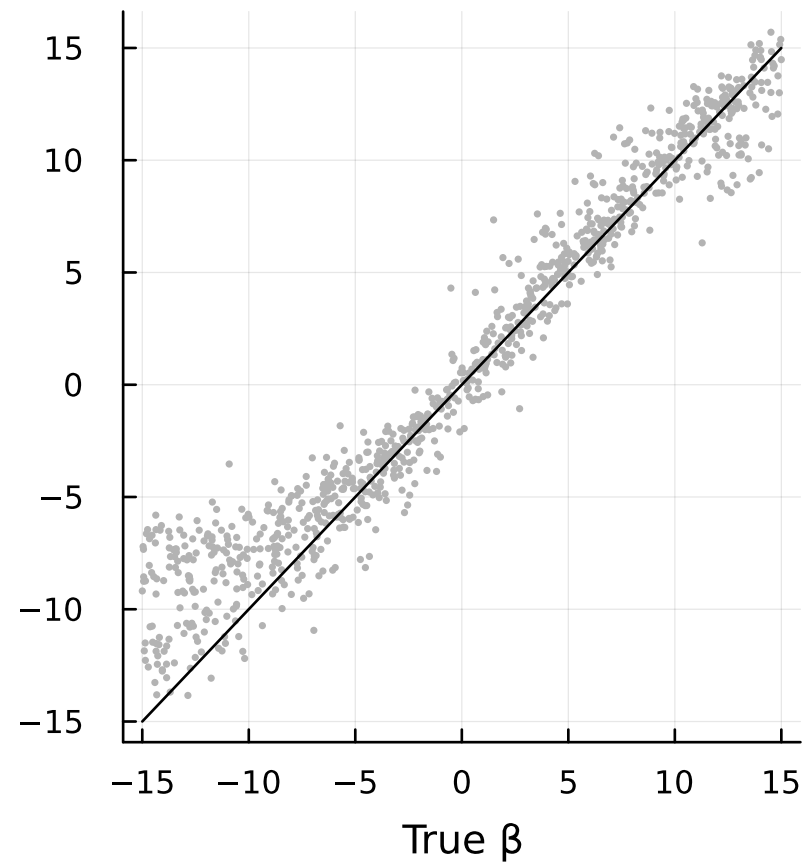Estimated  $\alpha$ 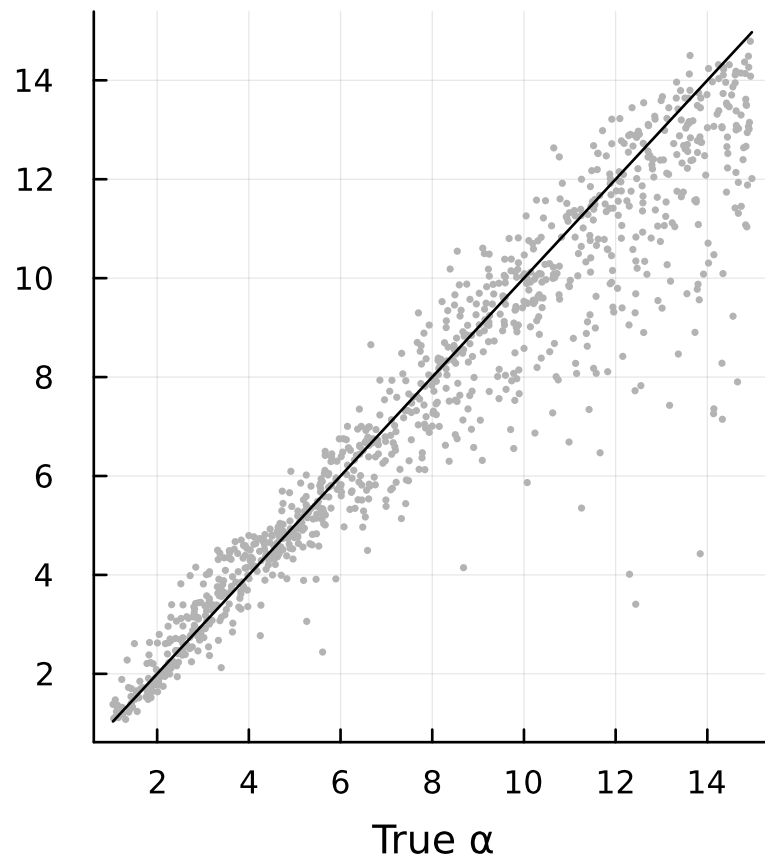Estimated  $\kappa$ 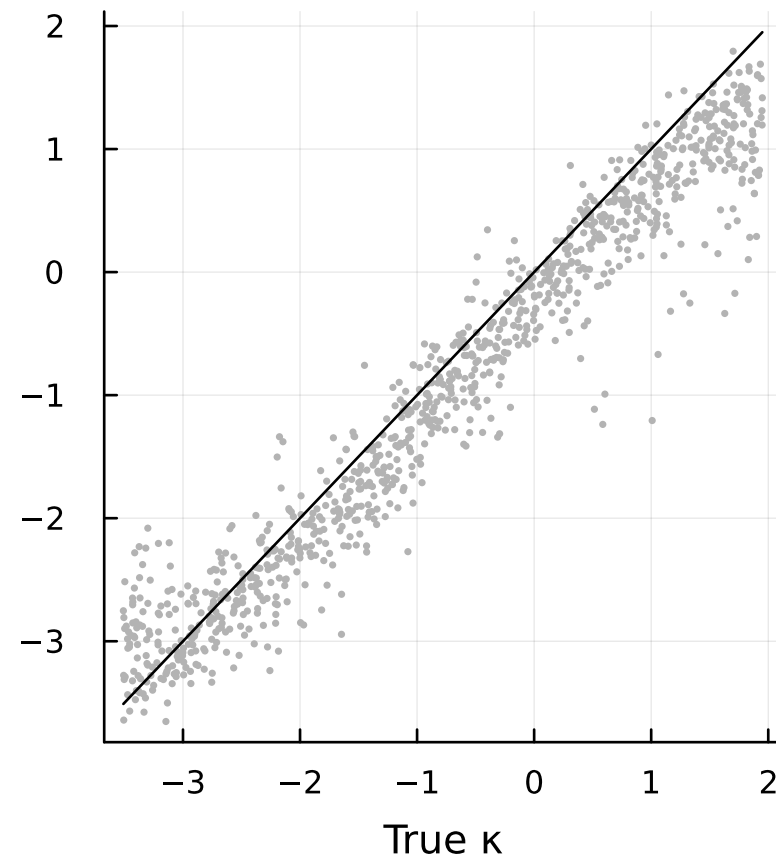

Supplement: Supplementary file 1 — (zip 5334 KB) [file 10687_2025_521_MOESM1_ESM.zip › SupplementaryMaterial/Images/estimatesNBE_wcm_mod2.pdf]

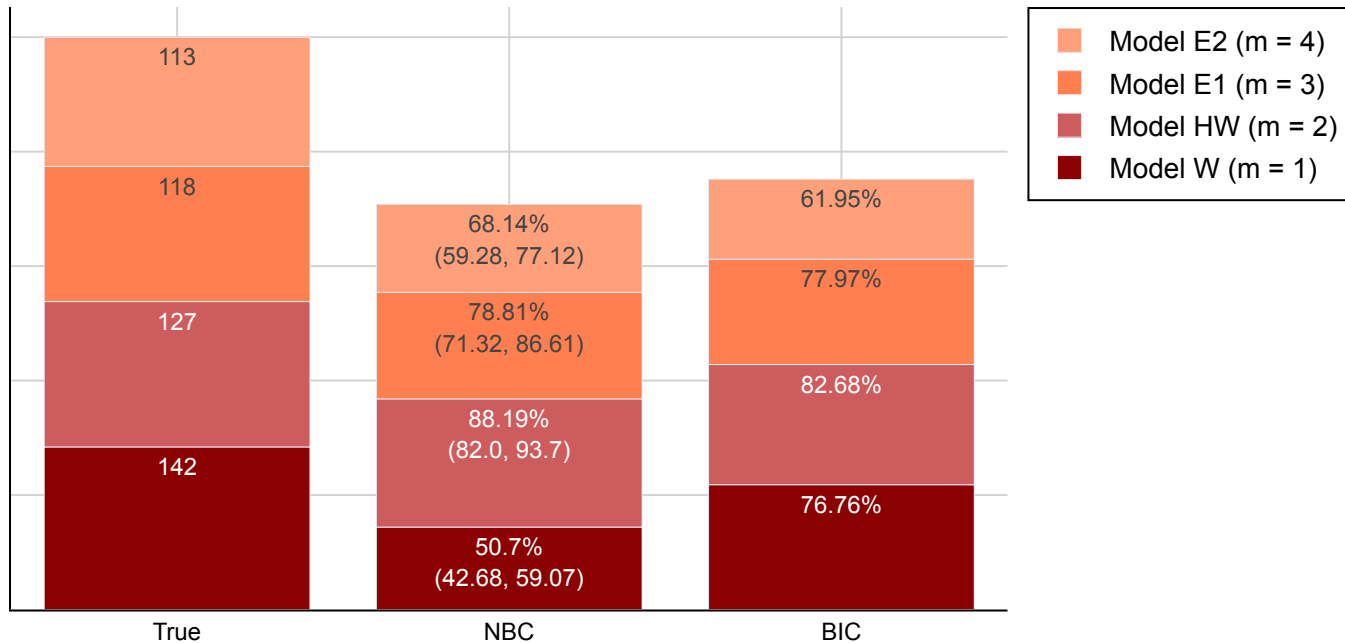

Supplement: Supplementary file 1 — (zip 5334 KB) [file 10687_2025_521_MOESM1_ESM.zip › SupplementaryMaterial/Images/multiclass_n1400.pdf]

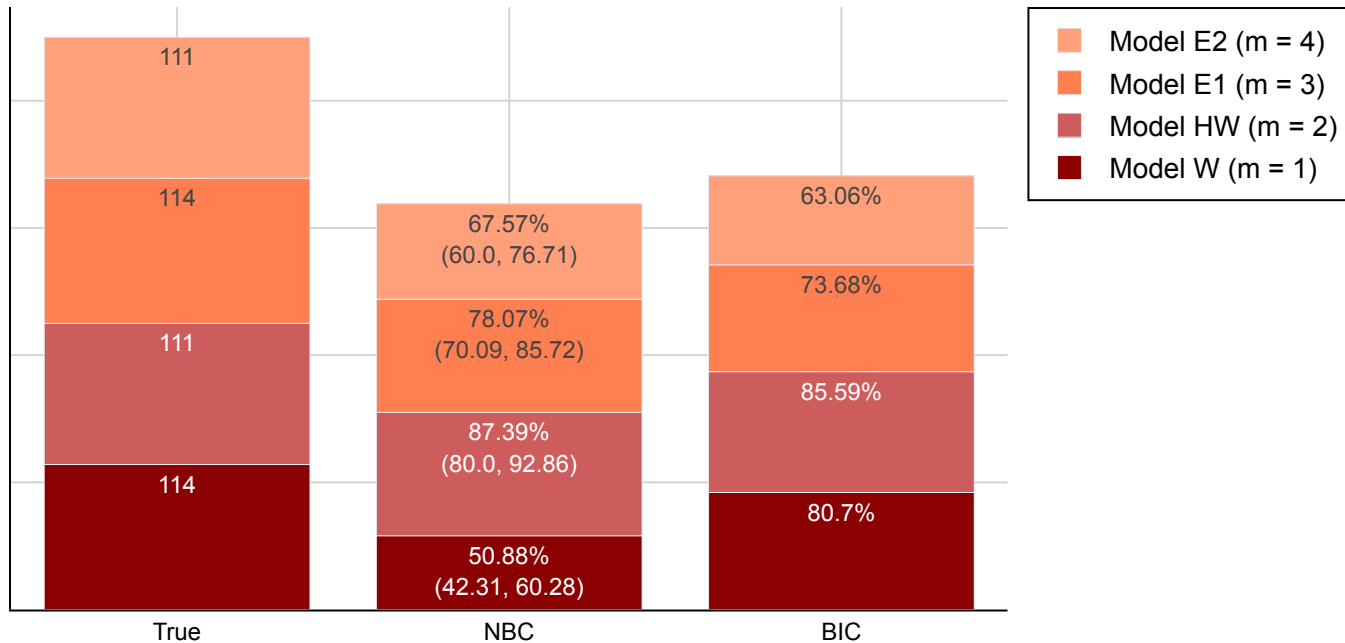

Supplement: Supplementary file 1 — (zip 5334 KB) [file 10687_2025_521_MOESM1_ESM.zip › SupplementaryMaterial/Images/multiclass_n1400_1_450.pdf]

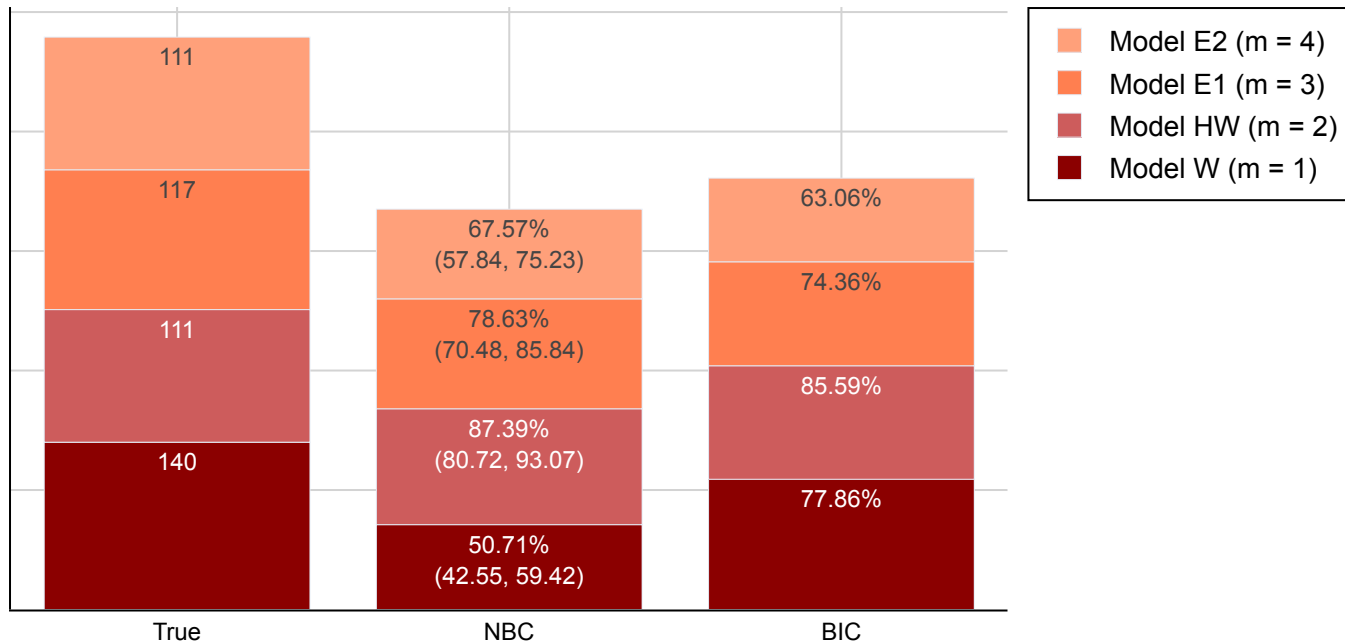

Supplement: Supplementary file 1 — (zip 5334 KB) [file 10687_2025_521_MOESM1_ESM.zip › SupplementaryMaterial/Images/multiclass_n1400_1_479.pdf]

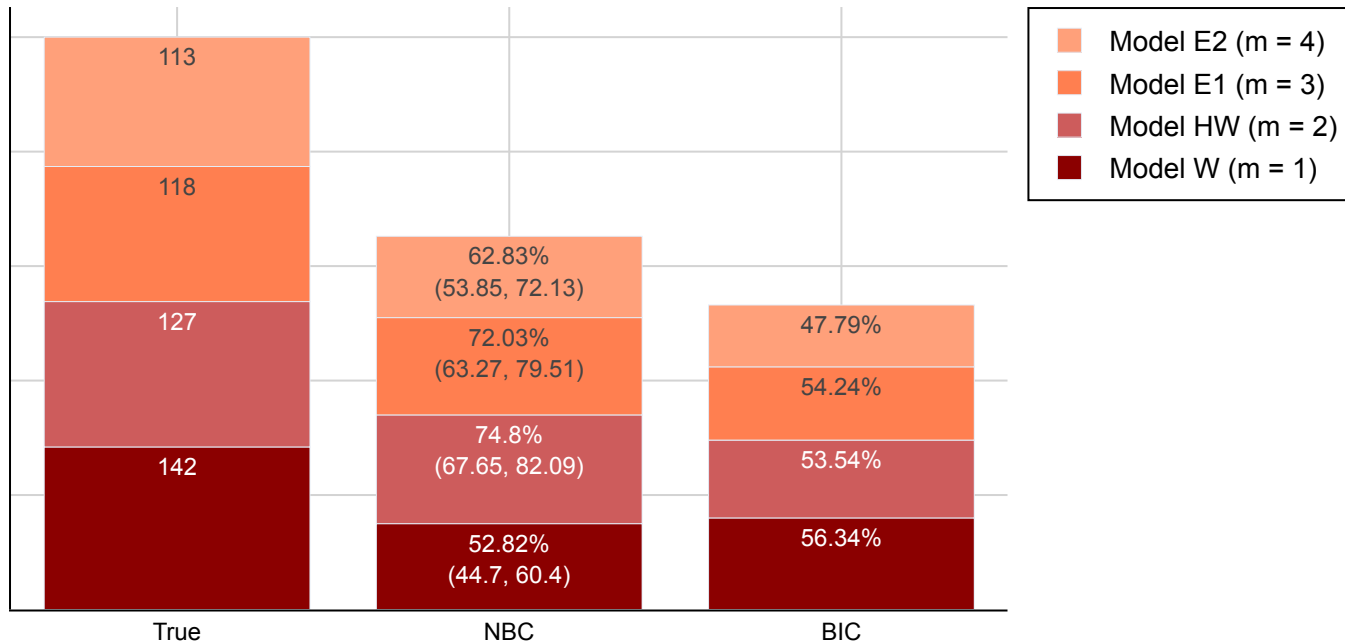

Supplement: Supplementary file 1 — (zip 5334 KB) [file 10687_2025_521_MOESM1_ESM.zip › SupplementaryMaterial/Images/multiclass_n200.pdf]

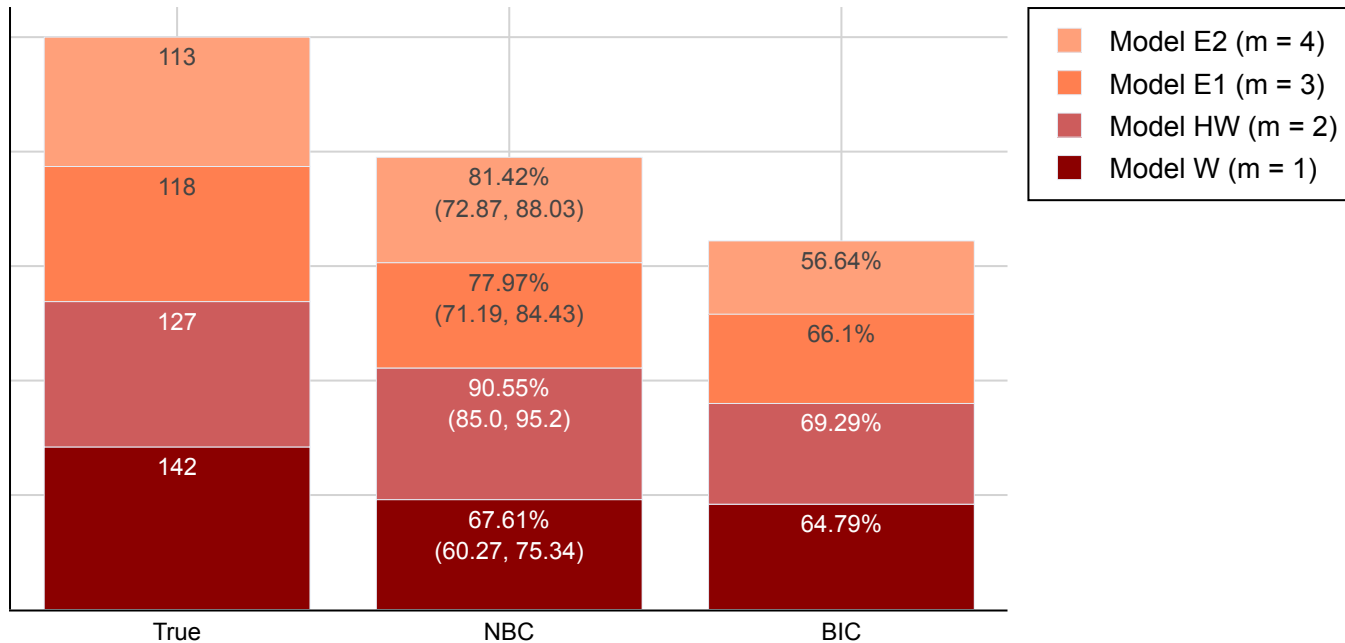

Supplement: Supplementary file 1 — (zip 5334 KB) [file 10687_2025_521_MOESM1_ESM.zip › SupplementaryMaterial/Images/multiclass_n2000.pdf]

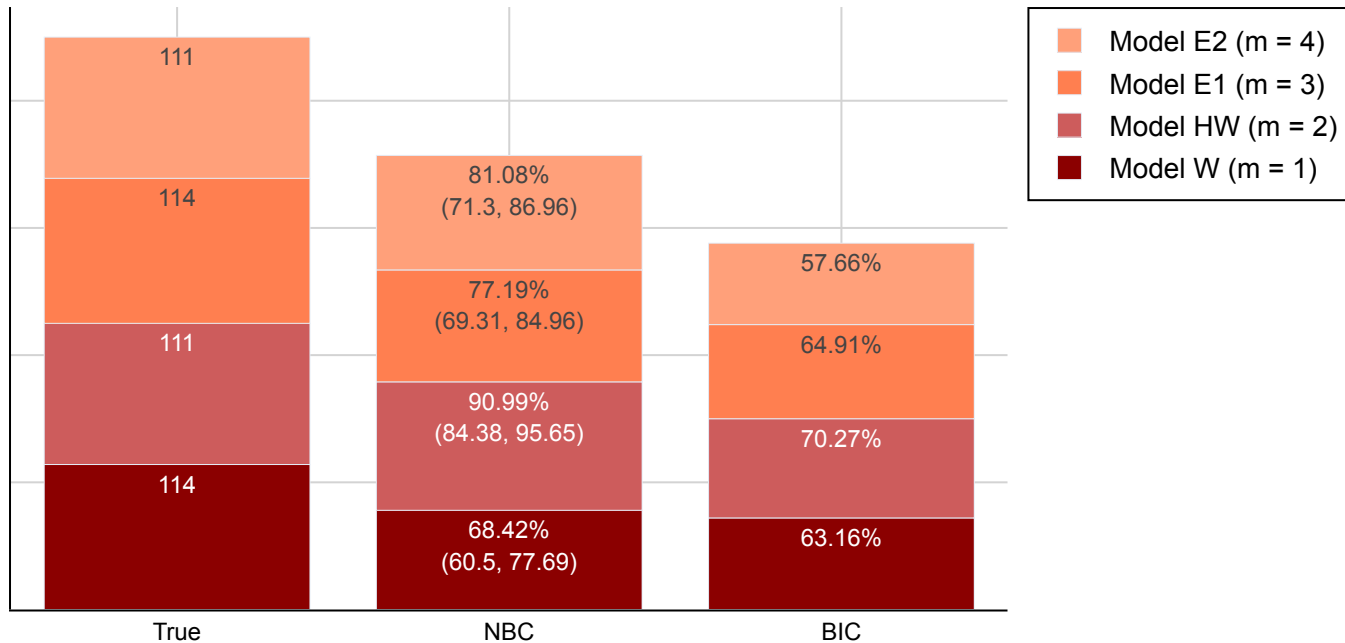

Supplement: Supplementary file 1 — (zip 5334 KB) [file 10687_2025_521_MOESM1_ESM.zip › SupplementaryMaterial/Images/multiclass_n2000_1_450.pdf]

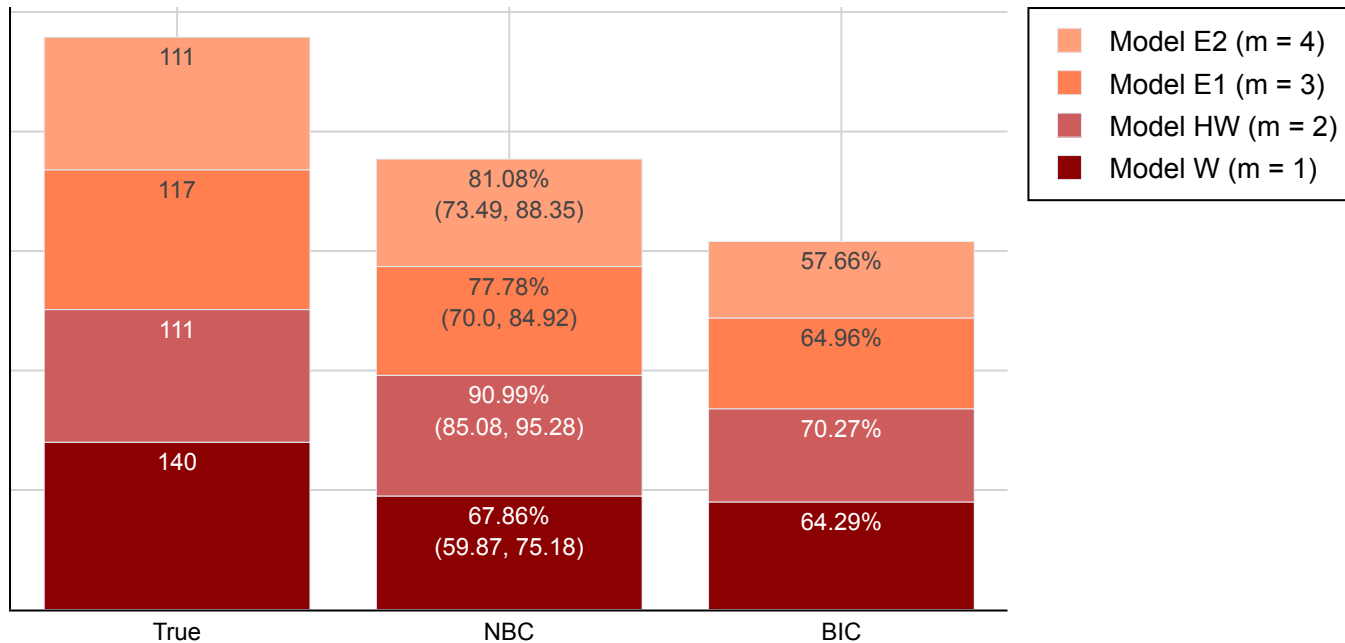

Supplement: Supplementary file 1 — (zip 5334 KB) [file 10687_2025_521_MOESM1_ESM.zip › SupplementaryMaterial/Images/multiclass_n2000_1_479.pdf]

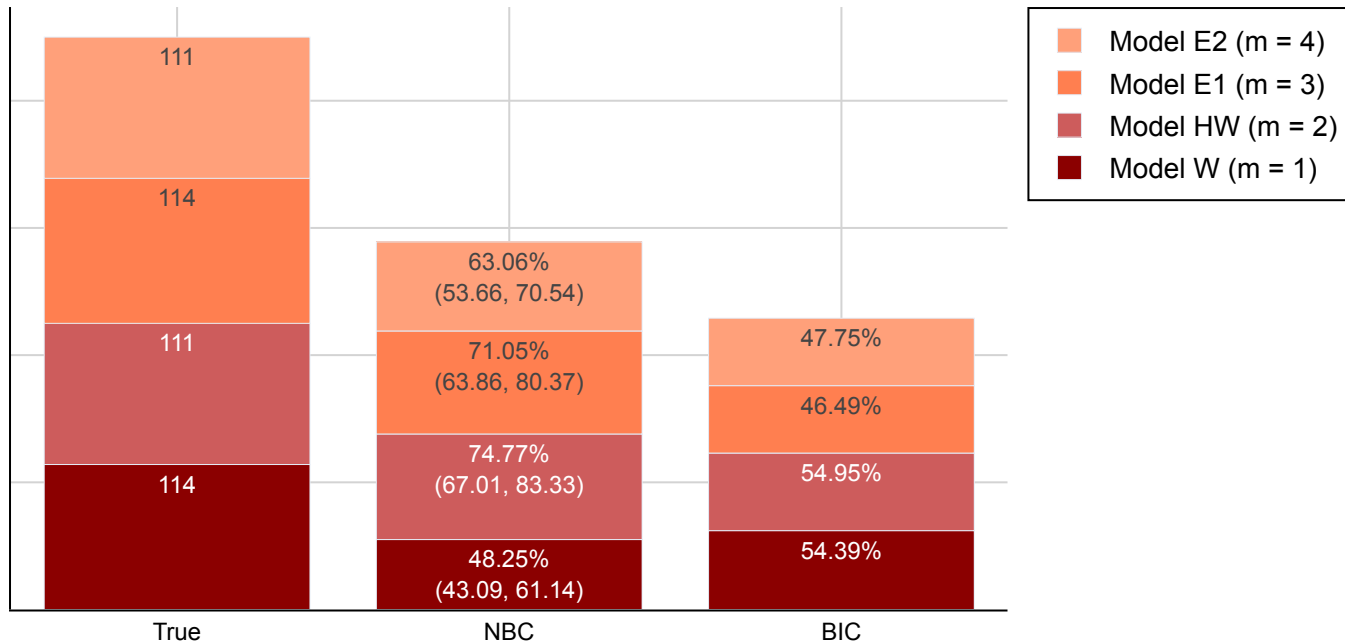

Supplement: Supplementary file 1 — (zip 5334 KB) [file 10687_2025_521_MOESM1_ESM.zip › SupplementaryMaterial/Images/multiclass_n200_1_450.pdf]

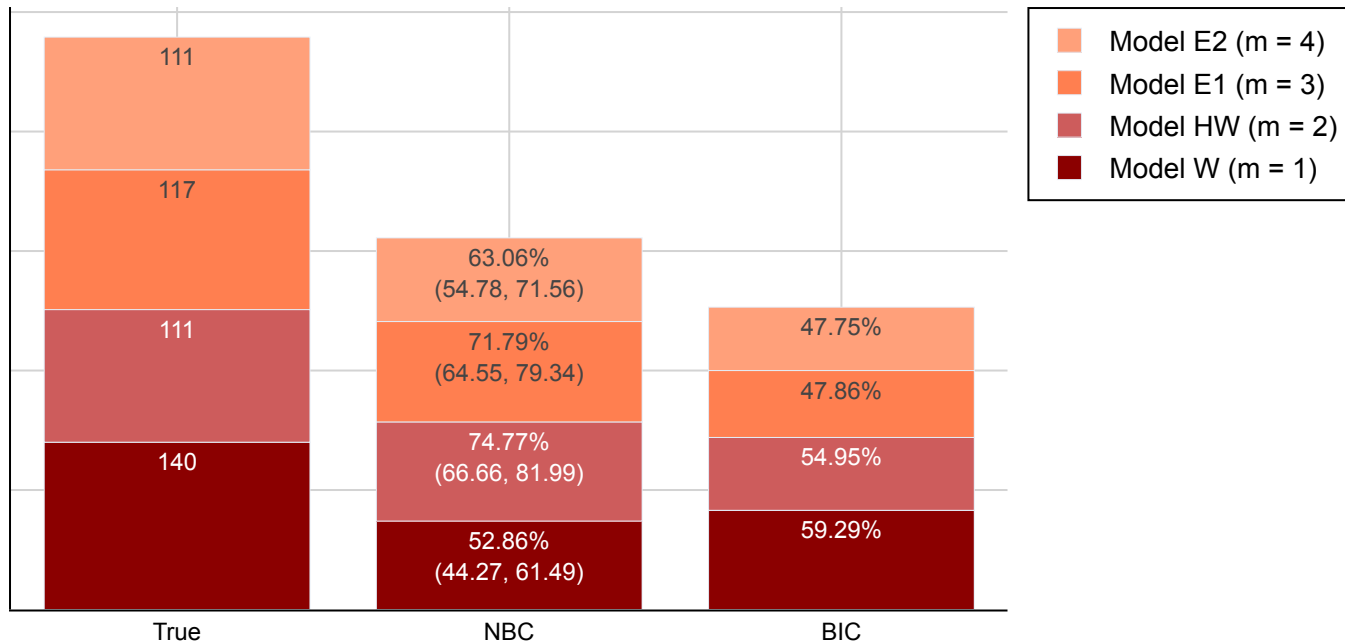

Supplement: Supplementary file 1 — (zip 5334 KB) [file 10687_2025_521_MOESM1_ESM.zip › SupplementaryMaterial/Images/multiclass_n200_1_479.pdf]

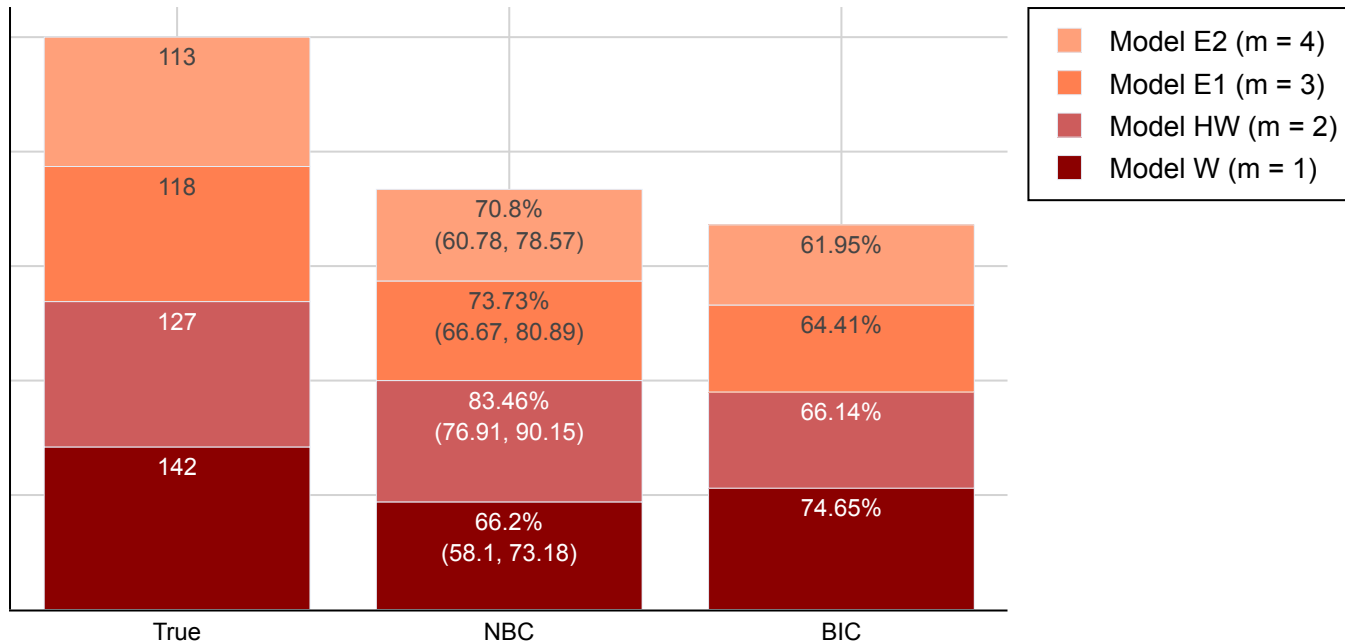

Supplement: Supplementary file 1 — (zip 5334 KB) [file 10687_2025_521_MOESM1_ESM.zip › SupplementaryMaterial/Images/multiclass_n500.pdf]

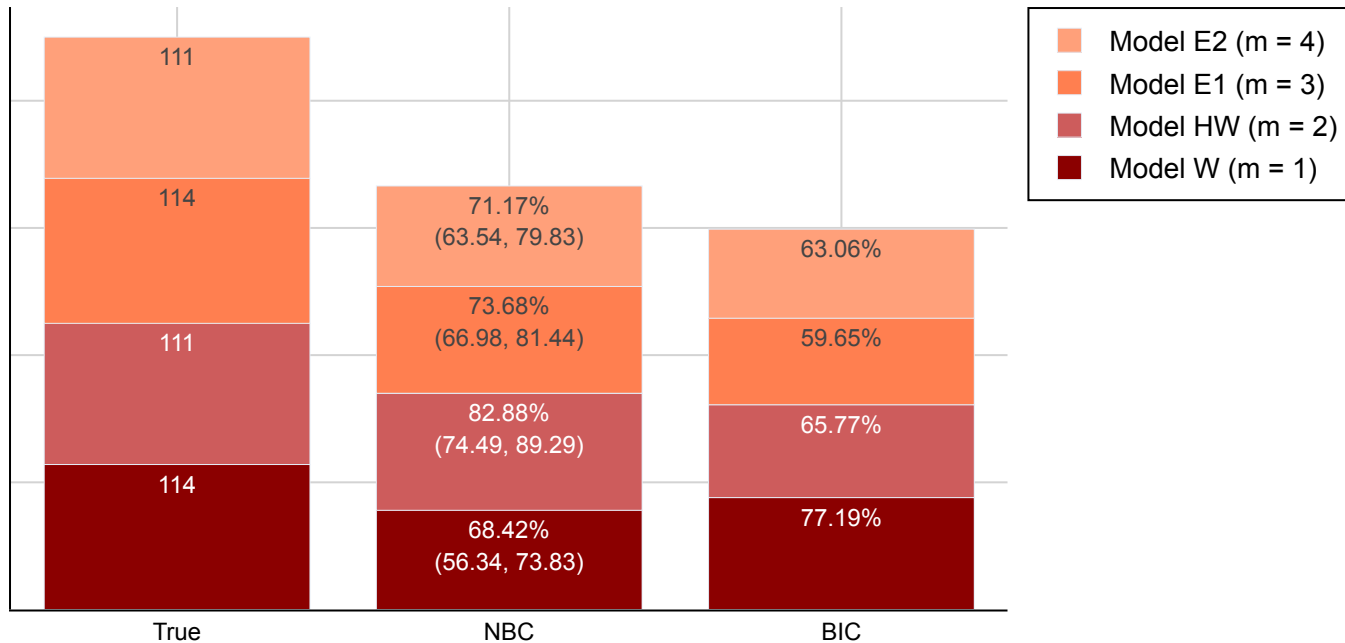

Supplement: Supplementary file 1 — (zip 5334 KB) [file 10687_2025_521_MOESM1_ESM.zip › SupplementaryMaterial/Images/multiclass_n500_1_450.pdf]

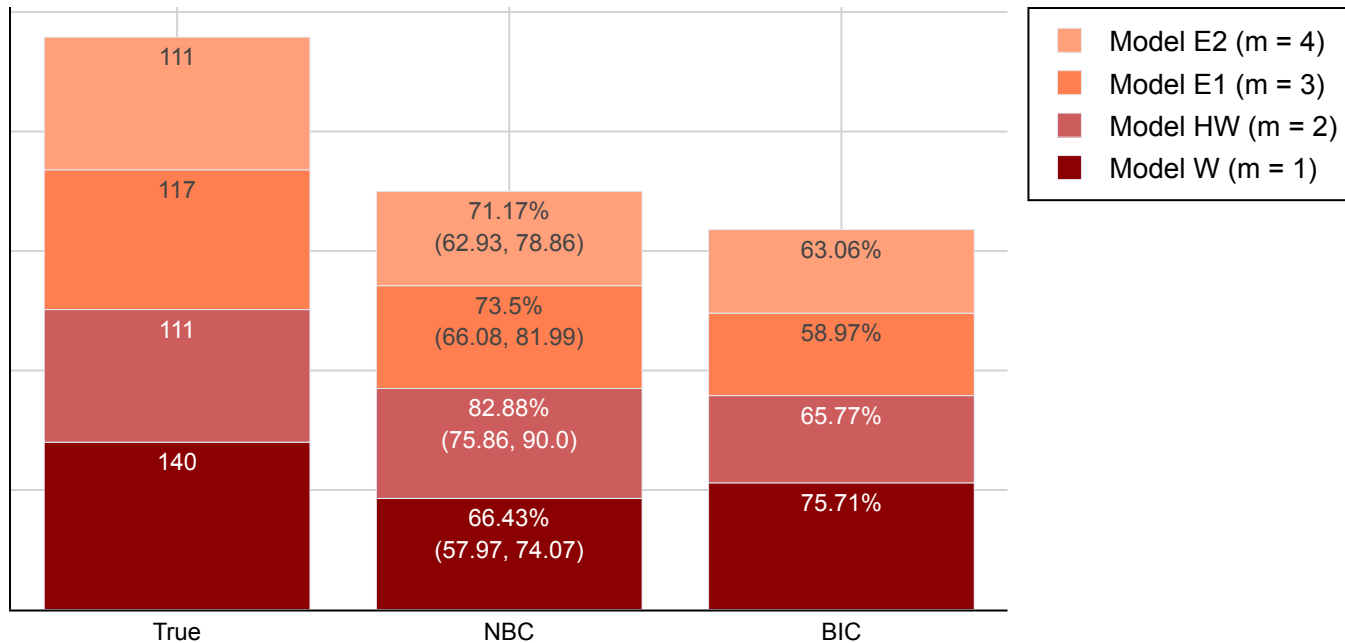

Supplement: Supplementary file 1 — (zip 5334 KB) [file 10687_2025_521_MOESM1_ESM.zip › SupplementaryMaterial/Images/multiclass_n500_1_479.pdf]
